# Assessing the causal influence of biomechanical factors on osteoporosis risk: A multivariable Mendelian randomization investigation

**DOI:** 10.1097/MD.0000000000049751

**Published:** 2026-07-24

**Authors:** Yong-Jun Dai, Xian-Pei Xiao, Hong-Xu Li, Jun-Jie Mao, Yin-Fei Luo, Bi-Yuan Qin

**Affiliations:** a Department of Orthopedics, Luojiang District People’s Hospital of Deyang City, Deyang, Sichuan Province, China; b Department of Critical Care Medicine, Luojiang District People’s Hospital of Deyang City, Deyang, Sichuan Province, China; c School of Clinical Medicine, Chengdu Medical College, Chengdu, Sichuan Province, China; d Department of Science and Education, Deyang People’s Hospital, Deyang, Sichuan Province, China.

**Keywords:** ankle spacing width, biomechanics, bone mineral density, Mendelian randomization

## Abstract

While observational studies indicate correlations between multiple biomechanical traits and osteoporosis, causal inference has been constrained, impeding the clinical application of these measures in risk stratification. This Mendelian randomization (MR) study examines causal associations of ankle spacing width (ASW), height, body mass index (BMI), grip strength, and usual walking pace with bone mineral density (BMD) across skeletal sites and age groups. Genetic proxies for the exposures were derived from the Medical Research Council Integrative Epidemiology Unit and UK Biobank resources. Genetic association estimates for the outcomes were acquired from the Genetic Factors for Osteoporosis Consortium. Two-sample and multivariable MR analyses were applied to infer causality. Genetically proxied ASW (encompassing left, right, and combined measures) and height showed an inverse association with estimated BMD (eBMD; Beta < 0, *P*. adjusted < .05). In contrast, genetically predicted BMI was associated with an increase in eBMD (Beta > 0, *P*. adjusted < .05). Moreover, genetically predicted ASW was linked to lower total body BMD, with the inverse association being most pronounced in the 0 to 15 years age group. Multivariable MR analyses confirmed that ASW remained independently associated with both eBMD and total body BMD after adjusting for confounding factors. Our findings offer genetic evidence indicative of a causal detrimental effect of ASW and height on BMD, especially during early life stages, whereas BMI demonstrates a potentially protective causal role.

## 1. Introduction

Osteoporosis (OP) is a prevalent metabolic bone disorder characterized by reduced bone mineral density (BMD), which predisposes individuals to pain, functional impairment, and an increased risk of fragility fractures.^[1]^ It represents a major public health challenge worldwide, causing significant morbidity, mortality, and socioeconomic burden, with an estimated 9 million osteoporotic fractures occurring annually.^[2]^ Despite its status as the diagnostic gold standard, dual-energy X-ray absorptiometry (DEXA)-measured BMD inadequately assesses overall fracture risk. This limitation is particularly evident in patients who sustain fractures despite BMD values above the osteoporotic threshold.^[3]^ This gap underscores the need for complementary biomarkers that better reflect biomechanical vulnerability and improve risk stratification.

Certain biomechanical and anthropometric traits – including ankle spacing width (ASW), height, body mass index (BMI), grip strength, and customary walking pace – have received interest as potential markers for skeletal health status. These traits collectively capture distinct dimensions of skeletal structure, mechanical loading, and neuromuscular function, which are theoretically linked to bone integrity. Observational studies suggest that these factors may influence bone quality, fall propensity, and fracture risk.^[4,5]^ For instance, ASW may reflect cumulative mechanical loading and bone adaptation over the lifespan, whereas muscle-related traits (e.g., grip strength and gait speed) are associated with both neuromuscular stability and skeletal strength. Nevertheless, conventional observational designs cannot fully address confounding or reverse causation, leaving the causal roles of these factors unresolved.

Mendelian randomization (MR) offers a robust causal inference framework that uses genetic variants as instrumental variables (IVs), thereby mitigating biases from residual confounding and reverse causation. This study employs MR to examine the causal effects of ASW and other biomechanical and anthropometric traits on site- and age-specific BMD. We aim to provide genetic evidence that may refine risk stratification and inform early-life intervention strategies for OP, particularly across different age windows.

## 2. Methods

### 2.1. Study design

**Figure 1.**
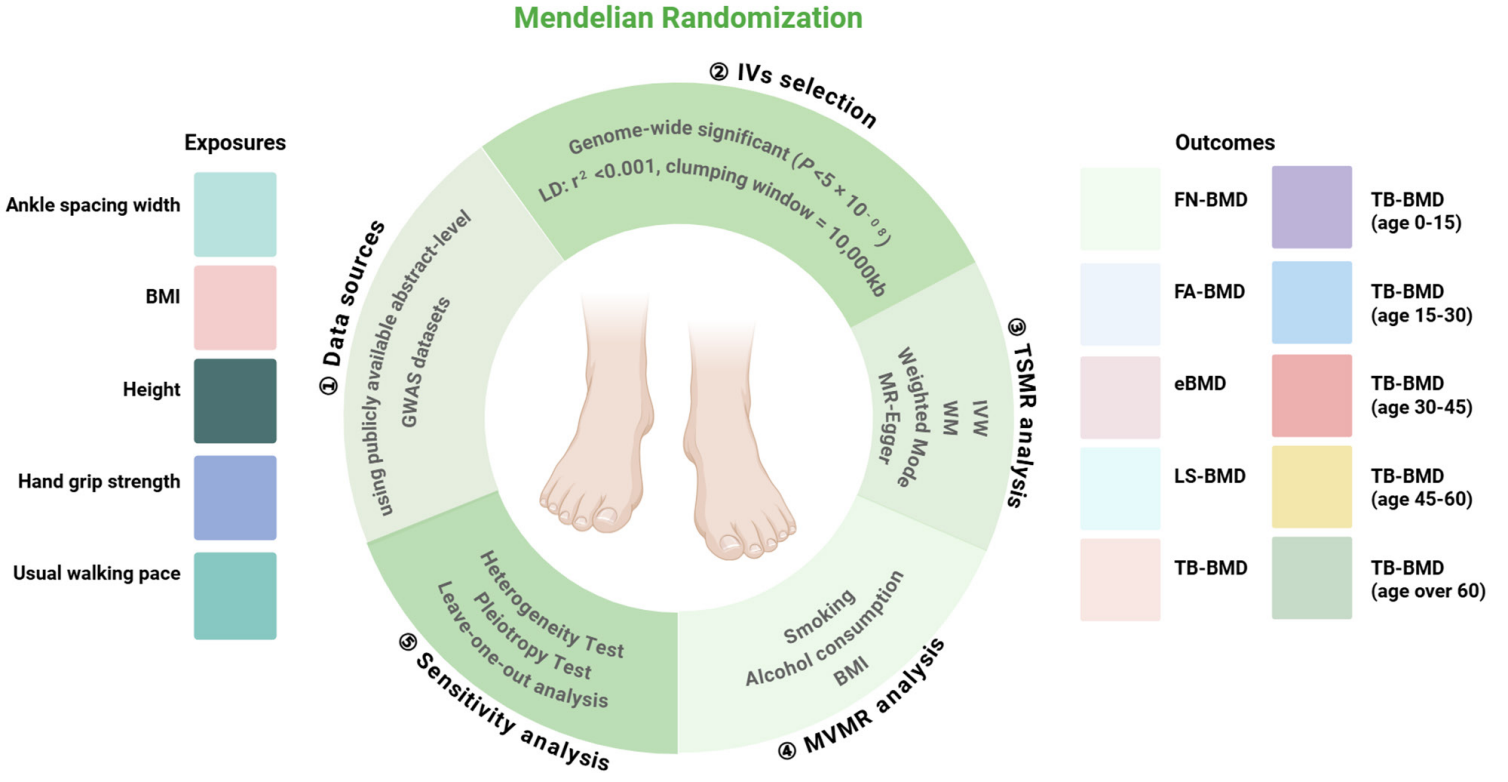
The flowchart of MR study design. BMD = bone mineral density, BMI = body mass index, eBMD = estimated from quantitative heel ultrasounds BMD, FA = forearm, FN = femoral neck, GWAS = genome-wide association study, IVs = instrumental variables, IVW = inverse-variance weighted, LD = linkage disequilibrium, LS = lumbar spine, MR = Mendelian randomization, TB-BMD = total body BMD, WM = weighted median.

We conducted a two-sample and multivariable MR analysis to evaluate the causal associations of 5 biomechanical/anthropometric traits – ASW, height, BMI, grip strength, and usual walking pace – with estimated BMD (eBMD) and total body BMD (TB-BMD), with age-stratified analyses in the 0 to 15, 15 to 30, 30 to 45, 45 to 60, and ≥60 years subgroups. We used publicly available GWAS summary statistics. Detailed information on the exposure and outcome GWAS datasets, including sample sizes, ancestry, and data sources, is provided in Supplementary Table 1, Supplemental Digital Content 2. Every originating project had received ethics approval from appropriate boards, with participants giving informed consent. The Bonferroni method was used to correct for multiple comparisons. This study was reported in accordance with the STROBE-MR guidelines (Supplementary Text 1, Supplemental Digital Content 1),^[6,7]^ and a schematic of the study design is presented in Figure 1.

### 2.2. Data sources

IVs for ASW (analyzed separately for left and right sides, detailed in Supplementary Text 1, Supplemental Digital Content 1), height, BMI, grip strength (left and right separately), and usual walking pace were obtained from the IEU Open GWAS platform (https://gwas.mrcieu.ac.uk) and the UK Biobank.^[8]^ These datasets are based on large-scale European-ancestry populations with standardized phenotypic measurements.

BMD served as the primary outcome, representing OP-related phenotypes. Summary-level genetic data for site-specific BMD assessments were acquired, including lumbar spine, forearm, femoral neck, eBMD, and TB-BMD, as well as age-specific TB-BMD (Supplementary Table 1, Supplemental Digital Content 2). Age-specific TB-BMD was categorized as ≤15, 15 to 30, 30 to 45, 45 to 60, and ≥60 years, in accordance with the age groupings used in the original GWAS publications from the Genetic Factors for Osteoporosis Consortium. These intervals were chosen to reflect critical periods in bone development, peak bone mass acquisition, and subsequent bone loss.^[9–11]^ DEXA was used for all BMD measurements except for eBMD, which was derived from quantitative ultrasonography. All datasets underwent standardization on the Genetic Factors for Osteoporosis Consortium portal to ensure uniformity in allele encoding and effect directions.

### 2.3. IVs selection and statistical analysis

In MR analysis, single-nucleotide polymorphisms (SNPs) were used as IVs to evaluate the causal effects of biomechanical traits, including ASW, height, BMI, grip strength, and usual walking pace, on OP-related outcomes.^[6]^ The selection of genetic instruments adhered to 3 core MR assumptions. First, adhering to the relevance assumption, SNPs exhibiting genome-wide significance (*P* < 5 × 10^−08^) for association with each exposure trait were selected. Linkage disequilibrium pruning was performed using stringent parameters (*r*^2^ < 0.001, within a 10,000 kb window) based on European reference panels to ensure independence among instruments.^[12]^ Second, to meet the independence assumption, IVs linked to potential confounding variables (such as BMI, smoking, and alcohol consumption) were systematically identified and removed using the PhenoScanner database.^[13]^ This step aimed to minimize bias from pleiotropic pathways related to known risk factors. Third, concerning the exclusion restriction assumption (i.e., IVs influence the outcome solely via the exposure), the MR-PRESSO test was implemented to detect and correct for horizontal pleiotropy.^[14]^ To mitigate weak instrument bias, we computed the *F*-statistic for each SNP using the formula:


$$F=R^{2}\times \left(N-2\right)/\left(1-R^{2}\right),$$

where *R*^2^ represents the proportion of variance in the exposure explained by the SNP.^[15]^ Only instruments with *F*-statistics > 10 were retained, ensuring sufficient strength for reliable causal estimation. Finally, the Bonferroni correction was used to reduce the likelihood of false-positive results by adjusting the significance threshold for the *P* value.

For the primary two-sample MR analyses, the inverse-variance weighted (IVW) method delivers the most accurate results when all MR assumptions are strictly satisfied – specifically, no pleiotropy, valid instruments, and confounder independence. In contrast, the weighted median (WM) method addresses horizontal pleiotropy by assuming that a majority (>50%) of the IVs remain valid.^[16,17]^ Therefore, the results that are consistently supported by both the IVW and WM methods are considered to indicate statistically significant differences.

Multivariable MR analyses were performed to estimate the direct effect of each exposure independent of the others, adjusting for BMI, smoking, and alcohol consumption – covariates selected based on their established biological relevance to bone metabolism and data availability.^[18]^ The MR-PRESSO framework was employed to detect and correct for horizontal pleiotropy, remove outlier SNPs, estimate corrected causal effects, and further assess potential differences between the results before and after correction. Associations with a *P*-value < .05 were considered statistically significant.

### 2.4. Sensitivity analysis

To ensure the robustness of the results, several sensitivity analyses were performed. We employed MR-Egger regression to detect directional pleiotropy. Weighted mode estimation was also applied. Heterogeneity across SNPs was examined using the Cochran’s *Q* test. Additionally, a leave-one-out analysis assessed the impact of individual variants on causal estimates.^[19,20]^

## 3. Results

Following linkage disequilibrium clumping, 368 independent SNPs were significantly associated with ASW. This included 197 SNPs for left ASW and 212 SNPs for right ASW. Additionally, we identified 503 SNPs related to BMI, 157 for left-hand grip strength, 176 for right-hand grip strength, 256 for height, and 57 for usual walking pace. We used the PhenoScanner V2 tool (http://www.phenoscanner.medschl.cam.ac.uk)to query no SNPs overlapping with potential confounding factors. The *F*-statistics for the included IVs varied from 28.62 to 1426.17, indicating a low risk of weak instrument bias. Additionally, we computed the proportion of variance explained for each exposure: ASW accounted for 10.17%, left ASW for 8.10%, and right ASW for 8.46%. BMI contributed 5.37%, while left- and right-hand grip strength explained 1.62% and 1.79%, respectively. Height represented 16.22% of the variance, and usual walking pace accounted for 0.50% (Supplementary Table 2, Supplemental Digital Content 3).

### 3.1. Causal effects of ASW and biomechanical traits on site-specific BMD

**Figure 2.**
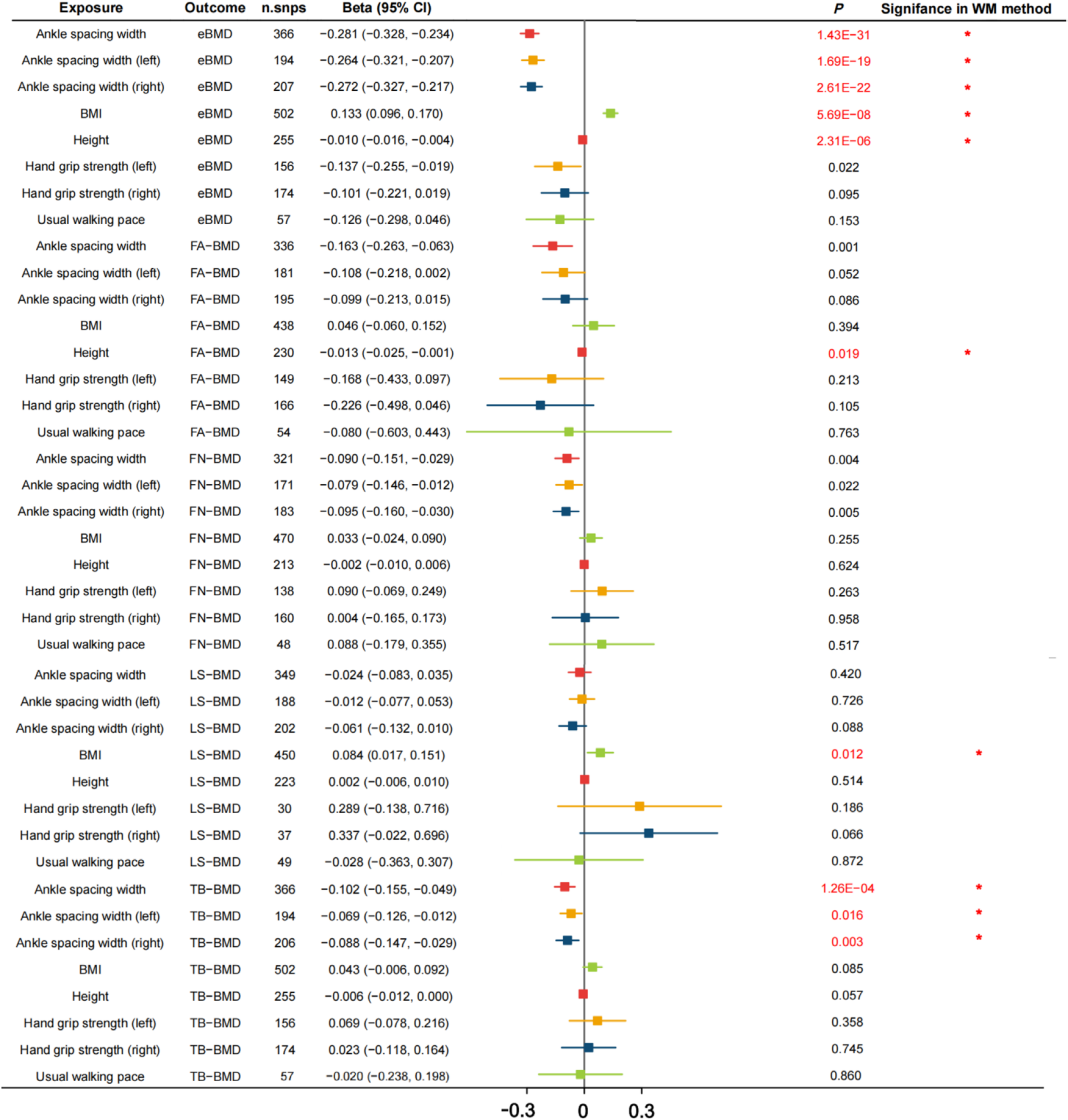
Forest plot of causal association of biomechanical measures on site-specific BMD with IVW and WM method. BMD = bone mineral density, BMI = body mass index, CI = confidence interval, eBMD = estimated from quantitative heel ultrasounds BMD, FA = forearm, FN = femoral neck, LS = lumbar spine, N. SNPs = number of single-nucleotide polymorphisms, TB-BMD = total body BMD, WM = weighted median.

**Figure 3.**
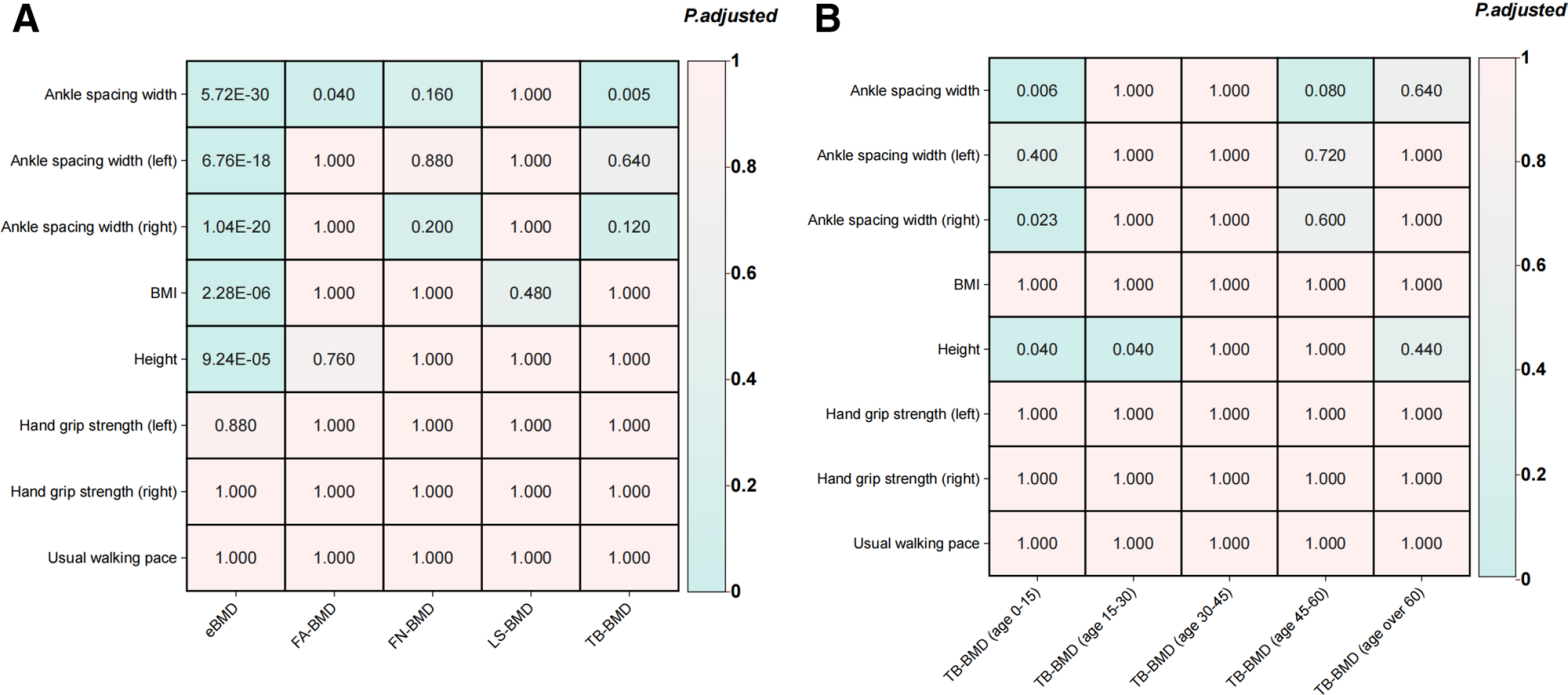
Bonferroni test adjusting results of causal association with biomechanical measures on BMD. (A) Bonferroni test adjusting results of causal association with biomechanical measures on sites-specific BMD. (B) Bonferroni test adjusting results of causal association with biomechanical measures on age-specific TB-BMD. BMD = bone mineral density, BMI = body mass index, eBMD = estimated from quantitative heel ultrasounds BMD, FA = forearm, FN = femoral neck, LS = lumbar spine, TB-BMD = total body BMD.

We evaluated the causal effects of biomechanical traits on site-specific BMD. Both the Bonferroni-corrected IVW method and the WM method provided strong evidence supporting a causal relationship between ASW and reduced eBMD (Beta = −0.281, 95% CI: −0.328, −0.234, *P* = 1.43 × 10^−31^). Notably, we also found significant causal associations between decreased eBMD and both left (Beta = −0.264, 95% CI: −0.321, −0.207, *P* = 1.69 × 10^−19^) and right ASW (Beta = −0.282, 95% CI: −0.327, −0.217, *P* = 2.61 × 10^−22^). Additionally, BMI demonstrated a protective effect on eBMD (Beta = 0.133, 95% CI: 0.096, 0.170, *P* = 5.69 × 10^−08^). In contrast, height was identified as a risk factor for both eBMD (Beta = 0.010, 95% CI: 0.004, 0.016, *P* = 2.31 × 10^−06^) and TB-BMD (Beta = −0.102, 95% CI: −0.155, −0.049, *P* = 1.26 × 10^−04^; Supplementary Table 3, Supplemental Digital Content 4 & Figs. 2 and 3).

### 3.2. Causal effects of ASW and biomechanical traits on age-specific BMD

Age-stratified analyses indicated a putative causal effect of ASW on TB-BMD specifically within the 0 to 15-year age group (Beta: −0.161, 95% CI: −0.245, −0.077, *P* = 1.56 × 10^−04^; Table 1). The Bonferroni-corrected *P* value was <.05. No significant horizontal pleiotropy was observed in the sensitivity analysis. Furthermore, in populations aged 15 to 30, 30 to 45, 45 to 60, and >60 years, no evidence of a causal relationship between biomechanical factors and changes in TB-BMD was observed (Table 1).

**Table 1:** Causal association with biomechanical factors on age-specific BMD.

Exposure	Outcome	SNP	IVW			Weighted methods				MR-Egger regression				
						Weighted median		Weighted Mode		MR-Egger		Intercept		
			Beta (SE)	*P* value	*P* heterogeneity	Beta (SE)	*P* value	Beta (SE)	*P* value	Beta (SE)	*P* value	Intercept	SE	*P* value
Ankle spacing width	TB-BMD (age 0–15)	366	−0.161 (0.043)	1.56E−04	4.29E−08	−0.162 (0.057)	.004	−0.202 (0.145)	.163	−0.044 (0.164)	.789	1.21E−02	5.48E−03	.029
Ankle spacing width (left)	TB-BMD (age 0–15)	192	−0.117 (0.045)	.010	2.66E−03	−0.049 (0.101)	.629	−0.053 (0.206)	.797	−0.127 (0.106)	.231	9.48E−04	3.65E−03	.795
Ankle spacing width (right)	TB-BMD (age 0–15)	206	−0.147 (0.043)	5.69E−04	8.21E−03	−0.112 (0.067)	.095	−0.154 (0.126)	.222	−0.158 (0.115)	.171	6.70E−03	5.39E−03	.215
BMI	TB-BMD (age 0–15)	502	0.008 (0.043)	.848	2.63E−02	0.053 (0.071)	.456	0.166 (0.135)	.218	0.064 (0.115)	.578	−1.00E−03	0.002	.601
Height	TB-BMD (age 0–15)	255	−0.017 (0.005)	.001	2.93E−12	−0.020 (0.006)	.001	−0.025 (0.012)	.043	−0.041 (0.014)	.005	0.006	0.004	.084
Hand grip strength (left)	TB-BMD (age 0–15)	156	0.156 (0.113)	.167	.107	0.010 (0.163)	.951	−0.432 (0.448)	.337	0.526 (0.450)	.245	−0.004	0.005	.398
Hand grip strength (right)	TB-BMD (age 0–15)	174	0.044 (0.122)	.717	.001	−0.093 (0.157)	.553	−0.554 (0.401)	.169	0.503 (0.476)	.292	−0.005	0.005	.320
Usual walking pace	TB-BMD (age 0–15)	56	0.065 (0.219)	.765	.508	0.081 (0.315)	.797	0.092 (0.683)	.893	−0.307 (0.961)	.750	0.003	0.009	.692
Ankle spacing width	TB-BMD (age 15–30)	365	−0.005 (0.065)	.940	1.17E−01	−0.165 (0.051)	.001	−0.148 (0.107)	.166	−0.037 (0.086)	.666	−4.24E−03	3.49E−03	.226
Ankle spacing width (left)	TB-BMD (age 15–30)	192	−0.001 (0.070)	.984	5.35E−01	−0.085 (0.045)	.060	−0.042 (0.099)	.673	−0.217 (0.096)	.025	−8.31E−04	2.37E−03	.726
Ankle spacing width (right)	TB-BMD (age 15–30)	206	−0.060 (0.068)	.379	5.87E−01	−0.103 (0.065)	.111	−0.281 (0.222)	.208	−0.396 (0.193)	.042	−3.93E−03	3.37E−03	.245
BMI	TB-BMD (age 15–30)	500	0.008 (0.043)	.848	9.51E−01	0.053 (0.071)	.456	0.166 (0.135)	.218	0.064 (0.115)	.578	2.00E−03	0.003	.439
Height	TB-BMD (age 15–30)	254	−0.017 (0.005)	.001	.001	−0.020 (0.006)	.001	−0.025 (0.012)	.043	−0.041 (0.014)	.005	−0.006	0.006	.312
Hand grip strength (left)	TB-BMD (age 15–30)	153	0.156 (0.113)	.167	.066	0.010 (0.163)	.951	−0.432 (0.448)	.337	0.526 (0.45)	.245	0.007	0.010	.449
Hand grip strength (right)	TB-BMD (age 15–30)	170	0.044 (0.122)	.717	.134	−0.093 (0.157)	.553	−0.554 (0.401)	.169	0.503 (0.476)	.292	−0.001	0.009	.866
Usual walking pace	TB-BMD (age 15–30)	28	0.065 (0.219)	.765	.288	0.081 (0.315)	.797	0.092 (0.683)	.893	−0.307 (0.961)	.75	0.019	0.022	.394
Ankle spacing width	TB-BMD (age 30–45)	365	−0.075 (0.046)	.105	8.02E−05	−0.022 (0.115)	.851	−0.077 (0.239)	.746	−0.022 (0.123)	.858	2.17E−03	3.85E−03	.573
Ankle spacing width (left)	TB-BMD (age 30–45)	194	−0.059 (0.050)	.234	2.19E−02	−0.136 (0.071)	.053	−0.210 (0.140)	.135	−0.130 (0.135)	.337	2.02E−03	2.57E−03	.432
Ankle spacing width (right)	TB-BMD (age 30–45)	207	−0.065 (0.050)	.191	1.61E−03	−0.096 (0.050)	.058	−0.115 (0.099)	.247	−0.112 (0.099)	.258	2.67E−03	3.95E−03	.499
BMI	TB-BMD (age 30–45)	497	0.071 (0.051)	.165	0.741	0.003 (0.079)	.972	−0.096 (0.161)	.550	−0.030 (0.142)	.834	2.00E−03	0.002	.445
Height	TB-BMD (age 30–45)	248	0.006 (0.005)	.279	.387	0.004 (0.007)	.598	0.007 (0.018)	.691	−0.003 (0.015)	.829	0.002	0.004	.520
Hand grip strength (left)	TB-BMD (age 30–45)	153	0.089 (0.134)	.505	.633	0.158 (0.181)	.381	0.568 (0.587)	.334	0.157 (0.536)	.770	−0.001	0.006	.896
Hand grip strength (right)	TB-BMD (age 30–45)	168	0.049 (0.134)	.715	.922	−0.046 (0.185)	.804	0.571 (0.561)	.310	−0.541 (0.515)	.294	0.007	0.006	.237
Usual walking pace	TB-BMD (age 30–45)	56	0.140 (0.305)	.647	.335	−0.004 (0.384)	.991	−0.133 (0.881)	.881	−0.222 (1.313)	.866	0.003	0.012	.778
Ankle spacing width	TB-BMD (age 45–60)	364	−0.118 (0.039)	.002	4.26E−17	−0.008 (0.050)	.874	−0.066 (0.102)	.518	−0.188 (0.115)	.103	2.21E−03	2.82E−03	.434
Ankle spacing width (left)	TB-BMD (age 45–60)	193	−0.099 (0.042)	.018	6.56E−08	−0.132 (0.061)	.031	−0.135 (0.216)	.533	−0.281 (0.191)	.142	−1.14E−03	1.93E−03	.556
Ankle spacing width (right)	TB-BMD (age 45–60)	206	−0.103 (0.043)	.015	1.81E−11	−0.042 (0.106)	.695	−0.206 (0.228)	.367	−0.018 (0.119)	.881	−3.71E−04	2.99E−03	.902
BMI	TB-BMD (age 45–60)	502	0.047 (0.037)	.206	9.01E−05	0.048 (0.054)	.370	−0.247 (0.145)	.089	0.034 (0.1)	.734	1.00E−03	0.002	.885
Height	TB-BMD (age 45–60)	255	0.003 (0.004)	.530	4.98E−14	0.001 (0.005)	.953	−0.005 (0.015)	.732	−0.010 (0.012)	.402	0.003	0.003	.256
Hand grip strength (left)	TB-BMD (age 45–60)	156	0.220 (0.113)	.051	2.87E−07	0.131 (0.138)	.342	−0.332 (0.434)	.446	0.157 (0.448)	.727	0.001	0.005	.883
Hand grip strength (right)	TB-BMD (age 45–60)	174	0.117 (0.11)	.289	.001	0.082 (0.129)	.523	−0.412 (0.43)	.340	0.207 (0.427)	.629	−0.001	0.005	.828
Usual walking pace	TB-BMD (age 45–60)	57	−0.081 (0.207)	.696	.052	0.044 (0.267)	.869	0.114 (0.545)	.835	0.888 (0.878)	.316	−0.009	0.008	.261
Ankle spacing width	TB-BMD (age over 60)	366	−0.084 (0.035)	.016	1.14E−14	−0.143 (0.073)	.049	−0.221 (0.142)	.120	−0.153 (0.139)	.272	2.72E−03	3.26E−03	.405
Ankle spacing width (left)	TB-BMD (age over 60)	194	−0.040 (0.036)	.271	8.59E−06	−0.092 (0.052)	.080	−0.082 (0.108)	.445	−0.053 (0.105)	.616	2.42E−03	2.16E−03	.263
Ankle spacing width (right)	TB-BMD (age over 60)	207	−0.065 (0.038)	.086	4.61E−10	−0.045 (0.049)	.357	−0.036 (0.099)	.716	−0.162 (0.119)	.175	1.78E−03	3.38E−03	.598
BMI	TB-BMD (age over 60)	502	0.045 (0.036)	.213	6.31E−09	−0.011 (0.052)	.826	−0.100 (0.136)	.461	0.100 (0.096)	.300	−1.00E−03	0.002	.538
Height	TB-BMD (age over 60)	253	−0.010 (0.004)	.011	5.67E−17	−0.018 (0.005)	.001	−0.018 (0.013)	.163	−0.021 (0.012)	.074	0.003	0.003	.339
Hand grip strength (left)	TB-BMD (age over 60)	156	−0.126 (0.103)	.221	4.30E−07	−0.074 (0.127)	.559	0.032 (0.282)	.909	0.076 (0.412)	.855	−0.002	0.005	.614
Hand grip strength (right)	TB-BMD (age over 60)	174	−0.041 (0.089)	.649	.001	−0.022 (0.120)	.854	0.014 (0.308)	.964	−0.339 (0.350)	.334	0.004	0.004	.379
Usual walking pace	TB-BMD (age over 60)	57	−0.090 (0.190)	.635	.047	0.163 (0.244)	.503	0.199 (0.515)	.700	−1.652 (0.800)	.044	0.015	0.007	.050

BMD = bone mineral density, BMI = body mass index, IVW = inverse-variance weighted, SE = standard error, SNP = single-nucleotide polymorphism, TB-BMD = total body BMD.

### 3.3. Direct causal effects of ASW and biomechanical traits on BMD

Previous studies have indicated associations between BMD and various factors, including smoking, alcohol consumption, BMI, and height. We performed multivariable MR analysis to examine the direct influence of ASW on the risk of eBMD and TB-BMD. After accounting for covariates including BMI, height, smoking, and alcohol intake, a strong inverse relationship with eBMD and TB-BMD remained (eBMD: Beta = −0.351, 95% CI: −0.407, −0.294, *P* = 7.05 × 10^−34^; TB-BMD: Beta = −0.152, 95% CI: −0.221, −0.082, *P* = 1.82 × 10^−05^). This finding was consistent in a subgroup of participants aged 60 years or older (Beta = −0.142, 95% CI: −0.241, −0.042, *P* = 1.82 × 10^−05^), as shown in Table 2.

**Table 2:** Direct IVW results of biomechanical factors on eBMD and TB-BMD.

Exposure	Confounders	Outcome	SNP	*P* value	OR (95% CI)
Ankle spacing width	BMI	eBMD	160	7.05E−34	−0.351 (−0.407, −0.294)
	Height				
	Alcoholic drinks per week	TB-BMD	160	1.82E−05	−0.152 (−0.221, −0.082)
	Smoking initiation	TB-BMD (0–15)	160	0.012	−0.142 (−0.241, −0.042)

BMD = bone mineral density, BMI = body mass index, eBMD = estimated from quantitative heel ultrasounds BMD, IVW = inverse-variance weighted, OR = odds ratio, SNP = single-nucleotide polymorphism, TB-BMD = total body BMD.

## 4. Discussion

This study provides novel genetic evidence supporting the causal effects of multiple biomechanical factors – particularly ASW, height, BMI, grip strength, and walking pace – on BMD. The robust and consistent ASW-BMD association supports its potential as a practical biomarker for OP management. These findings carry significant implications for real-world practice, suggesting that straightforward biomechanical assessments, especially ASW measurement, could substantially enhance individualized OP risk stratification and prevention strategies in routine care. By integrating ASW into existing clinical algorithms, healthcare providers may better identify at-risk individuals – particularly older adults and those in resource-limited settings – who might otherwise remain undetected using conventional risk factors alone.^[21]^

Although the effect size of ASW on TB-BMD may appear modest at the individual level, it is comparable in magnitude to many established genetic and biochemical markers of bone metabolism. When translated to the population level, such a reduction in BMD is clinically meaningful. Moreover, the consistent and independent association of ASW with BMD across multiple sites and analytical approaches supports its potential utility as a complementary biomarker, particularly in settings where access to DEXA is limited. The significant causal effect of ASW on lower BMD observed in the youngest age group (0–15 years) suggests potential clinical relevance for early-life intervention.^[22,23]^ Unlike complex imaging modalities, ASW measurement requires minimal equipment and training, making it particularly suitable for pediatric screening and early intervention programs.^[24]^ The persistence of this association after adjustment for conventional risk factors suggests that ASW captures unique biomechanical information not reflected in current assessment tools, which could help identify children and adolescents with impaired bone development who might be missed by traditional DEXA-based screening, especially in resource-limited environments where access to DEXA remains limited. Furthermore, the significant association observed in the 0 to 15-year age group suggests that early-life ASW measurements might help identify individuals who would benefit from preventive lifestyle interventions before significant bone loss occurs.^[25]^ The absence of significant associations between ASW and BMD in the 15 to 30- and 30 to 45-year age groups may reflect distinct physiological processes governing bone remodeling across the lifespan. During early adolescence (0–15 years), skeletal development is characterized by rapid bone accrual and high plasticity, where mechanical loading – proxied by ASW – may exert a more direct and measurable influence on bone architecture and density. In contrast, young and middle adulthood represent a period of relative skeletal stability, during which bone turnover is balanced and less susceptible to biomechanical influences alone. This divergence suggests that ASW may serve as a biomarker predominantly during phases of active bone modeling or degradation, rather than during metabolic equilibrium. As such, the lack of association in these groups likely reflects a developmental-degenerative dichotomy: ASW may impact bone health primarily during growth and advanced age – periods of heightened bone turnover – rather than during stable adulthood.

The divergent effects of biomechanical factors – with BMI exhibiting a protective association with BMD, while height and ASW are associated with reduced BMD – underscore the multifactorial and complex nature of OP pathogenesis. These findings emphasize the importance of a comprehensive and integrated approach to OP risk assessment that incorporates multiple biomechanical and anthropometric measures.^[26]^ Although grip strength and usual walking pace did not demonstrate direct effects on BMD in this analysis, their established roles in maintaining physical function and preventing falls suggest that they remain relevant to holistic fracture risk evaluation, particularly in the context of fall-related injury prevention.^[27]^ The lack of a direct association with fractures, despite clear effects on BMD, suggests that ASW’s clinical utility may lie primarily in identifying individuals with poor bone quality rather than predicting individual fracture events. This is consistent with the multifactorial nature of fracture pathogenesis, where bone quality, fall risk, and other factors interact to determine ultimate fracture risk.^[28]^

Several limitations should be acknowledged. First, our study exclusively utilized data from individuals of European descent; thus, the generalizability of our findings to other populations is limited. Second, further research is needed to validate our results across diverse ethnic groups. Third, potential horizontal pleiotropy remains a fundamental concern in any MR study. To address this issue, we excluded results that exhibited evidence of horizontal pleiotropy, which may, in turn, have led to overlooking certain causal relationships. Additionally, due to the lack of detailed demographic data on BMD, the causal relationship between sex-specific BMD and ASW remains uncertain. However, these limitations do not diminish the potential clinical value of our findings, as ASW measurement remains feasible across diverse populations. While ASW is practical and heritable, future studies should include more direct biomechanical traits, such as joint loading forces or bone strain indices, as genetic data become available.

To the best of our knowledge, this study represents the first comprehensive investigation into the causal relationship between multiple biomechanical indicators and bone health from a lifelong genetic perspective. Our criteria for determining significance are strictly defined, requiring consistent support from both the IVW and WM methods. By restricting the entire sample to individuals of European ancestry, we minimized the potential bias arising from population stratification. Additionally, our selection of exposure variables based on quantitative indicators helps to minimize subjective bias. The inclusion of BMD measurements at specific anatomical sites and age groups provides meaningful insights into localized and age-specific effects on bone health. Notably, analyzing BMD across different age groups allows for a more nuanced understanding of how biomechanical indicators influence bone health over time, potentially identifying vulnerable populations and long-term implications.

Our research indicates that easily measurable biomechanical parameters can offer clinically relevant insights into bone health status. The consistent inverse association between ASW and BMD across multiple analytical methods, combined with the practicality of its measurement, supports the incorporation of ASW assessment as an adjunct tool in OP management. Future implementation research should focus on evaluating the cost-effectiveness of integrating ASW into existing screening frameworks and on further exploring its utility in refining risk stratification and guiding intervention strategies across varied clinical contexts.

## 5. Conclusion

This study provides robust genetic evidence indicative of a causal detrimental effect of ASW and height on BMD, most notably in early life, contrasting with a putatively protective causal role for BMI. These associations persist after adjustment for lifestyle factors, supporting the potential utility of ASW as an auxiliary biomarker for OP risk stratification. Further clinical validation is warranted to integrate these biomechanical measures into preventive strategies.

## Author contributions

**Validation:** Yong-Jun Dai, Hong-Xu Li, Yin-Fei Luo.

**Visualization:** Yong-Jun Dai, Xian-Pei Xiao, Hong-Xu Li, Jun-Jie Mao.

**Supervision:** Yin-Fei Luo.

**Writing – original draft:** Xian-Pei Xiao.

**Writing – review & editing:** Yong-Jun Dai, Bi-Yuan Qin.








