# Supplementary material for: Assessing the causal influence of biomechanical factors on osteoporosis risk: A multivariable Mendelian randomization investigation
Source: Medicine (Baltimore). 2026 Jul 24;105(30):e49751. doi: 10.1097/MD.0000000000049751 (PMC13406190; doi:10.1097/MD.0000000000049751)
Supplement: Supplementary file 1 [file medi-105-e49751-s001.docx]

***Supplementary Text 1***

1. ***Definition of Ankle Spacing Width (ASW)***

Ankle Spacing Width (ASW) is defined as the linear distance between the most prominent medial and lateral bony points of the ankle joint. It is a direct anthropometric measure of the transverse diameter of the ankle mortise. Conceptually, ASW reflects the inherent morphological width of the ankle joint complex, which is influenced by the size and shape of the distal tibia, fibula, and talus. It is distinct from functional measures of ankle width that may change with weight-bearing or soft tissue swelling.

1. ***Anatomical Landmarks***

The measurement of ASW is based on two primary and easily identifiable bony landmarks.

- 1. ***Medial Malleolus (MM)***

This is the distal, subcutaneous prominence of the tibia. The specific point used for measurement is the most inferior and medial apex of the medial malleolus. It is typically easily palpated and often has a pyramidal shape.

- 1. ***Lateral Malleolus (LM)***

This is the distal, subcutaneous prominence of the fibula. It lies approximately 1-2 cm posterior and inferior to the medial malleolus. The specific point used for measurement is the most inferior and lateral apex of the lateral malleolus. It is generally more posteriorly located than the medial malleolus.

1. ***Standardized Measurement Protocol***

To ensure reproducibility and accuracy, ASW should be measured under a standardized protocol. The subject should be in a supine or seated position with the knee extended or slightly flexed. The ankle and foot must be in a relaxed, neutral position (approximately 90° of dorsiflexion/plantarflexion), with no inversion or eversion. The examiner palpates and precisely identifies the apex of MM and LM. The caliper tips are gently but firmly placed on these two bony landmarks. The caliper is held perpendicular to the long axis of the leg to ensure the measured distance is the true transverse diameter. The measurement should be repeated two to three times for each ankle, and the mean value should be used for analysis to reduce intra-observer error.
