# Supplementary material for: Assessing the causal influence of biomechanical factors on osteoporosis risk: A multivariable Mendelian randomization investigation
Source: Medicine (Baltimore). 2026 Jul 24;105(30):e49751. doi: 10.1097/MD.0000000000049751 (PMC13406190; doi:10.1097/MD.0000000000049751)
Supplement: Supplementary file 2 [file medi-105-e49751-s002.docx]

| **Supplementary table 1. Detailed in information regarding studies and datasets used in the present study** | | | | | | |
| --- | --- | --- | --- | --- | --- | --- |
| **Exposures / Outcomes** | **Population** | | **Sample size** | **Author / Consortium** | **category** | **GWAS ID** |
| Ankle spacing width | European | | 265,753 | MRC-IEU | Continuous | ukb-b-4080 |
| Ankle spacing width (left) | European | | 146,226 | MRC-IEU | Continuous | ukb-b-2122 |
| Ankle spacing width (right) | European | | 146,181 | MRC-IEU | Continuous | ukb-b-8607 |
| BMI | European | | 681,275 | MRC-IEU | Continuous | ieu-b-40 |
| Height | European | | 99,997 | Howe LJ | Continuous | ieu-b-4814 |
| Hand grip strength (left) | European | | 461,026 | MRC-IEU | Continuous | ukb-b-7478 |
| Hand grip strength (right) | European | | 461,089 | MRC-IEU | Continuous | ukb-b-10215 |
| Usual walking pace | European | | 459,915 | MRC-IEU | Continuous | ukb-b-4711 |
| FN-BMD | European | | 32,735 | GEFOS | Continuous | ieu-a-980 |
| FA-BMD | European | | 8,143 | GEFOS | Continuous | ieu-a-977 |
| eBMD | European | | 142,487 | GEFOS | Continuous | ebi-a-GCST006288 |
| LS-BMD | European | | 28,498 | GEFOS | Continuous | ieu-a-982 |
| TB-BMD | European | | 56,284 | Medina-Gomez C | Continuous | ebi-a-GCST005348 |
| TB-BMD (age 0-15) | European | | 11,807 | Medina-Gomez C | Continuous | ebi-a-GCST005345 |
| TB-BMD (age 15-30) | European | | 4,180 | Medina-Gomez C | Continuous | ebi-a-GCST005344 |
| TB-BMD (age 30-45) | European | | 10,062 | Medina-Gomez C | Continuous | ebi-a-GCST005346 |
| TB-BMD (age 45-60) | European | | 18,805 | Medina-Gomez C | Continuous | ebi-a-GCST005350 |
| TB-BMD (age over 60) | European | | 22,504 | Medina-Gomez C | Continuous | ebi-a-GCST005349 |
| Smoking | | European | 607,291 | MRC-IEU | Binary | ieu-b-4877 |
| Alcoholic drinks per week | | European | 335,394 | MRC-IEU | Binary | ieu-b-73 |

FN-BMD: Femoral neck bone mineral density; FA-BMD: Forearm bone mineral density; eBMD: Heel bone mineral density; LS-BMD: Lumbar spine bone mineral density; TB-BMD: total body bone mineral density.
