# Supplementary material for: Assessing the causal influence of biomechanical factors on osteoporosis risk: A multivariable Mendelian randomization investigation
Source: Medicine (Baltimore). 2026 Jul 24;105(30):e49751. doi: 10.1097/MD.0000000000049751 (PMC13406190; doi:10.1097/MD.0000000000049751)
Supplement: Supplementary file 3 [file medi-105-e49751-s003.docx]

| **Supplementary table 2. Single nucleotide polymorphisms (SNPs) associated with exposures and outcomes (used as IVs of Mendelian randomization)** | | | | | | | | | | | | |
| --- | --- | --- | --- | --- | --- | --- | --- | --- | --- | --- | --- | --- |
| Phenotype | SNP | Chr | Position | **Effect allele** | Other allele | EAF | Effect | Standard error | ***P*** value | N | **Variance explained** | *F*-statistic |
| Ankle spacing width (left) | rs7339938 | 1 | 2963493 | C | G | 1.91E-01 | 2.33E-02 | 3.94E-03 | 3.20E-09 | 146226 | 0.024% | 35.06 |
| Ankle spacing width (left) | rs112685832 | 1 | 155822629 | A | C | 1.14E-01 | 3.02E-02 | 4.92E-03 | 8.80E-10 | 146226 | 0.026% | 37.58 |
| Ankle spacing width (left) | rs10913200 | 1 | 176521655 | A | G | 2.83E-02 | -7.95E-02 | 9.25E-03 | 8.30E-18 | 146226 | 0.050% | 73.87 |
| Ankle spacing width (left) | rs2154319 | 1 | 41745770 | C | T | 2.17E-01 | 2.88E-02 | 3.77E-03 | 1.90E-14 | 146226 | 0.040% | 58.59 |
| Ankle spacing width (left) | rs4845852 | 1 | 11233559 | T | C | 7.40E-01 | -3.19E-02 | 3.50E-03 | 8.20E-20 | 146226 | 0.057% | 83.00 |
| Ankle spacing width (left) | rs111358652 | 1 | 51060775 | A | G | 2.81E-01 | -2.82E-02 | 3.43E-03 | 1.90E-16 | 146226 | 0.046% | 67.71 |
| Ankle spacing width (left) | rs28482086 | 1 | 75226252 | G | A | 6.57E-01 | 2.84E-02 | 3.25E-03 | 2.80E-18 | 146226 | 0.052% | 76.00 |
| Ankle spacing width (left) | rs12408387 | 1 | 119528329 | A | C | 3.05E-01 | 2.49E-02 | 3.34E-03 | 9.50E-14 | 146226 | 0.038% | 55.46 |
| Ankle spacing width (left) | rs56932405 | 1 | 176815743 | T | C | 1.21E-01 | -3.00E-02 | 4.73E-03 | 2.20E-10 | 146226 | 0.028% | 40.30 |
| Ankle spacing width (left) | rs11164632 | 1 | 103353900 | T | A | 6.07E-01 | -3.15E-02 | 3.17E-03 | 2.20E-23 | 146226 | 0.068% | 99.28 |
| Ankle spacing width (left) | rs2820323 | 1 | 201883160 | A | G | 3.49E-01 | 1.77E-02 | 3.23E-03 | 4.20E-08 | 146226 | 0.021% | 30.04 |
| Ankle spacing width (left) | rs6701211 | 1 | 214682808 | A | G | 8.15E-02 | -3.40E-02 | 5.65E-03 | 1.70E-09 | 146226 | 0.025% | 36.26 |
| Ankle spacing width (left) | rs11120513 | 1 | 215333982 | T | C | 6.73E-01 | -2.36E-02 | 3.28E-03 | 6.70E-13 | 146226 | 0.035% | 51.62 |
| Ankle spacing width (left) | rs1454376 | 2 | 33448665 | A | G | 5.20E-01 | -2.12E-02 | 3.10E-03 | 7.80E-12 | 146226 | 0.032% | 46.81 |
| Ankle spacing width (left) | rs7594766 | 2 | 42228804 | T | C | 2.33E-01 | -3.61E-02 | 3.65E-03 | 4.40E-23 | 146226 | 0.067% | 97.91 |
| Ankle spacing width (left) | rs10183783 | 2 | 145924502 | T | G | 4.47E-02 | -6.19E-02 | 7.45E-03 | 9.60E-17 | 146226 | 0.047% | 69.06 |
| Ankle spacing width (left) | rs700642 | 2 | 198600730 | G | T | 6.66E-01 | 1.86E-02 | 3.27E-03 | 1.30E-08 | 146226 | 0.022% | 32.34 |
| Ankle spacing width (left) | rs2373078 | 2 | 218392555 | T | C | 1.07E-01 | -3.03E-02 | 5.00E-03 | 1.40E-09 | 146226 | 0.025% | 36.71 |
| Ankle spacing width (left) | rs12714415 | 2 | 651430 | C | T | 1.63E-01 | -4.10E-02 | 4.23E-03 | 3.20E-22 | 146226 | 0.064% | 93.95 |
| Ankle spacing width (left) | rs12468920 | 2 | 66197123 | G | A | 3.06E-01 | -2.20E-02 | 3.36E-03 | 5.30E-11 | 146226 | 0.029% | 43.05 |
| Ankle spacing width (left) | rs11677976 | 2 | 112468218 | C | T | 4.09E-01 | -1.92E-02 | 3.13E-03 | 8.50E-10 | 146226 | 0.026% | 37.65 |
| Ankle spacing width (left) | rs56026429 | 2 | 230774106 | C | A | 3.68E-01 | 1.76E-02 | 3.22E-03 | 4.80E-08 | 146226 | 0.020% | 29.81 |
| Ankle spacing width (left) | rs77165542 | 2 | 430975 | T | C | 3.53E-02 | -6.01E-02 | 8.44E-03 | 1.10E-12 | 146226 | 0.035% | 50.62 |
| Ankle spacing width (left) | rs12713404 | 2 | 60006705 | T | G | 6.14E-01 | 3.01E-02 | 3.20E-03 | 4.10E-21 | 146226 | 0.061% | 88.93 |
| Ankle spacing width (left) | rs7568849 | 2 | 72320853 | C | T | 3.20E-01 | -2.28E-02 | 3.31E-03 | 5.80E-12 | 146226 | 0.032% | 47.41 |
| Ankle spacing width (left) | rs4075019 | 2 | 239949666 | C | A | 2.11E-01 | -2.30E-02 | 3.77E-03 | 1.00E-09 | 146226 | 0.025% | 37.27 |
| Ankle spacing width (left) | rs4832605 | 2 | 18722424 | C | T | 5.79E-01 | -3.64E-02 | 3.12E-03 | 2.30E-31 | 146226 | 0.093% | 135.75 |
| Ankle spacing width (left) | rs11895224 | 2 | 226922858 | C | T | 2.26E-01 | 2.93E-02 | 3.67E-03 | 1.60E-15 | 146226 | 0.043% | 63.48 |
| Ankle spacing width (left) | rs2596827 | 3 | 12546172 | G | T | 3.97E-01 | -1.77E-02 | 3.15E-03 | 2.00E-08 | 146226 | 0.022% | 31.51 |
| Ankle spacing width (left) | rs35772758 | 3 | 107297351 | C | T | 1.77E-01 | 2.31E-02 | 4.05E-03 | 1.20E-08 | 146226 | 0.022% | 32.56 |
| Ankle spacing width (left) | rs6773963 | 3 | 11511975 | A | G | 6.18E-01 | -2.28E-02 | 3.19E-03 | 8.70E-13 | 146226 | 0.035% | 51.13 |
| Ankle spacing width (left) | rs74681848 | 3 | 24493471 | T | C | 1.01E-01 | 2.94E-02 | 5.26E-03 | 2.20E-08 | 146226 | 0.021% | 31.30 |
| Ankle spacing width (left) | rs57863333 | 3 | 37263719 | A | G | 4.22E-01 | -1.73E-02 | 3.13E-03 | 3.40E-08 | 146226 | 0.021% | 30.44 |
| Ankle spacing width (left) | rs724016 | 3 | 141105570 | G | A | 4.46E-01 | 4.19E-02 | 3.10E-03 | 1.20E-41 | 146226 | 0.125% | 182.77 |
| Ankle spacing width (left) | rs57158761 | 3 | 185371172 | G | A | 4.39E-01 | -3.01E-02 | 3.12E-03 | 3.90E-22 | 146226 | 0.064% | 93.59 |
| Ankle spacing width (left) | rs9863778 | 3 | 55375951 | T | C | 7.33E-02 | -3.34E-02 | 5.85E-03 | 1.20E-08 | 146226 | 0.022% | 32.57 |
| Ankle spacing width (left) | rs832190 | 3 | 63842629 | T | C | 6.36E-01 | 1.92E-02 | 3.21E-03 | 2.30E-09 | 146226 | 0.024% | 35.71 |
| Ankle spacing width (left) | rs17275273 | 3 | 64944311 | C | T | 1.28E-01 | 7.14E-02 | 4.65E-03 | 3.80E-53 | 146226 | 0.161% | 235.50 |
| Ankle spacing width (left) | rs1040319 | 3 | 88098062 | G | T | 8.36E-01 | 2.40E-02 | 4.16E-03 | 8.30E-09 | 146226 | 0.023% | 33.21 |
| Ankle spacing width (left) | rs2270894 | 3 | 9975386 | G | C | 2.04E-01 | -3.23E-02 | 3.97E-03 | 3.80E-16 | 146226 | 0.045% | 66.32 |
| Ankle spacing width (left) | rs74720080 | 3 | 184000954 | G | A | 3.99E-02 | -4.45E-02 | 8.03E-03 | 2.90E-08 | 146226 | 0.021% | 30.75 |
| Ankle spacing width (left) | rs17082654 | 4 | 54243141 | G | A | 1.49E-01 | -2.42E-02 | 4.34E-03 | 2.40E-08 | 146226 | 0.021% | 31.10 |
| Ankle spacing width (left) | rs7671110 | 4 | 17874089 | T | C | 1.58E-01 | -3.36E-02 | 4.21E-03 | 1.70E-15 | 146226 | 0.043% | 63.38 |
| Ankle spacing width (left) | rs736436 | 4 | 1794188 | C | T | 1.98E-01 | -2.14E-02 | 3.91E-03 | 4.50E-08 | 146226 | 0.020% | 29.93 |
| Ankle spacing width (left) | rs13103161 | 4 | 106216459 | A | T | 3.87E-01 | -2.22E-02 | 3.17E-03 | 2.50E-12 | 146226 | 0.034% | 49.03 |
| Ankle spacing width (left) | rs72682784 | 4 | 124265193 | T | C | 3.93E-02 | -6.29E-02 | 7.96E-03 | 2.60E-15 | 146226 | 0.043% | 62.56 |
| Ankle spacing width (left) | rs2353939 | 4 | 145729724 | G | A | 4.36E-01 | 2.36E-02 | 3.12E-03 | 3.50E-14 | 146226 | 0.039% | 57.46 |
| Ankle spacing width (left) | rs1396193 | 4 | 175178914 | C | T | 1.82E-01 | -2.70E-02 | 4.01E-03 | 1.60E-11 | 146226 | 0.031% | 45.43 |
| Ankle spacing width (left) | rs61730641 | 4 | 87730980 | T | C | 1.66E-02 | -7.34E-02 | 1.21E-02 | 1.30E-09 | 146226 | 0.025% | 36.82 |
| Ankle spacing width (left) | rs17378658 | 4 | 95390157 | A | G | 2.10E-01 | 2.34E-02 | 3.80E-03 | 7.60E-10 | 146226 | 0.026% | 37.87 |
| Ankle spacing width (left) | rs34848742 | 4 | 123828042 | G | T | 7.88E-01 | -3.44E-02 | 3.77E-03 | 7.20E-20 | 146226 | 0.057% | 83.26 |
| Ankle spacing width (left) | rs6451624 | 5 | 42519262 | C | T | 4.65E-01 | -2.22E-02 | 3.08E-03 | 5.30E-13 | 146226 | 0.036% | 52.08 |
| Ankle spacing width (left) | rs6882235 | 5 | 50260139 | C | T | 4.14E-01 | 2.99E-02 | 3.15E-03 | 2.50E-21 | 146226 | 0.061% | 89.87 |
| Ankle spacing width (left) | rs7721882 | 5 | 131418948 | G | A | 4.34E-01 | 2.29E-02 | 3.12E-03 | 1.90E-13 | 146226 | 0.037% | 54.09 |
| Ankle spacing width (left) | rs3822742 | 5 | 139059017 | A | C | 3.72E-01 | 2.58E-02 | 3.20E-03 | 6.60E-16 | 146226 | 0.045% | 65.24 |
| Ankle spacing width (left) | rs13189570 | 5 | 158182309 | T | C | 2.41E-01 | -2.78E-02 | 3.62E-03 | 1.50E-14 | 146226 | 0.040% | 59.04 |
| Ankle spacing width (left) | rs1501709 | 5 | 39603834 | T | G | 2.44E-01 | -2.06E-02 | 3.59E-03 | 9.40E-09 | 146226 | 0.023% | 32.97 |
| Ankle spacing width (left) | rs78837755 | 5 | 51120392 | T | C | 7.29E-02 | 3.71E-02 | 5.95E-03 | 4.60E-10 | 146226 | 0.027% | 38.82 |
| Ankle spacing width (left) | rs4364342 | 5 | 51205024 | C | T | 8.49E-01 | 3.26E-02 | 4.34E-03 | 5.50E-14 | 146226 | 0.039% | 56.54 |
| Ankle spacing width (left) | rs11746090 | 5 | 77339846 | A | G | 3.07E-01 | 1.93E-02 | 3.35E-03 | 9.30E-09 | 146226 | 0.023% | 32.99 |
| Ankle spacing width (left) | rs56197583 | 5 | 173336792 | A | G | 3.02E-01 | -3.36E-02 | 3.36E-03 | 1.60E-23 | 146226 | 0.068% | 99.96 |
| Ankle spacing width (left) | rs34361 | 5 | 74971846 | G | A | 6.53E-01 | 1.88E-02 | 3.25E-03 | 7.10E-09 | 146226 | 0.023% | 33.51 |
| Ankle spacing width (left) | rs479632 | 5 | 134364518 | G | C | 2.50E-01 | -5.11E-02 | 3.56E-03 | 1.10E-46 | 146226 | 0.141% | 205.87 |
| Ankle spacing width (left) | rs79623207 | 6 | 34383659 | C | T | 1.93E-02 | 9.17E-02 | 1.12E-02 | 2.60E-16 | 146226 | 0.046% | 67.07 |
| Ankle spacing width (left) | rs1017741 | 6 | 81536262 | C | T | 4.78E-01 | -1.79E-02 | 3.09E-03 | 7.10E-09 | 146226 | 0.023% | 33.51 |
| Ankle spacing width (left) | rs2430189 | 6 | 139825880 | C | T | 5.00E-01 | -1.84E-02 | 3.08E-03 | 2.10E-09 | 146226 | 0.025% | 35.84 |
| Ankle spacing width (left) | rs3129962 | 6 | 32379383 | A | G | 1.27E-01 | 3.10E-02 | 4.62E-03 | 1.90E-11 | 146226 | 0.031% | 45.09 |
| Ankle spacing width (left) | rs6569648 | 6 | 130349119 | T | C | 7.61E-01 | -4.27E-02 | 3.61E-03 | 2.10E-32 | 146226 | 0.096% | 140.47 |
| Ankle spacing width (left) | rs544241 | 6 | 19973680 | T | C | 2.67E-01 | -2.40E-02 | 3.56E-03 | 1.70E-11 | 146226 | 0.031% | 45.23 |
| Ankle spacing width (left) | rs9379832 | 6 | 26186200 | G | A | 2.55E-01 | -2.27E-02 | 3.56E-03 | 1.80E-10 | 146226 | 0.028% | 40.64 |
| Ankle spacing width (left) | rs2178899 | 6 | 31606756 | T | A | 1.29E-01 | -3.91E-02 | 4.58E-03 | 1.30E-17 | 146226 | 0.050% | 72.92 |
| Ankle spacing width (left) | rs16887114 | 6 | 55636651 | T | A | 6.81E-02 | -6.91E-02 | 6.12E-03 | 1.50E-29 | 146226 | 0.087% | 127.40 |
| Ankle spacing width (left) | rs1361148 | 6 | 133547899 | G | T | 5.56E-01 | 2.80E-02 | 3.10E-03 | 1.90E-19 | 146226 | 0.056% | 81.31 |
| Ankle spacing width (left) | rs2296131 | 6 | 1872339 | C | T | 6.21E-01 | -1.75E-02 | 3.19E-03 | 3.70E-08 | 146226 | 0.021% | 30.28 |
| Ankle spacing width (left) | rs9267922 | 6 | 32206465 | A | G | 2.25E-01 | 2.09E-02 | 3.79E-03 | 3.60E-08 | 146226 | 0.021% | 30.37 |
| Ankle spacing width (left) | rs150445982 | 6 | 55676621 | T | C | 2.27E-02 | -8.09E-02 | 1.08E-02 | 8.50E-14 | 146226 | 0.038% | 55.69 |
| Ankle spacing width (left) | rs12189907 | 6 | 85369497 | C | T | 4.28E-01 | -2.81E-02 | 3.11E-03 | 1.70E-19 | 146226 | 0.056% | 81.53 |
| Ankle spacing width (left) | rs4946935 | 6 | 109000742 | G | A | 7.14E-01 | 2.23E-02 | 3.44E-03 | 8.10E-11 | 146226 | 0.029% | 42.23 |
| Ankle spacing width (left) | rs112202910 | 6 | 1388229 | C | G | 2.58E-01 | 2.59E-02 | 3.51E-03 | 1.50E-13 | 146226 | 0.037% | 54.57 |
| Ankle spacing width (left) | rs114056237 | 6 | 41877671 | A | G | 1.24E-02 | -7.85E-02 | 1.39E-02 | 1.60E-08 | 146226 | 0.022% | 31.97 |
| Ankle spacing width (left) | rs1502201 | 6 | 55265497 | A | G | 7.45E-01 | 3.26E-02 | 3.53E-03 | 2.10E-20 | 146226 | 0.059% | 85.66 |
| Ankle spacing width (left) | rs4709746 | 6 | 164133001 | T | C | 1.35E-01 | 3.37E-02 | 4.55E-03 | 1.10E-13 | 146226 | 0.038% | 55.11 |
| Ankle spacing width (left) | rs2215169 | 7 | 15956470 | C | T | 6.64E-01 | 2.17E-02 | 3.26E-03 | 2.90E-11 | 146226 | 0.030% | 44.25 |
| Ankle spacing width (left) | rs7782292 | 7 | 25864782 | C | A | 2.46E-01 | 3.59E-02 | 3.59E-03 | 1.50E-23 | 146226 | 0.068% | 100.01 |
| Ankle spacing width (left) | rs810366 | 7 | 74140925 | C | G | 2.07E-01 | 2.59E-02 | 3.82E-03 | 1.10E-11 | 146226 | 0.031% | 46.06 |
| Ankle spacing width (left) | rs6946415 | 7 | 150684548 | G | A | 6.28E-01 | 1.81E-02 | 3.19E-03 | 1.30E-08 | 146226 | 0.022% | 32.30 |
| Ankle spacing width (left) | rs2080246 | 7 | 27569704 | A | G | 6.92E-01 | -5.97E-02 | 3.35E-03 | 3.60E-71 | 146226 | 0.217% | 318.15 |
| Ankle spacing width (left) | rs17172722 | 7 | 46620312 | T | C | 4.20E-01 | 1.97E-02 | 3.12E-03 | 2.90E-10 | 146226 | 0.027% | 39.76 |
| Ankle spacing width (left) | rs10263705 | 7 | 130027633 | G | A | 3.48E-01 | 1.85E-02 | 3.24E-03 | 1.10E-08 | 146226 | 0.022% | 32.65 |
| Ankle spacing width (left) | rs6954290 | 7 | 92283098 | T | G | 3.26E-01 | 4.17E-02 | 3.29E-03 | 9.90E-37 | 146226 | 0.109% | 160.26 |
| Ankle spacing width (left) | rs62621812 | 7 | 127015083 | A | G | 2.05E-02 | 1.37E-01 | 1.12E-02 | 3.00E-34 | 146226 | 0.102% | 148.88 |
| Ankle spacing width (left) | rs2896395 | 7 | 127511705 | C | T | 7.08E-01 | 1.95E-02 | 3.40E-03 | 9.60E-09 | 146226 | 0.023% | 32.92 |
| Ankle spacing width (left) | rs3847072 | 7 | 105091519 | A | G | 2.43E-01 | -2.17E-02 | 3.71E-03 | 5.20E-09 | 146226 | 0.023% | 34.09 |
| Ankle spacing width (left) | rs2533882 | 7 | 2846894 | T | G | 2.97E-01 | -2.15E-02 | 3.37E-03 | 1.70E-10 | 146226 | 0.028% | 40.74 |
| Ankle spacing width (left) | rs9770544 | 7 | 27232126 | G | C | 8.21E-01 | 5.11E-02 | 4.06E-03 | 2.60E-36 | 146226 | 0.108% | 158.33 |
| Ankle spacing width (left) | rs2598109 | 7 | 37971913 | T | C | 8.40E-01 | 2.45E-02 | 4.20E-03 | 5.50E-09 | 146226 | 0.023% | 34.00 |
| Ankle spacing width (left) | rs2942202 | 8 | 23418444 | C | A | 4.95E-01 | -2.13E-02 | 3.10E-03 | 6.20E-12 | 146226 | 0.032% | 47.25 |
| Ankle spacing width (left) | rs12541381 | 8 | 135649848 | A | G | 2.57E-01 | -2.82E-02 | 3.53E-03 | 1.20E-15 | 146226 | 0.044% | 64.01 |
| Ankle spacing width (left) | rs72639068 | 8 | 49487358 | C | T | 9.79E-02 | 3.41E-02 | 5.21E-03 | 5.70E-11 | 146226 | 0.029% | 42.93 |
| Ankle spacing width (left) | rs10112467 | 8 | 72460614 | A | G | 7.36E-02 | -4.46E-02 | 5.93E-03 | 5.80E-14 | 146226 | 0.039% | 56.43 |
| Ankle spacing width (left) | rs28420312 | 8 | 74560577 | A | G | 2.07E-01 | 2.53E-02 | 3.80E-03 | 3.10E-11 | 146226 | 0.030% | 44.12 |
| Ankle spacing width (left) | rs13271228 | 8 | 116597409 | G | T | 5.68E-01 | -2.08E-02 | 3.12E-03 | 2.50E-11 | 146226 | 0.030% | 44.57 |
| Ankle spacing width (left) | rs7815245 | 8 | 128383597 | T | C | 4.20E-01 | -2.03E-02 | 3.13E-03 | 7.60E-11 | 146226 | 0.029% | 42.34 |
| Ankle spacing width (left) | rs13267329 | 8 | 135735110 | A | G | 1.54E-01 | 2.79E-02 | 4.29E-03 | 8.10E-11 | 146226 | 0.029% | 42.24 |
| Ankle spacing width (left) | rs11136336 | 8 | 145007187 | A | G | 3.97E-01 | -2.18E-02 | 3.17E-03 | 6.20E-12 | 146226 | 0.032% | 47.28 |
| Ankle spacing width (left) | rs957240 | 8 | 10063508 | A | T | 2.01E-01 | 2.21E-02 | 3.88E-03 | 1.30E-08 | 146226 | 0.022% | 32.29 |
| Ankle spacing width (left) | rs72656010 | 8 | 57122215 | C | T | 1.33E-01 | -3.69E-02 | 4.57E-03 | 7.40E-16 | 146226 | 0.044% | 65.03 |
| Ankle spacing width (left) | rs9721192 | 8 | 57166109 | G | A | 2.28E-01 | 2.06E-02 | 3.69E-03 | 2.20E-08 | 146226 | 0.021% | 31.28 |
| Ankle spacing width (left) | rs7819550 | 8 | 74206582 | A | G | 1.94E-01 | 2.17E-02 | 3.91E-03 | 2.80E-08 | 146226 | 0.021% | 30.87 |
| Ankle spacing width (left) | rs969649 | 8 | 96666179 | T | C | 7.73E-01 | 3.57E-02 | 3.70E-03 | 5.50E-22 | 146226 | 0.063% | 92.91 |
| Ankle spacing width (left) | rs913277 | 9 | 96910136 | T | C | 7.03E-01 | -2.01E-02 | 3.40E-03 | 3.70E-09 | 146226 | 0.024% | 34.80 |
| Ankle spacing width (left) | rs28504650 | 9 | 98257842 | T | C | 3.54E-01 | 2.22E-02 | 3.24E-03 | 7.10E-12 | 146226 | 0.032% | 47.01 |
| Ankle spacing width (left) | rs2772033 | 9 | 133757080 | A | G | 1.11E-01 | -2.75E-02 | 4.94E-03 | 2.70E-08 | 146226 | 0.021% | 30.95 |
| Ankle spacing width (left) | rs4879811 | 9 | 34639474 | T | C | 5.16E-01 | -1.77E-02 | 3.10E-03 | 1.20E-08 | 146226 | 0.022% | 32.41 |
| Ankle spacing width (left) | rs10756486 | 9 | 13591185 | T | C | 7.63E-01 | -2.29E-02 | 3.64E-03 | 3.00E-10 | 146226 | 0.027% | 39.67 |
| Ankle spacing width (left) | rs12344772 | 9 | 118490315 | T | G | 8.40E-02 | -4.08E-02 | 5.60E-03 | 3.30E-13 | 146226 | 0.036% | 53.04 |
| Ankle spacing width (left) | rs35307904 | 9 | 78511889 | A | G | 1.23E-01 | -2.73E-02 | 4.72E-03 | 7.50E-09 | 146226 | 0.023% | 33.40 |
| Ankle spacing width (left) | rs3761849 | 9 | 123690957 | T | C | 6.20E-01 | 1.97E-02 | 3.20E-03 | 7.00E-10 | 146226 | 0.026% | 38.02 |
| Ankle spacing width (left) | rs72761015 | 9 | 139620333 | T | C | 1.25E-01 | -2.73E-02 | 4.68E-03 | 5.30E-09 | 146226 | 0.023% | 34.07 |
| Ankle spacing width (left) | rs2808277 | 10 | 27946997 | A | T | 4.61E-01 | 1.92E-02 | 3.10E-03 | 5.60E-10 | 146226 | 0.026% | 38.46 |
| Ankle spacing width (left) | rs10786679 | 10 | 104315667 | A | G | 2.81E-01 | -2.08E-02 | 3.44E-03 | 1.60E-09 | 146226 | 0.025% | 36.37 |
| Ankle spacing width (left) | rs7918664 | 10 | 28819640 | C | G | 2.79E-01 | -1.97E-02 | 3.45E-03 | 1.00E-08 | 146226 | 0.022% | 32.75 |
| Ankle spacing width (left) | rs603424 | 10 | 102075479 | A | G | 1.75E-01 | 7.63E-02 | 4.05E-03 | 2.50E-79 | 146226 | 0.243% | 355.61 |
| Ankle spacing width (left) | rs10995311 | 10 | 64564934 | G | C | 4.44E-01 | -1.76E-02 | 3.12E-03 | 1.80E-08 | 146226 | 0.022% | 31.74 |
| Ankle spacing width (left) | rs2244092 | 10 | 89777788 | G | A | 5.75E-01 | 1.90E-02 | 3.14E-03 | 1.50E-09 | 146226 | 0.025% | 36.54 |
| Ankle spacing width (left) | rs41306858 | 10 | 124157385 | C | T | 7.04E-02 | -3.34E-02 | 6.03E-03 | 3.10E-08 | 146226 | 0.021% | 30.65 |
| Ankle spacing width (left) | rs11007321 | 10 | 29232856 | C | T | 5.17E-01 | 2.62E-02 | 3.10E-03 | 3.50E-17 | 146226 | 0.049% | 71.04 |
| Ankle spacing width (left) | rs567602 | 11 | 1880476 | C | T | 6.30E-01 | -2.36E-02 | 3.20E-03 | 1.50E-13 | 146226 | 0.037% | 54.51 |
| Ankle spacing width (left) | rs11234641 | 11 | 86120586 | T | C | 2.32E-01 | -2.16E-02 | 3.65E-03 | 3.50E-09 | 146226 | 0.024% | 34.86 |
| Ankle spacing width (left) | rs11030104 | 11 | 27684517 | G | A | 2.03E-01 | -2.90E-02 | 3.84E-03 | 4.10E-14 | 146226 | 0.039% | 57.13 |
| Ankle spacing width (left) | rs11607174 | 11 | 10306028 | T | C | 5.51E-01 | -1.88E-02 | 3.11E-03 | 1.40E-09 | 146226 | 0.025% | 36.70 |
| Ankle spacing width (left) | rs72894429 | 11 | 43825923 | T | C | 5.29E-02 | 3.93E-02 | 6.91E-03 | 1.30E-08 | 146226 | 0.022% | 32.33 |
| Ankle spacing width (left) | rs603326 | 11 | 65556721 | G | C | 3.32E-01 | 2.42E-02 | 3.28E-03 | 1.60E-13 | 146226 | 0.037% | 54.40 |
| Ankle spacing width (left) | rs57307148 | 12 | 1033183 | A | C | 2.12E-01 | 2.09E-02 | 3.79E-03 | 3.50E-08 | 146226 | 0.021% | 30.43 |
| Ankle spacing width (left) | rs35756741 | 12 | 12868701 | T | C | 9.08E-02 | -3.80E-02 | 5.41E-03 | 2.20E-12 | 146226 | 0.034% | 49.32 |
| Ankle spacing width (left) | rs770082 | 12 | 89776485 | A | G | 4.35E-01 | 1.96E-02 | 3.13E-03 | 3.60E-10 | 146226 | 0.027% | 39.34 |
| Ankle spacing width (left) | rs17824882 | 12 | 65863825 | G | A | 6.18E-02 | 4.02E-02 | 6.47E-03 | 5.00E-10 | 146226 | 0.026% | 38.67 |
| Ankle spacing width (left) | rs76895963 | 12 | 4384844 | G | T | 2.07E-02 | 1.38E-01 | 1.19E-02 | 7.80E-31 | 146226 | 0.091% | 133.30 |
| Ankle spacing width (left) | rs822688 | 12 | 53493387 | T | C | 1.25E-01 | 2.74E-02 | 4.77E-03 | 8.80E-09 | 146226 | 0.023% | 33.08 |
| Ankle spacing width (left) | rs11175826 | 12 | 65977830 | T | C | 1.80E-02 | -1.32E-01 | 1.17E-02 | 1.50E-29 | 146226 | 0.087% | 127.43 |
| Ankle spacing width (left) | rs34426931 | 12 | 77615252 | A | C | 4.00E-01 | 2.01E-02 | 3.15E-03 | 1.80E-10 | 146226 | 0.028% | 40.72 |
| Ankle spacing width (left) | rs11048454 | 12 | 26460045 | A | C | 2.35E-01 | -2.36E-02 | 3.64E-03 | 8.80E-11 | 146226 | 0.029% | 42.07 |
| Ankle spacing width (left) | rs7487625 | 12 | 66319996 | C | A | 2.28E-01 | -5.65E-02 | 3.69E-03 | 7.00E-53 | 146226 | 0.160% | 234.27 |
| Ankle spacing width (left) | rs11111141 | 12 | 102429501 | C | A | 1.78E-01 | 2.25E-02 | 4.04E-03 | 2.60E-08 | 146226 | 0.021% | 30.96 |
| Ankle spacing width (left) | rs157165 | 13 | 51082944 | T | C | 2.27E-01 | -4.77E-02 | 3.70E-03 | 4.90E-38 | 146226 | 0.114% | 166.25 |
| Ankle spacing width (left) | rs35052580 | 13 | 30976240 | T | G | 5.16E-02 | 4.26E-02 | 7.07E-03 | 1.70E-09 | 146226 | 0.025% | 36.27 |
| Ankle spacing width (left) | rs837302 | 13 | 101191651 | G | C | 5.18E-01 | -2.31E-02 | 3.09E-03 | 7.70E-14 | 146226 | 0.038% | 55.87 |
| Ankle spacing width (left) | rs35551290 | 13 | 50466633 | T | C | 1.00E-01 | 3.23E-02 | 5.25E-03 | 7.80E-10 | 146226 | 0.026% | 37.81 |
| Ankle spacing width (left) | rs7986552 | 13 | 91919443 | G | A | 4.17E-01 | 1.84E-02 | 3.14E-03 | 5.00E-09 | 146226 | 0.023% | 34.19 |
| Ankle spacing width (left) | rs71413981 | 14 | 23774916 | A | G | 1.62E-01 | 2.70E-02 | 4.20E-03 | 1.30E-10 | 146226 | 0.028% | 41.24 |
| Ankle spacing width (left) | rs1870043 | 14 | 98405049 | A | G | 5.67E-01 | 2.78E-02 | 3.13E-03 | 8.00E-19 | 146226 | 0.054% | 78.49 |
| Ankle spacing width (left) | rs6572916 | 14 | 54103013 | T | G | 3.36E-01 | -2.52E-02 | 3.30E-03 | 2.40E-14 | 146226 | 0.040% | 58.14 |
| Ankle spacing width (left) | rs28929474 | 14 | 94844947 | T | C | 2.00E-02 | 8.79E-02 | 1.10E-02 | 1.50E-15 | 146226 | 0.044% | 63.64 |
| Ankle spacing width (left) | rs10133006 | 14 | 21749473 | G | A | 2.99E-01 | 1.89E-02 | 3.39E-03 | 2.70E-08 | 146226 | 0.021% | 30.93 |
| Ankle spacing width (left) | rs61991652 | 14 | 77518204 | T | C | 2.47E-01 | 2.05E-02 | 3.60E-03 | 1.30E-08 | 146226 | 0.022% | 32.29 |
| Ankle spacing width (left) | rs8014071 | 14 | 54431500 | G | A | 3.86E-01 | -2.67E-02 | 3.19E-03 | 5.90E-17 | 146226 | 0.048% | 70.01 |
| Ankle spacing width (left) | rs1954005 | 14 | 59611705 | G | A | 8.32E-01 | -2.46E-02 | 4.14E-03 | 2.70E-09 | 146226 | 0.024% | 35.38 |
| Ankle spacing width (left) | rs11844656 | 14 | 91459332 | C | G | 5.28E-01 | 1.88E-02 | 3.09E-03 | 1.20E-09 | 146226 | 0.025% | 37.03 |
| Ankle spacing width (left) | rs2439387 | 15 | 67024951 | C | T | 7.16E-01 | -2.83E-02 | 3.43E-03 | 1.60E-16 | 146226 | 0.047% | 68.06 |
| Ankle spacing width (left) | rs12908437 | 15 | 99287375 | C | T | 6.32E-01 | -2.34E-02 | 3.22E-03 | 3.70E-13 | 146226 | 0.036% | 52.81 |
| Ankle spacing width (left) | rs80182532 | 15 | 68269444 | C | T | 4.31E-02 | -4.53E-02 | 7.69E-03 | 3.80E-09 | 146226 | 0.024% | 34.72 |
| Ankle spacing width (left) | rs56178382 | 15 | 75898996 | T | A | 2.50E-01 | -2.15E-02 | 3.57E-03 | 1.60E-09 | 146226 | 0.025% | 36.45 |
| Ankle spacing width (left) | rs11247362 | 15 | 99210831 | T | C | 8.96E-01 | 2.91E-02 | 5.08E-03 | 1.00E-08 | 146226 | 0.022% | 32.75 |
| Ankle spacing width (left) | rs9941221 | 16 | 54258031 | C | T | 8.39E-01 | -2.95E-02 | 4.21E-03 | 2.70E-12 | 146226 | 0.033% | 48.88 |
| Ankle spacing width (left) | rs8059189 | 16 | 86417349 | A | G | 4.03E-01 | -1.81E-02 | 3.23E-03 | 2.30E-08 | 146226 | 0.021% | 31.26 |
| Ankle spacing width (left) | rs205191 | 16 | 25658104 | T | C | 5.35E-01 | 1.75E-02 | 3.11E-03 | 1.90E-08 | 146226 | 0.022% | 31.64 |
| Ankle spacing width (left) | rs6498089 | 16 | 28629300 | T | C | 6.24E-01 | -1.99E-02 | 3.19E-03 | 4.00E-10 | 146226 | 0.027% | 39.12 |
| Ankle spacing width (left) | rs12325400 | 16 | 30023786 | G | C | 3.98E-01 | 2.27E-02 | 3.15E-03 | 6.70E-13 | 146226 | 0.035% | 51.64 |
| Ankle spacing width (left) | rs12443634 | 16 | 81524274 | C | A | 7.15E-01 | -2.03E-02 | 3.45E-03 | 3.60E-09 | 146226 | 0.024% | 34.83 |
| Ankle spacing width (left) | rs9923544 | 16 | 53801985 | T | C | 4.25E-01 | 3.26E-02 | 3.12E-03 | 1.50E-25 | 146226 | 0.075% | 109.18 |
| Ankle spacing width (left) | rs11867227 | 17 | 29250911 | A | G | 2.67E-01 | -2.32E-02 | 3.49E-03 | 3.20E-11 | 146226 | 0.030% | 44.05 |
| Ankle spacing width (left) | rs8077036 | 17 | 67962340 | G | C | 3.43E-01 | -2.31E-02 | 3.39E-03 | 9.80E-12 | 146226 | 0.032% | 46.36 |
| Ankle spacing width (left) | rs147682226 | 17 | 79493146 | A | G | 2.50E-02 | 5.65E-02 | 1.03E-02 | 4.50E-08 | 146226 | 0.020% | 29.94 |
| Ankle spacing width (left) | rs11654636 | 17 | 2008904 | A | G | 3.77E-01 | -2.25E-02 | 3.19E-03 | 1.90E-12 | 146226 | 0.034% | 49.54 |
| Ankle spacing width (left) | rs2005172 | 17 | 61996255 | C | A | 6.40E-01 | 2.20E-02 | 3.26E-03 | 1.50E-11 | 146226 | 0.031% | 45.56 |
| Ankle spacing width (left) | rs523190 | 17 | 14403421 | A | G | 5.03E-01 | 2.26E-02 | 3.10E-03 | 2.80E-13 | 146226 | 0.036% | 53.31 |
| Ankle spacing width (left) | rs2159035 | 17 | 69121783 | A | G | 5.71E-01 | -2.24E-02 | 3.12E-03 | 6.70E-13 | 146226 | 0.035% | 51.64 |
| Ankle spacing width (left) | rs34822124 | 17 | 46648679 | T | G | 1.93E-01 | -2.82E-02 | 3.93E-03 | 7.60E-13 | 146226 | 0.035% | 51.38 |
| Ankle spacing width (left) | rs9910180 | 17 | 54247035 | G | A | 1.28E-01 | -2.91E-02 | 4.64E-03 | 3.30E-10 | 146226 | 0.027% | 39.49 |
| Ankle spacing width (left) | rs35572189 | 17 | 79419025 | A | G | 3.62E-01 | 3.42E-02 | 3.23E-03 | 3.30E-26 | 146226 | 0.077% | 112.15 |
| Ankle spacing width (left) | rs9675924 | 18 | 20730712 | G | A | 7.44E-01 | 2.46E-02 | 3.56E-03 | 5.30E-12 | 146226 | 0.033% | 47.59 |
| Ankle spacing width (left) | rs7229520 | 18 | 46516468 | A | G | 6.62E-01 | -3.54E-02 | 3.27E-03 | 3.20E-27 | 146226 | 0.080% | 116.79 |
| Ankle spacing width (left) | rs17782313 | 18 | 57851097 | C | T | 2.35E-01 | 3.70E-02 | 3.64E-03 | 2.50E-24 | 146226 | 0.071% | 103.55 |
| Ankle spacing width (left) | rs8104313 | 19 | 6115951 | G | A | 3.55E-01 | -2.07E-02 | 3.25E-03 | 2.00E-10 | 146226 | 0.028% | 40.46 |
| Ankle spacing width (left) | rs10401891 | 19 | 4962848 | T | C | 3.26E-01 | -3.20E-02 | 3.31E-03 | 4.00E-22 | 146226 | 0.064% | 93.54 |
| Ankle spacing width (left) | rs895819 | 19 | 13947292 | C | T | 3.35E-01 | 1.96E-02 | 3.30E-03 | 2.60E-09 | 146226 | 0.024% | 35.44 |
| Ankle spacing width (left) | rs561059 | 20 | 3011175 | G | A | 4.96E-01 | -1.74E-02 | 3.10E-03 | 1.80E-08 | 146226 | 0.022% | 31.74 |
| Ankle spacing width (left) | rs7270170 | 20 | 44526783 | A | G | 4.56E-01 | 1.75E-02 | 3.10E-03 | 1.60E-08 | 146226 | 0.022% | 31.95 |
| Ankle spacing width (left) | rs73622688 | 20 | 45523702 | A | C | 3.76E-01 | 2.03E-02 | 3.20E-03 | 2.20E-10 | 146226 | 0.028% | 40.29 |
| Ankle spacing width (left) | rs57668191 | 20 | 32289763 | G | A | 2.61E-01 | -2.90E-02 | 3.53E-03 | 2.40E-16 | 146226 | 0.046% | 67.27 |
| Ankle spacing width (left) | rs6512577 | 20 | 47865784 | T | C | 2.19E-01 | 2.05E-02 | 3.74E-03 | 4.00E-08 | 146226 | 0.021% | 30.15 |
| Ankle spacing width (left) | rs34031061 | 20 | 7593266 | C | G | 3.02E-01 | 2.26E-02 | 3.37E-03 | 2.00E-11 | 146226 | 0.031% | 45.00 |
| Ankle spacing width (left) | rs143384 | 20 | 34025756 | G | A | 4.07E-01 | 2.43E-02 | 3.14E-03 | 1.10E-14 | 146226 | 0.041% | 59.62 |
| Ankle spacing width (left) | rs2023682 | 22 | 30599570 | G | A | 2.99E-01 | 2.42E-02 | 3.39E-03 | 8.70E-13 | 146226 | 0.035% | 51.12 |
| Ankle spacing width (left) | rs62240962 | 22 | 42259524 | T | C | 8.39E-02 | -4.33E-02 | 5.58E-03 | 9.00E-15 | 146226 | 0.041% | 60.11 |
| Ankle spacing width (left) | rs5750927 | 22 | 40674593 | A | G | 4.00E-01 | 2.49E-02 | 3.16E-03 | 3.70E-15 | 146226 | 0.042% | 61.85 |
| Ankle spacing width (left) | rs1569414 | 22 | 45727565 | G | T | 2.59E-01 | 3.31E-02 | 3.52E-03 | 5.00E-21 | 146226 | 0.060% | 88.52 |
| Ankle spacing width | rs58265521 | 1 | 2943398 | A | C | 1.85E-01 | 2.58E-02 | 2.91E-03 | 7.60E-19 | 265753 | 0.030% | 78.59 |
| Ankle spacing width | rs12059628 | 1 | 44067473 | C | T | 4.05E-01 | -1.44E-02 | 2.29E-03 | 3.80E-10 | 265753 | 0.015% | 39.19 |
| Ankle spacing width | rs12408387 | 1 | 119528329 | A | C | 3.06E-01 | 2.50E-02 | 2.45E-03 | 1.50E-24 | 265753 | 0.039% | 104.61 |
| Ankle spacing width | rs11119840 | 1 | 212246168 | T | C | 4.92E-01 | 1.49E-02 | 2.25E-03 | 3.80E-11 | 265753 | 0.016% | 43.72 |
| Ankle spacing width | rs61819677 | 1 | 215388087 | C | T | 2.15E-01 | 2.40E-02 | 2.74E-03 | 1.90E-18 | 265753 | 0.029% | 76.74 |
| Ankle spacing width | rs3753639 | 1 | 154986091 | C | T | 2.43E-01 | 1.80E-02 | 2.64E-03 | 9.20E-12 | 265753 | 0.017% | 46.49 |
| Ankle spacing width | rs2666826 | 1 | 155658085 | C | T | 2.82E-01 | -1.81E-02 | 2.50E-03 | 5.10E-13 | 265753 | 0.020% | 52.17 |
| Ankle spacing width | rs560584 | 1 | 170090512 | T | C | 4.08E-01 | 1.26E-02 | 2.29E-03 | 3.70E-08 | 265753 | 0.011% | 30.28 |
| Ankle spacing width | rs2678204 | 1 | 201800511 | G | T | 3.41E-01 | 1.81E-02 | 2.38E-03 | 2.70E-14 | 265753 | 0.022% | 57.91 |
| Ankle spacing width | rs6700139 | 1 | 51337868 | C | T | 3.36E-01 | -2.32E-02 | 2.39E-03 | 2.50E-22 | 265753 | 0.036% | 94.48 |
| Ankle spacing width | rs12140153 | 1 | 62579891 | T | G | 9.44E-02 | -2.41E-02 | 3.95E-03 | 1.00E-09 | 265753 | 0.014% | 37.24 |
| Ankle spacing width | rs11162478 | 1 | 78839691 | T | C | 3.07E-01 | 1.81E-02 | 2.45E-03 | 1.60E-13 | 265753 | 0.020% | 54.40 |
| Ankle spacing width | rs993471 | 1 | 103385373 | A | G | 6.11E-01 | -2.44E-02 | 2.31E-03 | 4.00E-26 | 265753 | 0.042% | 111.78 |
| Ankle spacing width | rs140604451 | 1 | 110216436 | A | G | 2.51E-02 | 4.12E-02 | 7.25E-03 | 1.30E-08 | 265753 | 0.012% | 32.29 |
| Ankle spacing width | rs34276133 | 1 | 176472569 | A | C | 5.03E-02 | 3.91E-02 | 5.16E-03 | 3.70E-14 | 265753 | 0.022% | 57.30 |
| Ankle spacing width | rs1325596 | 1 | 176794066 | A | G | 5.47E-01 | 2.69E-02 | 2.27E-03 | 1.90E-32 | 265753 | 0.053% | 140.71 |
| Ankle spacing width | rs2791643 | 1 | 11207269 | T | C | 7.62E-01 | -2.58E-02 | 2.64E-03 | 2.10E-22 | 265753 | 0.036% | 94.83 |
| Ankle spacing width | rs2885697 | 1 | 41544279 | T | G | 6.64E-01 | -2.63E-02 | 2.38E-03 | 2.60E-28 | 265753 | 0.046% | 121.77 |
| Ankle spacing width | rs1969111 | 1 | 75263485 | T | A | 6.27E-01 | 3.09E-02 | 2.34E-03 | 1.10E-39 | 265753 | 0.065% | 173.83 |
| Ankle spacing width | rs1498405 | 1 | 77927948 | G | A | 2.72E-01 | 1.41E-02 | 2.55E-03 | 3.10E-08 | 265753 | 0.012% | 30.67 |
| Ankle spacing width | rs1973993 | 1 | 96943994 | C | T | 5.97E-01 | 1.50E-02 | 2.29E-03 | 5.40E-11 | 265753 | 0.016% | 43.04 |
| Ankle spacing width | rs11120327 | 1 | 214626397 | A | G | 3.97E-01 | -2.13E-02 | 2.30E-03 | 2.20E-20 | 265753 | 0.032% | 85.61 |
| Ankle spacing width | rs113064761 | 1 | 31549962 | A | G | 3.50E-01 | -1.48E-02 | 2.37E-03 | 4.00E-10 | 265753 | 0.015% | 39.12 |
| Ankle spacing width | rs652112 | 1 | 32345980 | C | T | 1.05E-01 | 2.51E-02 | 3.67E-03 | 8.20E-12 | 265753 | 0.018% | 46.72 |
| Ankle spacing width | rs11206186 | 1 | 54117985 | T | G | 5.54E-01 | 1.27E-02 | 2.27E-03 | 2.10E-08 | 265753 | 0.012% | 31.40 |
| Ankle spacing width | rs75699474 | 1 | 67396494 | T | C | 2.41E-02 | 4.23E-02 | 7.37E-03 | 9.00E-09 | 265753 | 0.012% | 33.05 |
| Ankle spacing width | rs3790609 | 1 | 113056990 | T | C | 1.80E-01 | -1.85E-02 | 2.94E-03 | 3.00E-10 | 265753 | 0.015% | 39.68 |
| Ankle spacing width | rs575908 | 1 | 177900098 | C | T | 2.98E-01 | 1.97E-02 | 2.46E-03 | 1.10E-15 | 265753 | 0.024% | 64.16 |
| Ankle spacing width | rs7527300 | 1 | 221477744 | T | C | 4.06E-01 | 1.91E-02 | 2.30E-03 | 1.30E-16 | 265753 | 0.026% | 68.52 |
| Ankle spacing width | rs13007086 | 2 | 630034 | T | A | 8.28E-01 | 3.67E-02 | 2.99E-03 | 1.10E-34 | 265753 | 0.057% | 150.97 |
| Ankle spacing width | rs10172544 | 2 | 85788270 | A | C | 4.09E-01 | 1.65E-02 | 2.29E-03 | 5.80E-13 | 265753 | 0.020% | 51.93 |
| Ankle spacing width | rs80185640 | 2 | 145641757 | G | A | 4.61E-02 | -5.88E-02 | 5.44E-03 | 2.80E-27 | 265753 | 0.044% | 117.05 |
| Ankle spacing width | rs139860835 | 2 | 145793696 | G | A | 1.46E-02 | 5.25E-02 | 9.38E-03 | 2.10E-08 | 265753 | 0.012% | 31.35 |
| Ankle spacing width | rs10932707 | 2 | 218114695 | C | T | 2.63E-01 | -1.68E-02 | 2.57E-03 | 6.30E-11 | 265753 | 0.016% | 42.72 |
| Ankle spacing width | rs57482375 | 2 | 227008320 | C | T | 2.24E-01 | 2.24E-02 | 2.72E-03 | 1.80E-16 | 265753 | 0.025% | 67.78 |
| Ankle spacing width | rs62191099 | 2 | 239857753 | A | G | 2.85E-01 | -1.37E-02 | 2.51E-03 | 4.60E-08 | 265753 | 0.011% | 29.87 |
| Ankle spacing width | rs11096542 | 2 | 18707873 | A | G | 5.93E-01 | -2.85E-02 | 2.30E-03 | 1.80E-35 | 265753 | 0.058% | 154.45 |
| Ankle spacing width | rs7590965 | 2 | 33454220 | C | A | 5.34E-01 | -1.53E-02 | 2.28E-03 | 1.70E-11 | 265753 | 0.017% | 45.31 |
| Ankle spacing width | rs11901863 | 2 | 100245075 | A | C | 5.18E-01 | -1.63E-02 | 2.26E-03 | 5.60E-13 | 265753 | 0.020% | 51.98 |
| Ankle spacing width | rs1451533 | 2 | 105466005 | A | G | 2.73E-01 | 1.52E-02 | 2.55E-03 | 2.70E-09 | 265753 | 0.013% | 35.39 |
| Ankle spacing width | rs7593730 | 2 | 161171454 | C | T | 7.77E-01 | 1.74E-02 | 2.71E-03 | 1.30E-10 | 265753 | 0.016% | 41.38 |
| Ankle spacing width | rs4673627 | 2 | 200848896 | A | G | 5.60E-01 | 1.64E-02 | 2.27E-03 | 4.80E-13 | 265753 | 0.020% | 52.28 |
| Ankle spacing width | rs77165542 | 2 | 430975 | T | C | 3.55E-02 | -5.47E-02 | 6.16E-03 | 6.90E-19 | 265753 | 0.030% | 78.80 |
| Ankle spacing width | rs10177811 | 2 | 42263580 | A | G | 1.93E-01 | -3.83E-02 | 2.87E-03 | 1.00E-40 | 265753 | 0.067% | 178.55 |
| Ankle spacing width | rs3791679 | 2 | 56096892 | G | A | 2.27E-01 | -1.65E-02 | 2.69E-03 | 8.40E-10 | 265753 | 0.014% | 37.67 |
| Ankle spacing width | rs2031050 | 2 | 66413916 | C | T | 1.42E-01 | 2.88E-02 | 3.27E-03 | 1.30E-18 | 265753 | 0.029% | 77.58 |
| Ankle spacing width | rs7607980 | 2 | 165551201 | C | T | 1.20E-01 | -2.03E-02 | 3.47E-03 | 5.10E-09 | 265753 | 0.013% | 34.14 |
| Ankle spacing width | rs4075018 | 2 | 239949681 | A | G | 2.06E-01 | -1.78E-02 | 2.78E-03 | 1.50E-10 | 265753 | 0.015% | 41.03 |
| Ankle spacing width | rs2888677 | 2 | 40638024 | A | G | 3.57E-01 | 1.75E-02 | 2.38E-03 | 1.70E-13 | 265753 | 0.020% | 54.36 |
| Ankle spacing width | rs1442873 | 2 | 60027032 | T | C | 5.90E-01 | 2.58E-02 | 2.31E-03 | 7.10E-29 | 265753 | 0.047% | 124.33 |
| Ankle spacing width | rs6739994 | 2 | 60770454 | T | C | 8.31E-01 | 2.07E-02 | 3.01E-03 | 6.40E-12 | 265753 | 0.018% | 47.21 |
| Ankle spacing width | rs12477050 | 2 | 72328144 | G | A | 3.07E-01 | -1.97E-02 | 2.45E-03 | 1.10E-15 | 265753 | 0.024% | 64.22 |
| Ankle spacing width | rs11691123 | 2 | 112463110 | C | A | 4.12E-01 | -1.79E-02 | 2.29E-03 | 5.10E-15 | 265753 | 0.023% | 61.20 |
| Ankle spacing width | rs113865045 | 2 | 176969703 | A | G | 6.29E-02 | 3.34E-02 | 4.66E-03 | 7.40E-13 | 265753 | 0.019% | 51.43 |
| Ankle spacing width | rs10498240 | 2 | 230734531 | A | C | 3.18E-01 | 1.59E-02 | 2.42E-03 | 5.30E-11 | 265753 | 0.016% | 43.05 |
| Ankle spacing width | rs2260116 | 2 | 147847165 | C | T | 6.16E-01 | -1.31E-02 | 2.33E-03 | 1.60E-08 | 265753 | 0.012% | 31.90 |
| Ankle spacing width | rs13424197 | 2 | 197379185 | G | C | 2.09E-01 | 2.15E-02 | 2.77E-03 | 9.10E-15 | 265753 | 0.023% | 60.08 |
| Ankle spacing width | rs6736742 | 2 | 218277149 | G | A | 7.12E-01 | 2.49E-02 | 2.49E-03 | 1.80E-23 | 265753 | 0.037% | 99.63 |
| Ankle spacing width | rs2325036 | 3 | 85819412 | C | A | 3.77E-01 | -1.51E-02 | 2.33E-03 | 8.20E-11 | 265753 | 0.016% | 42.21 |
| Ankle spacing width | rs9861443 | 3 | 88196211 | C | A | 7.15E-01 | 1.75E-02 | 2.51E-03 | 3.30E-12 | 265753 | 0.018% | 48.50 |
| Ankle spacing width | rs1454687 | 3 | 94038085 | G | C | 5.15E-01 | -1.35E-02 | 2.26E-03 | 2.20E-09 | 265753 | 0.013% | 35.80 |
| Ankle spacing width | rs62266925 | 3 | 129052586 | A | G | 1.01E-01 | 2.16E-02 | 3.75E-03 | 8.10E-09 | 265753 | 0.013% | 33.25 |
| Ankle spacing width | rs4683606 | 3 | 141110074 | G | A | 4.44E-01 | 4.36E-02 | 2.27E-03 | 3.40E-82 | 265753 | 0.139% | 368.78 |
| Ankle spacing width | rs77494444 | 3 | 185550500 | T | C | 5.28E-02 | -3.51E-02 | 5.03E-03 | 3.20E-12 | 265753 | 0.018% | 48.55 |
| Ankle spacing width | rs2270894 | 3 | 9975386 | G | C | 2.03E-01 | -3.43E-02 | 2.91E-03 | 4.20E-32 | 265753 | 0.052% | 139.08 |
| Ankle spacing width | rs7614498 | 3 | 52618941 | T | A | 4.10E-01 | -1.49E-02 | 2.29E-03 | 7.70E-11 | 265753 | 0.016% | 42.34 |
| Ankle spacing width | rs3732360 | 3 | 119536581 | T | C | 7.46E-01 | 1.69E-02 | 2.59E-03 | 7.10E-11 | 265753 | 0.016% | 42.49 |
| Ankle spacing width | rs876424 | 3 | 131637676 | G | T | 3.65E-01 | 1.68E-02 | 2.35E-03 | 8.30E-13 | 265753 | 0.019% | 51.20 |
| Ankle spacing width | rs7652177 | 3 | 171969077 | G | C | 5.05E-01 | 1.92E-02 | 2.26E-03 | 1.80E-17 | 265753 | 0.027% | 72.32 |
| Ankle spacing width | rs7631981 | 3 | 185273510 | A | G | 3.05E-01 | -3.62E-02 | 2.47E-03 | 1.10E-48 | 265753 | 0.081% | 215.04 |
| Ankle spacing width | rs7632937 | 3 | 14169079 | C | T | 3.79E-02 | -3.62E-02 | 5.90E-03 | 8.10E-10 | 265753 | 0.014% | 37.73 |
| Ankle spacing width | rs13074055 | 3 | 24476943 | T | C | 2.11E-01 | 2.03E-02 | 2.80E-03 | 3.40E-13 | 265753 | 0.020% | 52.95 |
| Ankle spacing width | rs4909017 | 3 | 32937596 | T | C | 5.57E-01 | 1.84E-02 | 2.27E-03 | 5.80E-16 | 265753 | 0.025% | 65.52 |
| Ankle spacing width | rs4132228 | 3 | 64708114 | T | C | 2.90E-01 | 1.65E-02 | 2.48E-03 | 2.40E-11 | 265753 | 0.017% | 44.61 |
| Ankle spacing width | rs28447555 | 3 | 107385668 | T | C | 1.80E-01 | 1.65E-02 | 2.95E-03 | 2.00E-08 | 265753 | 0.012% | 31.49 |
| Ankle spacing width | rs62295779 | 3 | 169868784 | G | A | 3.71E-02 | -3.41E-02 | 6.18E-03 | 3.30E-08 | 265753 | 0.011% | 30.54 |
| Ankle spacing width | rs512692 | 3 | 172170842 | T | A | 2.86E-01 | 1.62E-02 | 2.50E-03 | 9.90E-11 | 265753 | 0.016% | 41.83 |
| Ankle spacing width | rs1860968 | 3 | 37091325 | A | G | 4.57E-01 | -1.30E-02 | 2.26E-03 | 1.00E-08 | 265753 | 0.012% | 32.83 |
| Ankle spacing width | rs7653309 | 3 | 142479007 | T | G | 2.15E-01 | 1.64E-02 | 2.75E-03 | 2.50E-09 | 265753 | 0.013% | 35.56 |
| Ankle spacing width | rs355772 | 3 | 154036706 | A | G | 3.93E-01 | 1.66E-02 | 2.31E-03 | 7.50E-13 | 265753 | 0.019% | 51.40 |
| Ankle spacing width | rs4684789 | 3 | 11597087 | T | G | 3.03E-01 | 1.52E-02 | 2.46E-03 | 6.00E-10 | 265753 | 0.014% | 38.31 |
| Ankle spacing width | rs1185783 | 3 | 12490986 | G | A | 8.94E-01 | -2.61E-02 | 3.68E-03 | 1.40E-12 | 265753 | 0.019% | 50.18 |
| Ankle spacing width | rs17275273 | 3 | 64944311 | C | T | 1.27E-01 | 6.78E-02 | 3.41E-03 | 4.80E-88 | 265753 | 0.149% | 395.69 |
| Ankle spacing width | rs11923371 | 3 | 99515556 | G | A | 9.44E-02 | -2.17E-02 | 3.86E-03 | 2.00E-08 | 265753 | 0.012% | 31.49 |
| Ankle spacing width | rs10018649 | 4 | 2248264 | G | C | 9.61E-02 | -2.30E-02 | 3.83E-03 | 2.00E-09 | 265753 | 0.014% | 35.97 |
| Ankle spacing width | rs36090882 | 4 | 54379392 | A | C | 1.42E-01 | 2.02E-02 | 3.27E-03 | 6.70E-10 | 265753 | 0.014% | 38.11 |
| Ankle spacing width | rs140557441 | 4 | 87659122 | T | C | 1.58E-02 | -6.05E-02 | 9.10E-03 | 3.10E-11 | 265753 | 0.017% | 44.13 |
| Ankle spacing width | rs2647268 | 4 | 106211443 | G | A | 3.85E-01 | -1.91E-02 | 2.32E-03 | 2.20E-16 | 265753 | 0.025% | 67.38 |
| Ankle spacing width | rs113061374 | 4 | 1052662 | T | C | 4.81E-02 | -3.59E-02 | 5.29E-03 | 1.10E-11 | 265753 | 0.017% | 46.09 |
| Ankle spacing width | rs2131354 | 4 | 145599908 | A | G | 5.26E-01 | 2.46E-02 | 2.26E-03 | 1.60E-27 | 265753 | 0.044% | 118.19 |
| Ankle spacing width | rs17060428 | 4 | 175167253 | A | G | 1.81E-01 | -2.73E-02 | 2.93E-03 | 1.00E-20 | 265753 | 0.033% | 87.07 |
| Ankle spacing width | rs13130484 | 4 | 45175691 | T | C | 4.34E-01 | 1.43E-02 | 2.28E-03 | 3.40E-10 | 265753 | 0.015% | 39.43 |
| Ankle spacing width | rs4693210 | 4 | 89208565 | G | A | 4.38E-01 | 1.32E-02 | 2.28E-03 | 6.50E-09 | 265753 | 0.013% | 33.69 |
| Ankle spacing width | rs7671110 | 4 | 17874089 | T | C | 1.58E-01 | -3.00E-02 | 3.09E-03 | 2.70E-22 | 265753 | 0.035% | 94.31 |
| Ankle spacing width | rs1401718 | 4 | 52801096 | T | C | 2.63E-01 | 1.95E-02 | 2.56E-03 | 2.90E-14 | 265753 | 0.022% | 57.80 |
| Ankle spacing width | rs34848742 | 4 | 123828042 | G | T | 7.88E-01 | -3.31E-02 | 2.76E-03 | 3.30E-33 | 265753 | 0.054% | 144.15 |
| Ankle spacing width | rs72678990 | 4 | 124070923 | T | G | 3.98E-02 | -6.36E-02 | 5.78E-03 | 4.00E-28 | 265753 | 0.045% | 120.92 |
| Ankle spacing width | rs2702544 | 4 | 15120587 | G | A | 6.59E-01 | -1.80E-02 | 2.38E-03 | 4.70E-14 | 265753 | 0.021% | 56.84 |
| Ankle spacing width | rs1583974 | 4 | 100287812 | G | C | 4.15E-01 | 1.27E-02 | 2.30E-03 | 3.60E-08 | 265753 | 0.011% | 30.33 |
| Ankle spacing width | rs769246 | 4 | 122741343 | G | A | 3.29E-01 | 1.32E-02 | 2.40E-03 | 4.30E-08 | 265753 | 0.011% | 30.02 |
| Ankle spacing width | rs1037599 | 5 | 97783491 | G | T | 4.66E-01 | 1.81E-02 | 2.28E-03 | 2.20E-15 | 265753 | 0.024% | 62.87 |
| Ankle spacing width | rs4706020 | 5 | 130674076 | A | G | 3.78E-01 | 1.59E-02 | 2.34E-03 | 9.30E-12 | 265753 | 0.017% | 46.48 |
| Ankle spacing width | rs17666172 | 5 | 178503220 | C | T | 2.82E-01 | -1.63E-02 | 2.51E-03 | 7.40E-11 | 265753 | 0.016% | 42.41 |
| Ankle spacing width | rs1858138 | 5 | 42395281 | T | A | 5.51E-01 | 2.75E-02 | 2.27E-03 | 8.20E-34 | 265753 | 0.055% | 146.91 |
| Ankle spacing width | rs458036 | 5 | 55816081 | C | A | 7.62E-01 | 1.53E-02 | 2.65E-03 | 8.00E-09 | 265753 | 0.013% | 33.27 |
| Ankle spacing width | rs60940635 | 5 | 122755221 | G | A | 4.39E-01 | -2.44E-02 | 2.28E-03 | 8.80E-27 | 265753 | 0.043% | 114.77 |
| Ankle spacing width | rs1952655 | 5 | 157783484 | G | A | 3.26E-01 | -1.37E-02 | 2.41E-03 | 1.20E-08 | 265753 | 0.012% | 32.50 |
| Ankle spacing width | rs13189570 | 5 | 158182309 | T | C | 2.40E-01 | -2.88E-02 | 2.65E-03 | 1.50E-27 | 265753 | 0.044% | 118.24 |
| Ankle spacing width | rs2444779 | 5 | 43203125 | C | T | 2.86E-01 | 1.40E-02 | 2.51E-03 | 2.20E-08 | 265753 | 0.012% | 31.27 |
| Ankle spacing width | rs27279 | 5 | 50238519 | T | C | 5.21E-01 | 3.05E-02 | 2.46E-03 | 3.00E-35 | 265753 | 0.058% | 153.48 |
| Ankle spacing width | rs34912177 | 5 | 127932957 | C | A | 1.43E-01 | -2.04E-02 | 3.23E-03 | 3.00E-10 | 265753 | 0.015% | 39.68 |
| Ankle spacing width | rs572289 | 5 | 172328680 | C | T | 6.96E-01 | 1.58E-02 | 2.50E-03 | 2.40E-10 | 265753 | 0.015% | 40.12 |
| Ankle spacing width | rs2964131 | 5 | 5492718 | T | A | 6.43E-01 | -1.33E-02 | 2.36E-03 | 1.50E-08 | 265753 | 0.012% | 32.03 |
| Ankle spacing width | rs13178364 | 5 | 51203229 | A | G | 8.13E-01 | 2.14E-02 | 2.92E-03 | 2.70E-13 | 265753 | 0.020% | 53.38 |
| Ankle spacing width | rs31211 | 5 | 134363145 | A | G | 2.51E-01 | -4.92E-02 | 2.60E-03 | 8.30E-80 | 265753 | 0.134% | 357.84 |
| Ankle spacing width | rs258789 | 5 | 142542013 | G | A | 2.80E-01 | 1.57E-02 | 2.54E-03 | 6.40E-10 | 265753 | 0.014% | 38.20 |
| Ankle spacing width | rs6897617 | 5 | 173301982 | A | G | 2.88E-01 | -2.13E-02 | 2.49E-03 | 1.20E-17 | 265753 | 0.028% | 73.11 |
| Ankle spacing width | rs9623 | 5 | 67597321 | C | T | 4.59E-01 | -1.54E-02 | 2.26E-03 | 8.90E-12 | 265753 | 0.018% | 46.55 |
| Ankle spacing width | rs252748 | 5 | 77390494 | T | C | 5.92E-01 | -1.55E-02 | 2.30E-03 | 1.90E-11 | 265753 | 0.017% | 45.08 |
| Ankle spacing width | rs3922654 | 5 | 77684726 | A | G | 2.04E-01 | 1.75E-02 | 2.80E-03 | 3.60E-10 | 265753 | 0.015% | 39.30 |
| Ankle spacing width | rs10040658 | 5 | 139051015 | A | G | 3.71E-01 | 2.49E-02 | 2.35E-03 | 2.60E-26 | 265753 | 0.042% | 112.64 |
| Ankle spacing width | rs62396185 | 6 | 26180634 | C | G | 2.60E-01 | -2.83E-02 | 2.57E-03 | 2.90E-28 | 265753 | 0.046% | 121.52 |
| Ankle spacing width | rs17207671 | 6 | 31820616 | A | G | 1.01E-01 | -4.48E-02 | 3.74E-03 | 4.20E-33 | 265753 | 0.054% | 143.68 |
| Ankle spacing width | rs998584 | 6 | 43757896 | A | C | 4.83E-01 | -2.31E-02 | 2.26E-03 | 1.60E-24 | 265753 | 0.039% | 104.52 |
| Ankle spacing width | rs2209810 | 6 | 85364776 | A | G | 4.59E-01 | -3.06E-02 | 2.26E-03 | 1.00E-41 | 265753 | 0.069% | 183.15 |
| Ankle spacing width | rs505227 | 6 | 133592683 | C | T | 7.06E-01 | 2.55E-02 | 2.48E-03 | 9.10E-25 | 265753 | 0.040% | 105.58 |
| Ankle spacing width | rs9322332 | 6 | 152166801 | A | C | 4.55E-01 | 1.37E-02 | 2.26E-03 | 1.40E-09 | 265753 | 0.014% | 36.62 |
| Ankle spacing width | rs9405152 | 6 | 1868239 | C | T | 6.12E-01 | -1.49E-02 | 2.37E-03 | 3.40E-10 | 265753 | 0.015% | 39.46 |
| Ankle spacing width | rs1294438 | 6 | 6752059 | T | C | 3.55E-01 | 1.45E-02 | 2.38E-03 | 1.30E-09 | 265753 | 0.014% | 36.89 |
| Ankle spacing width | rs72868839 | 6 | 55624017 | T | A | 6.96E-02 | -6.73E-02 | 4.43E-03 | 5.20E-52 | 265753 | 0.087% | 230.26 |
| Ankle spacing width | rs150445982 | 6 | 55676621 | T | C | 2.28E-02 | -7.79E-02 | 7.90E-03 | 6.20E-23 | 265753 | 0.037% | 97.21 |
| Ankle spacing width | rs1398885 | 6 | 83460817 | G | T | 4.14E-01 | -1.34E-02 | 2.29E-03 | 4.10E-09 | 265753 | 0.013% | 34.57 |
| Ankle spacing width | rs652378 | 6 | 153466959 | C | T | 5.27E-01 | -1.27E-02 | 2.26E-03 | 2.00E-08 | 265753 | 0.012% | 31.52 |
| Ankle spacing width | rs2206943 | 6 | 160353822 | T | C | 2.78E-01 | 1.44E-02 | 2.52E-03 | 1.00E-08 | 265753 | 0.012% | 32.75 |
| Ankle spacing width | rs17630640 | 6 | 164107529 | G | A | 1.31E-01 | 2.78E-02 | 3.34E-03 | 8.90E-17 | 265753 | 0.026% | 69.21 |
| Ankle spacing width | rs9379130 | 6 | 7702659 | C | G | 4.90E-01 | 2.09E-02 | 2.27E-03 | 2.70E-20 | 265753 | 0.032% | 85.22 |
| Ankle spacing width | rs9358276 | 6 | 19806259 | C | T | 1.08E-01 | -3.42E-02 | 3.66E-03 | 9.30E-21 | 265753 | 0.033% | 87.31 |
| Ankle spacing width | rs3129116 | 6 | 29093788 | G | A | 6.10E-01 | 1.82E-02 | 2.31E-03 | 2.70E-15 | 265753 | 0.024% | 62.49 |
| Ankle spacing width | rs9272302 | 6 | 32603854 | T | C | 1.65E-01 | 2.38E-02 | 3.16E-03 | 5.20E-14 | 265753 | 0.021% | 56.67 |
| Ankle spacing width | rs33966734 | 6 | 41903798 | A | C | 1.36E-02 | -9.50E-02 | 9.86E-03 | 5.40E-22 | 265753 | 0.035% | 92.92 |
| Ankle spacing width | rs2153960 | 6 | 108988184 | A | G | 7.11E-01 | 2.57E-02 | 2.48E-03 | 3.30E-25 | 265753 | 0.040% | 107.61 |
| Ankle spacing width | rs9320287 | 6 | 109744690 | C | T | 3.49E-01 | -1.37E-02 | 2.36E-03 | 6.20E-09 | 265753 | 0.013% | 33.77 |
| Ankle spacing width | rs55996418 | 6 | 131422970 | T | C | 3.30E-01 | -1.68E-02 | 2.42E-03 | 4.50E-12 | 265753 | 0.018% | 47.90 |
| Ankle spacing width | rs9394569 | 6 | 12119885 | G | A | 3.52E-01 | -1.31E-02 | 2.36E-03 | 3.10E-08 | 265753 | 0.012% | 30.62 |
| Ankle spacing width | rs56348792 | 6 | 34161299 | T | C | 1.28E-01 | 2.41E-02 | 3.39E-03 | 1.20E-12 | 265753 | 0.019% | 50.51 |
| Ankle spacing width | rs1417391 | 6 | 54071413 | T | C | 6.48E-01 | -1.67E-02 | 2.37E-03 | 1.60E-12 | 265753 | 0.019% | 49.94 |
| Ankle spacing width | rs194633 | 6 | 82520021 | A | C | 4.42E-01 | -1.28E-02 | 2.27E-03 | 1.80E-08 | 265753 | 0.012% | 31.65 |
| Ankle spacing width | rs313185 | 6 | 86027615 | A | G | 1.98E-01 | -1.97E-02 | 2.82E-03 | 2.50E-12 | 265753 | 0.018% | 49.01 |
| Ankle spacing width | rs590015 | 6 | 139824992 | A | G | 5.00E-01 | 1.29E-02 | 2.25E-03 | 1.00E-08 | 265753 | 0.012% | 32.79 |
| Ankle spacing width | rs8180684 | 6 | 143200936 | T | C | 2.92E-01 | -1.83E-02 | 2.49E-03 | 1.80E-13 | 265753 | 0.020% | 54.27 |
| Ankle spacing width | rs6901077 | 6 | 41989718 | G | A | 7.55E-01 | -1.77E-02 | 2.62E-03 | 1.40E-11 | 265753 | 0.017% | 45.69 |
| Ankle spacing width | rs1546296 | 6 | 55266323 | C | A | 7.47E-01 | 3.16E-02 | 2.59E-03 | 2.40E-34 | 265753 | 0.056% | 149.35 |
| Ankle spacing width | rs17755849 | 6 | 130190757 | A | G | 3.83E-01 | -1.30E-02 | 2.33E-03 | 2.50E-08 | 265753 | 0.012% | 31.08 |
| Ankle spacing width | rs7451021 | 6 | 130381246 | C | T | 6.89E-01 | -3.65E-02 | 2.43E-03 | 7.10E-51 | 265753 | 0.085% | 225.08 |
| Ankle spacing width | rs2237477 | 7 | 50745307 | T | C | 3.07E-01 | 1.45E-02 | 2.45E-03 | 3.20E-09 | 265753 | 0.013% | 35.07 |
| Ankle spacing width | rs62621812 | 7 | 127015083 | A | G | 2.03E-02 | 1.06E-01 | 8.23E-03 | 1.20E-37 | 265753 | 0.062% | 164.43 |
| Ankle spacing width | rs315860 | 7 | 33926351 | G | A | 5.50E-01 | -1.42E-02 | 2.28E-03 | 4.10E-10 | 265753 | 0.015% | 39.07 |
| Ankle spacing width | rs13238996 | 7 | 74069645 | G | A | 2.42E-01 | 1.89E-02 | 2.63E-03 | 6.60E-13 | 265753 | 0.019% | 51.67 |
| Ankle spacing width | rs6973656 | 7 | 77422583 | G | A | 3.97E-01 | 1.43E-02 | 2.30E-03 | 5.70E-10 | 265753 | 0.014% | 38.42 |
| Ankle spacing width | rs10269774 | 7 | 92253972 | A | G | 3.26E-01 | 3.87E-02 | 2.41E-03 | 6.30E-58 | 265753 | 0.097% | 257.42 |
| Ankle spacing width | rs144278075 | 7 | 106170086 | C | T | 2.35E-02 | -4.30E-02 | 7.50E-03 | 1.00E-08 | 265753 | 0.012% | 32.81 |
| Ankle spacing width | rs11770163 | 7 | 116417848 | C | G | 3.32E-01 | 1.63E-02 | 2.40E-03 | 1.10E-11 | 265753 | 0.017% | 46.09 |
| Ankle spacing width | rs798502 | 7 | 2789880 | C | A | 2.97E-01 | -2.27E-02 | 2.47E-03 | 3.70E-20 | 265753 | 0.032% | 84.58 |
| Ankle spacing width | rs17156919 | 7 | 28650128 | A | G | 1.80E-01 | 2.28E-02 | 2.94E-03 | 9.00E-15 | 265753 | 0.023% | 60.11 |
| Ankle spacing width | rs822551 | 7 | 148650375 | G | A | 8.12E-01 | 2.20E-02 | 2.92E-03 | 4.50E-14 | 265753 | 0.021% | 56.95 |
| Ankle spacing width | rs10233384 | 7 | 33705225 | G | A | 2.71E-01 | 1.48E-02 | 2.54E-03 | 4.80E-09 | 265753 | 0.013% | 34.27 |
| Ankle spacing width | rs75728676 | 7 | 46633786 | A | T | 5.16E-01 | -1.44E-02 | 2.26E-03 | 1.70E-10 | 265753 | 0.015% | 40.73 |
| Ankle spacing width | rs2106930 | 7 | 92453892 | A | T | 1.41E-01 | 2.37E-02 | 3.27E-03 | 4.20E-13 | 265753 | 0.020% | 52.55 |
| Ankle spacing width | rs2252074 | 7 | 104594253 | G | T | 3.99E-01 | 1.42E-02 | 2.31E-03 | 7.10E-10 | 265753 | 0.014% | 38.00 |
| Ankle spacing width | rs12154627 | 7 | 130422934 | C | T | 4.96E-01 | -1.74E-02 | 2.28E-03 | 2.40E-14 | 265753 | 0.022% | 58.17 |
| Ankle spacing width | rs38205 | 7 | 15913588 | C | A | 6.23E-01 | 1.87E-02 | 2.35E-03 | 1.90E-15 | 265753 | 0.024% | 63.16 |
| Ankle spacing width | rs56376645 | 7 | 25886125 | A | G | 2.48E-01 | 3.00E-02 | 2.61E-03 | 2.00E-30 | 265753 | 0.049% | 131.39 |
| Ankle spacing width | rs1859165 | 7 | 27218468 | C | T | 9.06E-01 | 4.70E-02 | 3.86E-03 | 5.50E-34 | 265753 | 0.056% | 147.71 |
| Ankle spacing width | rs961725 | 7 | 27658070 | A | G | 7.28E-01 | -6.14E-02 | 2.53E-03 | 9.91E-130 | 265753 | 0.220% | 587.24 |
| Ankle spacing width | rs11525873 | 7 | 138817193 | C | T | 9.80E-02 | -2.19E-02 | 3.80E-03 | 8.40E-09 | 265753 | 0.012% | 33.18 |
| Ankle spacing width | rs4725984 | 7 | 150668514 | C | T | 6.42E-01 | 2.10E-02 | 2.37E-03 | 9.00E-19 | 265753 | 0.029% | 78.27 |
| Ankle spacing width | rs62493792 | 8 | 9170786 | A | T | 2.36E-01 | 1.92E-02 | 2.69E-03 | 7.90E-13 | 265753 | 0.019% | 51.30 |
| Ankle spacing width | rs76283376 | 8 | 23659920 | A | G | 8.34E-02 | -2.43E-02 | 4.10E-03 | 3.00E-09 | 265753 | 0.013% | 35.16 |
| Ankle spacing width | rs7837090 | 8 | 74221406 | G | C | 1.96E-01 | 1.97E-02 | 2.84E-03 | 3.80E-12 | 265753 | 0.018% | 48.21 |
| Ankle spacing width | rs7844179 | 8 | 74629388 | C | G | 2.13E-01 | 2.56E-02 | 2.76E-03 | 1.90E-20 | 265753 | 0.032% | 85.88 |
| Ankle spacing width | rs4469491 | 8 | 72387665 | T | C | 4.99E-01 | 1.66E-02 | 2.26E-03 | 2.00E-13 | 265753 | 0.020% | 54.03 |
| Ankle spacing width | rs969649 | 8 | 96666179 | T | C | 7.73E-01 | 3.16E-02 | 2.71E-03 | 1.60E-31 | 265753 | 0.051% | 136.47 |
| Ankle spacing width | rs4909323 | 8 | 135637692 | T | G | 3.95E-01 | -2.78E-02 | 2.31E-03 | 1.60E-33 | 265753 | 0.055% | 145.56 |
| Ankle spacing width | rs2942202 | 8 | 23418444 | C | A | 4.96E-01 | -1.64E-02 | 2.27E-03 | 5.20E-13 | 265753 | 0.020% | 52.12 |
| Ankle spacing width | rs16885613 | 8 | 36848357 | C | T | 1.68E-01 | 2.45E-02 | 3.02E-03 | 4.70E-16 | 265753 | 0.025% | 65.92 |
| Ankle spacing width | rs7821279 | 8 | 96491204 | A | G | 4.85E-01 | 1.86E-02 | 2.26E-03 | 2.10E-16 | 265753 | 0.025% | 67.51 |
| Ankle spacing width | rs6985162 | 8 | 109009951 | C | A | 3.59E-01 | 1.58E-02 | 2.39E-03 | 3.90E-11 | 265753 | 0.016% | 43.66 |
| Ankle spacing width | rs11783086 | 8 | 123980448 | C | T | 3.55E-01 | 1.59E-02 | 2.37E-03 | 2.30E-11 | 265753 | 0.017% | 44.71 |
| Ankle spacing width | rs3753121 | 8 | 143926380 | C | T | 5.56E-01 | 1.84E-02 | 2.27E-03 | 4.90E-16 | 265753 | 0.025% | 65.83 |
| Ankle spacing width | rs4073455 | 8 | 144978607 | G | T | 5.77E-01 | -2.11E-02 | 2.29E-03 | 3.00E-20 | 265753 | 0.032% | 84.97 |
| Ankle spacing width | rs10099531 | 8 | 49551946 | T | C | 7.11E-01 | -1.83E-02 | 2.50E-03 | 2.60E-13 | 265753 | 0.020% | 53.46 |
| Ankle spacing width | rs72656010 | 8 | 57122215 | C | T | 1.32E-01 | -4.44E-02 | 3.34E-03 | 2.90E-40 | 265753 | 0.066% | 176.47 |
| Ankle spacing width | rs2732106 | 8 | 71923839 | A | G | 5.23E-01 | 2.05E-02 | 2.26E-03 | 1.40E-19 | 265753 | 0.031% | 82.00 |
| Ankle spacing width | rs7015733 | 8 | 100911581 | C | T | 2.03E-01 | 1.55E-02 | 2.82E-03 | 3.70E-08 | 265753 | 0.011% | 30.30 |
| Ankle spacing width | rs10955753 | 8 | 116562169 | A | C | 5.52E-01 | -1.79E-02 | 2.28E-03 | 3.90E-15 | 265753 | 0.023% | 61.73 |
| Ankle spacing width | rs970945 | 9 | 12738641 | T | C | 3.03E-01 | -1.42E-02 | 2.46E-03 | 6.70E-09 | 265753 | 0.013% | 33.62 |
| Ankle spacing width | rs10993097 | 9 | 96977298 | T | A | 3.12E-01 | -1.56E-02 | 2.45E-03 | 2.00E-10 | 265753 | 0.015% | 40.48 |
| Ankle spacing width | rs28701981 | 9 | 98217581 | C | T | 3.50E-01 | 2.38E-02 | 2.38E-03 | 1.20E-23 | 265753 | 0.038% | 100.52 |
| Ankle spacing width | rs625647 | 9 | 110957231 | G | T | 4.60E-01 | 1.64E-02 | 2.28E-03 | 5.80E-13 | 265753 | 0.020% | 51.90 |
| Ankle spacing width | rs10781474 | 9 | 80562978 | T | C | 2.75E-01 | -2.25E-02 | 2.54E-03 | 8.80E-19 | 265753 | 0.029% | 78.31 |
| Ankle spacing width | rs61628776 | 9 | 95515900 | G | A | 1.41E-01 | -2.32E-02 | 3.25E-03 | 1.00E-12 | 265753 | 0.019% | 50.83 |
| Ankle spacing width | rs12005413 | 9 | 118500860 | A | C | 7.69E-02 | -4.69E-02 | 4.30E-03 | 8.90E-28 | 265753 | 0.045% | 119.32 |
| Ankle spacing width | rs11185670 | 9 | 137115785 | C | A | 1.35E-01 | 2.18E-02 | 3.34E-03 | 7.20E-11 | 265753 | 0.016% | 42.46 |
| Ankle spacing width | rs6478556 | 9 | 100842005 | T | C | 1.96E-01 | -1.65E-02 | 2.85E-03 | 6.80E-09 | 265753 | 0.013% | 33.58 |
| Ankle spacing width | rs12683397 | 9 | 112428589 | T | C | 1.89E-01 | 1.84E-02 | 2.89E-03 | 1.70E-10 | 265753 | 0.015% | 40.76 |
| Ankle spacing width | rs10780905 | 9 | 90841066 | A | G | 5.87E-01 | -1.50E-02 | 2.31E-03 | 8.50E-11 | 265753 | 0.016% | 42.14 |
| Ankle spacing width | rs116920211 | 9 | 126209230 | T | A | 3.37E-02 | 3.85E-02 | 6.31E-03 | 1.00E-09 | 265753 | 0.014% | 37.24 |
| Ankle spacing width | rs10979347 | 9 | 111175475 | G | T | 2.31E-01 | -2.09E-02 | 2.70E-03 | 9.60E-15 | 265753 | 0.023% | 59.97 |
| Ankle spacing width | rs78841852 | 9 | 119225730 | C | T | 4.23E-02 | -5.74E-02 | 5.64E-03 | 2.80E-24 | 265753 | 0.039% | 103.33 |
| Ankle spacing width | rs12552167 | 9 | 139322927 | T | C | 2.84E-01 | 1.61E-02 | 2.50E-03 | 1.20E-10 | 265753 | 0.016% | 41.50 |
| Ankle spacing width | rs2642280 | 10 | 27934156 | G | A | 4.61E-01 | 1.35E-02 | 2.27E-03 | 2.50E-09 | 265753 | 0.013% | 35.53 |
| Ankle spacing width | rs72814517 | 10 | 72440653 | T | C | 2.40E-01 | -2.15E-02 | 2.66E-03 | 6.90E-16 | 265753 | 0.025% | 65.15 |
| Ankle spacing width | rs662115 | 10 | 89772989 | T | C | 4.47E-01 | 1.62E-02 | 2.28E-03 | 1.20E-12 | 265753 | 0.019% | 50.54 |
| Ankle spacing width | rs603424 | 10 | 102075479 | A | G | 1.73E-01 | 7.75E-02 | 2.98E-03 | 3.70E-149 | 265753 | 0.254% | 676.57 |
| Ankle spacing width | rs11200607 | 10 | 124154626 | T | C | 2.47E-01 | -1.56E-02 | 2.62E-03 | 2.40E-09 | 265753 | 0.013% | 35.65 |
| Ankle spacing width | rs2132323 | 10 | 29226450 | G | A | 5.23E-01 | 2.36E-02 | 2.28E-03 | 3.50E-25 | 265753 | 0.040% | 107.50 |
| Ankle spacing width | rs7091291 | 10 | 95310539 | C | T | 1.62E-01 | 1.72E-02 | 3.06E-03 | 2.00E-08 | 265753 | 0.012% | 31.47 |
| Ankle spacing width | rs3802725 | 10 | 102673063 | T | G | 4.29E-01 | 1.38E-02 | 2.29E-03 | 1.40E-09 | 265753 | 0.014% | 36.69 |
| Ankle spacing width | rs11199828 | 10 | 122964630 | A | G | 7.25E-01 | 1.49E-02 | 2.55E-03 | 5.00E-09 | 265753 | 0.013% | 34.19 |
| Ankle spacing width | rs7077303 | 10 | 62753643 | C | T | 5.13E-01 | -1.26E-02 | 2.27E-03 | 2.90E-08 | 265753 | 0.012% | 30.79 |
| Ankle spacing width | rs11188956 | 10 | 98630588 | G | A | 1.06E-01 | 2.89E-02 | 3.68E-03 | 3.60E-15 | 265753 | 0.023% | 61.88 |
| Ankle spacing width | rs10795049 | 10 | 3493718 | T | C | 2.31E-01 | -1.55E-02 | 2.68E-03 | 8.00E-09 | 265753 | 0.013% | 33.26 |
| Ankle spacing width | rs7922843 | 10 | 25043853 | A | G | 6.48E-01 | -1.32E-02 | 2.37E-03 | 2.30E-08 | 265753 | 0.012% | 31.25 |
| Ankle spacing width | rs12411640 | 10 | 28806226 | A | G | 2.80E-01 | -1.69E-02 | 2.51E-03 | 1.60E-11 | 265753 | 0.017% | 45.35 |
| Ankle spacing width | rs79063713 | 10 | 101833449 | T | C | 1.39E-02 | 7.28E-02 | 9.74E-03 | 7.80E-14 | 265753 | 0.021% | 55.86 |
| Ankle spacing width | rs7103411 | 11 | 27700125 | T | C | 7.91E-01 | 3.20E-02 | 2.78E-03 | 1.40E-30 | 265753 | 0.050% | 132.14 |
| Ankle spacing width | rs614520 | 11 | 65664006 | A | G | 6.39E-01 | -1.95E-02 | 2.40E-03 | 4.90E-16 | 265753 | 0.025% | 65.83 |
| Ankle spacing width | rs2510382 | 11 | 68391731 | A | G | 1.66E-01 | -2.12E-02 | 3.04E-03 | 3.10E-12 | 265753 | 0.018% | 48.61 |
| Ankle spacing width | rs67330701 | 11 | 69079707 | T | C | 9.25E-02 | 2.53E-02 | 4.04E-03 | 4.20E-10 | 265753 | 0.015% | 39.02 |
| Ankle spacing width | rs112493 | 11 | 1991603 | A | G | 3.48E-01 | -1.99E-02 | 2.38E-03 | 5.20E-17 | 265753 | 0.026% | 70.26 |
| Ankle spacing width | rs3213217 | 11 | 2157793 | T | A | 2.33E-01 | -1.80E-02 | 2.69E-03 | 2.30E-11 | 265753 | 0.017% | 44.73 |
| Ankle spacing width | rs2237967 | 11 | 17492664 | C | T | 5.14E-01 | 1.76E-02 | 2.26E-03 | 8.80E-15 | 265753 | 0.023% | 60.15 |
| Ankle spacing width | rs1562782 | 11 | 10342711 | G | A | 4.09E-01 | -1.96E-02 | 2.30E-03 | 1.80E-17 | 265753 | 0.027% | 72.38 |
| Ankle spacing width | rs12807220 | 11 | 102077200 | A | G | 3.62E-01 | -1.78E-02 | 2.37E-03 | 4.50E-14 | 265753 | 0.021% | 56.95 |
| Ankle spacing width | rs7931091 | 11 | 528575 | G | A | 2.79E-01 | -1.79E-02 | 2.52E-03 | 1.40E-12 | 265753 | 0.019% | 50.25 |
| Ankle spacing width | rs597053 | 11 | 30441946 | C | T | 3.03E-01 | 1.36E-02 | 2.46E-03 | 3.60E-08 | 265753 | 0.011% | 30.34 |
| Ankle spacing width | rs7120548 | 11 | 47662932 | C | T | 3.01E-01 | -1.78E-02 | 2.46E-03 | 4.30E-13 | 265753 | 0.020% | 52.49 |
| Ankle spacing width | rs198428 | 11 | 61489705 | T | A | 6.16E-01 | -1.61E-02 | 2.32E-03 | 4.70E-12 | 265753 | 0.018% | 47.82 |
| Ankle spacing width | rs1622638 | 11 | 121800971 | A | G | 3.94E-01 | -2.48E-02 | 2.31E-03 | 6.20E-27 | 265753 | 0.043% | 115.47 |
| Ankle spacing width | rs12581860 | 12 | 9124190 | A | G | 4.17E-01 | 1.58E-02 | 2.32E-03 | 8.60E-12 | 265753 | 0.018% | 46.62 |
| Ankle spacing width | rs2446144 | 12 | 41845813 | T | C | 4.77E-01 | 1.62E-02 | 2.26E-03 | 9.30E-13 | 265753 | 0.019% | 50.98 |
| Ankle spacing width | rs10878292 | 12 | 65898179 | G | A | 3.93E-01 | -1.80E-02 | 2.32E-03 | 7.80E-15 | 265753 | 0.023% | 60.38 |
| Ankle spacing width | rs12819667 | 12 | 89763529 | T | C | 5.44E-01 | -1.36E-02 | 2.26E-03 | 1.90E-09 | 265753 | 0.014% | 36.05 |
| Ankle spacing width | rs11045239 | 12 | 20579694 | A | G | 4.00E-01 | -1.44E-02 | 2.32E-03 | 4.90E-10 | 265753 | 0.015% | 38.73 |
| Ankle spacing width | rs7309492 | 12 | 28073172 | A | T | 5.69E-01 | 1.25E-02 | 2.30E-03 | 4.90E-08 | 265753 | 0.011% | 29.76 |
| Ankle spacing width | rs11049486 | 12 | 28406157 | T | C | 5.50E-01 | -1.27E-02 | 2.27E-03 | 2.10E-08 | 265753 | 0.012% | 31.36 |
| Ankle spacing width | rs79573772 | 12 | 65964504 | C | T | 8.73E-02 | -5.74E-02 | 4.03E-03 | 5.50E-46 | 265753 | 0.076% | 202.64 |
| Ankle spacing width | rs11111093 | 12 | 102327309 | C | T | 1.26E-01 | 2.59E-02 | 3.41E-03 | 3.10E-14 | 265753 | 0.022% | 57.65 |
| Ankle spacing width | rs35756741 | 12 | 12868701 | T | C | 9.30E-02 | -2.93E-02 | 3.90E-03 | 5.70E-14 | 265753 | 0.021% | 56.49 |
| Ankle spacing width | rs2175723 | 12 | 26473675 | A | G | 2.36E-01 | -3.65E-02 | 2.66E-03 | 7.20E-43 | 265753 | 0.071% | 188.39 |
| Ankle spacing width | rs2640562 | 12 | 56466473 | T | C | 3.48E-01 | -1.62E-02 | 2.37E-03 | 9.70E-12 | 265753 | 0.017% | 46.38 |
| Ankle spacing width | rs2279666 | 12 | 64503028 | T | C | 4.58E-01 | -1.25E-02 | 2.28E-03 | 4.30E-08 | 265753 | 0.011% | 29.99 |
| Ankle spacing width | rs7963773 | 12 | 122950035 | G | A | 7.42E-01 | 1.65E-02 | 2.59E-03 | 1.90E-10 | 265753 | 0.015% | 40.56 |
| Ankle spacing width | rs76895963 | 12 | 4384844 | G | T | 2.05E-02 | 1.53E-01 | 8.79E-03 | 2.50E-68 | 265753 | 0.115% | 305.16 |
| Ankle spacing width | rs771648 | 12 | 77547182 | C | T | 5.70E-01 | -1.69E-02 | 2.29E-03 | 1.30E-13 | 265753 | 0.021% | 54.81 |
| Ankle spacing width | rs117081218 | 12 | 102339359 | A | G | 3.66E-02 | -4.49E-02 | 6.20E-03 | 4.20E-13 | 265753 | 0.020% | 52.54 |
| Ankle spacing width | rs139429176 | 12 | 121632160 | T | C | 1.24E-02 | -6.72E-02 | 1.03E-02 | 7.30E-11 | 265753 | 0.016% | 42.44 |
| Ankle spacing width | rs1271309 | 12 | 124820705 | G | A | 8.37E-01 | -2.15E-02 | 3.06E-03 | 2.10E-12 | 265753 | 0.019% | 49.38 |
| Ankle spacing width | rs7132908 | 12 | 50263148 | A | G | 3.84E-01 | 1.39E-02 | 2.32E-03 | 1.90E-09 | 265753 | 0.014% | 36.09 |
| Ankle spacing width | rs1038196 | 12 | 66343400 | C | G | 5.14E-01 | -4.81E-02 | 2.26E-03 | 6.70E-101 | 265753 | 0.171% | 454.74 |
| Ankle spacing width | rs9510177 | 13 | 22977789 | C | T | 7.17E-01 | -1.53E-02 | 2.53E-03 | 1.50E-09 | 265753 | 0.014% | 36.59 |
| Ankle spacing width | rs3116598 | 13 | 51099616 | T | C | 2.19E-01 | -4.83E-02 | 2.74E-03 | 1.10E-69 | 265753 | 0.117% | 311.41 |
| Ankle spacing width | rs837309 | 13 | 101202748 | T | C | 5.18E-01 | -2.04E-02 | 2.26E-03 | 2.20E-19 | 265753 | 0.030% | 81.01 |
| Ankle spacing width | rs7328085 | 13 | 91979910 | C | T | 3.57E-01 | 1.38E-02 | 2.37E-03 | 6.20E-09 | 265753 | 0.013% | 33.76 |
| Ankle spacing width | rs7986001 | 13 | 114830132 | T | C | 4.36E-01 | 1.41E-02 | 2.28E-03 | 6.20E-10 | 265753 | 0.014% | 38.26 |
| Ankle spacing width | rs2812208 | 13 | 50707087 | C | G | 2.08E-02 | 7.32E-02 | 7.94E-03 | 2.80E-20 | 265753 | 0.032% | 85.14 |
| Ankle spacing width | rs9535713 | 13 | 52162010 | T | C | 3.91E-01 | -1.38E-02 | 2.33E-03 | 3.30E-09 | 265753 | 0.013% | 35.02 |
| Ankle spacing width | rs1924936 | 13 | 78443297 | A | T | 7.74E-01 | 2.01E-02 | 2.71E-03 | 1.20E-13 | 265753 | 0.021% | 54.95 |
| Ankle spacing width | rs750598 | 13 | 111028978 | A | G | 3.38E-01 | 1.84E-02 | 2.40E-03 | 1.60E-14 | 265753 | 0.022% | 58.94 |
| Ankle spacing width | rs2104736 | 13 | 30990048 | T | C | 3.83E-01 | -1.54E-02 | 2.38E-03 | 8.30E-11 | 265753 | 0.016% | 42.18 |
| Ankle spacing width | rs497857 | 13 | 80917527 | C | T | 3.88E-01 | -1.35E-02 | 2.33E-03 | 7.50E-09 | 265753 | 0.013% | 33.41 |
| Ankle spacing width | rs3210043 | 14 | 23779877 | A | C | 1.63E-01 | 2.32E-02 | 3.07E-03 | 3.60E-14 | 265753 | 0.022% | 57.39 |
| Ankle spacing width | rs17126672 | 14 | 54058910 | T | C | 1.92E-01 | 3.30E-02 | 2.88E-03 | 3.20E-30 | 265753 | 0.049% | 130.52 |
| Ankle spacing width | rs28929474 | 14 | 94844947 | T | C | 1.98E-02 | 7.09E-02 | 8.14E-03 | 3.20E-18 | 265753 | 0.029% | 75.78 |
| Ankle spacing width | rs78267487 | 14 | 98182714 | G | C | 2.96E-01 | 1.42E-02 | 2.50E-03 | 1.40E-08 | 265753 | 0.012% | 32.19 |
| Ankle spacing width | rs11158820 | 14 | 70347348 | G | A | 6.87E-01 | -1.97E-02 | 2.47E-03 | 1.30E-15 | 265753 | 0.024% | 63.91 |
| Ankle spacing width | rs4904743 | 14 | 91534872 | T | C | 2.90E-01 | -2.17E-02 | 2.51E-03 | 5.00E-18 | 265753 | 0.028% | 74.87 |
| Ankle spacing width | rs10459527 | 14 | 24795862 | C | T | 7.42E-01 | -1.72E-02 | 2.61E-03 | 4.90E-11 | 265753 | 0.016% | 43.22 |
| Ankle spacing width | rs76337496 | 14 | 53693793 | C | T | 2.58E-01 | 1.71E-02 | 2.58E-03 | 3.10E-11 | 265753 | 0.017% | 44.10 |
| Ankle spacing width | rs12887636 | 14 | 29702590 | G | T | 3.47E-01 | -1.45E-02 | 2.39E-03 | 1.50E-09 | 265753 | 0.014% | 36.51 |
| Ankle spacing width | rs11158249 | 14 | 59605315 | C | G | 7.86E-01 | -1.95E-02 | 2.77E-03 | 2.00E-12 | 265753 | 0.019% | 49.45 |
| Ankle spacing width | rs2738265 | 14 | 54422399 | G | C | 5.42E-01 | -2.59E-02 | 2.27E-03 | 4.00E-30 | 265753 | 0.049% | 130.04 |
| Ankle spacing width | rs2370982 | 14 | 79890677 | T | C | 2.15E-01 | 1.76E-02 | 2.78E-03 | 2.30E-10 | 265753 | 0.015% | 40.20 |
| Ankle spacing width | rs9646139 | 14 | 98391624 | A | G | 5.44E-01 | 2.93E-02 | 2.27E-03 | 5.30E-38 | 265753 | 0.062% | 166.10 |
| Ankle spacing width | rs28597847 | 15 | 51405322 | G | A | 4.29E-01 | -1.60E-02 | 2.29E-03 | 2.90E-12 | 265753 | 0.018% | 48.77 |
| Ankle spacing width | rs991157 | 15 | 67419013 | C | T | 6.88E-01 | -1.44E-02 | 2.44E-03 | 3.70E-09 | 265753 | 0.013% | 34.76 |
| Ankle spacing width | rs112238647 | 15 | 79051705 | T | C | 6.39E-02 | 2.89E-02 | 4.72E-03 | 9.50E-10 | 265753 | 0.014% | 37.43 |
| Ankle spacing width | rs1894401 | 15 | 91429042 | A | G | 5.25E-01 | 1.97E-02 | 2.27E-03 | 4.40E-18 | 265753 | 0.028% | 75.13 |
| Ankle spacing width | rs2593169 | 15 | 52224522 | C | G | 5.68E-01 | -1.26E-02 | 2.29E-03 | 3.80E-08 | 265753 | 0.011% | 30.23 |
| Ankle spacing width | rs8030777 | 15 | 99403952 | T | C | 2.71E-01 | 1.62E-02 | 2.56E-03 | 2.50E-10 | 265753 | 0.015% | 40.03 |
| Ankle spacing width | rs2439386 | 15 | 67025403 | T | C | 7.18E-01 | -2.00E-02 | 2.51E-03 | 1.90E-15 | 265753 | 0.024% | 63.12 |
| Ankle spacing width | rs12910672 | 15 | 75922697 | T | C | 2.52E-01 | 2.37E-02 | 2.61E-03 | 1.00E-19 | 265753 | 0.031% | 82.60 |
| Ankle spacing width | rs12591069 | 15 | 99236672 | A | T | 2.58E-01 | -1.82E-02 | 2.59E-03 | 2.30E-12 | 265753 | 0.019% | 49.23 |
| Ankle spacing width | rs35929659 | 16 | 2165630 | C | T | 1.80E-01 | -2.35E-02 | 2.97E-03 | 2.30E-15 | 265753 | 0.024% | 62.79 |
| Ankle spacing width | rs62036658 | 16 | 28850371 | G | T | 4.02E-01 | 2.08E-02 | 2.31E-03 | 1.70E-19 | 265753 | 0.031% | 81.56 |
| Ankle spacing width | rs55872725 | 16 | 53809123 | T | C | 4.04E-01 | 3.69E-02 | 2.31E-03 | 1.80E-57 | 265753 | 0.096% | 255.34 |
| Ankle spacing width | rs8052905 | 16 | 73097663 | A | G | 5.71E-02 | 3.12E-02 | 4.91E-03 | 2.20E-10 | 265753 | 0.015% | 40.31 |
| Ankle spacing width | rs9927272 | 16 | 88346709 | A | G | 6.13E-01 | 1.37E-02 | 2.35E-03 | 6.30E-09 | 265753 | 0.013% | 33.73 |
| Ankle spacing width | rs4988483 | 16 | 1129010 | A | C | 5.67E-02 | -4.27E-02 | 5.01E-03 | 1.70E-17 | 265753 | 0.027% | 72.46 |
| Ankle spacing width | rs11076065 | 16 | 55074864 | C | G | 2.96E-01 | 1.42E-02 | 2.50E-03 | 1.40E-08 | 265753 | 0.012% | 32.20 |
| Ankle spacing width | rs2925979 | 16 | 81534790 | C | T | 7.00E-01 | -1.83E-02 | 2.47E-03 | 1.30E-13 | 265753 | 0.021% | 54.90 |
| Ankle spacing width | rs28529403 | 16 | 30134656 | C | T | 3.98E-01 | -2.92E-02 | 2.32E-03 | 2.30E-36 | 265753 | 0.060% | 158.60 |
| Ankle spacing width | rs72789541 | 16 | 15127534 | A | T | 2.96E-01 | -1.63E-02 | 2.48E-03 | 6.00E-11 | 265753 | 0.016% | 42.82 |
| Ankle spacing width | rs775208 | 16 | 70315911 | C | T | 5.08E-01 | 1.28E-02 | 2.27E-03 | 1.70E-08 | 265753 | 0.012% | 31.84 |
| Ankle spacing width | rs7186061 | 16 | 80934495 | C | A | 8.49E-01 | -2.65E-02 | 3.16E-03 | 4.50E-17 | 265753 | 0.027% | 70.53 |
| Ankle spacing width | rs1728394 | 16 | 86410525 | C | T | 2.83E-01 | 1.69E-02 | 2.51E-03 | 1.60E-11 | 265753 | 0.017% | 45.41 |
| Ankle spacing width | rs728868 | 16 | 50942445 | A | G | 3.15E-01 | 1.38E-02 | 2.44E-03 | 1.60E-08 | 265753 | 0.012% | 31.95 |
| Ankle spacing width | rs1498786 | 16 | 51527032 | T | A | 6.20E-01 | 1.74E-02 | 2.34E-03 | 1.10E-13 | 265753 | 0.021% | 55.24 |
| Ankle spacing width | rs79319469 | 17 | 2292883 | T | C | 1.93E-01 | -1.79E-02 | 2.86E-03 | 4.20E-10 | 265753 | 0.015% | 39.02 |
| Ankle spacing width | rs12051649 | 17 | 18117529 | G | C | 2.57E-01 | 1.48E-02 | 2.59E-03 | 1.00E-08 | 265753 | 0.012% | 32.84 |
| Ankle spacing width | rs315498 | 17 | 30020768 | T | C | 6.58E-01 | -1.69E-02 | 2.40E-03 | 1.60E-12 | 265753 | 0.019% | 49.90 |
| Ankle spacing width | rs16948744 | 17 | 48246162 | A | G | 3.59E-01 | -1.33E-02 | 2.36E-03 | 1.70E-08 | 265753 | 0.012% | 31.83 |
| Ankle spacing width | rs6788 | 17 | 68131526 | T | C | 1.12E-01 | -1.98E-02 | 3.60E-03 | 3.90E-08 | 265753 | 0.011% | 30.17 |
| Ankle spacing width | rs11655860 | 17 | 79411662 | A | G | 3.56E-01 | 2.82E-02 | 2.39E-03 | 2.60E-32 | 265753 | 0.053% | 140.02 |
| Ankle spacing width | rs1043515 | 17 | 36922196 | G | A | 5.66E-01 | 1.88E-02 | 2.28E-03 | 1.60E-16 | 265753 | 0.026% | 68.01 |
| Ankle spacing width | rs6847 | 17 | 46147807 | A | T | 2.03E-01 | 2.03E-02 | 2.81E-03 | 5.60E-13 | 265753 | 0.020% | 52.00 |
| Ankle spacing width | rs12453125 | 17 | 58046076 | G | A | 5.88E-01 | -2.03E-02 | 2.30E-03 | 1.30E-18 | 265753 | 0.029% | 77.60 |
| Ankle spacing width | rs956218 | 17 | 69145336 | A | G | 5.80E-01 | -2.20E-02 | 2.29E-03 | 8.60E-22 | 265753 | 0.035% | 92.02 |
| Ankle spacing width | rs72815304 | 17 | 2007004 | T | C | 3.79E-01 | -2.45E-02 | 2.33E-03 | 9.40E-26 | 265753 | 0.041% | 110.09 |
| Ankle spacing width | rs62072974 | 17 | 54228452 | T | A | 1.23E-01 | -2.80E-02 | 3.45E-03 | 4.90E-16 | 265753 | 0.025% | 65.84 |
| Ankle spacing width | rs236586 | 17 | 68203546 | G | A | 4.88E-01 | -1.36E-02 | 2.26E-03 | 1.70E-09 | 265753 | 0.014% | 36.23 |
| Ankle spacing width | rs4246443 | 17 | 79978594 | C | T | 6.92E-01 | 1.85E-02 | 2.45E-03 | 4.20E-14 | 265753 | 0.021% | 57.06 |
| Ankle spacing width | rs35850753 | 17 | 7578671 | T | C | 1.98E-02 | 6.62E-02 | 8.27E-03 | 1.20E-15 | 265753 | 0.024% | 64.14 |
| Ankle spacing width | rs11651289 | 17 | 62013474 | T | C | 2.48E-01 | 1.82E-02 | 2.63E-03 | 5.10E-12 | 265753 | 0.018% | 47.65 |
| Ankle spacing width | rs35663354 | 17 | 79045557 | T | G | 5.33E-01 | -1.36E-02 | 2.29E-03 | 2.90E-09 | 265753 | 0.013% | 35.28 |
| Ankle spacing width | rs150746336 | 17 | 79532081 | T | C | 1.97E-02 | 5.19E-02 | 8.50E-03 | 1.00E-09 | 265753 | 0.014% | 37.32 |
| Ankle spacing width | rs573187 | 17 | 14401450 | T | A | 5.01E-01 | 1.40E-02 | 2.26E-03 | 5.70E-10 | 265753 | 0.014% | 38.43 |
| Ankle spacing width | rs62070648 | 17 | 29210595 | A | G | 2.68E-01 | -2.85E-02 | 2.55E-03 | 5.20E-29 | 265753 | 0.047% | 124.96 |
| Ankle spacing width | rs2074027 | 17 | 67502138 | A | G | 5.83E-01 | 1.50E-02 | 2.30E-03 | 7.40E-11 | 265753 | 0.016% | 42.40 |
| Ankle spacing width | rs10401092 | 18 | 2782181 | T | C | 2.25E-01 | -1.52E-02 | 2.72E-03 | 2.30E-08 | 265753 | 0.012% | 31.18 |
| Ankle spacing width | rs55854145 | 18 | 45928049 | C | A | 5.45E-02 | -3.75E-02 | 5.02E-03 | 7.50E-14 | 265753 | 0.021% | 55.93 |
| Ankle spacing width | rs2046136 | 18 | 60813981 | G | A | 3.04E-01 | 1.95E-02 | 2.46E-03 | 2.30E-15 | 265753 | 0.024% | 62.78 |
| Ankle spacing width | rs12953563 | 18 | 22716990 | T | C | 4.20E-01 | 1.51E-02 | 2.30E-03 | 5.50E-11 | 265753 | 0.016% | 43.00 |
| Ankle spacing width | rs7235010 | 18 | 20724810 | A | G | 7.85E-01 | 3.23E-02 | 2.75E-03 | 8.60E-32 | 265753 | 0.052% | 137.67 |
| Ankle spacing width | rs1288796 | 18 | 46596847 | A | G | 8.74E-01 | 1.95E-02 | 3.42E-03 | 1.20E-08 | 265753 | 0.012% | 32.43 |
| Ankle spacing width | rs6567160 | 18 | 57829135 | C | T | 2.33E-01 | 4.20E-02 | 2.68E-03 | 3.50E-55 | 265753 | 0.092% | 244.82 |
| Ankle spacing width | rs111859507 | 18 | 13084980 | G | A | 9.92E-02 | -2.63E-02 | 3.80E-03 | 4.90E-12 | 265753 | 0.018% | 47.71 |
| Ankle spacing width | rs11660019 | 18 | 42883857 | T | C | 1.22E-01 | -2.36E-02 | 3.47E-03 | 1.10E-11 | 265753 | 0.017% | 46.21 |
| Ankle spacing width | rs7229491 | 18 | 46516424 | C | G | 6.61E-01 | -3.94E-02 | 2.40E-03 | 9.00E-61 | 265753 | 0.102% | 270.46 |
| Ankle spacing width | rs17066856 | 18 | 58049656 | C | T | 9.17E-02 | -2.97E-02 | 3.94E-03 | 4.40E-14 | 265753 | 0.021% | 56.98 |
| Ankle spacing width | rs11670606 | 19 | 6096400 | C | T | 2.50E-01 | -1.46E-02 | 2.62E-03 | 2.70E-08 | 265753 | 0.012% | 30.94 |
| Ankle spacing width | rs2163832 | 19 | 10745764 | C | T | 6.60E-01 | -1.83E-02 | 2.40E-03 | 2.20E-14 | 265753 | 0.022% | 58.37 |
| Ankle spacing width | rs78869060 | 19 | 4444742 | G | A | 2.75E-01 | 1.69E-02 | 2.54E-03 | 2.80E-11 | 265753 | 0.017% | 44.33 |
| Ankle spacing width | rs4808844 | 19 | 18811966 | G | A | 3.99E-01 | -1.49E-02 | 2.32E-03 | 1.40E-10 | 265753 | 0.015% | 41.12 |
| Ankle spacing width | rs11672697 | 19 | 33876955 | T | C | 5.05E-01 | 1.94E-02 | 2.27E-03 | 1.40E-17 | 265753 | 0.027% | 72.85 |
| Ankle spacing width | rs11671304 | 19 | 47564643 | T | C | 6.70E-01 | 1.73E-02 | 2.42E-03 | 8.90E-13 | 265753 | 0.019% | 51.07 |
| Ankle spacing width | rs3826851 | 19 | 46974233 | A | G | 2.50E-01 | 1.46E-02 | 2.61E-03 | 2.50E-08 | 265753 | 0.012% | 31.10 |
| Ankle spacing width | rs57753335 | 19 | 47298001 | C | T | 2.27E-01 | -1.59E-02 | 2.74E-03 | 7.30E-09 | 265753 | 0.013% | 33.46 |
| Ankle spacing width | rs67625472 | 19 | 4968620 | C | T | 2.76E-01 | -2.77E-02 | 2.54E-03 | 7.90E-28 | 265753 | 0.045% | 119.56 |
| Ankle spacing width | rs2042919 | 19 | 7963949 | G | A | 2.68E-01 | -1.52E-02 | 2.56E-03 | 3.10E-09 | 265753 | 0.013% | 35.12 |
| Ankle spacing width | rs73004967 | 19 | 19717056 | G | A | 6.86E-02 | -2.48E-02 | 4.49E-03 | 3.40E-08 | 265753 | 0.011% | 30.45 |
| Ankle spacing width | rs6117854 | 20 | 7551554 | A | G | 3.26E-01 | 1.97E-02 | 2.44E-03 | 5.80E-16 | 265753 | 0.025% | 65.52 |
| Ankle spacing width | rs55990870 | 20 | 32302908 | T | C | 2.63E-01 | -3.02E-02 | 2.57E-03 | 6.90E-32 | 265753 | 0.052% | 138.10 |
| Ankle spacing width | rs143384 | 20 | 34025756 | G | A | 4.04E-01 | 2.03E-02 | 2.31E-03 | 1.20E-18 | 265753 | 0.029% | 77.65 |
| Ankle spacing width | rs1161237 | 20 | 2863980 | G | A | 5.05E-01 | -1.38E-02 | 2.27E-03 | 1.10E-09 | 265753 | 0.014% | 37.05 |
| Ankle spacing width | rs1411297 | 20 | 6488806 | C | A | 6.54E-01 | -1.35E-02 | 2.39E-03 | 1.60E-08 | 265753 | 0.012% | 31.96 |
| Ankle spacing width | rs1007708 | 20 | 25190959 | T | C | 4.82E-01 | 1.46E-02 | 2.27E-03 | 1.30E-10 | 265753 | 0.016% | 41.25 |
| Ankle spacing width | rs6130929 | 20 | 44405013 | A | G | 6.56E-01 | 1.32E-02 | 2.38E-03 | 3.30E-08 | 265753 | 0.011% | 30.51 |
| Ankle spacing width | rs1007330 | 20 | 45526121 | T | C | 3.83E-01 | 2.01E-02 | 2.33E-03 | 6.80E-18 | 265753 | 0.028% | 74.28 |
| Ankle spacing width | rs6091546 | 20 | 51104220 | T | C | 1.84E-01 | -2.39E-02 | 2.93E-03 | 3.20E-16 | 265753 | 0.025% | 66.65 |
| Ankle spacing width | rs6063079 | 20 | 45804275 | C | G | 4.88E-01 | 1.75E-02 | 2.28E-03 | 1.80E-14 | 265753 | 0.022% | 58.69 |
| Ankle spacing width | rs6134830 | 20 | 13225350 | G | A | 2.20E-01 | 1.53E-02 | 2.74E-03 | 2.20E-08 | 265753 | 0.012% | 31.35 |
| Ankle spacing width | rs41311445 | 22 | 42070374 | C | A | 9.55E-02 | -3.08E-02 | 3.86E-03 | 1.70E-15 | 265753 | 0.024% | 63.40 |
| Ankle spacing width | rs59663694 | 22 | 30355493 | A | G | 3.87E-01 | -1.61E-02 | 2.33E-03 | 4.10E-12 | 265753 | 0.018% | 48.09 |
| Ankle spacing width | rs28415976 | 22 | 50730220 | G | A | 3.16E-01 | 1.42E-02 | 2.44E-03 | 5.70E-09 | 265753 | 0.013% | 33.94 |
| Ankle spacing width | rs1569414 | 22 | 45727565 | G | T | 2.61E-01 | 3.75E-02 | 2.57E-03 | 3.80E-48 | 265753 | 0.080% | 212.54 |
| Ankle spacing width | rs73149918 | 22 | 22077631 | A | G | 1.71E-01 | 1.94E-02 | 3.02E-03 | 1.40E-10 | 265753 | 0.015% | 41.13 |
| Ankle spacing width | rs6001817 | 22 | 40561759 | T | G | 5.98E-01 | -1.72E-02 | 2.31E-03 | 1.10E-13 | 265753 | 0.021% | 55.13 |
| Ankle spacing width (right) | rs1044299 | 1 | 176811873 | T | C | 5.49E-01 | 2.72E-02 | 3.14E-03 | 4.90E-18 | 146181 | 0.051% | 74.92 |
| Ankle spacing width (right) | rs4845852 | 1 | 11233559 | T | C | 7.39E-01 | -3.14E-02 | 3.54E-03 | 8.10E-19 | 146181 | 0.054% | 78.47 |
| Ankle spacing width (right) | rs74080008 | 1 | 51047717 | T | G | 2.81E-01 | -2.65E-02 | 3.47E-03 | 1.90E-14 | 146181 | 0.040% | 58.61 |
| Ankle spacing width (right) | rs12033524 | 1 | 119454212 | C | T | 2.06E-01 | 2.97E-02 | 3.87E-03 | 1.80E-14 | 146181 | 0.040% | 58.77 |
| Ankle spacing width (right) | rs2666826 | 1 | 155658085 | C | T | 2.84E-01 | -2.04E-02 | 3.46E-03 | 4.10E-09 | 146181 | 0.024% | 34.60 |
| Ankle spacing width (right) | rs8030 | 1 | 177897975 | T | C | 2.97E-01 | 2.14E-02 | 3.41E-03 | 3.80E-10 | 146181 | 0.027% | 39.21 |
| Ankle spacing width (right) | rs6667764 | 1 | 215281223 | G | C | 6.74E-01 | -2.44E-02 | 3.33E-03 | 2.30E-13 | 146181 | 0.037% | 53.70 |
| Ankle spacing width (right) | rs6682438 | 1 | 33784146 | C | T | 6.71E-01 | 1.97E-02 | 3.31E-03 | 2.50E-09 | 146181 | 0.024% | 35.56 |
| Ankle spacing width (right) | rs2154319 | 1 | 41745770 | C | T | 2.17E-01 | 2.97E-02 | 3.81E-03 | 7.50E-15 | 146181 | 0.041% | 60.47 |
| Ankle spacing width (right) | rs1465483 | 1 | 75195243 | C | T | 6.86E-01 | 2.92E-02 | 3.36E-03 | 3.60E-18 | 146181 | 0.052% | 75.51 |
| Ankle spacing width (right) | rs2147324 | 1 | 150255587 | C | T | 5.25E-01 | -1.77E-02 | 3.13E-03 | 1.60E-08 | 146181 | 0.022% | 31.96 |
| Ankle spacing width (right) | rs72744814 | 1 | 201884683 | G | C | 7.76E-02 | -3.51E-02 | 5.84E-03 | 1.80E-09 | 146181 | 0.025% | 36.15 |
| Ankle spacing width (right) | rs7550419 | 1 | 214681969 | A | G | 7.03E-02 | -3.49E-02 | 6.13E-03 | 1.20E-08 | 146181 | 0.022% | 32.46 |
| Ankle spacing width (right) | rs11118684 | 1 | 221473284 | G | A | 4.26E-01 | 2.08E-02 | 3.18E-03 | 6.70E-11 | 146181 | 0.029% | 42.61 |
| Ankle spacing width (right) | rs11164632 | 1 | 103353900 | T | A | 6.07E-01 | -3.17E-02 | 3.20E-03 | 4.70E-23 | 146181 | 0.067% | 97.75 |
| Ankle spacing width (right) | rs4832605 | 2 | 18722424 | C | T | 5.79E-01 | -3.66E-02 | 3.16E-03 | 4.50E-31 | 146181 | 0.092% | 134.37 |
| Ankle spacing width (right) | rs7594766 | 2 | 42228804 | T | C | 2.33E-01 | -3.50E-02 | 3.70E-03 | 2.80E-21 | 146181 | 0.061% | 89.69 |
| Ankle spacing width (right) | rs2140240 | 2 | 226988137 | A | G | 2.74E-01 | 3.17E-02 | 3.67E-03 | 6.20E-18 | 146181 | 0.051% | 74.46 |
| Ankle spacing width (right) | rs66906321 | 2 | 630070 | C | T | 8.19E-01 | 4.04E-02 | 4.12E-03 | 9.70E-23 | 146181 | 0.066% | 96.33 |
| Ankle spacing width (right) | rs2722599 | 2 | 5896921 | C | G | 6.54E-01 | 1.92E-02 | 3.31E-03 | 5.80E-09 | 146181 | 0.023% | 33.89 |
| Ankle spacing width (right) | rs1454379 | 2 | 33440045 | A | G | 5.19E-01 | -2.16E-02 | 3.14E-03 | 6.20E-12 | 146181 | 0.032% | 47.26 |
| Ankle spacing width (right) | rs12998298 | 2 | 66331826 | G | A | 9.14E-01 | -3.48E-02 | 5.67E-03 | 8.20E-10 | 146181 | 0.026% | 37.71 |
| Ankle spacing width (right) | rs10183783 | 2 | 145924502 | T | G | 4.48E-02 | -6.21E-02 | 7.54E-03 | 1.60E-16 | 146181 | 0.047% | 68.03 |
| Ankle spacing width (right) | rs12613291 | 2 | 200858121 | A | C | 5.48E-01 | 1.82E-02 | 3.14E-03 | 6.80E-09 | 146181 | 0.023% | 33.60 |
| Ankle spacing width (right) | rs2618152 | 2 | 218312598 | C | G | 7.25E-01 | 2.08E-02 | 3.51E-03 | 2.80E-09 | 146181 | 0.024% | 35.30 |
| Ankle spacing width (right) | rs1522811 | 2 | 226992454 | A | C | 2.25E-01 | 3.10E-02 | 3.73E-03 | 1.10E-16 | 146181 | 0.047% | 68.83 |
| Ankle spacing width (right) | rs77165542 | 2 | 430975 | T | C | 3.53E-02 | -5.90E-02 | 8.54E-03 | 5.00E-12 | 146181 | 0.033% | 47.69 |
| Ankle spacing width (right) | rs12713404 | 2 | 60006705 | T | G | 6.14E-01 | 2.77E-02 | 3.24E-03 | 1.30E-17 | 146181 | 0.050% | 73.06 |
| Ankle spacing width (right) | rs10490585 | 2 | 66081374 | T | C | 9.93E-02 | -3.08E-02 | 5.27E-03 | 4.80E-09 | 146181 | 0.023% | 34.27 |
| Ankle spacing width (right) | rs10185019 | 2 | 72329685 | T | C | 3.43E-01 | -2.22E-02 | 3.30E-03 | 1.60E-11 | 146181 | 0.031% | 45.36 |
| Ankle spacing width (right) | rs11677976 | 2 | 112468218 | C | T | 4.09E-01 | -1.97E-02 | 3.17E-03 | 5.90E-10 | 146181 | 0.026% | 38.36 |
| Ankle spacing width (right) | rs847150 | 2 | 176968223 | A | G | 2.03E-01 | 2.17E-02 | 3.88E-03 | 2.40E-08 | 146181 | 0.021% | 31.15 |
| Ankle spacing width (right) | rs78292764 | 2 | 40687004 | G | C | 1.83E-01 | 2.33E-02 | 4.04E-03 | 7.50E-09 | 146181 | 0.023% | 33.39 |
| Ankle spacing width (right) | rs4075019 | 2 | 239949666 | C | A | 2.11E-01 | -2.43E-02 | 3.82E-03 | 2.00E-10 | 146181 | 0.028% | 40.50 |
| Ankle spacing width (right) | rs2270894 | 3 | 9975386 | G | C | 2.04E-01 | -3.46E-02 | 4.02E-03 | 6.80E-18 | 146181 | 0.051% | 74.28 |
| Ankle spacing width (right) | rs1466835 | 3 | 12579944 | T | G | 3.91E-01 | -1.86E-02 | 3.19E-03 | 6.10E-09 | 146181 | 0.023% | 33.80 |
| Ankle spacing width (right) | rs150672351 | 3 | 64972526 | A | G | 6.68E-02 | 3.52E-02 | 6.26E-03 | 1.80E-08 | 146181 | 0.022% | 31.74 |
| Ankle spacing width (right) | rs1040319 | 3 | 88098062 | G | T | 8.36E-01 | 2.49E-02 | 4.21E-03 | 3.40E-09 | 146181 | 0.024% | 34.94 |
| Ankle spacing width (right) | rs4402960 | 3 | 185511687 | T | G | 3.13E-01 | -3.40E-02 | 3.36E-03 | 4.40E-24 | 146181 | 0.070% | 102.46 |
| Ankle spacing width (right) | rs6787924 | 3 | 55387030 | T | C | 7.30E-02 | -3.32E-02 | 6.00E-03 | 3.00E-08 | 146181 | 0.021% | 30.69 |
| Ankle spacing width (right) | rs724016 | 3 | 141105570 | G | A | 4.46E-01 | 4.10E-02 | 3.14E-03 | 5.20E-39 | 146181 | 0.117% | 170.69 |
| Ankle spacing width (right) | rs6773963 | 3 | 11511975 | A | G | 6.18E-01 | -2.21E-02 | 3.23E-03 | 8.20E-12 | 146181 | 0.032% | 46.71 |
| Ankle spacing width (right) | rs17275273 | 3 | 64944311 | C | T | 1.28E-01 | 6.50E-02 | 4.71E-03 | 2.60E-43 | 146181 | 0.130% | 190.36 |
| Ankle spacing width (right) | rs2353939 | 4 | 145729724 | G | A | 4.36E-01 | 2.35E-02 | 3.16E-03 | 1.00E-13 | 146181 | 0.038% | 55.33 |
| Ankle spacing width (right) | rs17082654 | 4 | 54243141 | G | A | 1.49E-01 | -2.44E-02 | 4.39E-03 | 2.90E-08 | 146181 | 0.021% | 30.77 |
| Ankle spacing width (right) | rs12509014 | 4 | 123825919 | T | C | 7.89E-01 | -3.59E-02 | 3.82E-03 | 4.60E-21 | 146181 | 0.061% | 88.68 |
| Ankle spacing width (right) | rs1396193 | 4 | 175178914 | C | T | 1.82E-01 | -3.23E-02 | 4.06E-03 | 1.80E-15 | 146181 | 0.043% | 63.24 |
| Ankle spacing width (right) | rs7671110 | 4 | 17874089 | T | C | 1.58E-01 | -3.54E-02 | 4.27E-03 | 1.00E-16 | 146181 | 0.047% | 68.97 |
| Ankle spacing width (right) | rs61730641 | 4 | 87730980 | T | C | 1.65E-02 | -6.73E-02 | 1.23E-02 | 3.90E-08 | 146181 | 0.021% | 30.18 |
| Ankle spacing width (right) | rs17378658 | 4 | 95390157 | A | G | 2.10E-01 | 2.11E-02 | 3.85E-03 | 4.40E-08 | 146181 | 0.020% | 29.96 |
| Ankle spacing width (right) | rs13103161 | 4 | 106216459 | A | T | 3.87E-01 | -2.21E-02 | 3.21E-03 | 6.10E-12 | 146181 | 0.032% | 47.28 |
| Ankle spacing width (right) | rs72678990 | 4 | 124070923 | T | G | 3.94E-02 | -5.68E-02 | 8.03E-03 | 1.60E-12 | 146181 | 0.034% | 49.91 |
| Ankle spacing width (right) | rs56197583 | 5 | 173336792 | A | G | 3.02E-01 | -3.55E-02 | 3.41E-03 | 1.70E-25 | 146181 | 0.074% | 108.91 |
| Ankle spacing width (right) | rs30351 | 5 | 55794632 | A | G | 7.44E-01 | 2.11E-02 | 3.59E-03 | 4.30E-09 | 146181 | 0.024% | 34.48 |
| Ankle spacing width (right) | rs247008 | 5 | 131447104 | G | A | 6.70E-01 | 1.92E-02 | 3.34E-03 | 9.70E-09 | 146181 | 0.023% | 32.90 |
| Ankle spacing width (right) | rs31211 | 5 | 134363145 | A | G | 2.51E-01 | -5.06E-02 | 3.61E-03 | 1.30E-44 | 146181 | 0.134% | 196.43 |
| Ankle spacing width (right) | rs3822742 | 5 | 139059017 | A | C | 3.72E-01 | 2.39E-02 | 3.24E-03 | 1.50E-13 | 146181 | 0.037% | 54.54 |
| Ankle spacing width (right) | rs7701443 | 5 | 142792650 | G | A | 4.09E-01 | -1.79E-02 | 3.17E-03 | 1.70E-08 | 146181 | 0.022% | 31.82 |
| Ankle spacing width (right) | rs6882235 | 5 | 50260139 | C | T | 4.15E-01 | 2.99E-02 | 3.19E-03 | 6.80E-21 | 146181 | 0.060% | 87.93 |
| Ankle spacing width (right) | rs13355315 | 5 | 111225001 | A | C | 6.68E-01 | -1.90E-02 | 3.32E-03 | 1.00E-08 | 146181 | 0.022% | 32.78 |
| Ankle spacing width (right) | rs1582931 | 5 | 122657199 | A | G | 4.72E-01 | -2.05E-02 | 3.16E-03 | 7.60E-11 | 146181 | 0.029% | 42.36 |
| Ankle spacing width (right) | rs13189570 | 5 | 158182309 | T | C | 2.41E-01 | -2.64E-02 | 3.67E-03 | 5.70E-13 | 146181 | 0.036% | 51.94 |
| Ankle spacing width (right) | rs13189275 | 5 | 42521327 | C | A | 4.66E-01 | -2.55E-02 | 3.12E-03 | 3.00E-16 | 146181 | 0.046% | 66.83 |
| Ankle spacing width (right) | rs4364342 | 5 | 51205024 | C | T | 8.49E-01 | 2.99E-02 | 4.39E-03 | 1.00E-11 | 146181 | 0.032% | 46.29 |
| Ankle spacing width (right) | rs34361 | 5 | 74971846 | G | A | 6.53E-01 | 2.14E-02 | 3.29E-03 | 8.10E-11 | 146181 | 0.029% | 42.24 |
| Ankle spacing width (right) | rs7738895 | 6 | 19968313 | T | C | 2.69E-01 | -2.48E-02 | 3.52E-03 | 1.60E-12 | 146181 | 0.034% | 49.96 |
| Ankle spacing width (right) | rs2635727 | 6 | 50820940 | C | T | 7.54E-01 | 2.05E-02 | 3.61E-03 | 1.50E-08 | 146181 | 0.022% | 32.10 |
| Ankle spacing width (right) | rs1502201 | 6 | 55265497 | A | G | 7.45E-01 | 3.25E-02 | 3.57E-03 | 9.60E-20 | 146181 | 0.057% | 82.69 |
| Ankle spacing width (right) | rs509453 | 6 | 133796444 | C | T | 7.75E-01 | -2.24E-02 | 3.74E-03 | 2.20E-09 | 146181 | 0.024% | 35.79 |
| Ankle spacing width (right) | rs3129962 | 6 | 32379383 | A | G | 1.27E-01 | 3.32E-02 | 4.67E-03 | 1.20E-12 | 146181 | 0.035% | 50.48 |
| Ankle spacing width (right) | rs62405901 | 6 | 33475542 | A | G | 8.92E-02 | 3.24E-02 | 5.47E-03 | 3.10E-09 | 146181 | 0.024% | 35.10 |
| Ankle spacing width (right) | rs12189907 | 6 | 85369497 | C | T | 4.29E-01 | -3.04E-02 | 3.15E-03 | 4.60E-22 | 146181 | 0.064% | 93.27 |
| Ankle spacing width (right) | rs7740107 | 6 | 130374461 | A | T | 7.37E-01 | -4.39E-02 | 3.54E-03 | 2.10E-35 | 146181 | 0.105% | 154.21 |
| Ankle spacing width (right) | rs2296131 | 6 | 1872339 | C | T | 6.21E-01 | -1.77E-02 | 3.23E-03 | 4.30E-08 | 146181 | 0.021% | 29.99 |
| Ankle spacing width (right) | rs114056237 | 6 | 41877671 | A | G | 1.24E-02 | -8.22E-02 | 1.40E-02 | 4.90E-09 | 146181 | 0.023% | 34.24 |
| Ankle spacing width (right) | rs9352895 | 6 | 81590856 | A | T | 4.21E-01 | -2.14E-02 | 3.16E-03 | 1.20E-11 | 146181 | 0.031% | 46.02 |
| Ankle spacing width (right) | rs4946935 | 6 | 109000742 | G | A | 7.14E-01 | 2.27E-02 | 3.48E-03 | 6.90E-11 | 146181 | 0.029% | 42.56 |
| Ankle spacing width (right) | rs9379832 | 6 | 26186200 | G | A | 2.55E-01 | -2.12E-02 | 3.61E-03 | 3.90E-09 | 146181 | 0.024% | 34.69 |
| Ankle spacing width (right) | rs79623207 | 6 | 34383659 | C | T | 1.93E-02 | 8.96E-02 | 1.13E-02 | 2.50E-15 | 146181 | 0.043% | 62.65 |
| Ankle spacing width (right) | rs511490 | 6 | 133568151 | C | T | 7.47E-01 | 3.10E-02 | 3.60E-03 | 6.80E-18 | 146181 | 0.051% | 74.28 |
| Ankle spacing width (right) | rs112202910 | 6 | 1388229 | C | G | 2.58E-01 | 2.59E-02 | 3.55E-03 | 3.30E-13 | 146181 | 0.036% | 53.05 |
| Ankle spacing width (right) | rs2178899 | 6 | 31606756 | T | A | 1.29E-01 | -4.25E-02 | 4.63E-03 | 4.90E-20 | 146181 | 0.057% | 84.02 |
| Ankle spacing width (right) | rs9267922 | 6 | 32206465 | A | G | 2.25E-01 | 2.19E-02 | 3.83E-03 | 1.10E-08 | 146181 | 0.022% | 32.57 |
| Ankle spacing width (right) | rs79451598 | 6 | 55635692 | C | T | 6.74E-02 | -7.53E-02 | 6.23E-03 | 1.40E-33 | 146181 | 0.100% | 145.86 |
| Ankle spacing width (right) | rs150445982 | 6 | 55676621 | T | C | 2.27E-02 | -7.44E-02 | 1.10E-02 | 1.20E-11 | 146181 | 0.031% | 45.91 |
| Ankle spacing width (right) | rs4709746 | 6 | 164133001 | T | C | 1.35E-01 | 3.42E-02 | 4.60E-03 | 1.00E-13 | 146181 | 0.038% | 55.30 |
| Ankle spacing width (right) | rs2080246 | 7 | 27569704 | A | G | 6.92E-01 | -6.11E-02 | 3.39E-03 | 1.20E-72 | 146181 | 0.222% | 324.98 |
| Ankle spacing width (right) | rs7784776 | 7 | 46620145 | G | A | 3.90E-01 | 2.03E-02 | 3.20E-03 | 2.30E-10 | 146181 | 0.027% | 40.18 |
| Ankle spacing width (right) | rs960273 | 7 | 2857876 | C | T | 2.95E-01 | -2.32E-02 | 3.42E-03 | 1.30E-11 | 146181 | 0.031% | 45.75 |
| Ankle spacing width (right) | rs56376645 | 7 | 25886125 | A | G | 2.47E-01 | 3.85E-02 | 3.63E-03 | 2.80E-26 | 146181 | 0.077% | 112.51 |
| Ankle spacing width (right) | rs35525401 | 7 | 74402206 | T | G | 2.06E-01 | 2.79E-02 | 3.98E-03 | 2.40E-12 | 146181 | 0.034% | 49.16 |
| Ankle spacing width (right) | rs2732749 | 7 | 84825607 | G | A | 2.37E-01 | -2.02E-02 | 3.67E-03 | 3.90E-08 | 146181 | 0.021% | 30.22 |
| Ankle spacing width (right) | rs2215169 | 7 | 15956470 | C | T | 6.63E-01 | 2.26E-02 | 3.30E-03 | 7.50E-12 | 146181 | 0.032% | 46.90 |
| Ankle spacing width (right) | rs2429215 | 7 | 139985962 | A | G | 3.27E-01 | -1.84E-02 | 3.35E-03 | 4.20E-08 | 146181 | 0.021% | 30.08 |
| Ankle spacing width (right) | rs17777642 | 7 | 104599208 | A | G | 3.92E-01 | 2.03E-02 | 3.20E-03 | 2.50E-10 | 146181 | 0.027% | 40.04 |
| Ankle spacing width (right) | rs62621812 | 7 | 127015083 | A | G | 2.05E-02 | 1.31E-01 | 1.13E-02 | 8.50E-31 | 146181 | 0.091% | 133.13 |
| Ankle spacing width (right) | rs9770544 | 7 | 27232126 | G | C | 8.21E-01 | 5.08E-02 | 4.11E-03 | 4.20E-35 | 146181 | 0.104% | 152.81 |
| Ankle spacing width (right) | rs10228450 | 7 | 27498420 | C | T | 5.13E-01 | -2.14E-02 | 3.13E-03 | 9.30E-12 | 146181 | 0.032% | 46.47 |
| Ankle spacing width (right) | rs2598109 | 7 | 37971913 | T | C | 8.40E-01 | 2.41E-02 | 4.25E-03 | 1.30E-08 | 146181 | 0.022% | 32.29 |
| Ankle spacing width (right) | rs2282978 | 7 | 92264410 | C | T | 3.27E-01 | 4.09E-02 | 3.32E-03 | 8.40E-35 | 146181 | 0.103% | 151.44 |
| Ankle spacing width (right) | rs10263705 | 7 | 130027633 | G | A | 3.47E-01 | 2.31E-02 | 3.28E-03 | 1.70E-12 | 146181 | 0.034% | 49.85 |
| Ankle spacing width (right) | rs2942202 | 8 | 23418444 | C | A | 4.95E-01 | -1.99E-02 | 3.14E-03 | 2.30E-10 | 146181 | 0.027% | 40.17 |
| Ankle spacing width (right) | rs72639077 | 8 | 49527235 | G | T | 9.90E-02 | 3.41E-02 | 5.25E-03 | 8.40E-11 | 146181 | 0.029% | 42.17 |
| Ankle spacing width (right) | rs1385236 | 8 | 96673521 | G | T | 2.26E-01 | -3.64E-02 | 3.75E-03 | 2.70E-22 | 146181 | 0.064% | 94.34 |
| Ankle spacing width (right) | rs13272487 | 8 | 117478905 | A | T | 2.17E-01 | -2.11E-02 | 3.82E-03 | 3.40E-08 | 146181 | 0.021% | 30.46 |
| Ankle spacing width (right) | rs7007568 | 8 | 128364906 | C | G | 4.22E-01 | -1.97E-02 | 3.16E-03 | 4.50E-10 | 146181 | 0.027% | 38.89 |
| Ankle spacing width (right) | rs13267329 | 8 | 135735110 | A | G | 1.54E-01 | 2.59E-02 | 4.34E-03 | 2.30E-09 | 146181 | 0.024% | 35.69 |
| Ankle spacing width (right) | rs72656010 | 8 | 57122215 | C | T | 1.33E-01 | -3.61E-02 | 4.63E-03 | 6.60E-15 | 146181 | 0.042% | 60.70 |
| Ankle spacing width (right) | rs28420312 | 8 | 74560577 | A | G | 2.07E-01 | 2.61E-02 | 3.85E-03 | 1.30E-11 | 146181 | 0.031% | 45.79 |
| Ankle spacing width (right) | rs2721938 | 8 | 116635611 | T | C | 6.02E-01 | -2.11E-02 | 3.19E-03 | 4.10E-11 | 146181 | 0.030% | 43.58 |
| Ankle spacing width (right) | rs12541381 | 8 | 135649848 | A | G | 2.57E-01 | -2.96E-02 | 3.57E-03 | 1.30E-16 | 146181 | 0.047% | 68.49 |
| Ankle spacing width (right) | rs1134030 | 8 | 145051484 | T | C | 3.70E-01 | -2.41E-02 | 3.26E-03 | 1.50E-13 | 146181 | 0.037% | 54.53 |
| Ankle spacing width (right) | rs7832976 | 8 | 10065961 | G | A | 1.77E-01 | 2.27E-02 | 4.13E-03 | 4.00E-08 | 146181 | 0.021% | 30.15 |
| Ankle spacing width (right) | rs10112467 | 8 | 72460614 | A | G | 7.37E-02 | -4.75E-02 | 6.01E-03 | 2.60E-15 | 146181 | 0.043% | 62.56 |
| Ankle spacing width (right) | rs7819550 | 8 | 74206582 | A | G | 1.94E-01 | 2.17E-02 | 3.96E-03 | 3.90E-08 | 146181 | 0.021% | 30.20 |
| Ankle spacing width (right) | rs913277 | 9 | 96910136 | T | C | 7.03E-01 | -2.13E-02 | 3.44E-03 | 6.10E-10 | 146181 | 0.026% | 38.29 |
| Ankle spacing width (right) | rs10756486 | 9 | 13591185 | T | C | 7.63E-01 | -2.02E-02 | 3.68E-03 | 4.30E-08 | 146181 | 0.021% | 30.00 |
| Ankle spacing width (right) | rs35307904 | 9 | 78511889 | A | G | 1.23E-01 | -2.88E-02 | 4.78E-03 | 1.70E-09 | 146181 | 0.025% | 36.33 |
| Ankle spacing width (right) | rs2772033 | 9 | 133757080 | A | G | 1.11E-01 | -2.80E-02 | 5.00E-03 | 2.20E-08 | 146181 | 0.021% | 31.32 |
| Ankle spacing width (right) | rs28504650 | 9 | 98257842 | T | C | 3.54E-01 | 2.24E-02 | 3.28E-03 | 7.80E-12 | 146181 | 0.032% | 46.82 |
| Ankle spacing width (right) | rs12344772 | 9 | 118490315 | T | G | 8.39E-02 | -4.33E-02 | 5.67E-03 | 2.20E-14 | 146181 | 0.040% | 58.33 |
| Ankle spacing width (right) | rs3761849 | 9 | 123690957 | T | C | 6.20E-01 | 2.01E-02 | 3.24E-03 | 5.60E-10 | 146181 | 0.026% | 38.47 |
| Ankle spacing width (right) | rs115850137 | 9 | 139619958 | A | G | 1.26E-01 | -2.86E-02 | 4.73E-03 | 1.40E-09 | 146181 | 0.025% | 36.63 |
| Ankle spacing width (right) | rs13291277 | 9 | 34630812 | A | T | 3.43E-01 | 1.94E-02 | 3.31E-03 | 4.30E-09 | 146181 | 0.024% | 34.48 |
| Ankle spacing width (right) | rs2795054 | 9 | 112544702 | T | A | 7.50E-01 | -2.02E-02 | 3.64E-03 | 3.00E-08 | 146181 | 0.021% | 30.72 |
| Ankle spacing width (right) | rs4749368 | 10 | 29228259 | C | A | 5.23E-01 | 2.52E-02 | 3.15E-03 | 1.40E-15 | 146181 | 0.044% | 63.75 |
| Ankle spacing width (right) | rs12761779 | 10 | 63782043 | G | C | 3.50E-01 | -1.88E-02 | 3.32E-03 | 1.50E-08 | 146181 | 0.022% | 32.03 |
| Ankle spacing width (right) | rs4746475 | 10 | 64358685 | T | C | 1.65E-01 | -2.52E-02 | 4.24E-03 | 2.80E-09 | 146181 | 0.024% | 35.30 |
| Ankle spacing width (right) | rs7918664 | 10 | 28819640 | C | G | 2.78E-01 | -2.06E-02 | 3.49E-03 | 3.40E-09 | 146181 | 0.024% | 34.94 |
| Ankle spacing width (right) | rs80123520 | 10 | 124136529 | A | G | 7.04E-02 | -3.54E-02 | 6.10E-03 | 6.50E-09 | 146181 | 0.023% | 33.67 |
| Ankle spacing width (right) | rs967186 | 10 | 130705973 | T | G | 7.02E-01 | 2.07E-02 | 3.42E-03 | 1.30E-09 | 146181 | 0.025% | 36.78 |
| Ankle spacing width (right) | rs603424 | 10 | 102075479 | A | G | 1.75E-01 | 7.83E-02 | 4.10E-03 | 2.10E-81 | 146181 | 0.249% | 365.19 |
| Ankle spacing width (right) | rs74606364 | 10 | 104198111 | A | G | 6.88E-02 | -3.62E-02 | 6.20E-03 | 5.20E-09 | 146181 | 0.023% | 34.13 |
| Ankle spacing width (right) | rs11030104 | 11 | 27684517 | G | A | 2.03E-01 | -2.92E-02 | 3.88E-03 | 5.10E-14 | 146181 | 0.039% | 56.69 |
| Ankle spacing width (right) | rs1151523 | 11 | 65665200 | T | C | 5.45E-01 | -2.44E-02 | 3.14E-03 | 7.80E-15 | 146181 | 0.041% | 60.38 |
| Ankle spacing width (right) | rs11234641 | 11 | 86120586 | T | C | 2.32E-01 | -2.08E-02 | 3.70E-03 | 1.90E-08 | 146181 | 0.022% | 31.63 |
| Ankle spacing width (right) | rs73398082 | 11 | 2095273 | T | C | 1.86E-01 | -2.65E-02 | 4.02E-03 | 3.90E-11 | 146181 | 0.030% | 43.64 |
| Ankle spacing width (right) | rs112330217 | 11 | 466966 | C | G | 5.48E-02 | -3.83E-02 | 6.91E-03 | 3.00E-08 | 146181 | 0.021% | 30.68 |
| Ankle spacing width (right) | rs907614 | 11 | 1877533 | C | T | 6.13E-01 | -2.35E-02 | 3.21E-03 | 2.70E-13 | 146181 | 0.037% | 53.39 |
| Ankle spacing width (right) | rs7944706 | 11 | 10331311 | A | G | 4.33E-01 | 1.97E-02 | 3.16E-03 | 4.40E-10 | 146181 | 0.027% | 38.94 |
| Ankle spacing width (right) | rs314760 | 11 | 68117612 | A | C | 3.06E-01 | -2.02E-02 | 3.40E-03 | 3.10E-09 | 146181 | 0.024% | 35.15 |
| Ankle spacing width (right) | rs12273670 | 11 | 69171983 | G | T | 3.99E-01 | 1.76E-02 | 3.21E-03 | 3.90E-08 | 146181 | 0.021% | 30.20 |
| Ankle spacing width (right) | rs57307148 | 12 | 1033183 | A | C | 2.12E-01 | 2.28E-02 | 3.83E-03 | 2.60E-09 | 146181 | 0.024% | 35.47 |
| Ankle spacing width (right) | rs76895963 | 12 | 4384844 | G | T | 2.07E-02 | 1.33E-01 | 1.21E-02 | 3.20E-28 | 146181 | 0.083% | 121.33 |
| Ankle spacing width (right) | rs76944680 | 12 | 65599819 | T | C | 2.13E-02 | 6.87E-02 | 1.13E-02 | 1.30E-09 | 146181 | 0.025% | 36.85 |
| Ankle spacing width (right) | rs11111141 | 12 | 102429501 | C | A | 1.78E-01 | 2.32E-02 | 4.09E-03 | 1.40E-08 | 146181 | 0.022% | 32.21 |
| Ankle spacing width (right) | rs35756741 | 12 | 12868701 | T | C | 9.08E-02 | -3.38E-02 | 5.47E-03 | 6.40E-10 | 146181 | 0.026% | 38.19 |
| Ankle spacing width (right) | rs7959150 | 12 | 26428063 | G | A | 7.34E-01 | -2.55E-02 | 3.53E-03 | 4.90E-13 | 146181 | 0.036% | 52.26 |
| Ankle spacing width (right) | rs7487625 | 12 | 66319996 | C | A | 2.28E-01 | -5.50E-02 | 3.73E-03 | 4.90E-49 | 146181 | 0.148% | 216.65 |
| Ankle spacing width (right) | rs34426931 | 12 | 77615252 | A | C | 4.00E-01 | 2.21E-02 | 3.19E-03 | 4.00E-12 | 146181 | 0.033% | 48.13 |
| Ankle spacing width (right) | rs4758677 | 12 | 122675081 | A | G | 2.69E-01 | 2.02E-02 | 3.56E-03 | 1.40E-08 | 146181 | 0.022% | 32.19 |
| Ankle spacing width (right) | rs11175826 | 12 | 65977830 | T | C | 1.78E-02 | -1.30E-01 | 1.19E-02 | 5.40E-28 | 146181 | 0.082% | 120.32 |
| Ankle spacing width (right) | rs61754164 | 12 | 53684619 | G | A | 3.51E-01 | -1.83E-02 | 3.27E-03 | 2.20E-08 | 146181 | 0.021% | 31.27 |
| Ankle spacing width (right) | rs770083 | 12 | 89776284 | C | T | 4.50E-01 | 1.98E-02 | 3.15E-03 | 3.10E-10 | 146181 | 0.027% | 39.62 |
| Ankle spacing width (right) | rs35551290 | 13 | 50466633 | T | C | 9.99E-02 | 3.09E-02 | 5.31E-03 | 6.20E-09 | 146181 | 0.023% | 33.78 |
| Ankle spacing width (right) | rs2760307 | 13 | 101190329 | C | A | 5.19E-01 | -2.52E-02 | 3.13E-03 | 8.40E-16 | 146181 | 0.044% | 64.78 |
| Ankle spacing width (right) | rs157165 | 13 | 51082944 | T | C | 2.27E-01 | -4.86E-02 | 3.74E-03 | 1.50E-38 | 146181 | 0.115% | 168.60 |
| Ankle spacing width (right) | rs7986552 | 13 | 91919443 | G | A | 4.17E-01 | 2.21E-02 | 3.18E-03 | 3.90E-12 | 146181 | 0.033% | 48.20 |
| Ankle spacing width (right) | rs35052580 | 13 | 30976240 | T | G | 5.16E-02 | 4.30E-02 | 7.15E-03 | 1.90E-09 | 146181 | 0.025% | 36.11 |
| Ankle spacing width (right) | rs10133006 | 14 | 21749473 | G | A | 2.99E-01 | 1.99E-02 | 3.43E-03 | 7.20E-09 | 146181 | 0.023% | 33.49 |
| Ankle spacing width (right) | rs71413981 | 14 | 23774916 | A | G | 1.62E-01 | 2.67E-02 | 4.25E-03 | 3.50E-10 | 146181 | 0.027% | 39.39 |
| Ankle spacing width (right) | rs6572916 | 14 | 54103013 | T | G | 3.36E-01 | -2.61E-02 | 3.34E-03 | 5.90E-15 | 146181 | 0.042% | 60.93 |
| Ankle spacing width (right) | rs8014071 | 14 | 54431500 | G | A | 3.86E-01 | -2.90E-02 | 3.23E-03 | 2.70E-19 | 146181 | 0.055% | 80.68 |
| Ankle spacing width (right) | rs11158820 | 14 | 70347348 | G | A | 6.86E-01 | -1.90E-02 | 3.40E-03 | 2.30E-08 | 146181 | 0.021% | 31.26 |
| Ankle spacing width (right) | rs11844656 | 14 | 91459332 | C | G | 5.28E-01 | 1.99E-02 | 3.13E-03 | 1.90E-10 | 146181 | 0.028% | 40.54 |
| Ankle spacing width (right) | rs1870043 | 14 | 98405049 | A | G | 5.67E-01 | 2.50E-02 | 3.17E-03 | 3.40E-15 | 146181 | 0.042% | 62.03 |
| Ankle spacing width (right) | rs28929474 | 14 | 94844947 | T | C | 2.00E-02 | 8.90E-02 | 1.11E-02 | 1.40E-15 | 146181 | 0.044% | 63.78 |
| Ankle spacing width (right) | rs80182532 | 15 | 68269444 | C | T | 4.31E-02 | -4.57E-02 | 7.78E-03 | 4.30E-09 | 146181 | 0.024% | 34.47 |
| Ankle spacing width (right) | rs28757157 | 15 | 51545401 | T | C | 4.39E-02 | -4.54E-02 | 7.68E-03 | 3.50E-09 | 146181 | 0.024% | 34.91 |
| Ankle spacing width (right) | rs2439387 | 15 | 67024951 | C | T | 7.16E-01 | -2.66E-02 | 3.47E-03 | 1.70E-14 | 146181 | 0.040% | 58.84 |
| Ankle spacing width (right) | rs4778950 | 15 | 82223654 | T | C | 2.24E-01 | -2.23E-02 | 3.75E-03 | 2.90E-09 | 146181 | 0.024% | 35.25 |
| Ankle spacing width (right) | rs2311769 | 15 | 99229793 | C | T | 6.77E-01 | 2.13E-02 | 3.34E-03 | 1.70E-10 | 146181 | 0.028% | 40.74 |
| Ankle spacing width (right) | rs7498665 | 16 | 28883241 | G | A | 4.00E-01 | 2.24E-02 | 3.20E-03 | 2.50E-12 | 146181 | 0.034% | 49.06 |
| Ankle spacing width (right) | rs4788190 | 16 | 29948401 | A | G | 5.49E-01 | 2.02E-02 | 3.14E-03 | 1.20E-10 | 146181 | 0.028% | 41.54 |
| Ankle spacing width (right) | rs1121980 | 16 | 53809247 | A | G | 4.25E-01 | 3.05E-02 | 3.16E-03 | 4.30E-22 | 146181 | 0.064% | 93.39 |
| Ankle spacing width (right) | rs9941221 | 16 | 54258031 | C | T | 8.39E-01 | -3.23E-02 | 4.26E-03 | 3.90E-14 | 146181 | 0.039% | 57.24 |
| Ankle spacing width (right) | rs12443634 | 16 | 81524274 | C | A | 7.14E-01 | -2.02E-02 | 3.49E-03 | 7.40E-09 | 146181 | 0.023% | 33.44 |
| Ankle spacing width (right) | rs12051245 | 16 | 783865 | C | T | 2.31E-01 | 2.06E-02 | 3.72E-03 | 3.30E-08 | 146181 | 0.021% | 30.54 |
| Ankle spacing width (right) | rs111414205 | 16 | 67151243 | T | C | 2.96E-02 | 5.86E-02 | 9.36E-03 | 3.90E-10 | 146181 | 0.027% | 39.15 |
| Ankle spacing width (right) | rs8059189 | 16 | 86417349 | A | G | 4.03E-01 | -1.86E-02 | 3.27E-03 | 1.40E-08 | 146181 | 0.022% | 32.24 |
| Ankle spacing width (right) | rs11076826 | 16 | 4427010 | C | G | 7.45E-01 | 2.07E-02 | 3.60E-03 | 8.60E-09 | 146181 | 0.023% | 33.13 |
| Ankle spacing width (right) | rs523190 | 17 | 14403421 | A | G | 5.03E-01 | 2.04E-02 | 3.14E-03 | 7.30E-11 | 146181 | 0.029% | 42.42 |
| Ankle spacing width (right) | rs62068770 | 17 | 29245375 | G | C | 2.67E-01 | -2.37E-02 | 3.54E-03 | 1.90E-11 | 146181 | 0.031% | 45.06 |
| Ankle spacing width (right) | rs34527992 | 17 | 46659561 | A | C | 1.90E-01 | -2.59E-02 | 4.00E-03 | 9.00E-11 | 146181 | 0.029% | 42.03 |
| Ankle spacing width (right) | rs72815304 | 17 | 2007004 | T | C | 3.78E-01 | -2.03E-02 | 3.23E-03 | 3.20E-10 | 146181 | 0.027% | 39.55 |
| Ankle spacing width (right) | rs8077036 | 17 | 67962340 | G | C | 3.43E-01 | -2.30E-02 | 3.43E-03 | 2.00E-11 | 146181 | 0.031% | 44.95 |
| Ankle spacing width (right) | rs2159035 | 17 | 69121783 | A | G | 5.71E-01 | -2.43E-02 | 3.16E-03 | 1.40E-14 | 146181 | 0.041% | 59.23 |
| Ankle spacing width (right) | rs2040347 | 17 | 61998879 | G | A | 6.65E-01 | 2.45E-02 | 3.33E-03 | 2.10E-13 | 146181 | 0.037% | 53.87 |
| Ankle spacing width (right) | rs35572189 | 17 | 79419025 | A | G | 3.62E-01 | 3.70E-02 | 3.27E-03 | 1.10E-29 | 146181 | 0.088% | 128.04 |
| Ankle spacing width (right) | rs9910180 | 17 | 54247035 | G | A | 1.28E-01 | -3.34E-02 | 4.69E-03 | 1.10E-12 | 146181 | 0.035% | 50.57 |
| Ankle spacing width (right) | rs150746336 | 17 | 79532081 | T | C | 1.90E-02 | 6.80E-02 | 1.20E-02 | 1.40E-08 | 146181 | 0.022% | 32.18 |
| Ankle spacing width (right) | rs9675924 | 18 | 20730712 | G | A | 7.43E-01 | 2.40E-02 | 3.60E-03 | 2.50E-11 | 146181 | 0.030% | 44.56 |
| Ankle spacing width (right) | rs9953366 | 18 | 46474192 | C | T | 6.78E-01 | -3.75E-02 | 3.41E-03 | 3.90E-28 | 146181 | 0.083% | 120.94 |
| Ankle spacing width (right) | rs754093 | 18 | 77246406 | G | T | 4.53E-01 | 1.82E-02 | 3.15E-03 | 7.90E-09 | 146181 | 0.023% | 33.30 |
| Ankle spacing width (right) | rs2276200 | 18 | 46784633 | A | G | 4.66E-02 | -4.15E-02 | 7.44E-03 | 2.40E-08 | 146181 | 0.021% | 31.14 |
| Ankle spacing width (right) | rs6567160 | 18 | 57829135 | C | T | 2.34E-01 | 3.62E-02 | 3.69E-03 | 8.50E-23 | 146181 | 0.066% | 96.60 |
| Ankle spacing width (right) | rs78030362 | 19 | 18575193 | G | A | 7.45E-02 | 3.46E-02 | 5.99E-03 | 7.20E-09 | 146181 | 0.023% | 33.48 |
| Ankle spacing width (right) | rs7255672 | 19 | 13950321 | C | T | 3.04E-01 | 2.04E-02 | 3.44E-03 | 3.30E-09 | 146181 | 0.024% | 34.97 |
| Ankle spacing width (right) | rs10853981 | 19 | 4965064 | A | G | 3.30E-01 | -3.22E-02 | 3.34E-03 | 4.70E-22 | 146181 | 0.064% | 93.23 |
| Ankle spacing width (right) | rs11673527 | 19 | 6126818 | A | G | 6.79E-01 | 1.86E-02 | 3.37E-03 | 3.30E-08 | 146181 | 0.021% | 30.53 |
| Ankle spacing width (right) | rs561059 | 20 | 3011175 | G | A | 4.96E-01 | -1.77E-02 | 3.13E-03 | 1.50E-08 | 146181 | 0.022% | 32.00 |
| Ankle spacing width (right) | rs34031061 | 20 | 7593266 | C | G | 3.02E-01 | 2.30E-02 | 3.41E-03 | 1.70E-11 | 146181 | 0.031% | 45.32 |
| Ankle spacing width (right) | rs143384 | 20 | 34025756 | G | A | 4.07E-01 | 2.38E-02 | 3.18E-03 | 6.80E-14 | 146181 | 0.038% | 56.13 |
| Ankle spacing width (right) | rs67375757 | 20 | 47916295 | C | T | 2.19E-01 | 2.26E-02 | 3.78E-03 | 2.40E-09 | 146181 | 0.024% | 35.61 |
| Ankle spacing width (right) | rs57668191 | 20 | 32289763 | G | A | 2.61E-01 | -3.12E-02 | 3.57E-03 | 2.60E-18 | 146181 | 0.052% | 76.15 |
| Ankle spacing width (right) | rs73622688 | 20 | 45523702 | A | C | 3.76E-01 | 2.16E-02 | 3.24E-03 | 2.90E-11 | 146181 | 0.030% | 44.23 |
| Ankle spacing width (right) | rs4239647 | 20 | 45757543 | G | C | 5.53E-01 | 1.77E-02 | 3.15E-03 | 2.10E-08 | 146181 | 0.022% | 31.44 |
| Ankle spacing width (right) | rs2836631 | 21 | 40065906 | G | T | 4.87E-01 | 1.92E-02 | 3.14E-03 | 1.00E-09 | 146181 | 0.026% | 37.31 |
| Ankle spacing width (right) | rs62240962 | 22 | 42259524 | T | C | 8.39E-02 | -4.26E-02 | 5.65E-03 | 4.70E-14 | 146181 | 0.039% | 56.85 |
| Ankle spacing width (right) | rs6007594 | 22 | 45728370 | A | G | 2.59E-01 | 3.57E-02 | 3.57E-03 | 1.20E-23 | 146181 | 0.069% | 100.42 |
| Ankle spacing width (right) | rs2023682 | 22 | 30599570 | G | A | 2.99E-01 | 2.31E-02 | 3.43E-03 | 1.70E-11 | 146181 | 0.031% | 45.30 |
| Ankle spacing width (right) | rs5750927 | 22 | 40674593 | A | G | 4.00E-01 | 2.25E-02 | 3.20E-03 | 2.10E-12 | 146181 | 0.034% | 49.43 |
| Height | rs1003484 | 11 | 2167618 | G | A | 3.02E-01 | 2.74E-01 | 3.24E-02 | 2.57E-17 | 99997 | 0.071% | 71.46 |
| Height | rs1004759 | 1 | 170776549 | G | A | 1.23E-01 | 2.16E-01 | 3.74E-02 | 7.66E-09 | 99997 | 0.033% | 33.29 |
| Height | rs1005640 | 22 | 20789074 | C | T | 4.61E-01 | 1.53E-01 | 2.69E-02 | 1.51E-08 | 99997 | 0.032% | 32.14 |
| Height | rs1008834 | 17 | 46968008 | C | G | 4.35E-01 | -1.85E-01 | 2.66E-02 | 4.26E-12 | 99997 | 0.048% | 48.16 |
| Height | rs10100216 | 8 | 76005097 | T | C | 4.14E-01 | -1.89E-01 | 2.78E-02 | 9.64E-12 | 99997 | 0.046% | 46.32 |
| Height | rs10232076 | 7 | 98948904 | T | C | 8.45E-02 | 2.92E-01 | 4.92E-02 | 3.00E-09 | 99997 | 0.035% | 35.22 |
| Height | rs10242990 | 7 | 139730500 | G | C | 2.94E-01 | 1.77E-01 | 3.00E-02 | 3.50E-09 | 99997 | 0.035% | 34.89 |
| Height | rs1043515 | 17 | 36922196 | G | A | 4.33E-01 | 1.85E-01 | 2.72E-02 | 9.15E-12 | 99997 | 0.046% | 46.41 |
| Height | rs1046934 | 1 | 184023529 | C | A | 3.27E-01 | 2.24E-01 | 2.77E-02 | 6.42E-16 | 99997 | 0.065% | 65.39 |
| Height | rs10483727 | 14 | 61072875 | C | T | 4.04E-01 | -2.50E-01 | 2.72E-02 | 4.12E-20 | 99997 | 0.084% | 84.27 |
| Height | rs1064048 | 12 | 3392663 | T | C | 6.46E-02 | 3.71E-01 | 5.74E-02 | 1.01E-10 | 99997 | 0.042% | 41.77 |
| Height | rs10748128 | 12 | 69827658 | T | G | 3.53E-01 | 1.81E-01 | 2.83E-02 | 1.46E-10 | 99997 | 0.041% | 41.04 |
| Height | rs10777536 | 12 | 94120732 | A | G | 5.00E-01 | -1.85E-01 | 2.71E-02 | 1.07E-11 | 99997 | 0.046% | 46.35 |
| Height | rs10778161 | 12 | 102367384 | G | A | 2.96E-01 | 2.45E-01 | 2.98E-02 | 2.10E-16 | 99997 | 0.068% | 67.65 |
| Height | rs10793850 | 6 | 7715955 | T | G | 4.59E-01 | 2.97E-01 | 2.66E-02 | 5.95E-29 | 99997 | 0.125% | 124.92 |
| Height | rs10846654 | 12 | 124799458 | A | G | 3.34E-01 | -2.20E-01 | 2.79E-02 | 3.04E-15 | 99997 | 0.062% | 62.35 |
| Height | rs10853650 | 18 | 22718440 | C | G | 3.39E-01 | 1.59E-01 | 2.86E-02 | 2.83E-08 | 99997 | 0.031% | 30.75 |
| Height | rs10903252 | 5 | 179733934 | C | T | 3.44E-01 | 2.32E-01 | 2.71E-02 | 1.14E-17 | 99997 | 0.073% | 73.29 |
| Height | rs10940473 | 5 | 54873285 | T | C | 3.09E-01 | -2.03E-01 | 2.89E-02 | 2.21E-12 | 99997 | 0.049% | 49.19 |
| Height | rs10950949 | 7 | 23519260 | C | A | 3.74E-01 | -2.15E-01 | 2.69E-02 | 1.21E-15 | 99997 | 0.064% | 64.00 |
| Height | rs11057898 | 12 | 125391057 | A | G | 1.72E-01 | -2.09E-01 | 3.58E-02 | 5.39E-09 | 99997 | 0.034% | 33.95 |
| Height | rs11103398 | 9 | 139134474 | C | T | 2.99E-01 | 1.87E-01 | 3.12E-02 | 2.29E-09 | 99997 | 0.036% | 35.73 |
| Height | rs11118356 | 1 | 219777110 | T | C | 4.63E-01 | -2.01E-01 | 2.64E-02 | 2.58E-14 | 99997 | 0.058% | 57.91 |
| Height | rs111227886 | 4 | 1199518 | T | C | 4.37E-02 | -3.67E-01 | 6.67E-02 | 3.72E-08 | 99997 | 0.030% | 30.24 |
| Height | rs111251222 | 5 | 176735612 | G | A | 1.93E-01 | 3.08E-01 | 3.13E-02 | 7.40E-23 | 99997 | 0.097% | 96.95 |
| Height | rs11141729 | 9 | 89887534 | G | A | 1.99E-01 | -1.92E-01 | 3.19E-02 | 1.66E-09 | 99997 | 0.036% | 36.38 |
| Height | rs1118162 | 5 | 33274990 | G | A | 9.54E-02 | 2.95E-01 | 4.52E-02 | 6.29E-11 | 99997 | 0.043% | 42.65 |
| Height | rs11205303 | 1 | 149906413 | C | T | 3.69E-01 | 3.38E-01 | 2.84E-02 | 1.07E-32 | 99997 | 0.142% | 141.89 |
| Height | rs11208985 | 1 | 67464666 | A | G | 2.08E-01 | -1.80E-01 | 3.19E-02 | 1.72E-08 | 99997 | 0.032% | 31.80 |
| Height | rs1125308 | 20 | 35834024 | C | G | 2.06E-01 | 2.03E-01 | 3.27E-02 | 5.35E-10 | 99997 | 0.038% | 38.46 |
| Height | rs11259931 | 15 | 84580022 | A | G | 4.69E-01 | 3.40E-01 | 2.62E-02 | 1.76E-38 | 99997 | 0.168% | 168.30 |
| Height | rs112635299 | 14 | 94838142 | T | G | 1.69E-02 | 9.30E-01 | 1.03E-01 | 1.42E-19 | 99997 | 0.082% | 81.88 |
| Height | rs1129146 | 5 | 172550204 | T | C | 7.36E-02 | 3.22E-01 | 5.68E-02 | 1.46E-08 | 99997 | 0.032% | 32.04 |
| Height | rs115781177 | 2 | 33348494 | G | A | 8.15E-02 | -3.27E-01 | 5.24E-02 | 4.41E-10 | 99997 | 0.039% | 38.89 |
| Height | rs11605215 | 11 | 30443248 | A | G | 2.18E-01 | -1.78E-01 | 3.24E-02 | 3.90E-08 | 99997 | 0.030% | 30.15 |
| Height | rs11611726 | 12 | 12883794 | A | G | 1.32E-01 | -2.63E-01 | 4.42E-02 | 2.84E-09 | 99997 | 0.035% | 35.27 |
| Height | rs11620360 | 13 | 30345437 | G | T | 2.09E-01 | 1.91E-01 | 3.13E-02 | 1.07E-09 | 99997 | 0.037% | 37.24 |
| Height | rs11628929 | 14 | 24844054 | G | A | 3.44E-01 | -1.99E-01 | 2.83E-02 | 2.07E-12 | 99997 | 0.049% | 49.49 |
| Height | rs116376456 | 2 | 227326633 | T | A | 1.09E-02 | -7.92E-01 | 1.32E-01 | 1.69E-09 | 99997 | 0.036% | 36.30 |
| Height | rs11669516 | 19 | 19532682 | A | G | 1.65E-01 | -2.63E-01 | 3.40E-02 | 9.99E-15 | 99997 | 0.060% | 59.88 |
| Height | rs11681299 | 2 | 88901732 | T | C | 3.03E-01 | 1.70E-01 | 2.89E-02 | 4.10E-09 | 99997 | 0.035% | 34.68 |
| Height | rs116950396 | 12 | 124210007 | C | A | 3.98E-02 | 4.56E-01 | 7.24E-02 | 3.22E-10 | 99997 | 0.040% | 39.58 |
| Height | rs11720098 | 3 | 72395570 | C | T | 2.14E-01 | -2.57E-01 | 3.21E-02 | 1.23E-15 | 99997 | 0.064% | 64.15 |
| Height | rs11731421 | 4 | 1749160 | A | G | 3.36E-01 | 1.77E-01 | 2.83E-02 | 4.10E-10 | 99997 | 0.039% | 38.98 |
| Height | rs11852530 | 15 | 99550930 | T | C | 2.36E-01 | -1.81E-01 | 3.28E-02 | 3.34E-08 | 99997 | 0.031% | 30.52 |
| Height | rs11880992 | 19 | 2176403 | A | G | 4.34E-01 | 2.29E-01 | 2.71E-02 | 2.67E-17 | 99997 | 0.072% | 71.65 |
| Height | rs11935978 | 4 | 13253576 | G | A | 4.43E-01 | 1.74E-01 | 2.79E-02 | 4.56E-10 | 99997 | 0.039% | 38.85 |
| Height | rs11955153 | 5 | 170864548 | C | T | 2.36E-01 | -2.45E-01 | 3.14E-02 | 5.70E-15 | 99997 | 0.061% | 60.93 |
| Height | rs12145922 | 1 | 89146234 | A | C | 4.03E-01 | 1.74E-01 | 2.74E-02 | 2.36E-10 | 99997 | 0.040% | 40.23 |
| Height | rs12184491 | 12 | 28688552 | C | T | 2.91E-01 | -2.45E-01 | 2.92E-02 | 5.52E-17 | 99997 | 0.070% | 70.28 |
| Height | rs12211255 | 6 | 76188330 | A | C | 1.12E-01 | 3.07E-01 | 4.24E-02 | 4.63E-13 | 99997 | 0.052% | 52.25 |
| Height | rs12344515 | 9 | 113801231 | T | C | 2.51E-01 | -1.85E-01 | 3.19E-02 | 6.92E-09 | 99997 | 0.033% | 33.49 |
| Height | rs12464687 | 2 | 219884484 | G | A | 9.25E-02 | -3.33E-01 | 4.76E-02 | 2.72E-12 | 99997 | 0.049% | 48.79 |
| Height | rs12511456 | 4 | 17975603 | T | C | 1.27E-01 | -5.73E-01 | 3.77E-02 | 4.23E-52 | 99997 | 0.231% | 231.16 |
| Height | rs12529733 | 6 | 109654132 | C | T | 4.43E-01 | -1.58E-01 | 2.74E-02 | 7.93E-09 | 99997 | 0.033% | 33.38 |
| Height | rs12763284 | 10 | 104508202 | G | A | 4.70E-01 | 2.21E-01 | 2.83E-02 | 6.29E-15 | 99997 | 0.061% | 60.87 |
| Height | rs12790261 | 11 | 66988048 | A | C | 6.76E-02 | -7.96E-01 | 5.28E-02 | 2.35E-51 | 99997 | 0.226% | 226.99 |
| Height | rs12811171 | 12 | 580772 | T | C | 3.46E-01 | 2.05E-01 | 2.80E-02 | 2.93E-13 | 99997 | 0.053% | 53.39 |
| Height | rs12901188 | 15 | 77364899 | C | G | 3.14E-01 | -1.70E-01 | 2.89E-02 | 4.09E-09 | 99997 | 0.035% | 34.64 |
| Height | rs12975319 | 19 | 3414088 | A | G | 2.99E-01 | 1.60E-01 | 2.91E-02 | 4.34E-08 | 99997 | 0.030% | 30.08 |
| Height | rs13035106 | 2 | 232349119 | A | G | 2.44E-01 | -1.90E-01 | 3.30E-02 | 8.12E-09 | 99997 | 0.033% | 33.22 |
| Height | rs13092352 | 3 | 52969697 | T | C | 7.75E-02 | 3.25E-01 | 4.50E-02 | 5.47E-13 | 99997 | 0.052% | 52.03 |
| Height | rs13245690 | 7 | 120785064 | G | A | 3.58E-01 | -1.54E-01 | 2.74E-02 | 1.80E-08 | 99997 | 0.032% | 31.75 |
| Height | rs13282766 | 8 | 23174883 | A | G | 2.15E-01 | -1.79E-01 | 3.07E-02 | 5.46E-09 | 99997 | 0.034% | 34.00 |
| Height | rs13291644 | 9 | 35940552 | A | G | 3.42E-01 | -1.75E-01 | 2.83E-02 | 6.32E-10 | 99997 | 0.038% | 38.19 |
| Height | rs13338700 | 16 | 781292 | T | C | 2.39E-01 | 2.17E-01 | 3.36E-02 | 1.04E-10 | 99997 | 0.042% | 41.75 |
| Height | rs13387130 | 2 | 121600201 | A | G | 4.67E-02 | -4.32E-01 | 6.06E-02 | 1.07E-12 | 99997 | 0.051% | 50.72 |
| Height | rs1344853 | 12 | 29211556 | T | A | 3.10E-01 | -1.61E-01 | 2.86E-02 | 1.77E-08 | 99997 | 0.032% | 31.69 |
| Height | rs136029 | 22 | 46236425 | A | G | 4.19E-01 | 1.54E-01 | 2.74E-02 | 1.89E-08 | 99997 | 0.032% | 31.55 |
| Height | rs1379263 | 5 | 71849569 | A | G | 7.95E-02 | 2.99E-01 | 4.78E-02 | 4.38E-10 | 99997 | 0.039% | 39.00 |
| Height | rs138803287 | 2 | 96580405 | C | T | 1.89E-02 | -4.97E-01 | 9.02E-02 | 3.56E-08 | 99997 | 0.030% | 30.40 |
| Height | rs1401795 | 17 | 54839652 | G | A | 4.80E-01 | -1.57E-01 | 2.73E-02 | 9.19E-09 | 99997 | 0.033% | 33.03 |
| Height | rs1415359 | 1 | 103564441 | T | C | 3.82E-01 | -1.57E-01 | 2.74E-02 | 1.00E-08 | 99997 | 0.033% | 32.92 |
| Height | rs1415701 | 6 | 130345835 | A | G | 2.77E-01 | -3.02E-01 | 3.07E-02 | 7.75E-23 | 99997 | 0.097% | 97.02 |
| Height | rs1417488 | 1 | 218523730 | T | C | 2.49E-01 | 1.82E-01 | 3.06E-02 | 2.60E-09 | 99997 | 0.035% | 35.41 |
| Height | rs143384 | 20 | 34025756 | G | A | 4.12E-01 | 5.08E-01 | 2.80E-02 | 2.33E-73 | 99997 | 0.328% | 328.90 |
| Height | rs145798814 | 1 | 51084589 | T | C | 3.38E-02 | 6.46E-01 | 7.91E-02 | 3.07E-16 | 99997 | 0.067% | 66.78 |
| Height | rs1490384 | 6 | 126851160 | T | C | 4.93E-01 | 3.24E-01 | 2.72E-02 | 1.34E-32 | 99997 | 0.141% | 141.45 |
| Height | rs1507996 | 4 | 122745729 | T | C | 2.84E-01 | 1.97E-01 | 3.07E-02 | 1.42E-10 | 99997 | 0.041% | 41.22 |
| Height | rs1508822 | 5 | 33011913 | A | G | 4.73E-01 | 2.21E-01 | 2.68E-02 | 1.65E-16 | 99997 | 0.068% | 67.88 |
| Height | rs1532533 | 3 | 12635706 | A | G | 4.50E-01 | -1.53E-01 | 2.68E-02 | 1.08E-08 | 99997 | 0.033% | 32.59 |
| Height | rs153920 | 5 | 14748556 | C | T | 2.97E-01 | -1.93E-01 | 3.07E-02 | 3.43E-10 | 99997 | 0.039% | 39.40 |
| Height | rs1546646 | 2 | 191828596 | G | A | 4.07E-01 | -1.81E-01 | 2.78E-02 | 8.58E-11 | 99997 | 0.042% | 42.16 |
| Height | rs1561819 | 18 | 2712629 | G | A | 4.92E-01 | -1.45E-01 | 2.66E-02 | 4.74E-08 | 99997 | 0.030% | 29.84 |
| Height | rs1623354 | 4 | 40160011 | A | G | 2.18E-01 | 1.90E-01 | 3.22E-02 | 3.69E-09 | 99997 | 0.035% | 34.67 |
| Height | rs16937564 | 8 | 49423531 | C | T | 1.93E-01 | 2.00E-01 | 3.57E-02 | 2.09E-08 | 99997 | 0.031% | 31.48 |
| Height | rs16942324 | 15 | 89383854 | A | C | 1.89E-02 | -9.40E-01 | 1.05E-01 | 3.62E-19 | 99997 | 0.080% | 80.06 |
| Height | rs17106843 | 10 | 93367398 | A | T | 7.65E-02 | 2.70E-01 | 4.64E-02 | 6.31E-09 | 99997 | 0.034% | 33.78 |
| Height | rs17157112 | 7 | 28779946 | G | T | 4.89E-01 | -1.69E-01 | 2.74E-02 | 7.30E-10 | 99997 | 0.038% | 38.00 |
| Height | rs17178414 | 19 | 4945250 | T | C | 3.59E-01 | -1.89E-01 | 2.84E-02 | 3.06E-11 | 99997 | 0.044% | 44.15 |
| Height | rs17378160 | 1 | 176772029 | C | T | 9.54E-02 | -2.94E-01 | 4.50E-02 | 6.96E-11 | 99997 | 0.043% | 42.60 |
| Height | rs17638853 | 19 | 36234652 | C | T | 5.47E-02 | 3.37E-01 | 6.05E-02 | 2.58E-08 | 99997 | 0.031% | 31.01 |
| Height | rs177252 | 5 | 134343799 | G | A | 3.19E-01 | -2.14E-01 | 2.87E-02 | 8.18E-14 | 99997 | 0.056% | 55.65 |
| Height | rs1812175 | 4 | 145574844 | G | A | 1.86E-01 | 5.24E-01 | 3.55E-02 | 2.54E-49 | 99997 | 0.217% | 217.45 |
| Height | rs1979785 | 1 | 40777800 | G | T | 2.17E-01 | -2.09E-01 | 3.16E-02 | 3.60E-11 | 99997 | 0.044% | 43.87 |
| Height | rs1984119 | 9 | 98368761 | C | T | 2.65E-01 | -2.94E-01 | 3.09E-02 | 1.90E-21 | 99997 | 0.090% | 90.28 |
| Height | rs2000404 | 10 | 81109708 | C | T | 4.43E-01 | -2.48E-01 | 2.80E-02 | 8.27E-19 | 99997 | 0.078% | 78.38 |
| Height | rs2009382 | 8 | 116633906 | T | C | 3.92E-01 | -1.62E-01 | 2.84E-02 | 1.08E-08 | 99997 | 0.033% | 32.58 |
| Height | rs2015333 | 12 | 65862790 | C | T | 4.61E-01 | 1.55E-01 | 2.70E-02 | 1.10E-08 | 99997 | 0.033% | 32.74 |
| Height | rs2035901 | 4 | 145521867 | G | A | 4.92E-01 | 2.03E-01 | 2.69E-02 | 4.22E-14 | 99997 | 0.057% | 57.00 |
| Height | rs2045556 | 12 | 46673433 | G | A | 2.84E-01 | 1.65E-01 | 2.91E-02 | 1.43E-08 | 99997 | 0.032% | 32.07 |
| Height | rs2070776 | 17 | 62007498 | G | A | 3.47E-01 | 2.96E-01 | 2.76E-02 | 9.18E-27 | 99997 | 0.115% | 115.09 |
| Height | rs2127869 | 14 | 65794352 | C | T | 3.73E-01 | 1.62E-01 | 2.96E-02 | 4.49E-08 | 99997 | 0.030% | 29.99 |
| Height | rs2165241 | 15 | 74222202 | C | T | 4.72E-01 | 2.20E-01 | 2.63E-02 | 7.49E-17 | 99997 | 0.070% | 69.78 |
| Height | rs2230033 | 21 | 39671476 | A | G | 4.97E-01 | -1.93E-01 | 2.66E-02 | 4.69E-13 | 99997 | 0.052% | 52.43 |
| Height | rs2270894 | 3 | 9975386 | G | C | 2.16E-01 | -2.33E-01 | 3.49E-02 | 2.42E-11 | 99997 | 0.045% | 44.65 |
| Height | rs227724 | 17 | 54778817 | T | A | 3.56E-01 | 1.75E-01 | 2.85E-02 | 8.40E-10 | 99997 | 0.038% | 37.53 |
| Height | rs2282978 | 7 | 92264410 | C | T | 3.60E-01 | 3.45E-01 | 2.82E-02 | 1.70E-34 | 99997 | 0.150% | 149.93 |
| Height | rs2284746 | 1 | 17306675 | G | C | 5.00E-01 | 2.79E-01 | 2.66E-02 | 9.16E-26 | 99997 | 0.110% | 109.93 |
| Height | rs229048 | 21 | 28314030 | G | A | 2.55E-01 | 1.99E-01 | 3.19E-02 | 4.50E-10 | 99997 | 0.039% | 38.99 |
| Height | rs2295079 | 1 | 11322565 | G | C | 3.10E-01 | -1.83E-01 | 2.99E-02 | 8.76E-10 | 99997 | 0.038% | 37.62 |
| Height | rs2296316 | 14 | 65520246 | C | T | 4.61E-01 | -2.03E-01 | 2.69E-02 | 4.56E-14 | 99997 | 0.057% | 57.00 |
| Height | rs2302761 | 17 | 7358520 | T | C | 1.86E-01 | 2.47E-01 | 3.26E-02 | 3.69E-14 | 99997 | 0.057% | 57.41 |
| Height | rs2326458 | 16 | 84987679 | A | C | 2.25E-01 | -1.73E-01 | 3.03E-02 | 1.22E-08 | 99997 | 0.032% | 32.49 |
| Height | rs2350952 | 22 | 45843925 | C | G | 1.91E-01 | 1.97E-01 | 3.30E-02 | 2.32E-09 | 99997 | 0.036% | 35.71 |
| Height | rs2416564 | 9 | 119370679 | T | C | 4.23E-01 | -1.68E-01 | 2.76E-02 | 1.27E-09 | 99997 | 0.037% | 36.96 |
| Height | rs2427312 | 20 | 60970591 | T | C | 2.01E-01 | -1.86E-01 | 3.40E-02 | 4.30E-08 | 99997 | 0.030% | 30.02 |
| Height | rs2523582 | 6 | 31328092 | G | A | 2.02E-01 | -2.90E-01 | 3.73E-02 | 7.59E-15 | 99997 | 0.060% | 60.49 |
| Height | rs2547030 | 16 | 24853918 | A | G | 3.29E-01 | 2.06E-01 | 2.82E-02 | 2.67E-13 | 99997 | 0.054% | 53.57 |
| Height | rs2609301 | 6 | 168809806 | A | G | 2.74E-01 | -1.78E-01 | 3.07E-02 | 6.62E-09 | 99997 | 0.034% | 33.69 |
| Height | rs2610986 | 4 | 18037231 | T | C | 3.34E-01 | -1.98E-01 | 3.09E-02 | 1.56E-10 | 99997 | 0.041% | 40.85 |
| Height | rs2637326 | 10 | 27901359 | T | G | 4.61E-01 | 1.56E-01 | 2.71E-02 | 8.53E-09 | 99997 | 0.033% | 33.01 |
| Height | rs2724648 | 12 | 11858191 | T | C | 3.58E-01 | -2.10E-01 | 2.82E-02 | 8.39E-14 | 99997 | 0.056% | 55.61 |
| Height | rs273959 | 7 | 137598079 | C | A | 3.78E-01 | 1.55E-01 | 2.77E-02 | 2.29E-08 | 99997 | 0.031% | 31.27 |
| Height | rs2812208 | 13 | 50707087 | C | G | 3.08E-02 | 1.07E+00 | 8.58E-02 | 6.54E-36 | 99997 | 0.156% | 156.39 |
| Height | rs284662 | 19 | 41932275 | C | T | 4.03E-01 | -1.68E-01 | 2.73E-02 | 8.10E-10 | 99997 | 0.038% | 37.73 |
| Height | rs28535121 | 3 | 135892798 | C | T | 2.19E-01 | -2.24E-01 | 2.99E-02 | 6.74E-14 | 99997 | 0.056% | 56.27 |
| Height | rs2860321 | 6 | 152356619 | T | C | 2.81E-01 | 2.05E-01 | 2.92E-02 | 2.52E-12 | 99997 | 0.049% | 49.19 |
| Height | rs28657958 | 7 | 150543026 | A | G | 9.15E-02 | 2.50E-01 | 4.42E-02 | 1.48E-08 | 99997 | 0.032% | 32.04 |
| Height | rs28676499 | 12 | 123906307 | T | C | 2.06E-01 | 2.85E-01 | 3.55E-02 | 8.80E-16 | 99997 | 0.065% | 64.63 |
| Height | rs2876826 | 7 | 50581972 | G | A | 2.59E-01 | 1.81E-01 | 3.15E-02 | 9.12E-09 | 99997 | 0.033% | 32.94 |
| Height | rs2903385 | 4 | 106094427 | A | G | 4.88E-01 | 1.92E-01 | 2.64E-02 | 3.74E-13 | 99997 | 0.053% | 52.95 |
| Height | rs291970 | 10 | 121123633 | T | C | 2.22E-01 | 2.39E-01 | 3.44E-02 | 3.79E-12 | 99997 | 0.048% | 48.11 |
| Height | rs29636 | 5 | 171023919 | G | C | 2.73E-01 | 1.80E-01 | 3.00E-02 | 1.84E-09 | 99997 | 0.036% | 36.04 |
| Height | rs3116605 | 13 | 51117562 | C | T | 2.12E-01 | -3.45E-01 | 3.19E-02 | 3.02E-27 | 99997 | 0.117% | 116.83 |
| Height | rs314263 | 6 | 105392745 | T | C | 3.43E-01 | -3.37E-01 | 2.86E-02 | 4.64E-32 | 99997 | 0.139% | 139.09 |
| Height | rs34517439 | 1 | 78450517 | A | C | 9.15E-02 | 3.58E-01 | 4.25E-02 | 3.12E-17 | 99997 | 0.071% | 71.11 |
| Height | rs35628589 | 20 | 61440005 | T | C | 6.16E-02 | 3.33E-01 | 5.43E-02 | 8.95E-10 | 99997 | 0.038% | 37.59 |
| Height | rs35668185 | 5 | 168256455 | C | T | 2.14E-01 | -1.95E-01 | 3.28E-02 | 2.68E-09 | 99997 | 0.035% | 35.34 |
| Height | rs35874463 | 15 | 67457698 | G | A | 5.27E-02 | 5.58E-01 | 6.59E-02 | 2.71E-17 | 99997 | 0.072% | 71.57 |
| Height | rs35954730 | 10 | 12943111 | A | G | 3.13E-01 | -2.79E-01 | 3.02E-02 | 2.69E-20 | 99997 | 0.085% | 85.04 |
| Height | rs3748069 | 6 | 142767633 | G | A | 3.24E-01 | -4.08E-01 | 2.93E-02 | 3.98E-44 | 99997 | 0.193% | 193.42 |
| Height | rs3791679 | 2 | 56096892 | G | A | 2.29E-01 | -4.63E-01 | 3.10E-02 | 1.77E-50 | 99997 | 0.223% | 223.45 |
| Height | rs3794021 | 11 | 68567825 | C | G | 1.44E-01 | 2.39E-01 | 4.09E-02 | 5.51E-09 | 99997 | 0.034% | 34.03 |
| Height | rs3812277 | 7 | 135067234 | C | T | 3.54E-01 | 1.95E-01 | 2.85E-02 | 7.43E-12 | 99997 | 0.047% | 46.96 |
| Height | rs3823974 | 7 | 20442796 | C | T | 4.31E-01 | -2.28E-01 | 2.66E-02 | 1.04E-17 | 99997 | 0.073% | 73.27 |
| Height | rs3868142 | 16 | 67320223 | A | G | 7.16E-02 | -2.77E-01 | 4.91E-02 | 1.62E-08 | 99997 | 0.032% | 31.92 |
| Height | rs3959554 | 15 | 41443924 | G | A | 4.37E-01 | -1.63E-01 | 2.78E-02 | 4.94E-09 | 99997 | 0.034% | 34.25 |
| Height | rs4073154 | 3 | 129035485 | G | A | 1.86E-01 | 2.65E-01 | 3.27E-02 | 5.17E-16 | 99997 | 0.066% | 65.77 |
| Height | rs4284505 | 13 | 92001472 | G | A | 4.13E-01 | -1.88E-01 | 2.72E-02 | 4.43E-12 | 99997 | 0.048% | 47.77 |
| Height | rs45528934 | 14 | 23793305 | T | C | 1.77E-01 | 2.68E-01 | 3.85E-02 | 3.60E-12 | 99997 | 0.048% | 48.38 |
| Height | rs4629039 | 18 | 46485154 | A | T | 3.40E-01 | -1.86E-01 | 2.84E-02 | 6.12E-11 | 99997 | 0.043% | 42.89 |
| Height | rs4640244 | 17 | 21284223 | G | A | 3.81E-01 | -1.63E-01 | 2.79E-02 | 5.23E-09 | 99997 | 0.034% | 34.13 |
| Height | rs4713858 | 6 | 35402785 | G | A | 1.58E-01 | 2.53E-01 | 3.76E-02 | 1.84E-11 | 99997 | 0.045% | 45.24 |
| Height | rs4813800 | 20 | 6595646 | G | A | 3.81E-01 | -3.44E-01 | 2.73E-02 | 2.58E-36 | 99997 | 0.159% | 158.87 |
| Height | rs484498 | 6 | 116708729 | C | T | 4.85E-01 | 1.60E-01 | 2.64E-02 | 1.19E-09 | 99997 | 0.037% | 36.91 |
| Height | rs4894532 | 3 | 171974912 | T | G | 3.71E-01 | 2.62E-01 | 2.81E-02 | 1.27E-20 | 99997 | 0.087% | 86.93 |
| Height | rs4916664 | 5 | 88306561 | T | C | 4.83E-01 | -1.68E-01 | 2.71E-02 | 5.95E-10 | 99997 | 0.038% | 38.38 |
| Height | rs4930585 | 11 | 68411347 | C | T | 1.60E-01 | 2.08E-01 | 3.72E-02 | 2.37E-08 | 99997 | 0.031% | 31.14 |
| Height | rs493811 | 12 | 121277874 | T | G | 3.89E-01 | 1.53E-01 | 2.67E-02 | 1.14E-08 | 99997 | 0.033% | 32.71 |
| Height | rs4942556 | 13 | 47174585 | T | C | 1.80E-01 | 2.11E-01 | 3.26E-02 | 9.58E-11 | 99997 | 0.042% | 42.01 |
| Height | rs4955851 | 3 | 55479641 | A | G | 4.56E-01 | 1.61E-01 | 2.66E-02 | 1.57E-09 | 99997 | 0.036% | 36.50 |
| Height | rs4965599 | 15 | 100759884 | G | C | 3.39E-01 | -2.49E-01 | 2.85E-02 | 2.42E-18 | 99997 | 0.076% | 76.39 |
| Height | rs5024713 | 2 | 33568137 | A | G | 2.25E-01 | -2.46E-01 | 3.35E-02 | 1.92E-13 | 99997 | 0.054% | 54.05 |
| Height | rs506362 | 2 | 169721040 | A | C | 3.47E-01 | 1.69E-01 | 2.81E-02 | 1.94E-09 | 99997 | 0.036% | 36.00 |
| Height | rs52826764 | 2 | 20205541 | T | C | 2.68E-02 | -6.01E-01 | 9.04E-02 | 2.92E-11 | 99997 | 0.044% | 44.21 |
| Height | rs543650 | 6 | 152110943 | G | T | 4.17E-01 | 2.21E-01 | 2.69E-02 | 2.29E-16 | 99997 | 0.067% | 67.37 |
| Height | rs55763223 | 11 | 2773108 | C | G | 1.54E-01 | 2.13E-01 | 3.82E-02 | 2.43E-08 | 99997 | 0.031% | 31.06 |
| Height | rs55986002 | 5 | 115040734 | G | C | 2.88E-01 | 1.95E-01 | 2.98E-02 | 5.68E-11 | 99997 | 0.043% | 42.95 |
| Height | rs59051938 | 1 | 118850501 | T | G | 2.44E-01 | -3.07E-01 | 3.04E-02 | 5.17E-24 | 99997 | 0.102% | 102.05 |
| Height | rs59697941 | 4 | 73517661 | C | G | 4.27E-01 | 1.95E-01 | 2.70E-02 | 5.53E-13 | 99997 | 0.052% | 52.16 |
| Height | rs6020170 | 20 | 48597659 | G | A | 2.08E-01 | -2.46E-01 | 3.45E-02 | 1.08E-12 | 99997 | 0.051% | 50.80 |
| Height | rs6085661 | 20 | 6693128 | T | C | 3.53E-01 | 1.89E-01 | 2.79E-02 | 1.44E-11 | 99997 | 0.046% | 45.65 |
| Height | rs61144795 | 3 | 27513024 | C | T | 2.28E-01 | 1.78E-01 | 3.07E-02 | 6.52E-09 | 99997 | 0.034% | 33.77 |
| Height | rs61366632 | 6 | 81069346 | G | C | 4.18E-02 | 5.34E-01 | 6.10E-02 | 1.90E-18 | 99997 | 0.077% | 76.75 |
| Height | rs61775415 | 1 | 26391849 | C | A | 1.78E-01 | 2.15E-01 | 3.47E-02 | 5.57E-10 | 99997 | 0.038% | 38.46 |
| Height | rs61806090 | 1 | 172343604 | G | A | 1.90E-01 | 1.92E-01 | 3.24E-02 | 3.05E-09 | 99997 | 0.035% | 35.08 |
| Height | rs62068770 | 17 | 29245375 | G | C | 2.86E-01 | -3.39E-01 | 3.08E-02 | 3.50E-28 | 99997 | 0.121% | 121.21 |
| Height | rs62102286 | 18 | 46592408 | G | T | 4.37E-01 | 2.40E-01 | 2.71E-02 | 8.17E-19 | 99997 | 0.078% | 78.30 |
| Height | rs62372052 | 5 | 42724294 | G | A | 1.00E-01 | 3.47E-01 | 4.43E-02 | 4.57E-15 | 99997 | 0.061% | 61.42 |
| Height | rs62621197 | 19 | 8670147 | T | C | 5.27E-02 | -9.49E-01 | 8.80E-02 | 3.93E-27 | 99997 | 0.116% | 116.37 |
| Height | rs6437056 | 2 | 233097174 | A | G | 6.26E-02 | -4.73E-01 | 4.96E-02 | 1.66E-21 | 99997 | 0.091% | 90.86 |
| Height | rs6546548 | 2 | 70017022 | G | A | 4.55E-01 | -1.49E-01 | 2.66E-02 | 1.99E-08 | 99997 | 0.031% | 31.50 |
| Height | rs6573214 | 14 | 59268478 | G | A | 4.25E-01 | -1.54E-01 | 2.72E-02 | 1.46E-08 | 99997 | 0.032% | 32.05 |
| Height | rs659418 | 11 | 75284334 | G | T | 1.60E-01 | 3.91E-01 | 3.87E-02 | 5.08E-24 | 99997 | 0.102% | 102.13 |
| Height | rs6664147 | 1 | 227617519 | T | C | 2.31E-01 | -1.86E-01 | 3.16E-02 | 3.77E-09 | 99997 | 0.035% | 34.79 |
| Height | rs66835002 | 2 | 9655260 | C | T | 3.21E-01 | 1.84E-01 | 2.92E-02 | 2.87E-10 | 99997 | 0.040% | 39.62 |
| Height | rs66930764 | 6 | 164103243 | A | G | 1.25E-01 | 2.46E-01 | 3.88E-02 | 2.20E-10 | 99997 | 0.040% | 40.33 |
| Height | rs6720752 | 2 | 218273211 | G | A | 4.38E-01 | 1.92E-01 | 2.73E-02 | 2.01E-12 | 99997 | 0.049% | 49.51 |
| Height | rs67631072 | 1 | 38461821 | T | C | 4.67E-01 | -2.18E-01 | 2.81E-02 | 8.48E-15 | 99997 | 0.060% | 60.30 |
| Height | rs6763931 | 3 | 141102833 | A | G | 4.25E-01 | 5.32E-01 | 2.65E-02 | 6.02E-90 | 99997 | 0.401% | 403.02 |
| Height | rs67674827 | 8 | 78124967 | T | C | 2.91E-01 | 2.35E-01 | 3.00E-02 | 4.74E-15 | 99997 | 0.061% | 61.52 |
| Height | rs68014903 | 20 | 47912039 | T | G | 2.02E-01 | 2.59E-01 | 3.21E-02 | 6.76E-16 | 99997 | 0.065% | 65.05 |
| Height | rs6864688 | 5 | 112054086 | T | C | 4.93E-01 | -1.56E-01 | 2.71E-02 | 9.86E-09 | 99997 | 0.033% | 32.97 |
| Height | rs6900530 | 6 | 35280971 | T | C | 2.19E-02 | -7.34E-01 | 9.04E-02 | 4.49E-16 | 99997 | 0.066% | 65.94 |
| Height | rs6974574 | 7 | 38110073 | T | A | 3.63E-01 | 1.84E-01 | 2.80E-02 | 5.56E-11 | 99997 | 0.043% | 43.09 |
| Height | rs7027890 | 9 | 673545 | T | C | 4.99E-01 | -1.52E-01 | 2.70E-02 | 1.60E-08 | 99997 | 0.032% | 31.86 |
| Height | rs7084764 | 10 | 69960430 | A | G | 4.84E-01 | 1.64E-01 | 2.70E-02 | 1.25E-09 | 99997 | 0.037% | 36.85 |
| Height | rs7105783 | 11 | 12719794 | G | A | 3.85E-01 | 1.78E-01 | 2.91E-02 | 9.84E-10 | 99997 | 0.037% | 37.33 |
| Height | rs71313528 | 3 | 98988774 | A | G | 5.27E-02 | 2.84E-01 | 5.17E-02 | 3.94E-08 | 99997 | 0.030% | 30.20 |
| Height | rs7185686 | 16 | 84799013 | T | C | 2.49E-01 | 1.97E-01 | 3.17E-02 | 5.54E-10 | 99997 | 0.038% | 38.42 |
| Height | rs718810 | 22 | 33077216 | A | G | 8.55E-02 | -3.31E-01 | 4.88E-02 | 1.21E-11 | 99997 | 0.046% | 45.89 |
| Height | rs723149 | 7 | 46577056 | G | A | 4.59E-01 | -1.99E-01 | 2.85E-02 | 3.10E-12 | 99997 | 0.049% | 48.80 |
| Height | rs7235010 | 18 | 20724810 | A | G | 2.19E-01 | 4.40E-01 | 3.21E-02 | 1.04E-42 | 99997 | 0.188% | 188.14 |
| Height | rs72656010 | 8 | 57122215 | C | T | 1.50E-01 | -4.18E-01 | 3.96E-02 | 5.16E-26 | 99997 | 0.111% | 111.15 |
| Height | rs72721175 | 1 | 172227532 | G | A | 1.29E-01 | 2.93E-01 | 3.67E-02 | 1.31E-15 | 99997 | 0.064% | 63.74 |
| Height | rs7274811 | 20 | 32333181 | T | G | 2.40E-01 | -3.17E-01 | 3.08E-02 | 5.90E-25 | 99997 | 0.106% | 106.13 |
| Height | rs72755233 | 15 | 100692953 | A | G | 1.04E-01 | -6.08E-01 | 4.48E-02 | 5.01E-42 | 99997 | 0.184% | 184.42 |
| Height | rs72982988 | 18 | 57802714 | A | G | 2.34E-01 | 1.87E-01 | 3.15E-02 | 3.13E-09 | 99997 | 0.035% | 35.05 |
| Height | rs7305948 | 12 | 117505301 | T | C | 1.82E-01 | -2.28E-01 | 3.77E-02 | 1.47E-09 | 99997 | 0.036% | 36.51 |
| Height | rs73144827 | 7 | 73319690 | A | G | 1.34E-01 | -2.70E-01 | 4.04E-02 | 2.51E-11 | 99997 | 0.045% | 44.53 |
| Height | rs73175572 | 3 | 185490184 | G | A | 1.21E-01 | 3.97E-01 | 4.26E-02 | 1.44E-20 | 99997 | 0.087% | 86.63 |
| Height | rs74330453 | 3 | 51614203 | C | A | 1.49E-02 | 1.12E+00 | 9.92E-02 | 1.18E-29 | 99997 | 0.128% | 127.81 |
| Height | rs7466269 | 9 | 133464084 | G | A | 3.83E-01 | -2.38E-01 | 2.82E-02 | 2.63E-17 | 99997 | 0.071% | 71.35 |
| Height | rs751543 | 9 | 119122342 | T | C | 2.78E-01 | 2.26E-01 | 3.05E-02 | 1.23E-13 | 99997 | 0.055% | 54.90 |
| Height | rs754537 | 2 | 25176277 | T | A | 4.81E-01 | 2.63E-01 | 2.71E-02 | 2.69E-22 | 99997 | 0.094% | 94.40 |
| Height | rs7570235 | 2 | 242491353 | C | T | 3.60E-01 | -1.74E-01 | 2.76E-02 | 2.88E-10 | 99997 | 0.040% | 39.84 |
| Height | rs757608 | 17 | 59497277 | G | A | 3.17E-01 | -2.93E-01 | 2.82E-02 | 2.66E-25 | 99997 | 0.108% | 107.95 |
| Height | rs76497846 | 14 | 92531888 | A | G | 3.19E-01 | -2.39E-01 | 2.74E-02 | 2.68E-18 | 99997 | 0.076% | 76.15 |
| Height | rs76517946 | 2 | 68354936 | A | C | 7.36E-02 | -3.03E-01 | 5.08E-02 | 2.48E-09 | 99997 | 0.036% | 35.60 |
| Height | rs76937529 | 9 | 78505692 | T | C | 1.27E-01 | -3.01E-01 | 4.26E-02 | 1.72E-12 | 99997 | 0.050% | 49.86 |
| Height | rs7727544 | 5 | 131590534 | T | C | 4.92E-01 | 2.57E-01 | 2.66E-02 | 4.24E-22 | 99997 | 0.093% | 93.27 |
| Height | rs7742369 | 6 | 34165721 | G | A | 1.75E-01 | 4.06E-01 | 3.52E-02 | 7.66E-31 | 99997 | 0.133% | 133.23 |
| Height | rs77472938 | 15 | 70043522 | G | A | 7.26E-02 | -4.04E-01 | 4.39E-02 | 3.59E-20 | 99997 | 0.085% | 84.81 |
| Height | rs7753558 | 6 | 117523471 | A | C | 3.55E-01 | 1.96E-01 | 2.84E-02 | 4.89E-12 | 99997 | 0.048% | 47.73 |
| Height | rs77978038 | 8 | 57173316 | G | T | 2.33E-01 | 2.32E-01 | 3.12E-02 | 1.15E-13 | 99997 | 0.055% | 55.24 |
| Height | rs7816131 | 8 | 130719718 | T | A | 2.25E-01 | -3.18E-01 | 3.47E-02 | 5.27E-20 | 99997 | 0.084% | 83.72 |
| Height | rs7846369 | 8 | 13388296 | T | C | 6.16E-02 | -3.53E-01 | 6.05E-02 | 5.53E-09 | 99997 | 0.034% | 33.95 |
| Height | rs7870253 | 9 | 99231096 | A | T | 1.99E-01 | 3.13E-01 | 3.26E-02 | 6.55E-22 | 99997 | 0.092% | 92.36 |
| Height | rs788856 | 4 | 82141282 | A | G | 3.07E-01 | 2.92E-01 | 2.95E-02 | 4.10E-23 | 99997 | 0.098% | 98.24 |
| Height | rs79378907 | 22 | 29346667 | T | C | 3.58E-02 | -4.03E-01 | 6.84E-02 | 3.90E-09 | 99997 | 0.035% | 34.71 |
| Height | rs79409628 | 5 | 108113740 | T | G | 9.05E-02 | 3.51E-01 | 4.97E-02 | 1.76E-12 | 99997 | 0.050% | 49.76 |
| Height | rs7957632 | 12 | 93998321 | C | T | 2.41E-01 | 3.41E-01 | 3.18E-02 | 9.37E-27 | 99997 | 0.115% | 114.92 |
| Height | rs7968682 | 12 | 66371880 | T | G | 4.47E-01 | -3.86E-01 | 2.72E-02 | 8.21E-46 | 99997 | 0.201% | 201.39 |
| Height | rs798488 | 7 | 2802522 | C | T | 2.89E-01 | -3.88E-01 | 2.87E-02 | 1.74E-41 | 99997 | 0.182% | 182.48 |
| Height | rs8066863 | 17 | 68160901 | G | A | 1.20E-01 | -2.77E-01 | 4.15E-02 | 2.29E-11 | 99997 | 0.045% | 44.62 |
| Height | rs8074840 | 17 | 1674429 | C | T | 3.10E-01 | 1.76E-01 | 2.94E-02 | 2.12E-09 | 99997 | 0.036% | 35.80 |
| Height | rs822531 | 7 | 148629759 | T | C | 2.07E-01 | 2.59E-01 | 3.43E-02 | 4.44E-14 | 99997 | 0.057% | 57.06 |
| Height | rs8413 | 9 | 139323311 | C | T | 3.92E-01 | 1.92E-01 | 2.79E-02 | 5.49E-12 | 99997 | 0.047% | 47.46 |
| Height | rs848545 | 2 | 36705011 | C | T | 2.91E-01 | -1.66E-01 | 2.89E-02 | 1.03E-08 | 99997 | 0.033% | 32.87 |
| Height | rs849141 | 7 | 28185091 | G | A | 2.56E-01 | -3.20E-01 | 2.89E-02 | 1.49E-28 | 99997 | 0.122% | 122.52 |
| Height | rs862050 | 14 | 74981548 | G | C | 3.68E-01 | 2.06E-01 | 2.83E-02 | 2.76E-13 | 99997 | 0.053% | 53.19 |
| Height | rs888762 | 5 | 178547313 | A | C | 3.30E-01 | -1.72E-01 | 2.87E-02 | 2.24E-09 | 99997 | 0.036% | 35.87 |
| Height | rs889495 | 16 | 2233565 | C | T | 4.07E-01 | -2.25E-01 | 2.83E-02 | 1.59E-15 | 99997 | 0.063% | 63.38 |
| Height | rs894344 | 8 | 135612745 | G | A | 4.27E-01 | -2.59E-01 | 2.74E-02 | 3.20E-21 | 99997 | 0.089% | 89.42 |
| Height | rs9358913 | 6 | 26239404 | G | A | 2.91E-01 | -3.64E-01 | 3.01E-02 | 8.26E-34 | 99997 | 0.146% | 146.48 |
| Height | rs9369705 | 6 | 47470521 | A | C | 3.12E-01 | 2.05E-01 | 2.96E-02 | 4.43E-12 | 99997 | 0.048% | 48.06 |
| Height | rs9379084 | 6 | 7231843 | A | G | 1.32E-01 | -2.93E-01 | 4.48E-02 | 5.87E-11 | 99997 | 0.043% | 42.83 |
| Height | rs9442571 | 1 | 9349611 | A | T | 1.21E-01 | 2.57E-01 | 3.94E-02 | 6.67E-11 | 99997 | 0.042% | 42.51 |
| Height | rs945631 | 1 | 93426167 | A | G | 3.98E-02 | 4.82E-01 | 6.84E-02 | 1.90E-12 | 99997 | 0.050% | 49.55 |
| Height | rs955748 | 4 | 184215675 | G | A | 2.48E-01 | 1.77E-01 | 3.13E-02 | 1.58E-08 | 99997 | 0.032% | 31.87 |
| Height | rs9590409 | 13 | 115040497 | A | G | 2.57E-01 | -1.81E-01 | 3.14E-02 | 7.71E-09 | 99997 | 0.033% | 33.34 |
| Height | rs9672558 | 15 | 99180205 | C | T | 1.63E-01 | -2.99E-01 | 3.82E-02 | 5.42E-15 | 99997 | 0.061% | 61.22 |
| Height | rs9877909 | 3 | 158144264 | T | C | 4.50E-01 | -2.37E-01 | 2.67E-02 | 7.17E-19 | 99997 | 0.078% | 78.52 |
| Height | rs9919526 | 11 | 47451623 | T | C | 2.93E-01 | 1.86E-01 | 2.91E-02 | 1.62E-10 | 99997 | 0.041% | 40.85 |
| Height | rs9974406 | 21 | 47435594 | A | T | 2.64E-01 | -1.90E-01 | 3.16E-02 | 1.73E-09 | 99997 | 0.036% | 36.23 |
| Hand grip strength (right) | rs58670122 | 1 | 22492613 | G | A | 1.43E-01 | -1.32E-02 | 2.15E-03 | 7.40E-10 | 461089 | 0.008% | 37.90 |
| Hand grip strength (right) | rs10798876 | 1 | 32074514 | G | C | 5.52E-01 | 8.79E-03 | 1.50E-03 | 4.30E-09 | 461089 | 0.007% | 34.47 |
| Hand grip strength (right) | rs10798483 | 1 | 176799143 | A | G | 5.47E-01 | 1.45E-02 | 1.49E-03 | 2.80E-22 | 461089 | 0.020% | 94.22 |
| Hand grip strength (right) | rs6693965 | 1 | 10378416 | T | G | 1.28E-01 | -1.63E-02 | 2.23E-03 | 3.20E-13 | 461089 | 0.012% | 53.09 |
| Hand grip strength (right) | rs4927015 | 1 | 54072471 | A | G | 5.83E-01 | 1.31E-02 | 1.51E-03 | 5.00E-18 | 461089 | 0.016% | 74.90 |
| Hand grip strength (right) | rs1952256 | 1 | 184035116 | G | A | 3.45E-01 | 9.77E-03 | 1.56E-03 | 4.10E-10 | 461089 | 0.008% | 39.04 |
| Hand grip strength (right) | rs35304341 | 1 | 200971049 | A | G | 8.95E-02 | -1.43E-02 | 2.60E-03 | 4.00E-08 | 461089 | 0.007% | 30.13 |
| Hand grip strength (right) | rs823130 | 1 | 205714372 | T | C | 4.33E-01 | -1.24E-02 | 1.50E-03 | 1.70E-16 | 461089 | 0.015% | 67.90 |
| Hand grip strength (right) | rs56144131 | 1 | 208977656 | C | T | 1.49E-01 | -1.31E-02 | 2.09E-03 | 3.80E-10 | 461089 | 0.009% | 39.23 |
| Hand grip strength (right) | rs7549184 | 1 | 215412906 | A | G | 7.87E-01 | 1.06E-02 | 1.81E-03 | 5.80E-09 | 461089 | 0.007% | 33.90 |
| Hand grip strength (right) | rs10799428 | 1 | 227798565 | T | C | 1.87E-01 | -1.44E-02 | 1.90E-03 | 4.60E-14 | 461089 | 0.012% | 56.89 |
| Hand grip strength (right) | rs12562146 | 1 | 86247267 | A | T | 1.45E-01 | 1.19E-02 | 2.12E-03 | 2.00E-08 | 461089 | 0.007% | 31.48 |
| Hand grip strength (right) | rs2147461 | 1 | 191051627 | C | T | 1.17E-01 | 1.34E-02 | 2.31E-03 | 6.10E-09 | 461089 | 0.007% | 33.79 |
| Hand grip strength (right) | rs4121165 | 1 | 78276977 | A | G | 2.11E-01 | -1.20E-02 | 1.82E-03 | 4.80E-11 | 461089 | 0.009% | 43.25 |
| Hand grip strength (right) | rs6693567 | 1 | 150510660 | T | C | 7.33E-01 | -9.75E-03 | 1.68E-03 | 6.30E-09 | 461089 | 0.007% | 33.75 |
| Hand grip strength (right) | rs1892425 | 1 | 41744821 | A | G | 2.39E-01 | 1.13E-02 | 1.76E-03 | 1.10E-10 | 461089 | 0.009% | 41.61 |
| Hand grip strength (right) | rs150330307 | 1 | 160160801 | C | T | 3.19E-02 | -3.26E-02 | 4.22E-03 | 1.20E-14 | 461089 | 0.013% | 59.50 |
| Hand grip strength (right) | rs1550115 | 2 | 25041620 | T | C | 7.49E-01 | 1.53E-02 | 1.71E-03 | 4.50E-19 | 461089 | 0.017% | 79.63 |
| Hand grip strength (right) | rs1442883 | 2 | 59970660 | A | C | 2.53E-01 | -1.06E-02 | 1.71E-03 | 5.80E-10 | 461089 | 0.008% | 38.37 |
| Hand grip strength (right) | rs6711390 | 2 | 135629439 | T | C | 3.71E-01 | 1.33E-02 | 1.53E-03 | 4.00E-18 | 461089 | 0.016% | 75.30 |
| Hand grip strength (right) | rs35833641 | 2 | 179462494 | G | A | 3.12E-01 | 9.22E-03 | 1.60E-03 | 8.50E-09 | 461089 | 0.007% | 33.15 |
| Hand grip strength (right) | rs1840753 | 2 | 199060177 | T | C | 6.28E-02 | 1.77E-02 | 3.12E-03 | 1.40E-08 | 461089 | 0.007% | 32.14 |
| Hand grip strength (right) | rs1047891 | 2 | 211540507 | A | C | 3.16E-01 | 9.69E-03 | 1.60E-03 | 1.30E-09 | 461089 | 0.008% | 36.81 |
| Hand grip strength (right) | rs2894602 | 2 | 227249802 | G | A | 7.66E-01 | 1.01E-02 | 1.77E-03 | 9.90E-09 | 461089 | 0.007% | 32.86 |
| Hand grip strength (right) | rs7565148 | 2 | 44188396 | G | T | 5.01E-01 | -1.04E-02 | 1.49E-03 | 3.60E-12 | 461089 | 0.010% | 48.31 |
| Hand grip strength (right) | rs7576964 | 2 | 48601448 | T | G | 3.42E-01 | 9.75E-03 | 1.57E-03 | 5.30E-10 | 461089 | 0.008% | 38.58 |
| Hand grip strength (right) | rs34030812 | 2 | 144248905 | C | T | 3.67E-01 | -9.13E-03 | 1.54E-03 | 3.30E-09 | 461089 | 0.008% | 35.02 |
| Hand grip strength (right) | rs7575451 | 2 | 152352843 | G | C | 6.50E-01 | -1.06E-02 | 1.56E-03 | 9.30E-12 | 461089 | 0.010% | 46.47 |
| Hand grip strength (right) | rs12616285 | 2 | 169156907 | G | T | 1.49E-01 | 1.22E-02 | 2.09E-03 | 5.40E-09 | 461089 | 0.007% | 34.05 |
| Hand grip strength (right) | rs6715064 | 2 | 220041928 | T | C | 3.10E-01 | -9.21E-03 | 1.61E-03 | 9.60E-09 | 461089 | 0.007% | 32.91 |
| Hand grip strength (right) | rs1641457 | 2 | 40421990 | G | T | 2.23E-01 | 1.31E-02 | 1.79E-03 | 2.40E-13 | 461089 | 0.012% | 53.61 |
| Hand grip strength (right) | rs3771498 | 2 | 70720070 | T | C | 5.14E-01 | 1.42E-02 | 1.49E-03 | 1.90E-21 | 461089 | 0.020% | 90.49 |
| Hand grip strength (right) | rs12052508 | 2 | 72757843 | T | C | 8.79E-01 | -1.34E-02 | 2.28E-03 | 4.70E-09 | 461089 | 0.007% | 34.30 |
| Hand grip strength (right) | rs10193039 | 2 | 99692373 | T | A | 2.81E-01 | -1.03E-02 | 1.65E-03 | 4.10E-10 | 461089 | 0.008% | 39.08 |
| Hand grip strength (right) | rs2194747 | 2 | 218126181 | G | A | 7.07E-01 | 9.85E-03 | 1.64E-03 | 1.90E-09 | 461089 | 0.008% | 36.12 |
| Hand grip strength (right) | rs6792762 | 3 | 38574491 | A | G | 4.20E-01 | -9.12E-03 | 1.52E-03 | 1.80E-09 | 461089 | 0.008% | 36.19 |
| Hand grip strength (right) | rs7652177 | 3 | 171969077 | G | C | 5.05E-01 | 8.58E-03 | 1.49E-03 | 7.90E-09 | 461089 | 0.007% | 33.31 |
| Hand grip strength (right) | rs2194411 | 3 | 185548663 | A | G | 1.28E-01 | 1.43E-02 | 2.25E-03 | 2.10E-10 | 461089 | 0.009% | 40.37 |
| Hand grip strength (right) | rs1440152 | 3 | 98489915 | G | C | 4.45E-01 | 8.27E-03 | 1.50E-03 | 3.50E-08 | 461089 | 0.007% | 30.41 |
| Hand grip strength (right) | rs2362972 | 3 | 158163272 | A | C | 5.78E-01 | -8.57E-03 | 1.51E-03 | 1.30E-08 | 461089 | 0.007% | 32.30 |
| Hand grip strength (right) | rs62234790 | 3 | 13750642 | A | C | 2.46E-01 | 1.06E-02 | 1.73E-03 | 8.80E-10 | 461089 | 0.008% | 37.58 |
| Hand grip strength (right) | rs35701422 | 3 | 85575775 | C | T | 6.26E-01 | -8.69E-03 | 1.53E-03 | 1.50E-08 | 461089 | 0.007% | 32.07 |
| Hand grip strength (right) | rs2341184 | 3 | 196930781 | C | T | 2.72E-01 | 1.01E-02 | 1.67E-03 | 1.70E-09 | 461089 | 0.008% | 36.24 |
| Hand grip strength (right) | rs35457492 | 3 | 70194564 | C | A | 4.95E-01 | 8.34E-03 | 1.49E-03 | 2.20E-08 | 461089 | 0.007% | 31.28 |
| Hand grip strength (right) | rs9757079 | 3 | 53155158 | T | C | 3.18E-01 | 9.80E-03 | 1.60E-03 | 8.40E-10 | 461089 | 0.008% | 37.67 |
| Hand grip strength (right) | rs71298370 | 3 | 71164965 | A | G | 8.62E-02 | 1.66E-02 | 2.71E-03 | 8.90E-10 | 461089 | 0.008% | 37.56 |
| Hand grip strength (right) | rs9853018 | 3 | 141101961 | T | C | 4.43E-01 | 1.02E-02 | 1.49E-03 | 8.80E-12 | 461089 | 0.010% | 46.57 |
| Hand grip strength (right) | rs34587452 | 4 | 1009900 | C | G | 2.15E-01 | -1.11E-02 | 1.81E-03 | 9.10E-10 | 461089 | 0.008% | 37.51 |
| Hand grip strength (right) | rs7657558 | 4 | 30648636 | G | T | 7.20E-01 | 1.07E-02 | 1.66E-03 | 1.10E-10 | 461089 | 0.009% | 41.63 |
| Hand grip strength (right) | rs114924396 | 4 | 119755621 | G | A | 5.36E-02 | -1.91E-02 | 3.32E-03 | 8.20E-09 | 461089 | 0.007% | 33.23 |
| Hand grip strength (right) | rs13146142 | 4 | 17931318 | C | T | 1.59E-01 | -2.08E-02 | 2.03E-03 | 1.40E-24 | 461089 | 0.023% | 104.78 |
| Hand grip strength (right) | rs13106087 | 4 | 145566864 | C | T | 8.30E-01 | 1.29E-02 | 1.98E-03 | 6.20E-11 | 461089 | 0.009% | 42.75 |
| Hand grip strength (right) | rs997850 | 4 | 154838434 | C | G | 6.05E-01 | -9.02E-03 | 1.53E-03 | 3.70E-09 | 461089 | 0.008% | 34.77 |
| Hand grip strength (right) | rs13107325 | 4 | 103188709 | T | C | 7.49E-02 | -2.75E-02 | 2.82E-03 | 2.10E-22 | 461089 | 0.021% | 94.78 |
| Hand grip strength (right) | rs13169333 | 5 | 153064994 | C | T | 2.60E-01 | 9.29E-03 | 1.70E-03 | 4.30E-08 | 461089 | 0.007% | 30.00 |
| Hand grip strength (right) | rs75457267 | 5 | 102658770 | T | C | 5.12E-02 | -1.87E-02 | 3.39E-03 | 3.40E-08 | 461089 | 0.007% | 30.44 |
| Hand grip strength (right) | rs12522139 | 5 | 122699812 | G | T | 1.71E-01 | -1.14E-02 | 1.97E-03 | 6.80E-09 | 461089 | 0.007% | 33.60 |
| Hand grip strength (right) | rs6882168 | 5 | 39402647 | T | C | 3.37E-01 | -9.09E-03 | 1.58E-03 | 7.90E-09 | 461089 | 0.007% | 33.29 |
| Hand grip strength (right) | rs13356200 | 5 | 67820946 | G | T | 3.94E-01 | -9.21E-03 | 1.53E-03 | 1.80E-09 | 461089 | 0.008% | 36.19 |
| Hand grip strength (right) | rs6870324 | 5 | 141787317 | G | C | 2.70E-01 | -9.99E-03 | 1.68E-03 | 3.00E-09 | 461089 | 0.008% | 35.20 |
| Hand grip strength (right) | rs13355365 | 5 | 154942606 | T | C | 3.80E-01 | -8.47E-03 | 1.53E-03 | 3.40E-08 | 461089 | 0.007% | 30.46 |
| Hand grip strength (right) | rs4868110 | 5 | 171164168 | T | A | 3.23E-01 | -9.71E-03 | 1.59E-03 | 1.00E-09 | 461089 | 0.008% | 37.31 |
| Hand grip strength (right) | rs2431112 | 5 | 103931707 | A | G | 4.41E-01 | -1.12E-02 | 1.50E-03 | 8.60E-14 | 461089 | 0.012% | 55.67 |
| Hand grip strength (right) | rs2631360 | 5 | 131707429 | A | G | 5.19E-01 | -1.12E-02 | 1.49E-03 | 5.40E-14 | 461089 | 0.012% | 56.57 |
| Hand grip strength (right) | rs2322754 | 6 | 81046299 | A | G | 8.32E-01 | -1.17E-02 | 1.99E-03 | 3.80E-09 | 461089 | 0.008% | 34.74 |
| Hand grip strength (right) | rs9322822 | 6 | 105369598 | T | C | 3.20E-01 | 1.11E-02 | 1.59E-03 | 3.50E-12 | 461089 | 0.010% | 48.40 |
| Hand grip strength (right) | rs9388051 | 6 | 122577108 | A | G | 1.86E-01 | 1.06E-02 | 1.91E-03 | 2.70E-08 | 461089 | 0.007% | 30.90 |
| Hand grip strength (right) | rs7451021 | 6 | 130381246 | C | T | 6.89E-01 | -1.58E-02 | 1.61E-03 | 7.70E-23 | 461089 | 0.021% | 96.78 |
| Hand grip strength (right) | rs113835839 | 6 | 13784625 | T | C | 2.48E-01 | -9.86E-03 | 1.72E-03 | 1.10E-08 | 461089 | 0.007% | 32.70 |
| Hand grip strength (right) | rs11243202 | 6 | 7719065 | C | T | 4.86E-01 | 1.16E-02 | 1.49E-03 | 6.50E-15 | 461089 | 0.013% | 60.76 |
| Hand grip strength (right) | rs645144 | 6 | 141180780 | C | T | 3.30E-01 | -8.69E-03 | 1.59E-03 | 4.30E-08 | 461089 | 0.007% | 30.00 |
| Hand grip strength (right) | rs721101 | 6 | 155632844 | C | T | 2.71E-01 | 9.47E-03 | 1.67E-03 | 1.60E-08 | 461089 | 0.007% | 31.97 |
| Hand grip strength (right) | rs77485342 | 6 | 30842866 | T | C | 1.80E-02 | 3.53E-02 | 5.59E-03 | 2.60E-10 | 461089 | 0.009% | 39.96 |
| Hand grip strength (right) | rs9267806 | 6 | 32110886 | A | G | 2.56E-01 | -1.67E-02 | 1.70E-03 | 7.70E-23 | 461089 | 0.021% | 96.78 |
| Hand grip strength (right) | rs185320691 | 6 | 32490292 | C | G | 1.04E-01 | -2.02E-02 | 2.69E-03 | 5.60E-14 | 461089 | 0.012% | 56.52 |
| Hand grip strength (right) | rs113315602 | 6 | 32574575 | C | A | 9.58E-02 | -2.13E-02 | 2.67E-03 | 1.40E-15 | 461089 | 0.014% | 63.70 |
| Hand grip strength (right) | rs1125 | 6 | 149979416 | A | G | 3.37E-01 | -1.00E-02 | 1.57E-03 | 1.90E-10 | 461089 | 0.009% | 40.57 |
| Hand grip strength (right) | rs9396861 | 6 | 18404133 | A | C | 5.99E-01 | -9.63E-03 | 1.55E-03 | 5.70E-10 | 461089 | 0.008% | 38.43 |
| Hand grip strength (right) | rs35175534 | 6 | 32530029 | C | A | 1.40E-01 | -1.92E-02 | 2.36E-03 | 4.20E-16 | 461089 | 0.014% | 66.16 |
| Hand grip strength (right) | rs1885690 | 6 | 109672998 | A | C | 4.10E-01 | -8.40E-03 | 1.51E-03 | 2.80E-08 | 461089 | 0.007% | 30.86 |
| Hand grip strength (right) | rs852520 | 7 | 5695267 | A | C | 6.62E-01 | -8.90E-03 | 1.57E-03 | 1.50E-08 | 461089 | 0.007% | 32.03 |
| Hand grip strength (right) | rs4549685 | 7 | 39326478 | T | C | 3.30E-01 | 9.71E-03 | 1.58E-03 | 8.10E-10 | 461089 | 0.008% | 37.75 |
| Hand grip strength (right) | rs112330055 | 7 | 23109316 | A | G | 6.30E-02 | 1.80E-02 | 3.19E-03 | 1.70E-08 | 461089 | 0.007% | 31.78 |
| Hand grip strength (right) | rs7790322 | 7 | 2830498 | T | C | 4.16E-01 | -8.60E-03 | 1.51E-03 | 1.20E-08 | 461089 | 0.007% | 32.49 |
| Hand grip strength (right) | rs6962338 | 7 | 69160985 | G | A | 4.40E-02 | -2.03E-02 | 3.62E-03 | 2.00E-08 | 461089 | 0.007% | 31.54 |
| Hand grip strength (right) | rs6977081 | 7 | 150542515 | T | G | 3.34E-01 | 1.29E-02 | 1.59E-03 | 5.70E-16 | 461089 | 0.014% | 65.53 |
| Hand grip strength (right) | rs2389763 | 7 | 17307847 | C | T | 5.92E-01 | -8.33E-03 | 1.51E-03 | 3.80E-08 | 461089 | 0.007% | 30.26 |
| Hand grip strength (right) | rs2717351 | 7 | 19019880 | G | A | 2.12E-01 | 1.28E-02 | 1.83E-03 | 3.40E-12 | 461089 | 0.011% | 48.44 |
| Hand grip strength (right) | rs9639938 | 7 | 46262729 | G | C | 5.41E-01 | 8.73E-03 | 1.49E-03 | 5.20E-09 | 461089 | 0.007% | 34.11 |
| Hand grip strength (right) | rs10278546 | 7 | 100516003 | C | A | 1.95E-01 | 1.08E-02 | 1.88E-03 | 1.10E-08 | 461089 | 0.007% | 32.59 |
| Hand grip strength (right) | rs4730984 | 7 | 120655676 | T | G | 2.40E-01 | 1.05E-02 | 1.74E-03 | 1.80E-09 | 461089 | 0.008% | 36.17 |
| Hand grip strength (right) | rs4737446 | 8 | 57665019 | T | G | 6.95E-01 | 1.03E-02 | 1.62E-03 | 2.40E-10 | 461089 | 0.009% | 40.10 |
| Hand grip strength (right) | rs62509875 | 8 | 110360944 | G | A | 1.71E-01 | -1.32E-02 | 1.97E-03 | 2.40E-11 | 461089 | 0.010% | 44.61 |
| Hand grip strength (right) | rs1486925 | 8 | 78827617 | C | T | 3.15E-01 | -9.53E-03 | 1.60E-03 | 2.90E-09 | 461089 | 0.008% | 35.26 |
| Hand grip strength (right) | rs6473015 | 8 | 78178485 | C | A | 2.86E-01 | 9.60E-03 | 1.65E-03 | 5.50E-09 | 461089 | 0.007% | 34.00 |
| Hand grip strength (right) | rs7871404 | 9 | 99262296 | G | A | 1.89E-01 | 1.19E-02 | 1.90E-03 | 3.40E-10 | 461089 | 0.009% | 39.42 |
| Hand grip strength (right) | rs116922558 | 9 | 118802375 | G | A | 3.99E-02 | -2.46E-02 | 3.86E-03 | 1.80E-10 | 461089 | 0.009% | 40.69 |
| Hand grip strength (right) | rs2208562 | 9 | 119344528 | T | C | 6.10E-01 | -1.16E-02 | 1.52E-03 | 3.10E-14 | 461089 | 0.013% | 57.67 |
| Hand grip strength (right) | rs7034200 | 9 | 4289050 | A | C | 4.80E-01 | 8.69E-03 | 1.49E-03 | 5.30E-09 | 461089 | 0.007% | 34.06 |
| Hand grip strength (right) | rs113851275 | 9 | 98297220 | A | G | 1.08E-01 | 1.31E-02 | 2.40E-03 | 4.30E-08 | 461089 | 0.007% | 30.03 |
| Hand grip strength (right) | rs11998884 | 9 | 33684436 | T | C | 6.18E-02 | 1.73E-02 | 3.12E-03 | 2.80E-08 | 461089 | 0.007% | 30.81 |
| Hand grip strength (right) | rs600038 | 9 | 136151806 | C | T | 2.07E-01 | -1.04E-02 | 1.83E-03 | 1.50E-08 | 461089 | 0.007% | 32.02 |
| Hand grip strength (right) | rs10761411 | 9 | 136973826 | T | C | 8.12E-01 | -1.15E-02 | 2.02E-03 | 1.30E-08 | 461089 | 0.007% | 32.37 |
| Hand grip strength (right) | rs72820369 | 10 | 81251539 | T | A | 1.18E-01 | 1.61E-02 | 2.33E-03 | 4.40E-12 | 461089 | 0.010% | 47.92 |
| Hand grip strength (right) | rs4751671 | 10 | 116138744 | A | G | 5.31E-01 | 8.23E-03 | 1.50E-03 | 4.10E-08 | 461089 | 0.007% | 30.12 |
| Hand grip strength (right) | rs4752689 | 10 | 124131176 | A | G | 5.84E-01 | 8.75E-03 | 1.51E-03 | 6.40E-09 | 461089 | 0.007% | 33.71 |
| Hand grip strength (right) | rs12412806 | 10 | 24860913 | A | G | 2.94E-01 | -9.10E-03 | 1.64E-03 | 3.00E-08 | 461089 | 0.007% | 30.71 |
| Hand grip strength (right) | rs2273555 | 10 | 104127171 | A | G | 6.06E-01 | 1.10E-02 | 1.52E-03 | 4.20E-13 | 461089 | 0.011% | 52.57 |
| Hand grip strength (right) | rs4962700 | 10 | 126479989 | G | C | 3.02E-01 | 9.33E-03 | 1.64E-03 | 1.20E-08 | 461089 | 0.007% | 32.45 |
| Hand grip strength (right) | rs1556659 | 10 | 130834698 | T | C | 3.82E-01 | 1.75E-02 | 1.54E-03 | 3.80E-30 | 461089 | 0.028% | 130.17 |
| Hand grip strength (right) | rs12763284 | 10 | 104508202 | G | A | 4.66E-01 | 9.60E-03 | 1.49E-03 | 1.20E-10 | 461089 | 0.009% | 41.53 |
| Hand grip strength (right) | rs11022513 | 11 | 12840986 | T | C | 5.69E-01 | -9.21E-03 | 1.51E-03 | 1.10E-09 | 461089 | 0.008% | 37.20 |
| Hand grip strength (right) | rs11039348 | 11 | 47728617 | A | G | 3.48E-01 | -9.77E-03 | 1.56E-03 | 3.90E-10 | 461089 | 0.008% | 39.16 |
| Hand grip strength (right) | rs2244621 | 11 | 64026219 | T | C | 1.44E-01 | 1.16E-02 | 2.12E-03 | 4.50E-08 | 461089 | 0.006% | 29.94 |
| Hand grip strength (right) | rs61389091 | 11 | 74427921 | T | C | 4.18E-02 | 2.21E-02 | 3.74E-03 | 3.60E-09 | 461089 | 0.008% | 34.82 |
| Hand grip strength (right) | rs34845616 | 11 | 133792644 | A | G | 2.46E-01 | 9.80E-03 | 1.74E-03 | 1.70E-08 | 461089 | 0.007% | 31.83 |
| Hand grip strength (right) | rs12790261 | 11 | 66988048 | A | C | 8.24E-02 | -2.64E-02 | 2.71E-03 | 2.00E-22 | 461089 | 0.021% | 94.88 |
| Hand grip strength (right) | rs72977282 | 11 | 74300441 | A | T | 4.14E-01 | -1.68E-02 | 1.51E-03 | 1.20E-28 | 461089 | 0.027% | 123.29 |
| Hand grip strength (right) | rs6592737 | 11 | 77322619 | T | A | 3.72E-01 | -9.25E-03 | 1.54E-03 | 2.00E-09 | 461089 | 0.008% | 36.00 |
| Hand grip strength (right) | rs10770125 | 11 | 2169014 | G | A | 4.77E-01 | 8.44E-03 | 1.49E-03 | 1.40E-08 | 461089 | 0.007% | 32.19 |
| Hand grip strength (right) | rs1635527 | 12 | 48396364 | C | G | 5.47E-01 | 9.75E-03 | 1.50E-03 | 6.80E-11 | 461089 | 0.009% | 42.56 |
| Hand grip strength (right) | rs76895963 | 12 | 4384844 | G | T | 2.07E-02 | 3.60E-02 | 5.76E-03 | 4.10E-10 | 461089 | 0.008% | 39.05 |
| Hand grip strength (right) | rs7301953 | 12 | 124405871 | A | G | 3.12E-01 | -1.15E-02 | 1.60E-03 | 6.40E-13 | 461089 | 0.011% | 51.73 |
| Hand grip strength (right) | rs10846071 | 12 | 15016236 | T | C | 3.94E-01 | -1.57E-02 | 1.52E-03 | 6.50E-25 | 461089 | 0.023% | 106.25 |
| Hand grip strength (right) | rs12823922 | 12 | 24186697 | G | A | 2.22E-01 | -1.14E-02 | 1.79E-03 | 1.60E-10 | 461089 | 0.009% | 40.87 |
| Hand grip strength (right) | rs10784502 | 12 | 66343810 | T | C | 5.12E-01 | -1.11E-02 | 1.49E-03 | 7.10E-14 | 461089 | 0.012% | 56.04 |
| Hand grip strength (right) | rs7963801 | 12 | 79685226 | C | T | 5.72E-01 | -1.13E-02 | 1.51E-03 | 8.40E-14 | 461089 | 0.012% | 55.70 |
| Hand grip strength (right) | rs12316046 | 12 | 15054415 | G | A | 3.78E-01 | -1.62E-02 | 1.53E-03 | 3.80E-26 | 461089 | 0.024% | 111.86 |
| Hand grip strength (right) | rs4768725 | 12 | 46848478 | C | T | 7.00E-01 | 9.06E-03 | 1.62E-03 | 2.30E-08 | 461089 | 0.007% | 31.24 |
| Hand grip strength (right) | rs7953280 | 12 | 94136009 | C | G | 5.07E-01 | -8.99E-03 | 1.49E-03 | 1.80E-09 | 461089 | 0.008% | 36.16 |
| Hand grip strength (right) | rs3118914 | 13 | 51116901 | T | G | 2.15E-01 | -1.94E-02 | 1.81E-03 | 5.90E-27 | 461089 | 0.025% | 115.56 |
| Hand grip strength (right) | rs2296316 | 14 | 65520246 | C | T | 4.64E-01 | -8.21E-03 | 1.50E-03 | 4.60E-08 | 461089 | 0.006% | 29.90 |
| Hand grip strength (right) | rs12889267 | 14 | 21542766 | G | A | 1.67E-01 | -1.23E-02 | 1.99E-03 | 6.70E-10 | 461089 | 0.008% | 38.11 |
| Hand grip strength (right) | rs935728 | 14 | 80957923 | T | C | 3.28E-01 | 9.55E-03 | 1.59E-03 | 1.80E-09 | 461089 | 0.008% | 36.23 |
| Hand grip strength (right) | rs7148603 | 14 | 36683779 | A | G | 3.59E-01 | 9.31E-03 | 1.59E-03 | 4.70E-09 | 461089 | 0.007% | 34.33 |
| Hand grip strength (right) | rs10483727 | 14 | 61072875 | C | T | 6.10E-01 | -9.02E-03 | 1.52E-03 | 3.30E-09 | 461089 | 0.008% | 34.99 |
| Hand grip strength (right) | rs9652468 | 15 | 56823913 | A | G | 2.49E-01 | -1.25E-02 | 1.72E-03 | 3.30E-13 | 461089 | 0.011% | 53.00 |
| Hand grip strength (right) | rs2871865 | 15 | 99194896 | G | C | 1.16E-01 | -2.37E-02 | 2.32E-03 | 1.80E-24 | 461089 | 0.023% | 104.22 |
| Hand grip strength (right) | rs2165241 | 15 | 74222202 | C | T | 5.09E-01 | 1.22E-02 | 1.49E-03 | 1.90E-16 | 461089 | 0.015% | 67.72 |
| Hand grip strength (right) | rs4553566 | 15 | 58336319 | C | T | 4.53E-01 | -9.30E-03 | 1.49E-03 | 4.60E-10 | 461089 | 0.008% | 38.85 |
| Hand grip strength (right) | rs12914702 | 15 | 96887277 | A | G | 3.00E-01 | 1.08E-02 | 1.69E-03 | 1.90E-10 | 461089 | 0.009% | 40.54 |
| Hand grip strength (right) | rs12101479 | 15 | 74248548 | C | G | 2.37E-01 | -1.06E-02 | 1.76E-03 | 1.50E-09 | 461089 | 0.008% | 36.56 |
| Hand grip strength (right) | rs12899474 | 15 | 77391603 | T | C | 1.08E-01 | -1.49E-02 | 2.40E-03 | 5.40E-10 | 461089 | 0.008% | 38.53 |
| Hand grip strength (right) | rs246181 | 16 | 14392641 | T | C | 3.73E-01 | 9.70E-03 | 1.55E-03 | 3.70E-10 | 461089 | 0.009% | 39.27 |
| Hand grip strength (right) | rs11642954 | 16 | 24824248 | A | G | 1.95E-01 | -1.32E-02 | 1.88E-03 | 2.20E-12 | 461089 | 0.011% | 49.27 |
| Hand grip strength (right) | rs7196917 | 16 | 69896527 | G | A | 4.30E-01 | -1.06E-02 | 1.50E-03 | 1.90E-12 | 461089 | 0.011% | 49.58 |
| Hand grip strength (right) | rs8055199 | 16 | 84867404 | A | G | 6.60E-01 | -8.96E-03 | 1.57E-03 | 1.20E-08 | 461089 | 0.007% | 32.48 |
| Hand grip strength (right) | rs7206195 | 16 | 2145280 | T | C | 1.80E-01 | -1.53E-02 | 1.94E-03 | 2.80E-15 | 461089 | 0.014% | 62.42 |
| Hand grip strength (right) | rs248831 | 16 | 11281218 | A | G | 2.65E-01 | 9.89E-03 | 1.72E-03 | 8.40E-09 | 461089 | 0.007% | 33.18 |
| Hand grip strength (right) | rs62037412 | 16 | 28917746 | A | G | 3.57E-01 | 9.33E-03 | 1.55E-03 | 1.90E-09 | 461089 | 0.008% | 36.03 |
| Hand grip strength (right) | rs4785574 | 16 | 89568875 | G | A | 5.55E-01 | -1.04E-02 | 1.50E-03 | 4.50E-12 | 461089 | 0.010% | 47.89 |
| Hand grip strength (right) | rs3848369 | 16 | 415078 | T | C | 3.87E-01 | -9.54E-03 | 1.53E-03 | 4.70E-10 | 461089 | 0.008% | 38.80 |
| Hand grip strength (right) | rs76749769 | 16 | 3291408 | T | C | 9.12E-02 | 1.44E-02 | 2.58E-03 | 2.20E-08 | 461089 | 0.007% | 31.30 |
| Hand grip strength (right) | rs4784329 | 16 | 53910261 | C | A | 4.26E-01 | -1.33E-02 | 1.51E-03 | 9.00E-19 | 461089 | 0.017% | 78.27 |
| Hand grip strength (right) | rs7214252 | 17 | 27486673 | A | G | 2.11E-01 | -1.02E-02 | 1.83E-03 | 2.00E-08 | 461089 | 0.007% | 31.50 |
| Hand grip strength (right) | rs2854152 | 17 | 61986027 | G | A | 6.78E-01 | 1.10E-02 | 1.60E-03 | 6.00E-12 | 461089 | 0.010% | 47.33 |
| Hand grip strength (right) | rs1043515 | 17 | 36922196 | G | A | 5.66E-01 | 1.38E-02 | 1.50E-03 | 2.80E-20 | 461089 | 0.018% | 85.16 |
| Hand grip strength (right) | rs12452505 | 17 | 63556402 | G | C | 1.42E-01 | -1.44E-02 | 2.14E-03 | 1.40E-11 | 461089 | 0.010% | 45.67 |
| Hand grip strength (right) | rs2587505 | 17 | 77784268 | C | T | 4.20E-01 | -9.27E-03 | 1.51E-03 | 8.10E-10 | 461089 | 0.008% | 37.75 |
| Hand grip strength (right) | rs4793658 | 17 | 45878733 | C | A | 1.10E-01 | -1.37E-02 | 2.49E-03 | 3.80E-08 | 461089 | 0.007% | 30.24 |
| Hand grip strength (right) | rs56074046 | 17 | 7358930 | A | G | 3.72E-01 | -9.04E-03 | 1.54E-03 | 4.40E-09 | 461089 | 0.007% | 34.44 |
| Hand grip strength (right) | rs56365901 | 17 | 43960323 | G | A | 2.23E-01 | -1.43E-02 | 1.79E-03 | 1.80E-15 | 461089 | 0.014% | 63.27 |
| Hand grip strength (right) | rs10520770 | 18 | 46602964 | C | T | 4.49E-01 | 1.17E-02 | 1.50E-03 | 4.50E-15 | 461089 | 0.013% | 61.46 |
| Hand grip strength (right) | rs635538 | 18 | 53273614 | A | G | 9.14E-01 | -2.20E-02 | 2.66E-03 | 1.50E-16 | 461089 | 0.015% | 68.23 |
| Hand grip strength (right) | rs4369779 | 18 | 20735408 | C | T | 7.89E-01 | 1.72E-02 | 1.82E-03 | 3.40E-21 | 461089 | 0.019% | 89.28 |
| Hand grip strength (right) | rs34217742 | 19 | 37376830 | A | T | 1.24E-01 | 1.46E-02 | 2.28E-03 | 1.40E-10 | 461089 | 0.009% | 41.22 |
| Hand grip strength (right) | rs7249 | 19 | 18391171 | T | C | 3.66E-01 | 8.48E-03 | 1.54E-03 | 4.00E-08 | 461089 | 0.007% | 30.16 |
| Hand grip strength (right) | rs4802848 | 19 | 52218342 | C | G | 7.30E-01 | 1.11E-02 | 1.67E-03 | 3.80E-11 | 461089 | 0.009% | 43.73 |
| Hand grip strength (right) | rs36065733 | 19 | 2163771 | G | T | 4.73E-01 | 9.72E-03 | 1.50E-03 | 8.20E-11 | 461089 | 0.009% | 42.22 |
| Hand grip strength (right) | rs79723785 | 19 | 55818225 | C | T | 1.65E-02 | -3.41E-02 | 6.03E-03 | 1.60E-08 | 461089 | 0.007% | 31.90 |
| Hand grip strength (right) | rs7266065 | 20 | 47531817 | A | G | 3.23E-01 | 9.94E-03 | 1.59E-03 | 4.60E-10 | 461089 | 0.008% | 38.84 |
| Hand grip strength (right) | rs911642 | 20 | 13260252 | T | C | 3.76E-01 | 8.63E-03 | 1.54E-03 | 2.00E-08 | 461089 | 0.007% | 31.49 |
| Hand grip strength (right) | rs6063504 | 20 | 48981014 | G | C | 4.93E-01 | 8.62E-03 | 1.49E-03 | 7.80E-09 | 461089 | 0.007% | 33.33 |
| Hand grip strength (right) | rs143384 | 20 | 34025756 | G | A | 4.04E-01 | 2.30E-02 | 1.51E-03 | 2.50E-52 | 461089 | 0.050% | 231.74 |
| Hand grip strength (right) | rs2226685 | 21 | 40069825 | C | T | 7.59E-01 | 1.03E-02 | 1.74E-03 | 3.10E-09 | 461089 | 0.008% | 35.14 |
| Hand grip strength (right) | rs6006984 | 22 | 45714937 | C | T | 2.78E-01 | 1.03E-02 | 1.66E-03 | 5.10E-10 | 461089 | 0.008% | 38.65 |
| Usual walking pace | rs2297600 | 1 | 32207581 | G | T | 1.72E-01 | -1.16E-02 | 1.68E-03 | 6.20E-12 | 459915 | 0.010% | 47.26 |
| Usual walking pace | rs113825410 | 1 | 40057543 | G | A | 2.24E-01 | -8.81E-03 | 1.52E-03 | 6.40E-09 | 459915 | 0.007% | 33.71 |
| Usual walking pace | rs11548200 | 1 | 156290656 | C | T | 6.60E-02 | -1.60E-02 | 2.55E-03 | 3.30E-10 | 459915 | 0.009% | 39.52 |
| Usual walking pace | rs2644135 | 1 | 201856256 | G | C | 6.56E-01 | 7.51E-03 | 1.33E-03 | 1.70E-08 | 459915 | 0.007% | 31.78 |
| Usual walking pace | rs12747822 | 1 | 91201451 | A | T | 9.79E-02 | 1.18E-02 | 2.14E-03 | 3.80E-08 | 459915 | 0.007% | 30.24 |
| Usual walking pace | rs12042959 | 1 | 243533273 | G | A | 1.44E-01 | 1.26E-02 | 1.81E-03 | 4.10E-12 | 459915 | 0.010% | 48.06 |
| Usual walking pace | rs55680124 | 2 | 105984624 | T | C | 1.55E-01 | -1.07E-02 | 1.75E-03 | 8.30E-10 | 459915 | 0.008% | 37.69 |
| Usual walking pace | rs1592 | 2 | 135722143 | C | A | 3.95E-01 | 7.45E-03 | 1.29E-03 | 7.30E-09 | 459915 | 0.007% | 33.46 |
| Usual walking pace | rs139398785 | 2 | 161132876 | G | C | 1.40E-01 | -1.00E-02 | 1.82E-03 | 4.20E-08 | 459915 | 0.007% | 30.05 |
| Usual walking pace | rs11682482 | 2 | 226486479 | G | T | 6.81E-01 | 8.02E-03 | 1.36E-03 | 3.30E-09 | 459915 | 0.008% | 34.97 |
| Usual walking pace | rs2280406 | 3 | 49941436 | A | G | 5.06E-01 | -9.96E-03 | 1.27E-03 | 3.40E-15 | 459915 | 0.013% | 62.01 |
| Usual walking pace | rs830627 | 3 | 71675270 | A | G | 4.17E-01 | 7.42E-03 | 1.29E-03 | 7.70E-09 | 459915 | 0.007% | 33.34 |
| Usual walking pace | rs6763292 | 3 | 129044705 | G | A | 7.81E-01 | 9.59E-03 | 1.53E-03 | 3.80E-10 | 459915 | 0.009% | 39.23 |
| Usual walking pace | rs28519617 | 3 | 135874930 | G | T | 2.70E-01 | -8.13E-03 | 1.43E-03 | 1.40E-08 | 459915 | 0.007% | 32.16 |
| Usual walking pace | rs57800857 | 4 | 140863365 | C | A | 3.65E-01 | 8.68E-03 | 1.32E-03 | 5.30E-11 | 459915 | 0.009% | 43.06 |
| Usual walking pace | rs11732213 | 4 | 1704244 | C | T | 1.96E-01 | 9.16E-03 | 1.60E-03 | 9.40E-09 | 459915 | 0.007% | 32.95 |
| Usual walking pace | rs144333966 | 4 | 61109385 | G | A | 1.46E-02 | 3.05E-02 | 5.45E-03 | 2.20E-08 | 459915 | 0.007% | 31.28 |
| Usual walking pace | rs13107325 | 4 | 103188709 | T | C | 7.49E-02 | -2.42E-02 | 2.41E-03 | 7.10E-24 | 459915 | 0.022% | 101.51 |
| Usual walking pace | rs35711462 | 5 | 50847577 | G | A | 5.11E-01 | -7.17E-03 | 1.27E-03 | 1.70E-08 | 459915 | 0.007% | 31.86 |
| Usual walking pace | rs205262 | 6 | 34563164 | G | A | 2.69E-01 | -8.71E-03 | 1.43E-03 | 1.00E-09 | 459915 | 0.008% | 37.27 |
| Usual walking pace | rs11152989 | 6 | 96936061 | T | C | 3.12E-01 | -7.51E-03 | 1.37E-03 | 3.90E-08 | 459915 | 0.007% | 30.20 |
| Usual walking pace | rs4715208 | 6 | 50829471 | G | A | 7.53E-01 | -8.38E-03 | 1.47E-03 | 1.10E-08 | 459915 | 0.007% | 32.72 |
| Usual walking pace | rs4839898 | 6 | 97546759 | A | G | 1.10E-01 | 1.31E-02 | 2.06E-03 | 2.30E-10 | 459915 | 0.009% | 40.16 |
| Usual walking pace | rs9366651 | 6 | 26336696 | T | G | 5.07E-01 | 9.49E-03 | 1.27E-03 | 8.60E-14 | 459915 | 0.012% | 55.67 |
| Usual walking pace | rs7789719 | 7 | 66893916 | C | T | 7.82E-01 | 8.54E-03 | 1.54E-03 | 2.70E-08 | 459915 | 0.007% | 30.88 |
| Usual walking pace | rs11761141 | 7 | 69423362 | G | T | 3.26E-01 | -7.90E-03 | 1.35E-03 | 5.00E-09 | 459915 | 0.007% | 34.17 |
| Usual walking pace | rs7795394 | 7 | 113560607 | A | T | 6.25E-01 | 9.30E-03 | 1.31E-03 | 1.20E-12 | 459915 | 0.011% | 50.55 |
| Usual walking pace | rs4109292 | 10 | 126710654 | A | G | 4.94E-01 | 7.35E-03 | 1.27E-03 | 6.60E-09 | 459915 | 0.007% | 33.64 |
| Usual walking pace | rs7896518 | 10 | 65104500 | G | A | 4.28E-01 | 9.99E-03 | 1.29E-03 | 1.10E-14 | 459915 | 0.013% | 59.73 |
| Usual walking pace | rs10883618 | 10 | 103117653 | A | G | 3.72E-01 | 7.81E-03 | 1.31E-03 | 2.40E-09 | 459915 | 0.008% | 35.64 |
| Usual walking pace | rs10828258 | 10 | 21929734 | G | A | 3.19E-01 | -9.33E-03 | 1.36E-03 | 6.70E-12 | 459915 | 0.010% | 47.11 |
| Usual walking pace | rs2439823 | 10 | 99778226 | G | A | 5.46E-01 | -7.28E-03 | 1.27E-03 | 1.10E-08 | 459915 | 0.007% | 32.62 |
| Usual walking pace | rs11039324 | 11 | 47665686 | A | G | 4.04E-01 | -1.02E-02 | 1.29E-03 | 2.20E-15 | 459915 | 0.014% | 62.88 |
| Usual walking pace | rs9783304 | 11 | 43660255 | T | G | 6.89E-01 | 7.64E-03 | 1.37E-03 | 2.40E-08 | 459915 | 0.007% | 31.14 |
| Usual walking pace | rs10750025 | 11 | 113424042 | T | C | 6.82E-01 | -8.36E-03 | 1.37E-03 | 9.40E-10 | 459915 | 0.008% | 37.45 |
| Usual walking pace | rs10862220 | 12 | 81430599 | G | T | 6.75E-01 | 8.41E-03 | 1.35E-03 | 4.80E-10 | 459915 | 0.008% | 38.76 |
| Usual walking pace | rs2645979 | 12 | 84017043 | A | G | 3.57E-01 | 8.62E-03 | 1.32E-03 | 6.70E-11 | 459915 | 0.009% | 42.61 |
| Usual walking pace | rs2170670 | 12 | 16944621 | A | G | 6.07E-01 | -7.09E-03 | 1.30E-03 | 4.70E-08 | 459915 | 0.006% | 29.85 |
| Usual walking pace | rs12883788 | 14 | 33303540 | T | C | 4.60E-01 | -7.80E-03 | 1.27E-03 | 9.40E-10 | 459915 | 0.008% | 37.45 |
| Usual walking pace | rs45583845 | 14 | 57858194 | G | C | 3.43E-02 | -1.99E-02 | 3.63E-03 | 4.30E-08 | 459915 | 0.007% | 30.01 |
| Usual walking pace | rs8011870 | 14 | 80173397 | A | G | 2.88E-01 | -7.83E-03 | 1.41E-03 | 2.60E-08 | 459915 | 0.007% | 30.98 |
| Usual walking pace | rs8010773 | 14 | 46956863 | C | T | 3.82E-01 | -8.17E-03 | 1.30E-03 | 3.40E-10 | 459915 | 0.009% | 39.42 |
| Usual walking pace | rs11848096 | 14 | 100969235 | C | T | 3.87E-01 | -7.51E-03 | 1.31E-03 | 9.20E-09 | 459915 | 0.007% | 33.00 |
| Usual walking pace | rs8028757 | 15 | 75822794 | T | A | 1.30E-01 | 1.07E-02 | 1.92E-03 | 2.40E-08 | 459915 | 0.007% | 31.11 |
| Usual walking pace | rs11150623 | 16 | 28881001 | T | G | 6.45E-01 | -9.69E-03 | 1.32E-03 | 2.40E-13 | 459915 | 0.012% | 53.67 |
| Usual walking pace | rs62048402 | 16 | 53803223 | A | G | 4.04E-01 | -9.88E-03 | 1.29E-03 | 1.90E-14 | 459915 | 0.013% | 58.59 |
| Usual walking pace | rs4516268 | 17 | 1846831 | A | C | 1.94E-01 | 9.83E-03 | 1.61E-03 | 9.50E-10 | 459915 | 0.008% | 37.43 |
| Usual walking pace | rs4643373 | 17 | 47123423 | C | T | 3.00E-01 | 7.73E-03 | 1.38E-03 | 2.30E-08 | 459915 | 0.007% | 31.25 |
| Usual walking pace | rs11077815 | 17 | 74389890 | C | T | 6.24E-01 | -7.13E-03 | 1.31E-03 | 4.80E-08 | 459915 | 0.006% | 29.79 |
| Usual walking pace | rs613872 | 18 | 53210302 | T | G | 8.26E-01 | -1.47E-02 | 1.67E-03 | 1.30E-18 | 459915 | 0.017% | 77.51 |
| Usual walking pace | rs891387 | 18 | 21103909 | C | T | 4.95E-01 | 7.86E-03 | 1.27E-03 | 5.40E-10 | 459915 | 0.008% | 38.54 |
| Usual walking pace | rs11881338 | 19 | 18838014 | A | T | 4.87E-01 | 8.17E-03 | 1.27E-03 | 1.30E-10 | 459915 | 0.009% | 41.29 |
| Usual walking pace | rs2602731 | 19 | 4944771 | G | A | 6.81E-01 | -7.67E-03 | 1.37E-03 | 2.10E-08 | 459915 | 0.007% | 31.36 |
| Usual walking pace | rs273512 | 19 | 18224729 | T | C | 4.05E-01 | -9.67E-03 | 1.29E-03 | 6.90E-14 | 459915 | 0.012% | 56.08 |
| Usual walking pace | rs2037735 | 19 | 48012469 | T | C | 1.21E-01 | -1.13E-02 | 1.94E-03 | 6.70E-09 | 459915 | 0.007% | 33.63 |
| Usual walking pace | rs12461902 | 19 | 30265235 | A | G | 3.30E-01 | -8.01E-03 | 1.36E-03 | 3.60E-09 | 459915 | 0.008% | 34.83 |
| Usual walking pace | rs819167 | 20 | 32903687 | G | A | 9.36E-01 | -1.58E-02 | 2.59E-03 | 1.10E-09 | 459915 | 0.008% | 37.13 |
| BMI | rs7550711 | 1 | 110082886 | T | C | 3.06E-02 | 6.49E-02 | 5.00E-03 | 3.20E-38 | 681275 | 0.025% | 168.48 |
| BMI | rs7704281 | 5 | 50591460 | A | G | 4.53E-02 | 2.71E-02 | 4.10E-03 | 6.50E-11 | 681275 | 0.006% | 43.69 |
| BMI | rs7899106 | 10 | 87410904 | G | A | 4.78E-02 | 3.31E-02 | 3.70E-03 | 1.00E-18 | 681275 | 0.012% | 80.03 |
| BMI | rs11505821 | 7 | 76818677 | T | A | 6.01E-02 | 3.11E-02 | 3.50E-03 | 2.70E-19 | 681275 | 0.012% | 78.96 |
| BMI | rs9688431 | 6 | 73922654 | C | T | 6.03E-02 | -2.31E-02 | 3.50E-03 | 2.40E-11 | 681275 | 0.006% | 43.56 |
| BMI | rs4495304 | 6 | 31080718 | C | T | 6.70E-02 | -1.94E-02 | 3.30E-03 | 5.00E-09 | 681275 | 0.005% | 34.56 |
| BMI | rs17119937 | 8 | 14502274 | C | T | 6.91E-02 | 2.12E-02 | 3.60E-03 | 5.60E-09 | 681275 | 0.005% | 34.68 |
| BMI | rs16851483 | 3 | 141275436 | T | G | 6.93E-02 | 3.69E-02 | 3.50E-03 | 3.20E-26 | 681275 | 0.016% | 111.15 |
| BMI | rs13107325 | 4 | 103188709 | T | C | 7.37E-02 | 4.70E-02 | 3.20E-03 | 1.10E-47 | 681275 | 0.032% | 215.72 |
| BMI | rs8097783 | 18 | 58051294 | A | G | 7.55E-02 | -3.89E-02 | 3.10E-03 | 7.20E-36 | 681275 | 0.023% | 157.46 |
| BMI | rs12762034 | 10 | 33969931 | C | T | 7.58E-02 | 2.40E-02 | 3.20E-03 | 7.30E-14 | 681275 | 0.008% | 56.25 |
| BMI | rs13069244 | 3 | 180441172 | A | G | 7.75E-02 | 1.87E-02 | 3.20E-03 | 3.00E-09 | 681275 | 0.005% | 34.15 |
| BMI | rs12369179 | 12 | 122963550 | T | C | 8.78E-02 | -3.59E-02 | 3.10E-03 | 2.50E-31 | 681275 | 0.020% | 134.11 |
| BMI | rs17425707 | 1 | 57874879 | C | T | 1.00E-01 | 1.67E-02 | 2.80E-03 | 4.40E-09 | 681275 | 0.005% | 35.57 |
| BMI | rs17535749 | 3 | 10027724 | A | G | 1.02E-01 | 1.50E-02 | 2.70E-03 | 2.50E-08 | 681275 | 0.005% | 30.86 |
| BMI | rs10984756 | 9 | 122651784 | G | C | 1.05E-01 | 1.74E-02 | 2.90E-03 | 1.10E-09 | 681275 | 0.005% | 36.00 |
| BMI | rs12933482 | 16 | 72189604 | G | A | 1.05E-01 | 1.86E-02 | 2.80E-03 | 4.90E-11 | 681275 | 0.006% | 44.13 |
| BMI | rs7811342 | 7 | 138794618 | C | T | 1.06E-01 | -1.97E-02 | 2.90E-03 | 1.10E-11 | 681275 | 0.007% | 46.15 |
| BMI | rs1006896 | 3 | 88104411 | C | A | 1.06E-01 | -2.34E-02 | 2.70E-03 | 5.50E-18 | 681275 | 0.011% | 75.11 |
| BMI | rs12914489 | 15 | 74187937 | A | G | 1.10E-01 | 1.65E-02 | 2.60E-03 | 3.80E-10 | 681275 | 0.006% | 40.27 |
| BMI | rs4148155 | 4 | 89054667 | G | A | 1.13E-01 | -1.88E-02 | 2.60E-03 | 5.00E-13 | 681275 | 0.008% | 52.28 |
| BMI | rs12675063 | 8 | 132879047 | T | A | 1.13E-01 | 1.56E-02 | 2.60E-03 | 1.30E-09 | 681275 | 0.005% | 36.00 |
| BMI | rs17663412 | 5 | 167595121 | A | C | 1.14E-01 | 1.57E-02 | 2.70E-03 | 6.10E-09 | 681275 | 0.005% | 33.81 |
| BMI | rs12564992 | 1 | 174478100 | G | A | 1.14E-01 | 1.96E-02 | 2.60E-03 | 5.30E-14 | 681275 | 0.008% | 56.83 |
| BMI | rs1409818 | 20 | 21381121 | T | C | 1.16E-01 | 2.01E-02 | 2.90E-03 | 2.50E-12 | 681275 | 0.007% | 48.04 |
| BMI | rs769449 | 19 | 45410002 | A | G | 1.16E-01 | -2.54E-02 | 2.70E-03 | 2.30E-20 | 681275 | 0.013% | 88.50 |
| BMI | rs2143253 | 20 | 41987392 | A | G | 1.19E-01 | -1.88E-02 | 2.60E-03 | 1.10E-12 | 681275 | 0.008% | 52.28 |
| BMI | rs13191362 | 6 | 163033350 | G | A | 1.20E-01 | -2.36E-02 | 2.50E-03 | 5.90E-21 | 681275 | 0.013% | 89.11 |
| BMI | rs12098284 | 10 | 76047464 | T | C | 1.24E-01 | 1.78E-02 | 2.60E-03 | 1.80E-11 | 681275 | 0.007% | 46.87 |
| BMI | rs12429545 | 13 | 54102206 | A | G | 1.25E-01 | 3.16E-02 | 2.50E-03 | 9.60E-38 | 681275 | 0.023% | 159.77 |
| BMI | rs17446257 | 13 | 40749213 | A | G | 1.29E-01 | 1.53E-02 | 2.60E-03 | 2.90E-09 | 681275 | 0.005% | 34.63 |
| BMI | rs8123881 | 20 | 15819495 | G | A | 1.30E-01 | 1.96E-02 | 2.40E-03 | 4.40E-16 | 681275 | 0.010% | 66.69 |
| BMI | rs2051559 | 4 | 3298800 | C | T | 1.31E-01 | 1.76E-02 | 2.60E-03 | 5.00E-12 | 681275 | 0.007% | 45.82 |
| BMI | rs4722398 | 7 | 3125220 | T | C | 1.34E-01 | 1.58E-02 | 2.50E-03 | 3.60E-10 | 681275 | 0.006% | 39.94 |
| BMI | rs17014375 | 1 | 209543560 | G | T | 1.35E-01 | 1.72E-02 | 2.50E-03 | 1.10E-11 | 681275 | 0.007% | 47.33 |
| BMI | rs4556997 | 2 | 100814858 | A | C | 1.35E-01 | 1.97E-02 | 2.40E-03 | 6.90E-17 | 681275 | 0.010% | 67.38 |
| BMI | rs6712 | 22 | 50637922 | C | G | 1.37E-01 | 1.38E-02 | 2.50E-03 | 4.40E-08 | 681275 | 0.004% | 30.47 |
| BMI | rs3904244 | 10 | 27361527 | A | T | 1.38E-01 | 1.55E-02 | 2.50E-03 | 4.30E-10 | 681275 | 0.006% | 38.44 |
| BMI | rs1982441 | 8 | 28021769 | T | G | 1.38E-01 | 1.75E-02 | 2.60E-03 | 7.00E-12 | 681275 | 0.007% | 45.30 |
| BMI | rs16903285 | 5 | 87978252 | C | T | 1.41E-01 | 3.31E-02 | 2.60E-03 | 7.60E-38 | 681275 | 0.024% | 162.07 |
| BMI | rs10858334 | 9 | 137989785 | G | C | 1.42E-01 | 1.43E-02 | 2.60E-03 | 2.70E-08 | 681275 | 0.004% | 30.25 |
| BMI | rs10510419 | 3 | 12426936 | T | G | 1.42E-01 | -1.77E-02 | 2.30E-03 | 2.20E-14 | 681275 | 0.009% | 59.22 |
| BMI | rs10518694 | 15 | 53072673 | A | C | 1.42E-01 | 1.46E-02 | 2.50E-03 | 3.30E-09 | 681275 | 0.005% | 34.11 |
| BMI | rs774246 | 7 | 26990816 | G | A | 1.44E-01 | 1.53E-02 | 2.50E-03 | 5.40E-10 | 681275 | 0.005% | 37.45 |
| BMI | rs1477199 | 16 | 53712135 | G | A | 1.45E-01 | 2.28E-02 | 2.40E-03 | 9.40E-22 | 681275 | 0.013% | 90.25 |
| BMI | rs7615297 | 3 | 156299313 | G | C | 1.47E-01 | -1.49E-02 | 2.40E-03 | 5.70E-10 | 681275 | 0.006% | 38.54 |
| BMI | rs11889536 | 2 | 220163543 | G | A | 1.49E-01 | -1.89E-02 | 2.40E-03 | 6.40E-15 | 681275 | 0.009% | 62.02 |
| BMI | rs11084553 | 19 | 31019780 | G | A | 1.52E-01 | -2.10E-02 | 2.40E-03 | 1.80E-18 | 681275 | 0.011% | 76.56 |
| BMI | rs3764835 | 2 | 159519368 | A | G | 1.53E-01 | -1.41E-02 | 2.40E-03 | 3.10E-09 | 681275 | 0.005% | 34.52 |
| BMI | rs8097672 | 18 | 1839601 | T | A | 1.53E-01 | 2.00E-02 | 2.50E-03 | 8.40E-16 | 681275 | 0.009% | 64.00 |
| BMI | rs7871866 | 9 | 131027982 | C | G | 1.53E-01 | 1.87E-02 | 2.40E-03 | 2.30E-14 | 681275 | 0.009% | 60.71 |
| BMI | rs13174863 | 5 | 139080745 | G | A | 1.55E-01 | 1.92E-02 | 2.30E-03 | 2.90E-16 | 681275 | 0.010% | 69.69 |
| BMI | rs13240600 | 7 | 99064466 | G | A | 1.55E-01 | -2.04E-02 | 2.40E-03 | 3.50E-17 | 681275 | 0.011% | 72.25 |
| BMI | rs17001561 | 4 | 77096118 | A | G | 1.57E-01 | 1.51E-02 | 2.30E-03 | 3.80E-11 | 681275 | 0.006% | 43.10 |
| BMI | rs998732 | 19 | 19378671 | G | A | 1.58E-01 | -1.71E-02 | 2.20E-03 | 2.00E-14 | 681275 | 0.009% | 60.42 |
| BMI | rs4589691 | 2 | 144051398 | G | C | 1.58E-01 | 1.41E-02 | 2.40E-03 | 4.70E-09 | 681275 | 0.005% | 34.52 |
| BMI | rs12364470 | 11 | 134601012 | G | T | 1.63E-01 | 1.78E-02 | 2.20E-03 | 1.10E-15 | 681275 | 0.010% | 65.46 |
| BMI | rs2246012 | 6 | 131898208 | C | T | 1.63E-01 | 1.58E-02 | 2.20E-03 | 3.10E-13 | 681275 | 0.008% | 51.58 |
| BMI | rs17238110 | 15 | 62150364 | G | A | 1.63E-01 | -3.53E-02 | 5.00E-03 | 2.00E-12 | 681275 | 0.007% | 49.84 |
| BMI | rs10009336 | 4 | 44480783 | T | C | 1.64E-01 | -1.40E-02 | 2.20E-03 | 2.20E-10 | 681275 | 0.006% | 40.50 |
| BMI | rs10192119 | 2 | 164581241 | G | T | 1.67E-01 | 1.66E-02 | 2.20E-03 | 3.00E-14 | 681275 | 0.008% | 56.93 |
| BMI | rs7117238 | 11 | 78040259 | A | G | 1.68E-01 | -1.31E-02 | 2.20E-03 | 2.50E-09 | 681275 | 0.005% | 35.46 |
| BMI | rs6471941 | 8 | 62117973 | A | G | 1.68E-01 | 1.56E-02 | 2.10E-03 | 3.10E-13 | 681275 | 0.008% | 55.18 |
| BMI | rs40067 | 5 | 107439012 | A | G | 1.71E-01 | -2.66E-02 | 2.30E-03 | 7.10E-30 | 681275 | 0.020% | 133.75 |
| BMI | rs872281 | 14 | 40834177 | T | C | 1.73E-01 | -1.51E-02 | 2.30E-03 | 4.70E-11 | 681275 | 0.006% | 43.10 |
| BMI | rs8071182 | 17 | 55336155 | A | G | 1.74E-01 | 1.33E-02 | 2.20E-03 | 2.10E-09 | 681275 | 0.005% | 36.55 |
| BMI | rs1112613 | 13 | 53651850 | A | G | 1.76E-01 | -1.33E-02 | 2.30E-03 | 3.40E-09 | 681275 | 0.005% | 33.44 |
| BMI | rs2868975 | 3 | 116935323 | A | G | 1.78E-01 | -1.43E-02 | 2.30E-03 | 2.20E-10 | 681275 | 0.006% | 38.66 |
| BMI | rs17551974 | 2 | 142293146 | A | C | 1.78E-01 | -1.41E-02 | 2.20E-03 | 1.90E-10 | 681275 | 0.006% | 41.08 |
| BMI | rs17806379 | 20 | 51107290 | T | C | 1.79E-01 | -2.58E-02 | 2.20E-03 | 1.50E-30 | 681275 | 0.020% | 137.53 |
| BMI | rs10962550 | 9 | 16720329 | C | G | 1.80E-01 | 1.82E-02 | 2.20E-03 | 6.20E-16 | 681275 | 0.010% | 68.44 |
| BMI | rs987237 | 6 | 50803050 | G | A | 1.80E-01 | 4.09E-02 | 2.10E-03 | 9.30E-84 | 681275 | 0.056% | 379.32 |
| BMI | rs11538 | 22 | 18220831 | G | A | 1.81E-01 | 1.35E-02 | 2.30E-03 | 3.30E-09 | 681275 | 0.005% | 34.45 |
| BMI | rs3828783 | 6 | 33767727 | A | G | 1.81E-01 | -1.65E-02 | 2.10E-03 | 5.60E-15 | 681275 | 0.009% | 61.73 |
| BMI | rs1522569 | 4 | 171632637 | G | T | 1.82E-01 | -1.64E-02 | 2.20E-03 | 2.90E-13 | 681275 | 0.008% | 55.57 |
| BMI | rs9926784 | 16 | 19941968 | C | T | 1.82E-01 | -2.58E-02 | 2.10E-03 | 9.90E-35 | 681275 | 0.022% | 150.94 |
| BMI | rs12629015 | 3 | 119618053 | G | A | 1.85E-01 | -1.35E-02 | 2.30E-03 | 2.10E-09 | 681275 | 0.005% | 34.45 |
| BMI | rs1445652 | 2 | 155668460 | A | G | 1.86E-01 | 1.23E-02 | 2.20E-03 | 4.30E-08 | 681275 | 0.005% | 31.26 |
| BMI | rs6591407 | 11 | 56914157 | A | C | 1.86E-01 | -1.18E-02 | 2.10E-03 | 1.90E-08 | 681275 | 0.005% | 31.57 |
| BMI | rs17033117 | 3 | 35443653 | T | C | 1.87E-01 | 1.37E-02 | 2.20E-03 | 8.90E-10 | 681275 | 0.006% | 38.78 |
| BMI | rs6448587 | 4 | 28561990 | C | A | 1.89E-01 | -1.67E-02 | 2.30E-03 | 2.30E-13 | 681275 | 0.008% | 52.72 |
| BMI | rs11781699 | 8 | 118863061 | C | T | 1.90E-01 | 1.32E-02 | 2.10E-03 | 3.10E-10 | 681275 | 0.006% | 39.51 |
| BMI | rs7148846 | 14 | 40133821 | G | T | 1.90E-01 | 1.24E-02 | 2.20E-03 | 2.20E-08 | 681275 | 0.005% | 31.77 |
| BMI | rs17499593 | 2 | 172649755 | G | C | 1.90E-01 | 1.25E-02 | 2.20E-03 | 1.10E-08 | 681275 | 0.005% | 32.28 |
| BMI | rs895330 | 19 | 4060707 | G | C | 1.92E-01 | -2.01E-02 | 2.30E-03 | 5.50E-19 | 681275 | 0.011% | 76.37 |
| BMI | rs4516268 | 17 | 1846831 | A | C | 1.93E-01 | -2.17E-02 | 2.10E-03 | 5.20E-25 | 681275 | 0.016% | 106.78 |
| BMI | rs6265 | 11 | 27679916 | T | C | 1.95E-01 | -4.12E-02 | 2.10E-03 | 1.00E-86 | 681275 | 0.056% | 384.91 |
| BMI | rs543874 | 1 | 177889480 | G | A | 1.95E-01 | 4.75E-02 | 2.00E-03 | 1.20E-122 | 681275 | 0.083% | 564.06 |
| BMI | rs17203016 | 2 | 208255518 | G | A | 1.96E-01 | 1.50E-02 | 2.00E-03 | 2.10E-13 | 681275 | 0.008% | 56.25 |
| BMI | rs262130 | 6 | 142853486 | T | C | 1.97E-01 | 1.27E-02 | 2.30E-03 | 1.80E-08 | 681275 | 0.004% | 30.49 |
| BMI | rs12334877 | 8 | 67194171 | A | G | 1.98E-01 | -1.44E-02 | 2.20E-03 | 7.70E-11 | 681275 | 0.006% | 42.84 |
| BMI | rs6500208 | 16 | 49011249 | A | G | 2.01E-01 | 1.40E-02 | 2.00E-03 | 4.10E-12 | 681275 | 0.007% | 49.00 |
| BMI | rs12049202 | 1 | 77967523 | T | C | 2.03E-01 | 2.40E-02 | 2.20E-03 | 1.00E-28 | 681275 | 0.017% | 119.01 |
| BMI | rs12602912 | 17 | 65870073 | T | C | 2.05E-01 | 1.76E-02 | 2.10E-03 | 9.90E-18 | 681275 | 0.010% | 70.24 |
| BMI | rs11672660 | 19 | 46180184 | T | C | 2.05E-01 | -3.40E-02 | 2.10E-03 | 1.70E-60 | 681275 | 0.038% | 262.13 |
| BMI | rs7761673 | 6 | 70357368 | A | T | 2.06E-01 | -1.26E-02 | 2.10E-03 | 1.90E-09 | 681275 | 0.005% | 36.00 |
| BMI | rs10971709 | 9 | 33804813 | T | C | 2.06E-01 | 1.32E-02 | 2.10E-03 | 6.20E-10 | 681275 | 0.006% | 39.51 |
| BMI | rs2283093 | 7 | 126721231 | T | C | 2.07E-01 | 1.27E-02 | 2.10E-03 | 3.10E-09 | 681275 | 0.005% | 36.57 |
| BMI | rs11173522 | 12 | 60953472 | A | C | 2.08E-01 | 1.28E-02 | 2.10E-03 | 1.10E-09 | 681275 | 0.005% | 37.15 |
| BMI | rs17113297 | 10 | 102395982 | T | C | 2.08E-01 | 1.66E-02 | 2.10E-03 | 2.10E-15 | 681275 | 0.009% | 62.49 |
| BMI | rs9478671 | 6 | 155987825 | G | A | 2.09E-01 | 1.20E-02 | 2.10E-03 | 1.70E-08 | 681275 | 0.005% | 32.65 |
| BMI | rs11611246 | 12 | 939480 | T | G | 2.10E-01 | 2.40E-02 | 2.00E-03 | 5.00E-32 | 681275 | 0.021% | 144.00 |
| BMI | rs7144011 | 14 | 79940383 | T | G | 2.14E-01 | 2.82E-02 | 2.00E-03 | 5.20E-47 | 681275 | 0.029% | 198.81 |
| BMI | rs12448257 | 16 | 3599655 | A | G | 2.18E-01 | 1.84E-02 | 2.00E-03 | 8.10E-20 | 681275 | 0.012% | 84.64 |
| BMI | rs1465900 | 11 | 76473138 | C | A | 2.19E-01 | -1.25E-02 | 2.00E-03 | 4.80E-10 | 681275 | 0.006% | 39.06 |
| BMI | rs2832283 | 21 | 30690558 | A | G | 2.21E-01 | 1.15E-02 | 2.00E-03 | 5.80E-09 | 681275 | 0.005% | 33.06 |
| BMI | rs3807645 | 7 | 77830091 | A | G | 2.21E-01 | -1.66E-02 | 2.10E-03 | 2.40E-15 | 681275 | 0.009% | 62.49 |
| BMI | rs4660443 | 1 | 39591779 | T | C | 2.22E-01 | 1.64E-02 | 2.10E-03 | 6.80E-15 | 681275 | 0.009% | 60.99 |
| BMI | rs7724675 | 5 | 130440010 | A | G | 2.24E-01 | -1.19E-02 | 2.10E-03 | 9.50E-09 | 681275 | 0.005% | 32.11 |
| BMI | rs11656076 | 17 | 31464270 | A | G | 2.25E-01 | -1.42E-02 | 2.10E-03 | 5.60E-12 | 681275 | 0.007% | 45.72 |
| BMI | rs10492229 | 12 | 110602173 | T | C | 2.27E-01 | 1.42E-02 | 1.90E-03 | 7.70E-14 | 681275 | 0.008% | 55.86 |
| BMI | rs6461115 | 7 | 2103668 | G | A | 2.29E-01 | -1.44E-02 | 1.90E-03 | 1.20E-13 | 681275 | 0.008% | 57.44 |
| BMI | rs12041258 | 1 | 195047936 | C | T | 2.29E-01 | -1.46E-02 | 2.00E-03 | 9.50E-13 | 681275 | 0.008% | 53.29 |
| BMI | rs663129 | 18 | 57838401 | A | G | 2.30E-01 | 5.45E-02 | 1.90E-03 | 1.60E-178 | 681275 | 0.121% | 822.78 |
| BMI | rs13329567 | 15 | 68104367 | T | C | 2.31E-01 | -2.93E-02 | 2.00E-03 | 1.00E-50 | 681275 | 0.031% | 214.62 |
| BMI | rs11170468 | 12 | 39430048 | C | A | 2.33E-01 | -1.23E-02 | 1.90E-03 | 1.90E-10 | 681275 | 0.006% | 41.91 |
| BMI | rs3935648 | 17 | 79085335 | G | C | 2.33E-01 | -1.25E-02 | 2.20E-03 | 6.80E-09 | 681275 | 0.005% | 32.28 |
| BMI | rs11908637 | 20 | 47428485 | A | G | 2.36E-01 | -1.20E-02 | 2.10E-03 | 4.90E-09 | 681275 | 0.005% | 32.65 |
| BMI | rs11945861 | 4 | 65700865 | A | G | 2.37E-01 | -1.48E-02 | 2.00E-03 | 5.00E-13 | 681275 | 0.008% | 54.76 |
| BMI | rs11609659 | 12 | 108296260 | C | T | 2.37E-01 | -1.54E-02 | 2.00E-03 | 2.20E-14 | 681275 | 0.009% | 59.29 |
| BMI | rs17789218 | 6 | 100600097 | C | T | 2.39E-01 | 1.30E-02 | 1.90E-03 | 7.40E-12 | 681275 | 0.007% | 46.81 |
| BMI | rs11115176 | 12 | 82465797 | C | T | 2.40E-01 | -1.21E-02 | 1.90E-03 | 2.00E-10 | 681275 | 0.006% | 40.56 |
| BMI | rs9379827 | 6 | 26153335 | A | C | 2.41E-01 | -1.32E-02 | 1.90E-03 | 6.90E-12 | 681275 | 0.007% | 48.27 |
| BMI | rs2907948 | 7 | 150638484 | A | G | 2.43E-01 | -1.41E-02 | 1.90E-03 | 1.30E-13 | 681275 | 0.008% | 55.07 |
| BMI | rs3902951 | 14 | 69789755 | G | T | 2.46E-01 | 1.34E-02 | 2.00E-03 | 7.00E-12 | 681275 | 0.007% | 44.89 |
| BMI | rs2875762 | 6 | 124925032 | C | G | 2.47E-01 | 1.39E-02 | 2.00E-03 | 1.20E-11 | 681275 | 0.007% | 48.30 |
| BMI | rs11615578 | 12 | 121714935 | T | C | 2.47E-01 | 1.30E-02 | 2.00E-03 | 8.10E-11 | 681275 | 0.006% | 42.25 |
| BMI | rs13287131 | 9 | 92119579 | C | T | 2.49E-01 | 1.23E-02 | 2.00E-03 | 6.80E-10 | 681275 | 0.006% | 37.82 |
| BMI | rs4954638 | 2 | 137435455 | C | A | 2.49E-01 | -1.18E-02 | 2.00E-03 | 2.90E-09 | 681275 | 0.005% | 34.81 |
| BMI | rs16953563 | 15 | 66686770 | A | G | 2.52E-01 | -1.34E-02 | 2.00E-03 | 1.50E-11 | 681275 | 0.007% | 44.89 |
| BMI | rs12888545 | 14 | 88308044 | G | A | 2.52E-01 | 1.36E-02 | 2.00E-03 | 9.10E-12 | 681275 | 0.007% | 46.24 |
| BMI | rs12299814 | 12 | 90216146 | A | C | 2.53E-01 | -1.57E-02 | 2.00E-03 | 5.20E-15 | 681275 | 0.009% | 61.62 |
| BMI | rs2361988 | 16 | 398151 | C | T | 2.54E-01 | -1.55E-02 | 2.00E-03 | 5.20E-15 | 681275 | 0.009% | 60.06 |
| BMI | rs3754963 | 2 | 166185707 | T | A | 2.57E-01 | -1.23E-02 | 2.00E-03 | 3.30E-10 | 681275 | 0.006% | 37.82 |
| BMI | rs11736228 | 4 | 147376805 | T | A | 2.59E-01 | -1.39E-02 | 2.00E-03 | 4.10E-12 | 681275 | 0.007% | 48.30 |
| BMI | rs6561943 | 13 | 58356761 | T | C | 2.60E-01 | 1.19E-02 | 1.90E-03 | 4.20E-10 | 681275 | 0.006% | 39.23 |
| BMI | rs17724992 | 19 | 18454825 | G | A | 2.60E-01 | -1.83E-02 | 1.90E-03 | 1.00E-22 | 681275 | 0.014% | 92.77 |
| BMI | rs17056301 | 5 | 158271680 | C | T | 2.64E-01 | 1.18E-02 | 2.00E-03 | 2.40E-09 | 681275 | 0.005% | 34.81 |
| BMI | rs7488867 | 12 | 103699685 | T | C | 2.64E-01 | -2.04E-02 | 2.00E-03 | 8.40E-24 | 681275 | 0.015% | 104.04 |
| BMI | rs7869771 | 9 | 94180627 | C | A | 2.65E-01 | -1.40E-02 | 1.90E-03 | 4.90E-13 | 681275 | 0.008% | 54.29 |
| BMI | rs11855853 | 15 | 78012618 | T | C | 2.65E-01 | -1.45E-02 | 2.00E-03 | 2.40E-13 | 681275 | 0.008% | 52.56 |
| BMI | rs7599312 | 2 | 213413231 | A | G | 2.65E-01 | -1.86E-02 | 1.90E-03 | 6.90E-24 | 681275 | 0.014% | 95.83 |
| BMI | rs12922346 | 16 | 82438337 | C | G | 2.66E-01 | 1.36E-02 | 2.00E-03 | 1.00E-11 | 681275 | 0.007% | 46.24 |
| BMI | rs12422552 | 12 | 14413931 | C | G | 2.66E-01 | -1.34E-02 | 2.00E-03 | 1.60E-11 | 681275 | 0.007% | 44.89 |
| BMI | rs9650755 | 9 | 96484342 | G | A | 2.66E-01 | 1.54E-02 | 2.00E-03 | 2.80E-15 | 681275 | 0.009% | 59.29 |
| BMI | rs845084 | 10 | 125220036 | A | G | 2.68E-01 | 1.40E-02 | 2.00E-03 | 1.30E-12 | 681275 | 0.007% | 49.00 |
| BMI | rs7844647 | 8 | 34503776 | C | T | 2.68E-01 | -1.23E-02 | 1.80E-03 | 2.80E-11 | 681275 | 0.007% | 46.69 |
| BMI | rs17636031 | 10 | 126594078 | C | T | 2.70E-01 | 1.60E-02 | 1.90E-03 | 1.20E-17 | 681275 | 0.010% | 70.91 |
| BMI | rs6235 | 5 | 95728898 | G | C | 2.70E-01 | 1.75E-02 | 1.90E-03 | 1.50E-19 | 681275 | 0.012% | 84.83 |
| BMI | rs217671 | 14 | 62360464 | G | A | 2.72E-01 | 1.44E-02 | 1.90E-03 | 1.30E-13 | 681275 | 0.008% | 57.44 |
| BMI | rs10408324 | 19 | 51774806 | T | C | 2.74E-01 | -1.24E-02 | 1.90E-03 | 9.50E-11 | 681275 | 0.006% | 42.59 |
| BMI | rs1552893 | 3 | 194851700 | G | A | 2.78E-01 | -1.26E-02 | 1.90E-03 | 8.10E-11 | 681275 | 0.006% | 43.98 |
| BMI | rs4858193 | 3 | 20441050 | C | T | 2.78E-01 | -1.29E-02 | 1.90E-03 | 1.60E-11 | 681275 | 0.007% | 46.10 |
| BMI | rs3806572 | 2 | 55238677 | A | G | 2.79E-01 | -1.45E-02 | 1.90E-03 | 1.60E-14 | 681275 | 0.009% | 58.24 |
| BMI | rs3977755 | 10 | 104420210 | T | C | 2.80E-01 | -1.35E-02 | 1.90E-03 | 5.90E-13 | 681275 | 0.007% | 50.48 |
| BMI | rs999889 | 10 | 84279949 | A | G | 2.82E-01 | -1.08E-02 | 1.90E-03 | 1.40E-08 | 681275 | 0.005% | 32.31 |
| BMI | rs10915840 | 1 | 225668524 | A | G | 2.83E-01 | -1.18E-02 | 1.90E-03 | 1.30E-09 | 681275 | 0.006% | 38.57 |
| BMI | rs12779328 | 10 | 12943973 | T | C | 2.83E-01 | 1.05E-02 | 1.90E-03 | 4.50E-08 | 681275 | 0.004% | 30.54 |
| BMI | rs10197031 | 2 | 105454590 | C | T | 2.83E-01 | 1.66E-02 | 1.90E-03 | 1.90E-18 | 681275 | 0.011% | 76.33 |
| BMI | rs4518345 | 5 | 27185904 | A | G | 2.84E-01 | -1.17E-02 | 1.90E-03 | 1.00E-09 | 681275 | 0.006% | 37.92 |
| BMI | rs1830074 | 7 | 6718674 | C | T | 2.88E-01 | 1.15E-02 | 1.90E-03 | 1.40E-09 | 681275 | 0.005% | 36.63 |
| BMI | rs7334078 | 13 | 99120484 | C | T | 2.88E-01 | -1.21E-02 | 1.90E-03 | 2.20E-10 | 681275 | 0.006% | 40.56 |
| BMI | rs7703576 | 5 | 144543996 | C | T | 2.89E-01 | 1.03E-02 | 1.90E-03 | 4.80E-08 | 681275 | 0.004% | 29.39 |
| BMI | rs8192675 | 3 | 170724883 | C | T | 2.89E-01 | 1.52E-02 | 1.80E-03 | 1.40E-17 | 681275 | 0.010% | 71.31 |
| BMI | rs7970953 | 12 | 24075508 | A | G | 2.90E-01 | 1.35E-02 | 1.80E-03 | 9.80E-14 | 681275 | 0.008% | 56.25 |
| BMI | rs7903146 | 10 | 114758349 | T | C | 2.91E-01 | -1.81E-02 | 1.80E-03 | 1.30E-23 | 681275 | 0.015% | 101.11 |
| BMI | rs7640424 | 3 | 107820063 | T | C | 2.97E-01 | -1.36E-02 | 1.80E-03 | 2.30E-14 | 681275 | 0.008% | 57.09 |
| BMI | rs12593036 | 15 | 81058652 | G | A | 2.99E-01 | -1.54E-02 | 1.90E-03 | 3.80E-16 | 681275 | 0.010% | 65.70 |
| BMI | rs17405819 | 8 | 76806584 | C | T | 3.01E-01 | -2.15E-02 | 1.80E-03 | 4.30E-33 | 681275 | 0.021% | 142.67 |
| BMI | rs4639527 | 2 | 416815 | G | A | 3.01E-01 | 1.72E-02 | 1.90E-03 | 3.30E-20 | 681275 | 0.012% | 81.95 |
| BMI | rs10132280 | 14 | 25928179 | A | C | 3.02E-01 | -2.23E-02 | 1.80E-03 | 5.60E-35 | 681275 | 0.023% | 153.48 |
| BMI | rs9367368 | 6 | 13189275 | C | T | 3.03E-01 | -1.21E-02 | 1.80E-03 | 1.00E-11 | 681275 | 0.007% | 45.19 |
| BMI | rs11185111 | 1 | 107962328 | A | G | 3.04E-01 | -1.29E-02 | 1.90E-03 | 7.70E-12 | 681275 | 0.007% | 46.10 |
| BMI | rs4783830 | 16 | 54255346 | A | G | 3.07E-01 | -1.05E-02 | 1.90E-03 | 2.40E-08 | 681275 | 0.004% | 30.54 |
| BMI | rs10942267 | 5 | 80841914 | G | A | 3.09E-01 | -1.56E-02 | 1.90E-03 | 3.90E-17 | 681275 | 0.010% | 67.41 |
| BMI | rs2306537 | 12 | 133423695 | G | A | 3.09E-01 | 1.33E-02 | 1.90E-03 | 8.70E-13 | 681275 | 0.007% | 49.00 |
| BMI | rs2285178 | 22 | 38205989 | C | T | 3.11E-01 | 1.12E-02 | 1.90E-03 | 9.40E-09 | 681275 | 0.005% | 34.75 |
| BMI | rs7206608 | 16 | 82872628 | G | C | 3.15E-01 | 1.32E-02 | 1.90E-03 | 1.30E-12 | 681275 | 0.007% | 48.27 |
| BMI | rs491711 | 11 | 28742220 | C | A | 3.16E-01 | -1.15E-02 | 1.90E-03 | 1.10E-09 | 681275 | 0.005% | 36.63 |
| BMI | rs2931434 | 5 | 73159098 | T | C | 3.17E-01 | -1.04E-02 | 1.80E-03 | 1.40E-08 | 681275 | 0.005% | 33.38 |
| BMI | rs4937870 | 11 | 112826709 | G | A | 3.17E-01 | -1.09E-02 | 1.90E-03 | 8.80E-09 | 681275 | 0.005% | 32.91 |
| BMI | rs1320903 | 3 | 131758077 | A | G | 3.17E-01 | 2.16E-02 | 1.80E-03 | 9.20E-32 | 681275 | 0.021% | 144.00 |
| BMI | rs1412235 | 9 | 28410996 | C | G | 3.18E-01 | 2.46E-02 | 1.70E-03 | 6.00E-45 | 681275 | 0.031% | 209.40 |
| BMI | rs1538247 | 6 | 153395344 | C | T | 3.18E-01 | 1.08E-02 | 1.90E-03 | 1.00E-08 | 681275 | 0.005% | 32.31 |
| BMI | rs7025938 | 9 | 103088321 | G | C | 3.19E-01 | 1.66E-02 | 1.90E-03 | 3.70E-19 | 681275 | 0.011% | 76.33 |
| BMI | rs12680842 | 8 | 95582606 | G | A | 3.21E-01 | -1.33E-02 | 1.80E-03 | 4.40E-14 | 681275 | 0.008% | 54.60 |
| BMI | rs4968656 | 17 | 61616959 | G | A | 3.22E-01 | 1.16E-02 | 1.90E-03 | 8.20E-10 | 681275 | 0.005% | 37.27 |
| BMI | rs7819514 | 8 | 93204442 | A | G | 3.22E-01 | -1.07E-02 | 1.80E-03 | 5.70E-09 | 681275 | 0.005% | 35.34 |
| BMI | rs538579 | 3 | 62711674 | C | G | 3.23E-01 | 1.37E-02 | 1.90E-03 | 1.30E-13 | 681275 | 0.008% | 51.99 |
| BMI | rs17513613 | 19 | 30286822 | C | T | 3.24E-01 | 1.86E-02 | 1.80E-03 | 3.60E-26 | 681275 | 0.016% | 106.78 |
| BMI | rs1371108 | 2 | 81816251 | A | C | 3.25E-01 | 1.19E-02 | 1.80E-03 | 9.00E-11 | 681275 | 0.006% | 43.71 |
| BMI | rs12936083 | 17 | 4801887 | G | A | 3.27E-01 | 1.39E-02 | 1.90E-03 | 4.10E-13 | 681275 | 0.008% | 53.52 |
| BMI | rs17311369 | 15 | 47709199 | T | C | 3.28E-01 | -1.04E-02 | 1.90E-03 | 3.10E-08 | 681275 | 0.004% | 29.96 |
| BMI | rs9571687 | 13 | 67472713 | A | C | 3.29E-01 | -1.29E-02 | 1.80E-03 | 2.80E-12 | 681275 | 0.008% | 51.36 |
| BMI | rs705704 | 12 | 56435412 | A | G | 3.30E-01 | -1.31E-02 | 1.80E-03 | 1.90E-13 | 681275 | 0.008% | 52.97 |
| BMI | rs8090983 | 18 | 52586691 | G | A | 3.31E-01 | 1.18E-02 | 1.80E-03 | 2.00E-10 | 681275 | 0.006% | 42.98 |
| BMI | rs17710386 | 18 | 63461201 | C | T | 3.32E-01 | 1.26E-02 | 1.80E-03 | 1.00E-12 | 681275 | 0.007% | 49.00 |
| BMI | rs2162524 | 2 | 230817437 | C | T | 3.32E-01 | 1.55E-02 | 1.80E-03 | 4.10E-17 | 681275 | 0.011% | 74.15 |
| BMI | rs7084454 | 10 | 21821274 | A | G | 3.35E-01 | 1.93E-02 | 1.90E-03 | 4.00E-25 | 681275 | 0.015% | 103.18 |
| BMI | rs3800637 | 7 | 137403432 | C | T | 3.36E-01 | 1.15E-02 | 1.80E-03 | 5.10E-10 | 681275 | 0.006% | 40.82 |
| BMI | rs2820311 | 1 | 201841476 | G | A | 3.37E-01 | 2.35E-02 | 1.80E-03 | 4.10E-38 | 681275 | 0.025% | 170.45 |
| BMI | rs6772756 | 3 | 182312152 | G | A | 3.37E-01 | -1.04E-02 | 1.90E-03 | 4.00E-08 | 681275 | 0.004% | 29.96 |
| BMI | rs7998796 | 13 | 81020036 | G | A | 3.37E-01 | 1.05E-02 | 1.80E-03 | 1.10E-08 | 681275 | 0.005% | 34.03 |
| BMI | rs2744974 | 6 | 34579431 | T | C | 3.38E-01 | 2.49E-02 | 1.80E-03 | 1.40E-45 | 681275 | 0.028% | 191.36 |
| BMI | rs12905439 | 15 | 99521883 | G | C | 3.39E-01 | -1.18E-02 | 1.80E-03 | 1.40E-10 | 681275 | 0.006% | 42.98 |
| BMI | rs7626079 | 3 | 66427259 | T | C | 3.43E-01 | 1.10E-02 | 1.80E-03 | 1.60E-09 | 681275 | 0.005% | 37.35 |
| BMI | rs7102454 | 11 | 65594820 | C | T | 3.44E-01 | 1.58E-02 | 1.80E-03 | 2.40E-18 | 681275 | 0.011% | 77.05 |
| BMI | rs7222349 | 17 | 42304644 | A | G | 3.44E-01 | 1.15E-02 | 1.80E-03 | 3.30E-10 | 681275 | 0.006% | 40.82 |
| BMI | rs1863652 | 4 | 95991417 | A | G | 3.45E-01 | -1.15E-02 | 1.80E-03 | 1.40E-10 | 681275 | 0.006% | 40.82 |
| BMI | rs13132853 | 4 | 38680015 | G | A | 3.45E-01 | -1.42E-02 | 1.80E-03 | 4.70E-15 | 681275 | 0.009% | 62.23 |
| BMI | rs2235564 | 1 | 6713114 | T | C | 3.47E-01 | 1.31E-02 | 1.80E-03 | 3.70E-13 | 681275 | 0.008% | 52.97 |
| BMI | rs13263601 | 8 | 14095900 | C | A | 3.48E-01 | 1.54E-02 | 1.80E-03 | 2.20E-17 | 681275 | 0.011% | 73.20 |
| BMI | rs2228213 | 6 | 12124855 | A | G | 3.48E-01 | -1.39E-02 | 1.70E-03 | 4.60E-16 | 681275 | 0.010% | 66.85 |
| BMI | rs1330052 | 13 | 86536006 | G | C | 3.50E-01 | 1.32E-02 | 1.80E-03 | 1.50E-13 | 681275 | 0.008% | 53.78 |
| BMI | rs1804528 | 4 | 146056320 | A | G | 3.51E-01 | 1.09E-02 | 2.00E-03 | 3.00E-08 | 681275 | 0.004% | 29.70 |
| BMI | rs1266874 | 6 | 51779638 | G | A | 3.56E-01 | 1.40E-02 | 1.80E-03 | 9.80E-15 | 681275 | 0.009% | 60.49 |
| BMI | rs13147390 | 4 | 80712000 | C | T | 3.57E-01 | 1.03E-02 | 1.80E-03 | 1.00E-08 | 681275 | 0.005% | 32.74 |
| BMI | rs2836964 | 21 | 40631006 | C | T | 3.58E-01 | -1.10E-02 | 1.80E-03 | 1.30E-09 | 681275 | 0.005% | 37.35 |
| BMI | rs3829849 | 9 | 129390800 | T | C | 3.59E-01 | 9.80E-03 | 1.70E-03 | 5.90E-09 | 681275 | 0.005% | 33.23 |
| BMI | rs6764533 | 3 | 196088464 | A | G | 3.59E-01 | 1.16E-02 | 1.80E-03 | 1.40E-10 | 681275 | 0.006% | 41.53 |
| BMI | rs10169594 | 2 | 41637688 | C | T | 3.60E-01 | 1.21E-02 | 1.80E-03 | 2.00E-11 | 681275 | 0.007% | 45.19 |
| BMI | rs6556301 | 5 | 176527577 | T | G | 3.60E-01 | -1.11E-02 | 1.80E-03 | 4.10E-10 | 681275 | 0.006% | 38.03 |
| BMI | rs7780752 | 7 | 93241640 | C | T | 3.60E-01 | 1.39E-02 | 1.80E-03 | 1.00E-14 | 681275 | 0.009% | 59.63 |
| BMI | rs2009416 | 5 | 92415111 | T | C | 3.61E-01 | -1.21E-02 | 1.80E-03 | 1.10E-11 | 681275 | 0.007% | 45.19 |
| BMI | rs2065418 | 11 | 30422068 | G | T | 3.62E-01 | -1.66E-02 | 1.80E-03 | 3.60E-20 | 681275 | 0.012% | 85.05 |
| BMI | rs705217 | 1 | 34581472 | G | T | 3.65E-01 | -1.02E-02 | 1.80E-03 | 9.30E-09 | 681275 | 0.005% | 32.11 |
| BMI | rs17424296 | 5 | 60838903 | A | G | 3.66E-01 | -1.08E-02 | 1.80E-03 | 2.40E-09 | 681275 | 0.005% | 36.00 |
| BMI | rs3772882 | 3 | 81808602 | A | C | 3.66E-01 | 1.27E-02 | 1.80E-03 | 6.60E-13 | 681275 | 0.007% | 49.78 |
| BMI | rs200810 | 6 | 97922184 | C | T | 3.72E-01 | -1.36E-02 | 1.70E-03 | 5.50E-16 | 681275 | 0.009% | 64.00 |
| BMI | rs2124499 | 3 | 123093541 | C | G | 3.72E-01 | -1.23E-02 | 1.70E-03 | 3.40E-13 | 681275 | 0.008% | 52.35 |
| BMI | rs6593688 | 1 | 96322205 | G | A | 3.73E-01 | 1.37E-02 | 1.80E-03 | 8.60E-15 | 681275 | 0.009% | 57.93 |
| BMI | rs7037266 | 9 | 6942940 | A | C | 3.74E-01 | -1.12E-02 | 1.80E-03 | 3.50E-10 | 681275 | 0.006% | 38.72 |
| BMI | rs7535528 | 1 | 2444414 | A | G | 3.74E-01 | -1.52E-02 | 1.80E-03 | 1.40E-16 | 681275 | 0.010% | 71.31 |
| BMI | rs1948080 | 9 | 11852043 | G | T | 3.75E-01 | -1.37E-02 | 1.80E-03 | 1.10E-14 | 681275 | 0.009% | 57.93 |
| BMI | rs1891216 | 1 | 7728391 | G | T | 3.76E-01 | 1.07E-02 | 1.80E-03 | 2.40E-09 | 681275 | 0.005% | 35.34 |
| BMI | rs1158805 | 18 | 40736590 | A | C | 3.77E-01 | -1.37E-02 | 1.80E-03 | 1.20E-14 | 681275 | 0.009% | 57.93 |
| BMI | rs13047416 | 21 | 40309436 | G | C | 3.77E-01 | -1.54E-02 | 1.80E-03 | 2.20E-17 | 681275 | 0.011% | 73.20 |
| BMI | rs1472169 | 9 | 37209396 | T | C | 3.77E-01 | -1.39E-02 | 1.80E-03 | 2.80E-15 | 681275 | 0.009% | 59.63 |
| BMI | rs7138803 | 12 | 50247468 | A | G | 3.77E-01 | 3.00E-02 | 1.70E-03 | 2.30E-71 | 681275 | 0.046% | 311.42 |
| BMI | rs331966 | 4 | 143675717 | C | A | 3.79E-01 | 1.12E-02 | 1.80E-03 | 3.20E-10 | 681275 | 0.006% | 38.72 |
| BMI | rs11105839 | 12 | 91237920 | A | T | 3.80E-01 | -1.09E-02 | 1.70E-03 | 1.10E-10 | 681275 | 0.006% | 41.11 |
| BMI | rs4952843 | 2 | 46957845 | G | A | 3.81E-01 | -1.31E-02 | 1.80E-03 | 6.80E-14 | 681275 | 0.008% | 52.97 |
| BMI | rs709400 | 14 | 104149475 | G | A | 3.82E-01 | -1.50E-02 | 1.70E-03 | 4.60E-19 | 681275 | 0.011% | 77.85 |
| BMI | rs10811871 | 9 | 23200766 | G | A | 3.83E-01 | -1.08E-02 | 1.80E-03 | 1.60E-09 | 681275 | 0.005% | 36.00 |
| BMI | rs12033257 | 1 | 112318484 | G | A | 3.84E-01 | -1.46E-02 | 1.80E-03 | 2.40E-15 | 681275 | 0.010% | 65.79 |
| BMI | rs9547153 | 13 | 85903717 | G | A | 3.84E-01 | 9.80E-03 | 1.70E-03 | 8.70E-09 | 681275 | 0.005% | 33.23 |
| BMI | rs2325036 | 3 | 85819412 | C | A | 3.85E-01 | -1.81E-02 | 1.70E-03 | 3.60E-27 | 681275 | 0.017% | 113.36 |
| BMI | rs1327259 | 6 | 51177811 | G | A | 3.87E-01 | -1.55E-02 | 1.80E-03 | 1.70E-18 | 681275 | 0.011% | 74.15 |
| BMI | rs1836303 | 15 | 46539116 | G | A | 3.87E-01 | 1.16E-02 | 1.80E-03 | 5.30E-11 | 681275 | 0.006% | 41.53 |
| BMI | rs10750215 | 11 | 122505344 | T | G | 3.88E-01 | 1.08E-02 | 1.70E-03 | 1.30E-10 | 681275 | 0.006% | 40.36 |
| BMI | rs10269783 | 7 | 49616203 | A | G | 3.90E-01 | 1.33E-02 | 1.70E-03 | 1.40E-15 | 681275 | 0.009% | 61.21 |
| BMI | rs326896 | 4 | 112669571 | T | C | 3.93E-01 | -1.28E-02 | 1.80E-03 | 2.80E-13 | 681275 | 0.007% | 50.57 |
| BMI | rs1681740 | 10 | 118564313 | C | A | 3.93E-01 | -1.15E-02 | 1.80E-03 | 1.10E-10 | 681275 | 0.006% | 40.82 |
| BMI | rs10243319 | 7 | 147674678 | C | T | 3.94E-01 | -1.07E-02 | 1.80E-03 | 1.20E-09 | 681275 | 0.005% | 35.34 |
| BMI | rs11951673 | 5 | 95861012 | T | C | 3.94E-01 | -1.23E-02 | 1.70E-03 | 1.10E-13 | 681275 | 0.008% | 52.35 |
| BMI | rs175165 | 22 | 20116015 | G | T | 3.94E-01 | -1.03E-02 | 1.80E-03 | 5.20E-09 | 681275 | 0.005% | 32.74 |
| BMI | rs559231 | 18 | 39644247 | T | G | 3.96E-01 | 1.35E-02 | 1.80E-03 | 2.40E-14 | 681275 | 0.008% | 56.25 |
| BMI | rs2307111 | 5 | 75003678 | C | T | 3.96E-01 | -2.65E-02 | 1.60E-03 | 1.60E-58 | 681275 | 0.040% | 274.32 |
| BMI | rs8027205 | 15 | 98280959 | G | C | 3.97E-01 | -1.08E-02 | 1.80E-03 | 1.40E-09 | 681275 | 0.005% | 36.00 |
| BMI | rs6785245 | 3 | 82647990 | C | T | 3.97E-01 | 1.32E-02 | 1.70E-03 | 4.00E-14 | 681275 | 0.009% | 60.29 |
| BMI | rs756717 | 16 | 72996162 | A | G | 3.97E-01 | -1.48E-02 | 1.70E-03 | 5.40E-18 | 681275 | 0.011% | 75.79 |
| BMI | rs901630 | 6 | 98539519 | T | C | 3.97E-01 | -1.46E-02 | 1.70E-03 | 1.90E-18 | 681275 | 0.011% | 73.76 |
| BMI | rs12718572 | 7 | 50573325 | T | C | 4.02E-01 | -1.17E-02 | 1.80E-03 | 3.00E-11 | 681275 | 0.006% | 42.25 |
| BMI | rs1784460 | 11 | 118938371 | A | T | 4.04E-01 | 1.32E-02 | 1.80E-03 | 9.00E-14 | 681275 | 0.008% | 53.78 |
| BMI | rs7498665 | 16 | 28883241 | G | A | 4.04E-01 | 2.71E-02 | 1.70E-03 | 5.60E-60 | 681275 | 0.037% | 254.12 |
| BMI | rs12150665 | 17 | 34914787 | C | T | 4.06E-01 | -1.62E-02 | 1.70E-03 | 1.60E-22 | 681275 | 0.013% | 90.81 |
| BMI | rs2425840 | 20 | 44904838 | C | A | 4.06E-01 | 1.19E-02 | 1.80E-03 | 1.60E-11 | 681275 | 0.006% | 43.71 |
| BMI | rs13110266 | 4 | 162129844 | A | G | 4.07E-01 | -1.17E-02 | 1.70E-03 | 1.90E-12 | 681275 | 0.007% | 47.37 |
| BMI | rs2174307 | 9 | 73791849 | C | G | 4.07E-01 | 1.21E-02 | 1.70E-03 | 4.90E-12 | 681275 | 0.007% | 50.66 |
| BMI | rs1624134 | 10 | 34834482 | C | G | 4.07E-01 | 1.01E-02 | 1.80E-03 | 1.10E-08 | 681275 | 0.005% | 31.48 |
| BMI | rs4148866 | 12 | 123425575 | T | C | 4.07E-01 | 9.80E-03 | 1.80E-03 | 4.00E-08 | 681275 | 0.004% | 29.64 |
| BMI | rs2861683 | 2 | 67836507 | C | A | 4.07E-01 | -1.44E-02 | 1.70E-03 | 1.30E-16 | 681275 | 0.011% | 71.75 |
| BMI | rs355777 | 3 | 154034950 | C | G | 4.11E-01 | 1.53E-02 | 1.70E-03 | 1.40E-18 | 681275 | 0.012% | 81.00 |
| BMI | rs1927790 | 13 | 96922191 | C | T | 4.11E-01 | 1.48E-02 | 1.60E-03 | 1.80E-19 | 681275 | 0.013% | 85.56 |
| BMI | rs17207196 | 7 | 75101065 | T | C | 4.12E-01 | -2.21E-02 | 1.80E-03 | 2.10E-35 | 681275 | 0.022% | 150.74 |
| BMI | rs7124681 | 11 | 47529947 | A | C | 4.13E-01 | 2.63E-02 | 1.60E-03 | 3.20E-58 | 681275 | 0.040% | 270.19 |
| BMI | rs9538162 | 13 | 59265043 | C | T | 4.14E-01 | -1.56E-02 | 1.80E-03 | 4.80E-19 | 681275 | 0.011% | 75.11 |
| BMI | rs2365389 | 3 | 61236462 | T | C | 4.14E-01 | -1.74E-02 | 1.70E-03 | 1.30E-25 | 681275 | 0.015% | 104.76 |
| BMI | rs9522285 | 13 | 112230701 | A | G | 4.14E-01 | 1.27E-02 | 1.70E-03 | 2.50E-13 | 681275 | 0.008% | 55.81 |
| BMI | rs1048932 | 11 | 115044850 | A | C | 4.16E-01 | -1.60E-02 | 1.70E-03 | 3.80E-22 | 681275 | 0.013% | 88.58 |
| BMI | rs2429150 | 12 | 2152655 | C | A | 4.16E-01 | 1.11E-02 | 1.80E-03 | 2.70E-10 | 681275 | 0.006% | 38.03 |
| BMI | rs3749897 | 6 | 42532102 | T | C | 4.17E-01 | 1.22E-02 | 1.80E-03 | 8.40E-12 | 681275 | 0.007% | 45.94 |
| BMI | rs11066188 | 12 | 112610714 | A | G | 4.18E-01 | -1.20E-02 | 1.70E-03 | 8.10E-13 | 681275 | 0.007% | 49.83 |
| BMI | rs768840 | 14 | 73143457 | A | G | 4.18E-01 | 1.14E-02 | 1.80E-03 | 2.00E-10 | 681275 | 0.006% | 40.11 |
| BMI | rs11496125 | 7 | 103417557 | T | C | 4.21E-01 | 1.69E-02 | 1.70E-03 | 3.00E-22 | 681275 | 0.015% | 98.83 |
| BMI | rs12328930 | 2 | 175079125 | C | T | 4.24E-01 | 9.80E-03 | 1.70E-03 | 1.80E-08 | 681275 | 0.005% | 33.23 |
| BMI | rs889398 | 16 | 69556715 | T | C | 4.25E-01 | -1.96E-02 | 1.60E-03 | 1.30E-32 | 681275 | 0.022% | 150.06 |
| BMI | rs10914462 | 1 | 32125943 | G | A | 4.26E-01 | -1.12E-02 | 1.70E-03 | 1.50E-10 | 681275 | 0.006% | 43.40 |
| BMI | rs273504 | 19 | 18215247 | G | A | 4.27E-01 | 1.53E-02 | 1.80E-03 | 4.40E-18 | 681275 | 0.011% | 72.25 |
| BMI | rs1884389 | 20 | 1410582 | T | C | 4.29E-01 | -1.03E-02 | 1.70E-03 | 4.00E-09 | 681275 | 0.005% | 36.71 |
| BMI | rs1430387 | 18 | 58227112 | C | T | 4.30E-01 | -1.14E-02 | 1.70E-03 | 5.80E-11 | 681275 | 0.007% | 44.97 |
| BMI | rs10938397 | 4 | 45182527 | G | A | 4.32E-01 | 3.24E-02 | 1.60E-03 | 3.40E-86 | 681275 | 0.060% | 410.06 |
| BMI | rs12939549 | 17 | 78611724 | G | A | 4.34E-01 | -1.80E-02 | 1.60E-03 | 2.70E-28 | 681275 | 0.019% | 126.56 |
| BMI | rs10768994 | 11 | 43936945 | C | T | 4.34E-01 | -1.14E-02 | 1.70E-03 | 6.40E-12 | 681275 | 0.007% | 44.97 |
| BMI | rs13184896 | 5 | 122734005 | T | G | 4.35E-01 | -1.33E-02 | 1.60E-03 | 3.30E-16 | 681275 | 0.010% | 69.10 |
| BMI | rs10478110 | 5 | 112445734 | C | A | 4.35E-01 | 1.00E-02 | 1.70E-03 | 9.60E-09 | 681275 | 0.005% | 34.60 |
| BMI | rs7694732 | 4 | 115124089 | G | A | 4.38E-01 | -9.90E-03 | 1.70E-03 | 8.70E-09 | 681275 | 0.005% | 33.91 |
| BMI | rs2481665 | 1 | 62594677 | C | T | 4.41E-01 | -1.61E-02 | 1.60E-03 | 7.20E-23 | 681275 | 0.015% | 101.25 |
| BMI | rs2693826 | 2 | 6160943 | A | G | 4.42E-01 | -1.37E-02 | 1.70E-03 | 2.00E-15 | 681275 | 0.010% | 64.94 |
| BMI | rs4936175 | 11 | 132641959 | C | T | 4.45E-01 | 1.22E-02 | 1.70E-03 | 1.40E-12 | 681275 | 0.008% | 51.50 |
| BMI | rs7788008 | 7 | 112972483 | A | G | 4.45E-01 | -1.57E-02 | 1.70E-03 | 1.10E-19 | 681275 | 0.013% | 85.29 |
| BMI | rs1928295 | 9 | 120378483 | C | T | 4.46E-01 | -1.41E-02 | 1.60E-03 | 5.40E-18 | 681275 | 0.011% | 77.66 |
| BMI | rs9615905 | 22 | 48875699 | T | C | 4.50E-01 | 1.10E-02 | 1.70E-03 | 2.70E-10 | 681275 | 0.006% | 41.87 |
| BMI | rs7983065 | 13 | 33380786 | T | C | 4.50E-01 | -1.48E-02 | 1.70E-03 | 8.90E-18 | 681275 | 0.011% | 75.79 |
| BMI | rs4740619 | 9 | 15634326 | C | T | 4.52E-01 | -1.86E-02 | 1.60E-03 | 2.30E-30 | 681275 | 0.020% | 135.14 |
| BMI | rs2608703 | 12 | 41846769 | A | C | 4.55E-01 | 1.42E-02 | 1.70E-03 | 1.90E-16 | 681275 | 0.010% | 69.77 |
| BMI | rs825688 | 16 | 73595718 | T | C | 4.56E-01 | -9.50E-03 | 1.70E-03 | 4.70E-08 | 681275 | 0.005% | 31.23 |
| BMI | rs4307239 | 7 | 24354300 | G | A | 4.58E-01 | 1.15E-02 | 1.70E-03 | 3.90E-11 | 681275 | 0.007% | 45.76 |
| BMI | rs9408882 | 9 | 118664402 | A | G | 4.59E-01 | -9.30E-03 | 1.60E-03 | 1.30E-08 | 681275 | 0.005% | 33.79 |
| BMI | rs4358081 | 2 | 29100642 | C | A | 4.63E-01 | 9.70E-03 | 1.70E-03 | 1.50E-08 | 681275 | 0.005% | 32.56 |
| BMI | rs7685048 | 4 | 95027784 | T | C | 4.65E-01 | -1.01E-02 | 1.70E-03 | 4.10E-09 | 681275 | 0.005% | 35.30 |
| BMI | rs10968114 | 9 | 27800007 | C | A | 4.68E-01 | -1.13E-02 | 1.70E-03 | 6.10E-11 | 681275 | 0.006% | 44.18 |
| BMI | rs4986044 | 17 | 21261560 | T | C | 4.69E-01 | -1.64E-02 | 1.60E-03 | 3.30E-23 | 681275 | 0.015% | 105.06 |
| BMI | rs4072917 | 8 | 143300279 | A | G | 4.69E-01 | 1.15E-02 | 1.80E-03 | 6.90E-11 | 681275 | 0.006% | 40.82 |
| BMI | rs3007105 | 14 | 47367616 | T | C | 4.70E-01 | 1.42E-02 | 1.70E-03 | 1.10E-17 | 681275 | 0.010% | 69.77 |
| BMI | rs11118308 | 1 | 219633869 | G | A | 4.70E-01 | -1.01E-02 | 1.60E-03 | 4.80E-10 | 681275 | 0.006% | 39.85 |
| BMI | rs7172627 | 15 | 31877690 | G | A | 4.72E-01 | 1.17E-02 | 1.70E-03 | 1.10E-11 | 681275 | 0.007% | 47.37 |
| BMI | rs9294260 | 6 | 83433228 | A | G | 4.73E-01 | 1.47E-02 | 1.60E-03 | 1.80E-19 | 681275 | 0.012% | 84.41 |
| BMI | rs2163188 | 10 | 65314711 | C | G | 4.74E-01 | 1.31E-02 | 1.70E-03 | 2.00E-14 | 681275 | 0.009% | 59.38 |
| BMI | rs806600 | 5 | 172914939 | G | A | 4.75E-01 | -9.50E-03 | 1.70E-03 | 3.30E-08 | 681275 | 0.005% | 31.23 |
| BMI | rs10182181 | 2 | 25150296 | G | A | 4.75E-01 | 3.25E-02 | 1.60E-03 | 6.70E-90 | 681275 | 0.061% | 412.60 |
| BMI | rs3814883 | 16 | 29994922 | T | C | 4.76E-01 | 2.32E-02 | 1.70E-03 | 1.10E-40 | 681275 | 0.027% | 186.24 |
| BMI | rs1982725 | 19 | 30618771 | T | C | 4.78E-01 | 9.70E-03 | 1.70E-03 | 3.30E-08 | 681275 | 0.005% | 32.56 |
| BMI | rs1268065 | 6 | 126042783 | A | G | 4.79E-01 | -1.02E-02 | 1.70E-03 | 1.00E-09 | 681275 | 0.005% | 36.00 |
| BMI | rs902695 | 2 | 113955074 | A | G | 4.80E-01 | -1.03E-02 | 1.70E-03 | 2.20E-09 | 681275 | 0.005% | 36.71 |
| BMI | rs11880870 | 19 | 18830704 | G | A | 4.80E-01 | -1.89E-02 | 1.70E-03 | 1.00E-28 | 681275 | 0.018% | 123.60 |
| BMI | rs10733051 | 1 | 167280354 | G | A | 4.80E-01 | -9.70E-03 | 1.60E-03 | 2.90E-09 | 681275 | 0.005% | 36.75 |
| BMI | rs765875 | 6 | 143185683 | T | C | 4.81E-01 | -1.21E-02 | 1.70E-03 | 3.00E-12 | 681275 | 0.007% | 50.66 |
| BMI | rs138289 | 22 | 32182708 | T | A | 4.83E-01 | -1.03E-02 | 1.70E-03 | 3.30E-09 | 681275 | 0.005% | 36.71 |
| BMI | rs7239575 | 18 | 21120035 | C | T | 4.83E-01 | -2.02E-02 | 1.70E-03 | 7.40E-32 | 681275 | 0.021% | 141.19 |
| BMI | rs1503526 | 5 | 63020706 | C | T | 4.84E-01 | 1.40E-02 | 1.70E-03 | 5.50E-17 | 681275 | 0.010% | 67.82 |
| BMI | rs486359 | 6 | 160774441 | C | G | 4.85E-01 | 1.12E-02 | 1.70E-03 | 1.60E-11 | 681275 | 0.006% | 43.40 |
| BMI | rs16871902 | 5 | 3488462 | A | G | 4.88E-01 | 1.25E-02 | 1.70E-03 | 4.60E-13 | 681275 | 0.008% | 54.07 |
| BMI | rs2605603 | 11 | 93221105 | A | G | 4.89E-01 | -1.03E-02 | 1.60E-03 | 2.50E-10 | 681275 | 0.006% | 41.44 |
| BMI | rs10887578 | 10 | 88096047 | C | G | 4.90E-01 | 1.28E-02 | 1.70E-03 | 1.60E-13 | 681275 | 0.008% | 56.69 |
| BMI | rs38314 | 7 | 70067315 | A | G | 4.91E-01 | -1.20E-02 | 1.70E-03 | 4.70E-12 | 681275 | 0.007% | 49.83 |
| BMI | rs2367112 | 5 | 64168193 | G | T | 4.92E-01 | -1.19E-02 | 1.60E-03 | 2.30E-13 | 681275 | 0.008% | 55.32 |
| BMI | rs1064213 | 2 | 198950240 | A | G | 4.92E-01 | 1.20E-02 | 1.70E-03 | 2.40E-12 | 681275 | 0.007% | 49.83 |
| BMI | rs8036040 | 15 | 36402716 | A | C | 4.93E-01 | 1.09E-02 | 1.70E-03 | 2.70E-10 | 681275 | 0.006% | 41.11 |
| BMI | rs1492767 | 4 | 55221467 | T | C | 4.96E-01 | 9.40E-03 | 1.60E-03 | 1.00E-08 | 681275 | 0.005% | 34.52 |
| BMI | rs6011457 | 20 | 61530915 | A | T | 4.98E-01 | -1.16E-02 | 1.70E-03 | 2.70E-11 | 681275 | 0.007% | 46.56 |
| BMI | rs349088 | 11 | 84814393 | A | C | 4.98E-01 | -1.28E-02 | 1.70E-03 | 1.80E-13 | 681275 | 0.008% | 56.69 |
| BMI | rs12044597 | 1 | 1708801 | G | A | 5.03E-01 | 1.43E-02 | 1.60E-03 | 1.70E-18 | 681275 | 0.012% | 79.88 |
| BMI | rs8047395 | 16 | 53798523 | A | G | 5.06E-01 | 6.42E-02 | 1.70E-03 | 1.00E-200 | 681275 | 0.209% | 1426.17 |
| BMI | rs7196720 | 16 | 24534662 | C | T | 5.07E-01 | -1.29E-02 | 1.70E-03 | 7.30E-14 | 681275 | 0.008% | 57.58 |
| BMI | rs11713193 | 3 | 49924424 | A | G | 5.07E-01 | 2.39E-02 | 1.70E-03 | 2.40E-44 | 681275 | 0.029% | 197.65 |
| BMI | rs2479958 | 13 | 111984244 | G | A | 5.08E-01 | -1.54E-02 | 1.80E-03 | 1.50E-17 | 681275 | 0.011% | 73.20 |
| BMI | rs1843328 | 12 | 17111188 | A | C | 5.09E-01 | -9.90E-03 | 1.70E-03 | 7.90E-09 | 681275 | 0.005% | 33.91 |
| BMI | rs12416812 | 11 | 888632 | A | G | 5.09E-01 | 1.11E-02 | 1.60E-03 | 6.10E-12 | 681275 | 0.007% | 48.13 |
| BMI | rs4813619 | 20 | 2815715 | T | G | 5.10E-01 | -1.08E-02 | 1.80E-03 | 2.30E-09 | 681275 | 0.005% | 36.00 |
| BMI | rs7925214 | 11 | 130794253 | T | C | 5.13E-01 | 1.47E-02 | 1.80E-03 | 4.40E-17 | 681275 | 0.010% | 66.69 |
| BMI | rs10248136 | 7 | 39077397 | T | C | 5.14E-01 | -9.70E-03 | 1.70E-03 | 2.00E-08 | 681275 | 0.005% | 32.56 |
| BMI | rs16849710 | 1 | 202106797 | G | A | 5.15E-01 | -1.16E-02 | 1.80E-03 | 6.00E-11 | 681275 | 0.006% | 41.53 |
| BMI | rs9783858 | 18 | 42534584 | T | C | 5.19E-01 | 9.10E-03 | 1.70E-03 | 3.30E-08 | 681275 | 0.004% | 28.65 |
| BMI | rs9362662 | 6 | 90296588 | G | A | 5.20E-01 | -1.12E-02 | 1.70E-03 | 1.20E-10 | 681275 | 0.006% | 43.40 |
| BMI | rs3844598 | 5 | 140992235 | G | A | 5.21E-01 | 9.50E-03 | 1.70E-03 | 3.80E-08 | 681275 | 0.005% | 31.23 |
| BMI | rs1454687 | 3 | 94038085 | G | C | 5.23E-01 | -2.02E-02 | 1.70E-03 | 5.20E-32 | 681275 | 0.021% | 141.19 |
| BMI | rs2284746 | 1 | 17306675 | G | C | 5.24E-01 | -1.04E-02 | 1.70E-03 | 1.40E-09 | 681275 | 0.005% | 37.43 |
| BMI | rs4851029 | 2 | 104159785 | G | T | 5.25E-01 | 1.21E-02 | 1.70E-03 | 1.70E-12 | 681275 | 0.007% | 50.66 |
| BMI | rs6841761 | 4 | 25423538 | T | G | 5.25E-01 | -1.31E-02 | 1.60E-03 | 6.40E-16 | 681275 | 0.010% | 67.03 |
| BMI | rs4906908 | 15 | 27040082 | G | T | 5.25E-01 | 1.03E-02 | 1.70E-03 | 2.50E-09 | 681275 | 0.005% | 36.71 |
| BMI | rs6595205 | 5 | 119372533 | G | C | 5.31E-01 | -1.14E-02 | 1.60E-03 | 2.00E-12 | 681275 | 0.007% | 50.77 |
| BMI | rs1521527 | 2 | 165427825 | C | G | 5.32E-01 | -1.21E-02 | 1.70E-03 | 3.10E-12 | 681275 | 0.007% | 50.66 |
| BMI | rs12981256 | 19 | 1865901 | A | G | 5.33E-01 | 1.42E-02 | 1.80E-03 | 1.10E-15 | 681275 | 0.009% | 62.23 |
| BMI | rs6985109 | 8 | 10761585 | A | G | 5.34E-01 | -1.77E-02 | 1.70E-03 | 1.50E-26 | 681275 | 0.016% | 108.40 |
| BMI | rs7519259 | 1 | 66434743 | A | G | 5.36E-01 | 1.25E-02 | 1.70E-03 | 3.80E-13 | 681275 | 0.008% | 54.07 |
| BMI | rs1896767 | 16 | 62838304 | A | G | 5.38E-01 | -1.09E-02 | 1.70E-03 | 2.40E-10 | 681275 | 0.006% | 41.11 |
| BMI | rs7683836 | 4 | 180167906 | A | G | 5.41E-01 | -1.14E-02 | 1.70E-03 | 6.30E-11 | 681275 | 0.007% | 44.97 |
| BMI | rs1421334 | 8 | 30865733 | C | A | 5.43E-01 | -1.25E-02 | 1.80E-03 | 1.00E-12 | 681275 | 0.007% | 48.23 |
| BMI | rs6815910 | 4 | 55495793 | A | T | 5.44E-01 | -1.28E-02 | 1.70E-03 | 1.40E-13 | 681275 | 0.008% | 56.69 |
| BMI | rs3736485 | 15 | 51748610 | G | A | 5.44E-01 | -1.34E-02 | 1.60E-03 | 2.50E-16 | 681275 | 0.010% | 70.14 |
| BMI | rs2643452 | 4 | 18529220 | A | T | 5.45E-01 | 1.36E-02 | 1.70E-03 | 4.70E-15 | 681275 | 0.009% | 64.00 |
| BMI | rs9845966 | 3 | 13433158 | G | T | 5.48E-01 | -1.05E-02 | 1.70E-03 | 2.50E-10 | 681275 | 0.006% | 38.15 |
| BMI | rs17399237 | 2 | 35471626 | C | T | 5.50E-01 | -1.29E-02 | 1.70E-03 | 6.70E-14 | 681275 | 0.008% | 57.58 |
| BMI | rs2423668 | 20 | 12430673 | C | T | 5.51E-01 | -1.05E-02 | 1.90E-03 | 2.80E-08 | 681275 | 0.004% | 30.54 |
| BMI | rs10867256 | 9 | 81367391 | T | C | 5.53E-01 | -1.18E-02 | 1.70E-03 | 8.70E-12 | 681275 | 0.007% | 48.18 |
| BMI | rs4671328 | 2 | 58935282 | G | T | 5.53E-01 | -2.19E-02 | 1.70E-03 | 2.20E-36 | 681275 | 0.024% | 165.95 |
| BMI | rs10953740 | 7 | 113460282 | G | A | 5.53E-01 | -1.53E-02 | 1.70E-03 | 1.00E-18 | 681275 | 0.012% | 81.00 |
| BMI | rs6673081 | 1 | 154989595 | C | T | 5.53E-01 | -1.00E-02 | 1.80E-03 | 1.80E-08 | 681275 | 0.005% | 30.86 |
| BMI | rs189843 | 5 | 164600151 | C | G | 5.56E-01 | -9.80E-03 | 1.70E-03 | 1.70E-08 | 681275 | 0.005% | 33.23 |
| BMI | rs3731695 | 2 | 203820275 | C | T | 5.58E-01 | 1.16E-02 | 1.60E-03 | 7.90E-13 | 681275 | 0.008% | 52.56 |
| BMI | rs2317299 | 2 | 236903093 | C | T | 5.60E-01 | -1.06E-02 | 1.70E-03 | 1.30E-09 | 681275 | 0.006% | 38.88 |
| BMI | rs4880341 | 10 | 133992689 | T | C | 5.61E-01 | -1.18E-02 | 1.70E-03 | 1.10E-11 | 681275 | 0.007% | 48.18 |
| BMI | rs339991 | 15 | 60913637 | G | A | 5.63E-01 | 1.24E-02 | 1.80E-03 | 1.20E-12 | 681275 | 0.007% | 47.46 |
| BMI | rs7551507 | 1 | 74995225 | T | C | 5.63E-01 | -1.84E-02 | 1.60E-03 | 9.30E-30 | 681275 | 0.019% | 132.25 |
| BMI | rs1075901 | 17 | 15943910 | C | T | 5.64E-01 | 1.21E-02 | 1.60E-03 | 1.20E-13 | 681275 | 0.008% | 57.19 |
| BMI | rs1296328 | 4 | 137083193 | C | A | 5.66E-01 | -1.79E-02 | 1.80E-03 | 4.90E-24 | 681275 | 0.015% | 98.89 |
| BMI | rs427943 | 21 | 46570896 | C | A | 5.67E-01 | 1.70E-02 | 1.70E-03 | 7.30E-23 | 681275 | 0.015% | 100.00 |
| BMI | rs577525 | 10 | 99769388 | C | T | 5.68E-01 | 1.66E-02 | 1.70E-03 | 9.70E-22 | 681275 | 0.014% | 95.35 |
| BMI | rs11030618 | 11 | 29243293 | T | C | 5.68E-01 | 1.10E-02 | 1.70E-03 | 2.40E-10 | 681275 | 0.006% | 41.87 |
| BMI | rs1476322 | 3 | 161446055 | A | G | 5.69E-01 | 1.01E-02 | 1.70E-03 | 5.00E-09 | 681275 | 0.005% | 35.30 |
| BMI | rs10920678 | 1 | 190239907 | G | A | 5.71E-01 | -1.55E-02 | 1.60E-03 | 1.50E-21 | 681275 | 0.014% | 93.85 |
| BMI | rs1144387 | 13 | 78365190 | C | G | 5.71E-01 | 9.80E-03 | 1.70E-03 | 1.60E-08 | 681275 | 0.005% | 33.23 |
| BMI | rs592483 | 11 | 69445173 | T | C | 5.72E-01 | -1.47E-02 | 1.70E-03 | 2.00E-18 | 681275 | 0.011% | 74.77 |
| BMI | rs6804842 | 3 | 25106437 | G | A | 5.72E-01 | 1.56E-02 | 1.70E-03 | 3.60E-21 | 681275 | 0.012% | 84.21 |
| BMI | rs7715256 | 5 | 153537893 | T | G | 5.78E-01 | -1.66E-02 | 1.60E-03 | 2.20E-24 | 681275 | 0.016% | 107.64 |
| BMI | rs429343 | 2 | 147903382 | G | A | 5.81E-01 | -1.50E-02 | 1.70E-03 | 6.80E-18 | 681275 | 0.011% | 77.85 |
| BMI | rs11165643 | 1 | 96924097 | T | C | 5.83E-01 | 2.06E-02 | 1.70E-03 | 1.40E-35 | 681275 | 0.022% | 146.84 |
| BMI | rs11738695 | 5 | 108699161 | A | C | 5.86E-01 | 9.70E-03 | 1.70E-03 | 2.00E-08 | 681275 | 0.005% | 32.56 |
| BMI | rs4757144 | 11 | 13331226 | A | G | 5.88E-01 | 1.69E-02 | 1.80E-03 | 5.60E-22 | 681275 | 0.013% | 88.15 |
| BMI | rs7826312 | 8 | 32400115 | C | T | 5.88E-01 | 1.04E-02 | 1.70E-03 | 4.90E-10 | 681275 | 0.005% | 37.43 |
| BMI | rs1656377 | 3 | 158285280 | C | T | 5.89E-01 | 9.90E-03 | 1.70E-03 | 1.60E-08 | 681275 | 0.005% | 33.91 |
| BMI | rs4820408 | 22 | 40604945 | G | T | 5.92E-01 | -1.51E-02 | 1.70E-03 | 2.10E-19 | 681275 | 0.012% | 78.90 |
| BMI | rs1436344 | 3 | 104606144 | C | G | 5.92E-01 | 1.41E-02 | 1.70E-03 | 4.10E-16 | 681275 | 0.010% | 68.79 |
| BMI | rs4012234 | 20 | 32553047 | G | T | 5.92E-01 | 1.41E-02 | 1.80E-03 | 9.90E-16 | 681275 | 0.009% | 61.36 |
| BMI | rs977747 | 1 | 47684677 | G | T | 5.95E-01 | -1.69E-02 | 1.70E-03 | 1.30E-24 | 681275 | 0.015% | 98.83 |
| BMI | rs1260326 | 2 | 27730940 | C | T | 5.97E-01 | 1.05E-02 | 1.70E-03 | 3.90E-10 | 681275 | 0.006% | 38.15 |
| BMI | rs11251352 | 10 | 2585792 | G | A | 5.99E-01 | 1.09E-02 | 1.80E-03 | 7.00E-10 | 681275 | 0.005% | 36.67 |
| BMI | rs7318817 | 13 | 28617708 | T | C | 6.07E-01 | -1.55E-02 | 1.80E-03 | 2.70E-18 | 681275 | 0.011% | 74.15 |
| BMI | rs11739877 | 5 | 105876806 | T | C | 6.12E-01 | 1.17E-02 | 1.80E-03 | 6.60E-11 | 681275 | 0.006% | 42.25 |
| BMI | rs3732084 | 2 | 207174316 | C | T | 6.14E-01 | 1.07E-02 | 1.80E-03 | 1.10E-09 | 681275 | 0.005% | 35.34 |
| BMI | rs6545714 | 2 | 59307725 | A | G | 6.14E-01 | -1.91E-02 | 1.70E-03 | 9.10E-31 | 681275 | 0.019% | 126.23 |
| BMI | rs10742752 | 11 | 45438374 | C | T | 6.16E-01 | 1.24E-02 | 1.70E-03 | 1.10E-13 | 681275 | 0.008% | 53.20 |
| BMI | rs879620 | 16 | 4015729 | T | C | 6.18E-01 | 2.31E-02 | 1.80E-03 | 5.30E-38 | 681275 | 0.024% | 164.69 |
| BMI | rs215634 | 7 | 32369148 | G | A | 6.21E-01 | -1.52E-02 | 1.80E-03 | 2.60E-17 | 681275 | 0.010% | 71.31 |
| BMI | rs657452 | 1 | 49589847 | G | A | 6.22E-01 | -1.88E-02 | 1.70E-03 | 7.20E-29 | 681275 | 0.018% | 122.30 |
| BMI | rs10832778 | 11 | 17394073 | G | C | 6.22E-01 | 1.25E-02 | 1.70E-03 | 1.30E-13 | 681275 | 0.008% | 54.07 |
| BMI | rs4414033 | 1 | 156406853 | A | G | 6.27E-01 | 1.29E-02 | 1.80E-03 | 1.40E-12 | 681275 | 0.008% | 51.36 |
| BMI | rs391300 | 17 | 2216258 | C | T | 6.28E-01 | -1.19E-02 | 1.70E-03 | 3.10E-12 | 681275 | 0.007% | 49.00 |
| BMI | rs7181498 | 15 | 95271404 | C | T | 6.31E-01 | -1.63E-02 | 1.80E-03 | 1.00E-19 | 681275 | 0.012% | 82.00 |
| BMI | rs1321432 | 20 | 6614691 | C | A | 6.32E-01 | 2.01E-02 | 1.80E-03 | 3.50E-29 | 681275 | 0.018% | 124.69 |
| BMI | rs1528435 | 2 | 181550962 | T | C | 6.33E-01 | 1.64E-02 | 1.70E-03 | 9.10E-23 | 681275 | 0.014% | 93.07 |
| BMI | rs4800191 | 18 | 22461398 | C | G | 6.37E-01 | 1.03E-02 | 1.70E-03 | 2.50E-09 | 681275 | 0.005% | 36.71 |
| BMI | rs4929923 | 11 | 8639200 | C | T | 6.38E-01 | 1.81E-02 | 1.70E-03 | 7.20E-27 | 681275 | 0.017% | 113.36 |
| BMI | rs2007231 | 1 | 115266306 | T | C | 6.39E-01 | -1.04E-02 | 1.80E-03 | 5.20E-09 | 681275 | 0.005% | 33.38 |
| BMI | rs9989141 | 14 | 94006257 | T | C | 6.39E-01 | 1.62E-02 | 1.70E-03 | 3.60E-21 | 681275 | 0.013% | 90.81 |
| BMI | rs4864201 | 4 | 130731284 | C | T | 6.47E-01 | -1.41E-02 | 1.70E-03 | 1.50E-16 | 681275 | 0.010% | 68.79 |
| BMI | rs12888955 | 14 | 103256877 | A | G | 6.51E-01 | -1.78E-02 | 1.80E-03 | 1.40E-22 | 681275 | 0.014% | 97.79 |
| BMI | rs1187352 | 9 | 87293457 | C | T | 6.52E-01 | 1.19E-02 | 1.80E-03 | 6.00E-11 | 681275 | 0.006% | 43.71 |
| BMI | rs7557796 | 2 | 86766153 | C | T | 6.52E-01 | -1.60E-02 | 1.80E-03 | 2.30E-19 | 681275 | 0.012% | 79.01 |
| BMI | rs287104 | 19 | 34290995 | A | G | 6.60E-01 | 1.15E-02 | 1.70E-03 | 4.40E-11 | 681275 | 0.007% | 45.76 |
| BMI | rs4818225 | 21 | 42629895 | G | A | 6.61E-01 | 1.17E-02 | 1.80E-03 | 2.30E-10 | 681275 | 0.006% | 42.25 |
| BMI | rs1218822 | 13 | 28011963 | A | G | 6.66E-01 | 1.68E-02 | 1.70E-03 | 1.90E-22 | 681275 | 0.014% | 97.66 |
| BMI | rs7730004 | 5 | 43191033 | T | C | 6.69E-01 | 1.48E-02 | 1.80E-03 | 9.10E-16 | 681275 | 0.010% | 67.60 |
| BMI | rs2600226 | 3 | 12928762 | T | C | 6.70E-01 | -1.16E-02 | 1.90E-03 | 3.70E-10 | 681275 | 0.005% | 37.27 |
| BMI | rs1937683 | 10 | 53679060 | T | C | 6.70E-01 | 1.09E-02 | 1.80E-03 | 3.20E-09 | 681275 | 0.005% | 36.67 |
| BMI | rs2357760 | 6 | 120213880 | A | G | 6.75E-01 | 1.45E-02 | 1.70E-03 | 6.80E-17 | 681275 | 0.011% | 72.75 |
| BMI | rs13250058 | 8 | 112270826 | T | G | 6.77E-01 | 1.12E-02 | 1.80E-03 | 2.90E-10 | 681275 | 0.006% | 38.72 |
| BMI | rs3806114 | 6 | 20482335 | A | G | 6.77E-01 | -1.13E-02 | 1.80E-03 | 3.40E-10 | 681275 | 0.006% | 39.41 |
| BMI | rs7358465 | 11 | 89990280 | T | C | 6.78E-01 | 1.03E-02 | 1.90E-03 | 3.00E-08 | 681275 | 0.004% | 29.39 |
| BMI | rs1885728 | 6 | 5977833 | A | G | 6.79E-01 | 1.08E-02 | 1.90E-03 | 1.00E-08 | 681275 | 0.005% | 32.31 |
| BMI | rs4653017 | 1 | 33776728 | T | C | 6.82E-01 | 1.22E-02 | 1.80E-03 | 4.50E-11 | 681275 | 0.007% | 45.94 |
| BMI | rs9300422 | 13 | 98223320 | G | A | 6.90E-01 | -1.03E-02 | 1.80E-03 | 4.00E-09 | 681275 | 0.005% | 32.74 |
| BMI | rs10795422 | 10 | 16759312 | G | A | 6.91E-01 | 1.39E-02 | 1.90E-03 | 9.30E-14 | 681275 | 0.008% | 53.52 |
| BMI | rs4237643 | 11 | 43648368 | G | T | 6.94E-01 | -2.23E-02 | 1.90E-03 | 4.30E-33 | 681275 | 0.020% | 137.75 |
| BMI | rs818524 | 1 | 85201228 | C | T | 6.94E-01 | 1.06E-02 | 1.90E-03 | 3.40E-08 | 681275 | 0.005% | 31.12 |
| BMI | rs2411182 | 17 | 35059718 | A | G | 6.94E-01 | 1.23E-02 | 1.90E-03 | 7.70E-11 | 681275 | 0.006% | 41.91 |
| BMI | rs7637852 | 3 | 44041777 | G | A | 6.95E-01 | -1.39E-02 | 1.90E-03 | 1.70E-13 | 681275 | 0.008% | 53.52 |
| BMI | rs685870 | 11 | 64111928 | C | T | 7.04E-01 | 1.20E-02 | 1.90E-03 | 2.40E-10 | 681275 | 0.006% | 39.89 |
| BMI | rs9375702 | 6 | 130384187 | T | C | 7.05E-01 | -1.15E-02 | 1.90E-03 | 7.90E-10 | 681275 | 0.005% | 36.63 |
| BMI | rs962273 | 17 | 46978353 | C | T | 7.06E-01 | 1.37E-02 | 1.90E-03 | 2.60E-13 | 681275 | 0.008% | 51.99 |
| BMI | rs3800229 | 6 | 108996963 | T | G | 7.12E-01 | 1.75E-02 | 1.80E-03 | 1.40E-22 | 681275 | 0.014% | 94.52 |
| BMI | rs4842491 | 12 | 89905537 | T | C | 7.14E-01 | 9.80E-03 | 1.80E-03 | 4.00E-08 | 681275 | 0.004% | 29.64 |
| BMI | rs10878946 | 12 | 69642315 | T | C | 7.14E-01 | -1.41E-02 | 1.90E-03 | 3.60E-13 | 681275 | 0.008% | 55.07 |
| BMI | rs1285997 | 14 | 91513029 | G | C | 7.15E-01 | 1.42E-02 | 1.90E-03 | 1.20E-13 | 681275 | 0.008% | 55.86 |
| BMI | rs11150911 | 18 | 73498528 | C | A | 7.19E-01 | -1.33E-02 | 1.80E-03 | 4.70E-13 | 681275 | 0.008% | 54.60 |
| BMI | rs294704 | 5 | 152519088 | T | G | 7.24E-01 | -1.13E-02 | 1.90E-03 | 4.00E-09 | 681275 | 0.005% | 35.37 |
| BMI | rs12546578 | 8 | 85085268 | A | T | 7.25E-01 | 1.46E-02 | 2.00E-03 | 1.00E-13 | 681275 | 0.008% | 53.29 |
| BMI | rs1452075 | 3 | 62481063 | T | C | 7.28E-01 | 1.41E-02 | 1.80E-03 | 1.30E-14 | 681275 | 0.009% | 61.36 |
| BMI | rs7730898 | 5 | 170459675 | A | G | 7.29E-01 | 1.68E-02 | 1.80E-03 | 4.50E-20 | 681275 | 0.013% | 87.11 |
| BMI | rs9927848 | 16 | 23833071 | A | C | 7.33E-01 | -1.22E-02 | 2.00E-03 | 6.40E-10 | 681275 | 0.005% | 37.21 |
| BMI | rs1431659 | 8 | 73439070 | G | A | 7.34E-01 | -1.96E-02 | 1.90E-03 | 6.00E-24 | 681275 | 0.016% | 106.42 |
| BMI | rs876605 | 5 | 77801359 | G | A | 7.35E-01 | -1.08E-02 | 2.00E-03 | 3.40E-08 | 681275 | 0.004% | 29.16 |
| BMI | rs4786903 | 16 | 6697104 | G | A | 7.37E-01 | 1.25E-02 | 2.00E-03 | 3.50E-10 | 681275 | 0.006% | 39.06 |
| BMI | rs934224 | 2 | 16613889 | T | C | 7.40E-01 | 1.07E-02 | 2.00E-03 | 4.70E-08 | 681275 | 0.004% | 28.62 |
| BMI | rs1365466 | 18 | 36182440 | T | C | 7.41E-01 | -1.37E-02 | 1.90E-03 | 3.30E-13 | 681275 | 0.008% | 51.99 |
| BMI | rs2694047 | 8 | 116750548 | G | A | 7.47E-01 | 1.88E-02 | 2.00E-03 | 3.90E-21 | 681275 | 0.013% | 88.36 |
| BMI | rs6512302 | 20 | 62691550 | C | G | 7.51E-01 | 1.42E-02 | 2.00E-03 | 2.10E-12 | 681275 | 0.007% | 50.41 |
| BMI | rs947612 | 6 | 73738661 | A | G | 7.52E-01 | -1.16E-02 | 2.00E-03 | 5.60E-09 | 681275 | 0.005% | 33.64 |
| BMI | rs2791653 | 1 | 11129848 | G | A | 7.58E-01 | -1.41E-02 | 1.90E-03 | 1.30E-13 | 681275 | 0.008% | 55.07 |
| BMI | rs852056 | 20 | 17102860 | C | T | 7.58E-01 | -1.28E-02 | 2.00E-03 | 1.80E-10 | 681275 | 0.006% | 40.96 |
| BMI | rs6587552 | 1 | 151018861 | G | A | 7.59E-01 | -1.73E-02 | 2.00E-03 | 1.60E-17 | 681275 | 0.011% | 74.82 |
| BMI | rs10747488 | 1 | 98299475 | A | C | 7.60E-01 | -1.23E-02 | 2.00E-03 | 1.20E-09 | 681275 | 0.006% | 37.82 |
| BMI | rs156201 | 6 | 104847441 | C | G | 7.61E-01 | 1.23E-02 | 2.00E-03 | 5.80E-10 | 681275 | 0.006% | 37.82 |
| BMI | rs8181823 | 13 | 65477940 | C | A | 7.61E-01 | 1.27E-02 | 2.00E-03 | 4.10E-10 | 681275 | 0.006% | 40.32 |
| BMI | rs9304665 | 19 | 47602577 | A | T | 7.63E-01 | 2.29E-02 | 2.00E-03 | 2.90E-29 | 681275 | 0.019% | 131.10 |
| BMI | rs9951619 | 18 | 56882326 | G | T | 7.64E-01 | 1.56E-02 | 2.00E-03 | 1.40E-15 | 681275 | 0.009% | 60.84 |
| BMI | rs865809 | 3 | 183997735 | G | A | 7.68E-01 | -1.27E-02 | 2.00E-03 | 5.40E-10 | 681275 | 0.006% | 40.32 |
| BMI | rs4981693 | 14 | 29680331 | A | G | 7.71E-01 | 2.06E-02 | 2.00E-03 | 6.90E-24 | 681275 | 0.016% | 106.09 |
| BMI | rs7024334 | 9 | 109072075 | G | T | 7.74E-01 | -1.38E-02 | 2.00E-03 | 3.10E-12 | 681275 | 0.007% | 47.61 |
| BMI | rs645040 | 3 | 135926622 | T | G | 7.76E-01 | 1.71E-02 | 2.00E-03 | 2.50E-18 | 681275 | 0.011% | 73.10 |
| BMI | rs4310573 | 11 | 97855562 | T | C | 7.81E-01 | 1.16E-02 | 2.10E-03 | 3.50E-08 | 681275 | 0.004% | 30.51 |
| BMI | rs4430672 | 14 | 63094407 | C | T | 8.00E-01 | -1.27E-02 | 2.20E-03 | 3.90E-09 | 681275 | 0.005% | 33.32 |
| BMI | rs6443750 | 3 | 181329682 | C | T | 8.07E-01 | 1.48E-02 | 2.10E-03 | 3.20E-12 | 681275 | 0.007% | 49.67 |
| BMI | rs33500 | 3 | 42427191 | T | C | 8.08E-01 | -1.67E-02 | 2.20E-03 | 4.30E-14 | 681275 | 0.008% | 57.62 |
| BMI | rs2543132 | 8 | 15536311 | C | G | 8.13E-01 | 1.46E-02 | 2.20E-03 | 5.00E-11 | 681275 | 0.006% | 44.04 |
| BMI | rs1993709 | 1 | 72838529 | G | A | 8.18E-01 | 3.31E-02 | 2.10E-03 | 1.90E-57 | 681275 | 0.036% | 248.44 |
| BMI | rs9816226 | 3 | 185834499 | T | A | 8.20E-01 | 3.23E-02 | 2.10E-03 | 1.60E-52 | 681275 | 0.035% | 236.57 |
| BMI | rs226000 | 14 | 30488699 | T | C | 8.25E-01 | 1.19E-02 | 2.20E-03 | 3.60E-08 | 681275 | 0.004% | 29.26 |
| BMI | rs13021737 | 2 | 632348 | G | A | 8.32E-01 | 5.74E-02 | 2.10E-03 | 7.50E-157 | 681275 | 0.110% | 747.11 |
| BMI | rs6692586 | 1 | 23299906 | G | A | 8.32E-01 | -1.92E-02 | 2.30E-03 | 1.10E-16 | 681275 | 0.010% | 69.69 |
| BMI | rs930295 | 2 | 50233352 | C | A | 8.42E-01 | -2.11E-02 | 2.30E-03 | 1.00E-19 | 681275 | 0.012% | 84.16 |
| BMI | rs1241986 | 18 | 6873954 | A | G | 8.48E-01 | -1.39E-02 | 2.40E-03 | 1.10E-08 | 681275 | 0.005% | 33.54 |
| BMI | rs1535660 | 9 | 10371073 | C | T | 8.55E-01 | -1.47E-02 | 2.50E-03 | 5.20E-09 | 681275 | 0.005% | 34.57 |
| BMI | rs946824 | 1 | 243684019 | C | T | 8.59E-01 | -2.06E-02 | 2.60E-03 | 1.10E-15 | 681275 | 0.009% | 62.77 |
| BMI | rs9806742 | 15 | 73051219 | A | G | 8.83E-01 | 2.08E-02 | 2.60E-03 | 1.40E-15 | 681275 | 0.009% | 64.00 |
| BMI | rs754635 | 3 | 42305131 | G | C | 8.87E-01 | 1.98E-02 | 2.70E-03 | 2.20E-13 | 681275 | 0.008% | 53.78 |
| BMI | rs380857 | 9 | 101491066 | A | C | 8.88E-01 | -1.51E-02 | 2.70E-03 | 3.60E-08 | 681275 | 0.005% | 31.28 |
| BMI | rs10247983 | 7 | 114590228 | A | G | 9.21E-01 | 2.01E-02 | 3.30E-03 | 1.70E-09 | 681275 | 0.005% | 37.10 |
| BMI | rs4482463 | 2 | 205375909 | A | C | 9.21E-01 | -3.31E-02 | 3.30E-03 | 2.80E-23 | 681275 | 0.015% | 100.61 |
| BMI | rs208015 | 17 | 46252346 | C | T | 9.22E-01 | -3.56E-02 | 3.40E-03 | 1.40E-25 | 681275 | 0.016% | 109.63 |
| BMI | rs2943465 | 12 | 19265921 | C | T | 9.44E-01 | 2.48E-02 | 3.90E-03 | 2.00E-10 | 681275 | 0.006% | 40.44 |
| BMI | rs6050446 | 20 | 25195509 | G | A | 9.70E-01 | 3.43E-02 | 4.70E-03 | 4.40E-13 | 681275 | 0.008% | 53.26 |
| Hand grip strength (left) | rs6680160 | 1 | 32072737 | G | A | 6.28E-01 | 1.01E-02 | 1.54E-03 | 6.00E-11 | 461026 | 0.009% | 42.81 |
| Hand grip strength (left) | rs7516571 | 1 | 40733658 | G | A | 2.59E-01 | 9.38E-03 | 1.69E-03 | 3.10E-08 | 461026 | 0.007% | 30.66 |
| Hand grip strength (left) | rs150330307 | 1 | 160160801 | C | T | 3.19E-02 | -3.08E-02 | 4.22E-03 | 2.90E-13 | 461026 | 0.012% | 53.27 |
| Hand grip strength (left) | rs2800789 | 1 | 164578242 | C | A | 4.80E-01 | 8.26E-03 | 1.49E-03 | 3.10E-08 | 461026 | 0.007% | 30.63 |
| Hand grip strength (left) | rs1044299 | 1 | 176811873 | T | C | 5.46E-01 | 1.40E-02 | 1.49E-03 | 6.60E-21 | 461026 | 0.019% | 87.98 |
| Hand grip strength (left) | rs11121542 | 1 | 10393920 | A | G | 1.23E-01 | -1.58E-02 | 2.26E-03 | 3.00E-12 | 461026 | 0.011% | 48.67 |
| Hand grip strength (left) | rs4121165 | 1 | 78276977 | A | G | 2.11E-01 | -1.14E-02 | 1.82E-03 | 3.40E-10 | 461026 | 0.009% | 39.41 |
| Hand grip strength (left) | rs58670122 | 1 | 22492613 | G | A | 1.43E-01 | -1.18E-02 | 2.14E-03 | 3.60E-08 | 461026 | 0.007% | 30.35 |
| Hand grip strength (left) | rs10788958 | 1 | 54040670 | G | C | 6.45E-01 | 1.42E-02 | 1.56E-03 | 1.00E-19 | 461026 | 0.018% | 82.54 |
| Hand grip strength (left) | rs4335354 | 1 | 88899964 | A | C | 3.16E-01 | -9.39E-03 | 1.60E-03 | 4.60E-09 | 461026 | 0.007% | 34.36 |
| Hand grip strength (left) | rs1884447 | 1 | 185021410 | A | G | 4.01E-01 | 8.46E-03 | 1.51E-03 | 2.30E-08 | 461026 | 0.007% | 31.23 |
| Hand grip strength (left) | rs61818100 | 1 | 190962663 | C | T | 1.17E-01 | 1.34E-02 | 2.31E-03 | 6.20E-09 | 461026 | 0.007% | 33.76 |
| Hand grip strength (left) | rs823130 | 1 | 205714372 | T | C | 4.33E-01 | -1.13E-02 | 1.50E-03 | 4.40E-14 | 461026 | 0.012% | 57.00 |
| Hand grip strength (left) | rs11204664 | 1 | 150531380 | C | T | 5.79E-01 | -8.65E-03 | 1.50E-03 | 8.10E-09 | 461026 | 0.007% | 33.25 |
| Hand grip strength (left) | rs6689375 | 1 | 227721627 | T | A | 1.85E-01 | -1.60E-02 | 1.91E-03 | 5.30E-17 | 461026 | 0.015% | 70.21 |
| Hand grip strength (left) | rs6433478 | 2 | 175241482 | C | T | 5.44E-01 | 9.06E-03 | 1.50E-03 | 1.50E-09 | 461026 | 0.008% | 36.48 |
| Hand grip strength (left) | rs12473732 | 2 | 44118428 | T | C | 4.87E-01 | 1.10E-02 | 1.49E-03 | 1.40E-13 | 461026 | 0.012% | 54.66 |
| Hand grip strength (left) | rs7571789 | 2 | 70714793 | C | T | 5.23E-01 | 1.30E-02 | 1.49E-03 | 3.00E-18 | 461026 | 0.016% | 75.89 |
| Hand grip strength (left) | rs7575451 | 2 | 152352843 | G | C | 6.50E-01 | -9.73E-03 | 1.55E-03 | 3.80E-10 | 461026 | 0.009% | 39.24 |
| Hand grip strength (left) | rs1434095 | 2 | 179254330 | C | T | 8.75E-01 | 1.40E-02 | 2.26E-03 | 5.30E-10 | 461026 | 0.008% | 38.57 |
| Hand grip strength (left) | rs17630248 | 2 | 201137782 | C | T | 3.48E-01 | -9.18E-03 | 1.56E-03 | 4.10E-09 | 461026 | 0.008% | 34.59 |
| Hand grip strength (left) | rs1981612 | 2 | 199235664 | A | C | 4.56E-01 | 9.23E-03 | 1.51E-03 | 1.00E-09 | 461026 | 0.008% | 37.32 |
| Hand grip strength (left) | rs11125803 | 2 | 25052177 | T | C | 7.41E-01 | 1.43E-02 | 1.70E-03 | 3.80E-17 | 461026 | 0.015% | 70.88 |
| Hand grip strength (left) | rs1641457 | 2 | 40421990 | G | T | 2.23E-01 | 1.20E-02 | 1.78E-03 | 1.40E-11 | 461026 | 0.010% | 45.65 |
| Hand grip strength (left) | rs3819121 | 2 | 135622860 | C | T | 3.69E-01 | 1.41E-02 | 1.53E-03 | 2.90E-20 | 461026 | 0.018% | 85.03 |
| Hand grip strength (left) | rs10176878 | 2 | 59952274 | C | T | 1.91E-01 | -1.29E-02 | 1.90E-03 | 8.60E-12 | 461026 | 0.010% | 46.63 |
| Hand grip strength (left) | rs61286123 | 2 | 60205600 | C | T | 2.28E-01 | -1.01E-02 | 1.77E-03 | 1.20E-08 | 461026 | 0.007% | 32.55 |
| Hand grip strength (left) | rs34030812 | 2 | 144248905 | C | T | 3.67E-01 | -1.02E-02 | 1.54E-03 | 4.10E-11 | 461026 | 0.009% | 43.57 |
| Hand grip strength (left) | rs10205394 | 2 | 218150948 | C | G | 2.01E-01 | -1.13E-02 | 1.86E-03 | 1.10E-09 | 461026 | 0.008% | 37.18 |
| Hand grip strength (left) | rs1551042 | 3 | 85630551 | C | A | 6.47E-01 | -1.11E-02 | 1.55E-03 | 7.40E-13 | 461026 | 0.011% | 51.43 |
| Hand grip strength (left) | rs9866627 | 3 | 135522715 | A | C | 8.44E-02 | -1.56E-02 | 2.68E-03 | 5.70E-09 | 461026 | 0.007% | 33.94 |
| Hand grip strength (left) | rs112485536 | 3 | 195971019 | T | C | 7.55E-02 | 1.62E-02 | 2.82E-03 | 9.40E-09 | 461026 | 0.007% | 32.96 |
| Hand grip strength (left) | rs62253653 | 3 | 53013267 | G | A | 2.95E-01 | 1.07E-02 | 1.63E-03 | 5.50E-11 | 461026 | 0.009% | 43.00 |
| Hand grip strength (left) | rs6802071 | 3 | 38574237 | T | C | 4.35E-01 | -9.40E-03 | 1.50E-03 | 3.80E-10 | 461026 | 0.009% | 39.21 |
| Hand grip strength (left) | rs71298370 | 3 | 71164965 | A | G | 8.62E-02 | 1.49E-02 | 2.70E-03 | 3.80E-08 | 461026 | 0.007% | 30.26 |
| Hand grip strength (left) | rs13091492 | 3 | 81891476 | G | A | 3.73E-01 | -8.48E-03 | 1.53E-03 | 3.30E-08 | 461026 | 0.007% | 30.55 |
| Hand grip strength (left) | rs10934857 | 3 | 128199662 | A | G | 2.59E-01 | 9.29E-03 | 1.70E-03 | 4.80E-08 | 461026 | 0.006% | 29.78 |
| Hand grip strength (left) | rs4498020 | 3 | 13810820 | A | C | 7.24E-01 | -1.04E-02 | 1.67E-03 | 4.30E-10 | 461026 | 0.008% | 38.96 |
| Hand grip strength (left) | rs4677601 | 3 | 71368790 | G | A | 5.10E-01 | 9.06E-03 | 1.49E-03 | 1.10E-09 | 461026 | 0.008% | 37.21 |
| Hand grip strength (left) | rs2871960 | 3 | 141121814 | C | A | 4.45E-01 | 1.21E-02 | 1.49E-03 | 5.50E-16 | 461026 | 0.014% | 65.62 |
| Hand grip strength (left) | rs35609019 | 4 | 7847892 | C | G | 3.98E-01 | 9.48E-03 | 1.54E-03 | 7.70E-10 | 461026 | 0.008% | 37.84 |
| Hand grip strength (left) | rs13107325 | 4 | 103188709 | T | C | 7.49E-02 | -2.62E-02 | 2.82E-03 | 1.80E-20 | 461026 | 0.019% | 85.98 |
| Hand grip strength (left) | rs56338231 | 4 | 30867393 | G | A | 2.58E-01 | -1.08E-02 | 1.70E-03 | 1.70E-10 | 461026 | 0.009% | 40.84 |
| Hand grip strength (left) | rs13146142 | 4 | 17931318 | C | T | 1.59E-01 | -2.02E-02 | 2.03E-03 | 2.30E-23 | 461026 | 0.022% | 99.16 |
| Hand grip strength (left) | rs34587452 | 4 | 1009900 | C | G | 2.15E-01 | -1.14E-02 | 1.81E-03 | 3.20E-10 | 461026 | 0.009% | 39.53 |
| Hand grip strength (left) | rs13106087 | 4 | 145566864 | C | T | 8.30E-01 | 1.16E-02 | 1.97E-03 | 3.90E-09 | 461026 | 0.008% | 34.65 |
| Hand grip strength (left) | rs997850 | 4 | 154838434 | C | G | 6.05E-01 | -8.87E-03 | 1.53E-03 | 6.30E-09 | 461026 | 0.007% | 33.73 |
| Hand grip strength (left) | rs34722008 | 4 | 38659594 | A | G | 3.53E-01 | 8.59E-03 | 1.55E-03 | 3.30E-08 | 461026 | 0.007% | 30.50 |
| Hand grip strength (left) | rs2850379 | 4 | 102917419 | A | C | 4.32E-01 | -8.29E-03 | 1.50E-03 | 3.30E-08 | 461026 | 0.007% | 30.54 |
| Hand grip strength (left) | rs75497896 | 4 | 119636703 | C | T | 5.10E-02 | -2.07E-02 | 3.38E-03 | 9.10E-10 | 461026 | 0.008% | 37.51 |
| Hand grip strength (left) | rs116409670 | 5 | 37327472 | T | C | 7.98E-02 | -1.52E-02 | 2.74E-03 | 2.60E-08 | 461026 | 0.007% | 30.97 |
| Hand grip strength (left) | rs55681913 | 5 | 42687629 | C | T | 1.06E-01 | 1.38E-02 | 2.44E-03 | 1.60E-08 | 461026 | 0.007% | 31.96 |
| Hand grip strength (left) | rs13356200 | 5 | 67820946 | G | T | 3.94E-01 | -8.77E-03 | 1.53E-03 | 9.70E-09 | 461026 | 0.007% | 32.90 |
| Hand grip strength (left) | rs2431112 | 5 | 103931707 | A | G | 4.41E-01 | -9.58E-03 | 1.50E-03 | 1.40E-10 | 461026 | 0.009% | 41.10 |
| Hand grip strength (left) | rs2631360 | 5 | 131707429 | A | G | 5.19E-01 | -1.09E-02 | 1.48E-03 | 1.90E-13 | 461026 | 0.012% | 54.10 |
| Hand grip strength (left) | rs6882168 | 5 | 39402647 | T | C | 3.37E-01 | -9.35E-03 | 1.57E-03 | 2.80E-09 | 461026 | 0.008% | 35.33 |
| Hand grip strength (left) | rs113918482 | 5 | 161289270 | G | A | 2.23E-01 | -1.01E-02 | 1.79E-03 | 1.50E-08 | 461026 | 0.007% | 32.02 |
| Hand grip strength (left) | rs2974438 | 5 | 168250903 | A | G | 2.11E-01 | -1.01E-02 | 1.82E-03 | 3.30E-08 | 461026 | 0.007% | 30.51 |
| Hand grip strength (left) | rs185320691 | 6 | 32490292 | C | G | 1.04E-01 | -1.67E-02 | 2.69E-03 | 5.50E-10 | 461026 | 0.008% | 38.50 |
| Hand grip strength (left) | rs12528131 | 6 | 105389104 | G | A | 4.88E-01 | -8.83E-03 | 1.49E-03 | 2.80E-09 | 461026 | 0.008% | 35.31 |
| Hand grip strength (left) | rs9371201 | 6 | 150145001 | T | C | 3.35E-01 | -9.34E-03 | 1.57E-03 | 3.00E-09 | 461026 | 0.008% | 35.21 |
| Hand grip strength (left) | rs77485342 | 6 | 30842866 | T | C | 1.80E-02 | 3.30E-02 | 5.58E-03 | 3.50E-09 | 461026 | 0.008% | 34.88 |
| Hand grip strength (left) | rs9388769 | 6 | 130379954 | A | G | 6.73E-01 | -1.41E-02 | 1.58E-03 | 5.10E-19 | 461026 | 0.017% | 79.37 |
| Hand grip strength (left) | rs35175534 | 6 | 32530029 | C | A | 1.40E-01 | -1.64E-02 | 2.35E-03 | 3.60E-12 | 461026 | 0.010% | 48.35 |
| Hand grip strength (left) | rs113315602 | 6 | 32574575 | C | A | 9.58E-02 | -1.82E-02 | 2.66E-03 | 8.60E-12 | 461026 | 0.010% | 46.63 |
| Hand grip strength (left) | rs723588 | 6 | 81056634 | C | T | 1.43E-01 | 1.28E-02 | 2.12E-03 | 1.60E-09 | 461026 | 0.008% | 36.42 |
| Hand grip strength (left) | rs11243202 | 6 | 7719065 | C | T | 4.86E-01 | 9.72E-03 | 1.49E-03 | 7.00E-11 | 461026 | 0.009% | 42.53 |
| Hand grip strength (left) | rs4713506 | 6 | 32113980 | A | G | 2.56E-01 | -1.57E-02 | 1.70E-03 | 2.00E-20 | 461026 | 0.019% | 85.77 |
| Hand grip strength (left) | rs2038760 | 6 | 2680732 | T | C | 1.71E-01 | -1.15E-02 | 1.99E-03 | 6.60E-09 | 461026 | 0.007% | 33.66 |
| Hand grip strength (left) | rs41271299 | 6 | 19839415 | T | C | 5.12E-02 | 2.12E-02 | 3.36E-03 | 3.00E-10 | 461026 | 0.009% | 39.67 |
| Hand grip strength (left) | rs9371881 | 6 | 155638213 | A | G | 3.59E-01 | 9.48E-03 | 1.55E-03 | 9.60E-10 | 461026 | 0.008% | 37.40 |
| Hand grip strength (left) | rs4621706 | 7 | 39303296 | T | C | 5.44E-01 | -1.17E-02 | 1.50E-03 | 6.00E-15 | 461026 | 0.013% | 60.91 |
| Hand grip strength (left) | rs11769549 | 7 | 23122239 | A | T | 6.24E-02 | 2.05E-02 | 3.11E-03 | 4.50E-11 | 461026 | 0.009% | 43.37 |
| Hand grip strength (left) | rs12533765 | 7 | 127699186 | G | A | 2.80E-01 | -9.20E-03 | 1.65E-03 | 2.60E-08 | 461026 | 0.007% | 31.01 |
| Hand grip strength (left) | rs16870531 | 7 | 120660682 | T | C | 2.38E-01 | 1.12E-02 | 1.74E-03 | 1.30E-10 | 461026 | 0.009% | 41.30 |
| Hand grip strength (left) | rs13227429 | 7 | 140560023 | C | T | 5.61E-01 | -8.59E-03 | 1.50E-03 | 9.80E-09 | 461026 | 0.007% | 32.88 |
| Hand grip strength (left) | rs6977081 | 7 | 150542515 | T | G | 3.34E-01 | 1.48E-02 | 1.59E-03 | 1.40E-20 | 461026 | 0.019% | 86.48 |
| Hand grip strength (left) | rs12673062 | 7 | 4710677 | A | G | 2.16E-01 | -1.08E-02 | 1.81E-03 | 2.60E-09 | 461026 | 0.008% | 35.47 |
| Hand grip strength (left) | rs73307079 | 7 | 19020024 | C | T | 2.11E-01 | 1.11E-02 | 1.83E-03 | 1.40E-09 | 461026 | 0.008% | 36.64 |
| Hand grip strength (left) | rs6962338 | 7 | 69160985 | G | A | 4.40E-02 | -2.14E-02 | 3.62E-03 | 3.20E-09 | 461026 | 0.008% | 35.03 |
| Hand grip strength (left) | rs17282763 | 7 | 82520166 | C | T | 2.96E-01 | 8.94E-03 | 1.63E-03 | 4.40E-08 | 461026 | 0.007% | 29.97 |
| Hand grip strength (left) | rs821100 | 8 | 89448877 | G | A | 2.66E-01 | -1.02E-02 | 1.69E-03 | 1.50E-09 | 461026 | 0.008% | 36.58 |
| Hand grip strength (left) | rs59116179 | 8 | 22603454 | T | C | 6.17E-01 | 8.57E-03 | 1.54E-03 | 2.40E-08 | 461026 | 0.007% | 31.14 |
| Hand grip strength (left) | rs4737446 | 8 | 57665019 | T | G | 6.95E-01 | 1.04E-02 | 1.62E-03 | 1.20E-10 | 461026 | 0.009% | 41.41 |
| Hand grip strength (left) | rs1486925 | 8 | 78827617 | C | T | 3.15E-01 | -1.05E-02 | 1.60E-03 | 6.30E-11 | 461026 | 0.009% | 42.74 |
| Hand grip strength (left) | rs4398863 | 8 | 135695110 | C | G | 7.37E-01 | -9.45E-03 | 1.68E-03 | 2.00E-08 | 461026 | 0.007% | 31.47 |
| Hand grip strength (left) | rs4739739 | 8 | 81304576 | G | A | 4.15E-01 | -8.53E-03 | 1.50E-03 | 1.40E-08 | 461026 | 0.007% | 32.17 |
| Hand grip strength (left) | rs10097417 | 8 | 110361477 | G | A | 1.71E-01 | -1.32E-02 | 1.97E-03 | 1.90E-11 | 461026 | 0.010% | 45.08 |
| Hand grip strength (left) | rs7026798 | 9 | 16427378 | C | T | 4.32E-01 | 8.24E-03 | 1.51E-03 | 4.60E-08 | 461026 | 0.006% | 29.89 |
| Hand grip strength (left) | rs7856625 | 9 | 119345083 | T | C | 6.10E-01 | -1.11E-02 | 1.52E-03 | 2.50E-13 | 461026 | 0.012% | 53.55 |
| Hand grip strength (left) | rs16910750 | 9 | 99084471 | C | G | 1.60E-01 | 1.12E-02 | 2.04E-03 | 3.60E-08 | 461026 | 0.007% | 30.37 |
| Hand grip strength (left) | rs116922558 | 9 | 118802375 | G | A | 3.99E-02 | -2.16E-02 | 3.85E-03 | 2.10E-08 | 461026 | 0.007% | 31.36 |
| Hand grip strength (left) | rs2789514 | 9 | 129833029 | A | G | 8.68E-01 | 1.21E-02 | 2.21E-03 | 4.90E-08 | 461026 | 0.006% | 29.77 |
| Hand grip strength (left) | rs10988217 | 9 | 131888116 | G | A | 6.04E-01 | -9.21E-03 | 1.53E-03 | 1.70E-09 | 461026 | 0.008% | 36.25 |
| Hand grip strength (left) | rs11002322 | 10 | 79649653 | T | G | 3.40E-01 | -9.99E-03 | 1.57E-03 | 2.10E-10 | 461026 | 0.009% | 40.35 |
| Hand grip strength (left) | rs10786706 | 10 | 104500659 | T | C | 4.66E-01 | 1.00E-02 | 1.49E-03 | 1.70E-11 | 461026 | 0.010% | 45.26 |
| Hand grip strength (left) | rs35236379 | 10 | 5727292 | T | G | 1.42E-01 | 1.24E-02 | 2.12E-03 | 5.80E-09 | 461026 | 0.007% | 33.91 |
| Hand grip strength (left) | rs11003014 | 10 | 81231387 | G | A | 1.61E-01 | 1.14E-02 | 2.02E-03 | 1.60E-08 | 461026 | 0.007% | 31.99 |
| Hand grip strength (left) | rs4962700 | 10 | 126479989 | G | C | 3.02E-01 | 9.04E-03 | 1.63E-03 | 3.20E-08 | 461026 | 0.007% | 30.60 |
| Hand grip strength (left) | rs772014 | 10 | 104062494 | G | A | 3.93E-01 | -1.06E-02 | 1.52E-03 | 2.70E-12 | 461026 | 0.011% | 48.87 |
| Hand grip strength (left) | rs10821939 | 10 | 63751748 | A | G | 5.73E-01 | -9.35E-03 | 1.50E-03 | 4.90E-10 | 461026 | 0.008% | 38.71 |
| Hand grip strength (left) | rs1556659 | 10 | 130834698 | T | C | 3.82E-01 | 1.63E-02 | 1.53E-03 | 2.50E-26 | 461026 | 0.024% | 112.69 |
| Hand grip strength (left) | rs72977282 | 11 | 74300441 | A | T | 4.14E-01 | -1.56E-02 | 1.51E-03 | 7.80E-25 | 461026 | 0.023% | 105.90 |
| Hand grip strength (left) | rs4930236 | 11 | 68414000 | A | C | 8.36E-01 | 1.19E-02 | 2.02E-03 | 3.80E-09 | 461026 | 0.008% | 34.70 |
| Hand grip strength (left) | rs10831903 | 11 | 12758660 | T | C | 4.23E-01 | 9.29E-03 | 1.51E-03 | 8.10E-10 | 461026 | 0.008% | 37.75 |
| Hand grip strength (left) | rs12790261 | 11 | 66988048 | A | C | 8.24E-02 | -2.52E-02 | 2.70E-03 | 1.20E-20 | 461026 | 0.019% | 86.83 |
| Hand grip strength (left) | rs7124681 | 11 | 47529947 | A | C | 4.08E-01 | -1.17E-02 | 1.51E-03 | 1.00E-14 | 461026 | 0.013% | 59.89 |
| Hand grip strength (left) | rs61389091 | 11 | 74427921 | T | C | 4.17E-02 | 2.62E-02 | 3.73E-03 | 2.40E-12 | 461026 | 0.011% | 49.12 |
| Hand grip strength (left) | rs34845616 | 11 | 133792644 | A | G | 2.46E-01 | 1.08E-02 | 1.73E-03 | 4.00E-10 | 461026 | 0.008% | 39.09 |
| Hand grip strength (left) | rs76895963 | 12 | 4384844 | G | T | 2.07E-02 | 3.60E-02 | 5.75E-03 | 3.90E-10 | 461026 | 0.008% | 39.14 |
| Hand grip strength (left) | rs10846071 | 12 | 15016236 | T | C | 3.94E-01 | -1.66E-02 | 1.52E-03 | 5.30E-28 | 461026 | 0.026% | 120.36 |
| Hand grip strength (left) | rs11168357 | 12 | 48412138 | A | G | 2.46E-01 | -9.63E-03 | 1.73E-03 | 2.50E-08 | 461026 | 0.007% | 31.10 |
| Hand grip strength (left) | rs4575361 | 12 | 124410529 | T | A | 3.12E-01 | -1.08E-02 | 1.60E-03 | 1.60E-11 | 461026 | 0.010% | 45.46 |
| Hand grip strength (left) | rs12316046 | 12 | 15054415 | G | A | 3.78E-01 | -1.74E-02 | 1.53E-03 | 5.00E-30 | 461026 | 0.028% | 129.59 |
| Hand grip strength (left) | rs7970350 | 12 | 66360164 | T | C | 4.94E-01 | -1.01E-02 | 1.48E-03 | 8.60E-12 | 461026 | 0.010% | 46.63 |
| Hand grip strength (left) | rs7963801 | 12 | 79685226 | C | T | 5.72E-01 | -1.04E-02 | 1.51E-03 | 4.60E-12 | 461026 | 0.010% | 47.87 |
| Hand grip strength (left) | rs11111267 | 12 | 102811239 | G | A | 1.81E-01 | 1.08E-02 | 1.93E-03 | 1.90E-08 | 461026 | 0.007% | 31.56 |
| Hand grip strength (left) | rs3118903 | 13 | 51099577 | A | G | 2.20E-01 | -1.74E-02 | 1.80E-03 | 2.70E-22 | 461026 | 0.020% | 94.28 |
| Hand grip strength (left) | rs56060323 | 13 | 60473485 | T | C | 3.15E-01 | 9.07E-03 | 1.60E-03 | 1.50E-08 | 461026 | 0.007% | 32.03 |
| Hand grip strength (left) | rs12889267 | 14 | 21542766 | G | A | 1.67E-01 | -1.37E-02 | 1.99E-03 | 4.70E-12 | 461026 | 0.010% | 47.80 |
| Hand grip strength (left) | rs7148603 | 14 | 36683779 | A | G | 3.59E-01 | 9.57E-03 | 1.59E-03 | 1.60E-09 | 461026 | 0.008% | 36.42 |
| Hand grip strength (left) | rs2359239 | 14 | 75326771 | T | C | 3.92E-01 | -8.81E-03 | 1.52E-03 | 6.90E-09 | 461026 | 0.007% | 33.56 |
| Hand grip strength (left) | rs10144445 | 14 | 39695362 | G | C | 3.50E-01 | -9.37E-03 | 1.56E-03 | 1.90E-09 | 461026 | 0.008% | 36.11 |
| Hand grip strength (left) | rs28542042 | 15 | 74213357 | T | C | 3.09E-01 | 1.10E-02 | 1.62E-03 | 1.20E-11 | 461026 | 0.010% | 46.03 |
| Hand grip strength (left) | rs12906830 | 15 | 56963503 | C | T | 6.01E-01 | 1.08E-02 | 1.52E-03 | 9.30E-13 | 461026 | 0.011% | 51.00 |
| Hand grip strength (left) | rs3959716 | 15 | 73106615 | G | C | 5.67E-01 | -8.33E-03 | 1.50E-03 | 3.10E-08 | 461026 | 0.007% | 30.63 |
| Hand grip strength (left) | rs17466480 | 15 | 77390870 | G | A | 3.87E-01 | -1.18E-02 | 1.53E-03 | 9.80E-15 | 461026 | 0.013% | 59.93 |
| Hand grip strength (left) | rs12914702 | 15 | 96887277 | A | G | 3.00E-01 | 1.10E-02 | 1.69E-03 | 9.30E-11 | 461026 | 0.009% | 41.96 |
| Hand grip strength (left) | rs7176095 | 15 | 74886411 | G | A | 1.28E-01 | -1.34E-02 | 2.22E-03 | 1.70E-09 | 461026 | 0.008% | 36.29 |
| Hand grip strength (left) | rs2871865 | 15 | 99194896 | G | C | 1.16E-01 | -2.18E-02 | 2.32E-03 | 5.00E-21 | 461026 | 0.019% | 88.54 |
| Hand grip strength (left) | rs13337177 | 16 | 2175323 | T | G | 1.81E-01 | -1.43E-02 | 1.94E-03 | 1.60E-13 | 461026 | 0.012% | 54.39 |
| Hand grip strength (left) | rs11642954 | 16 | 24824248 | A | G | 1.95E-01 | -1.17E-02 | 1.88E-03 | 4.50E-10 | 461026 | 0.008% | 38.88 |
| Hand grip strength (left) | rs217181 | 16 | 72114002 | T | C | 1.93E-01 | 1.20E-02 | 1.88E-03 | 2.10E-10 | 461026 | 0.009% | 40.38 |
| Hand grip strength (left) | rs9944324 | 16 | 80929342 | G | A | 4.57E-01 | -8.55E-03 | 1.50E-03 | 1.20E-08 | 461026 | 0.007% | 32.44 |
| Hand grip strength (left) | rs7197751 | 16 | 84940033 | T | G | 3.63E-01 | -9.46E-03 | 1.56E-03 | 1.40E-09 | 461026 | 0.008% | 36.67 |
| Hand grip strength (left) | rs7196917 | 16 | 69896527 | G | A | 4.30E-01 | -1.17E-02 | 1.50E-03 | 5.40E-15 | 461026 | 0.013% | 61.10 |
| Hand grip strength (left) | rs181766 | 16 | 14394878 | C | T | 3.22E-01 | 9.63E-03 | 1.60E-03 | 1.90E-09 | 461026 | 0.008% | 36.08 |
| Hand grip strength (left) | rs3814877 | 16 | 30042677 | T | G | 4.02E-01 | 1.05E-02 | 1.51E-03 | 3.40E-12 | 461026 | 0.011% | 48.46 |
| Hand grip strength (left) | rs11076004 | 16 | 53913930 | A | G | 4.18E-01 | -1.15E-02 | 1.51E-03 | 2.10E-14 | 461026 | 0.013% | 58.43 |
| Hand grip strength (left) | rs113434679 | 17 | 44126765 | A | C | 2.00E-01 | -1.49E-02 | 1.88E-03 | 1.90E-15 | 461026 | 0.014% | 63.20 |
| Hand grip strength (left) | rs755547 | 17 | 43011908 | A | G | 1.89E-01 | 1.65E-02 | 1.90E-03 | 3.20E-18 | 461026 | 0.016% | 75.74 |
| Hand grip strength (left) | rs2532111 | 17 | 62017421 | G | A | 6.40E-01 | 1.03E-02 | 1.56E-03 | 4.10E-11 | 461026 | 0.009% | 43.58 |
| Hand grip strength (left) | rs999493 | 17 | 46625519 | A | G | 6.22E-01 | 1.29E-02 | 1.54E-03 | 6.20E-17 | 461026 | 0.015% | 69.91 |
| Hand grip strength (left) | rs2587505 | 17 | 77784268 | C | T | 4.20E-01 | -9.00E-03 | 1.51E-03 | 2.30E-09 | 461026 | 0.008% | 35.70 |
| Hand grip strength (left) | rs635538 | 18 | 53273614 | A | G | 9.14E-01 | -2.17E-02 | 2.66E-03 | 3.80E-16 | 461026 | 0.014% | 66.36 |
| Hand grip strength (left) | rs4308051 | 18 | 20735461 | G | T | 7.89E-01 | 1.60E-02 | 1.82E-03 | 1.40E-18 | 461026 | 0.017% | 77.33 |
| Hand grip strength (left) | rs62081464 | 18 | 35142133 | T | C | 2.27E-01 | -9.88E-03 | 1.78E-03 | 2.80E-08 | 461026 | 0.007% | 30.83 |
| Hand grip strength (left) | rs143002906 | 18 | 12992162 | T | C | 2.78E-02 | 2.62E-02 | 4.57E-03 | 9.80E-09 | 461026 | 0.007% | 32.88 |
| Hand grip strength (left) | rs35054365 | 18 | 46612306 | A | T | 4.38E-01 | 1.28E-02 | 1.50E-03 | 1.20E-17 | 461026 | 0.016% | 73.17 |
| Hand grip strength (left) | rs10403906 | 19 | 37376756 | A | G | 4.76E-01 | -1.00E-02 | 1.49E-03 | 1.50E-11 | 461026 | 0.010% | 45.54 |
| Hand grip strength (left) | rs11669079 | 19 | 52219938 | T | A | 7.05E-01 | 1.10E-02 | 1.63E-03 | 1.90E-11 | 461026 | 0.010% | 45.07 |
| Hand grip strength (left) | rs8101782 | 19 | 12507992 | C | A | 7.03E-01 | 9.55E-03 | 1.72E-03 | 2.90E-08 | 461026 | 0.007% | 30.77 |
| Hand grip strength (left) | rs8108461 | 19 | 2186757 | C | T | 5.73E-01 | 9.52E-03 | 1.51E-03 | 2.60E-10 | 461026 | 0.009% | 39.96 |
| Hand grip strength (left) | rs143384 | 20 | 34025756 | G | A | 4.04E-01 | 2.09E-02 | 1.51E-03 | 1.50E-43 | 461026 | 0.042% | 191.54 |
| Hand grip strength (left) | rs4811040 | 20 | 48968438 | G | C | 2.77E-01 | -9.25E-03 | 1.67E-03 | 3.00E-08 | 461026 | 0.007% | 30.72 |
| Hand grip strength (left) | rs9611273 | 22 | 40534466 | T | C | 2.53E-01 | 1.08E-02 | 1.73E-03 | 4.50E-10 | 461026 | 0.008% | 38.87 |
| Hand grip strength (left) | rs6006984 | 22 | 45714937 | C | T | 2.78E-01 | 9.85E-03 | 1.65E-03 | 2.60E-09 | 461026 | 0.008% | 35.45 |

SNP: single nucleotide polymorphism; IVW: inverse-variance weighted; se: standard error of beta; BMI: body mass index.
