# Supplementary material for: Assessing the causal influence of biomechanical factors on osteoporosis risk: A multivariable Mendelian randomization investigation
Source: Medicine (Baltimore). 2026 Jul 24;105(30):e49751. doi: 10.1097/MD.0000000000049751 (PMC13406190; doi:10.1097/MD.0000000000049751)
Supplement: Supplementary file 4 [file medi-105-e49751-s004.docx]

**Supplementary table 3. Causal association between ankle spacing width and sites-specific BMD**

| **Exposure** | **Outcome** | **SNP** | **IVW** | | | **Weighted Methods** | | | | **MR egger regression** | | | | |
| --- | --- | --- | --- | --- | --- | --- | --- | --- | --- | --- | --- | --- | --- | --- |
|  |  |  |  |  |  | **Weighted Median** | | **Weighted Mode** | | **MR egger** | | **Intercept** | | |
|  |  |  | **Beta (se)** | ***P* value** | ***P* heterogeneity** | **Beta (se)** | ***P* value** | **Beta (se)** | ***P* value** | **Beta (se)** | ***P* value** | **Intercept** | **se** | ***P* value** |
|  |  |  |  |  |  |  |  |  |  |  |  |  |  |  |
| Ankle spacing width | eBMD | 366 | -0.281(0.024) | 1.43E-31 | 3.95E-32 | -0.277(0.019) | 1.80E-50 | -0.394(0.039) | 1.40E-21 | -0.449(0.058) | 1.05E-13 | 1.91E-03 | 2.21E-03 | 0.388 |
| Ankle spacing width (left) | eBMD | 194 | -0.264(0.029) | 1.69E-19 | 3.05E-68 | -0.276(0.022) | 9.27E-35 | -0.378(0.051) | 2.76E-12 | -0.412(0.078) | 3.04E-07 | 5.93E-04 | 1.48E-03 | 0.688 |
| Ankle spacing width (right) | eBMD | 207 | -0.272(0.028) | 2.61E-22 | 8.90E-46 | -0.279(0.021) | 1.15E-41 | -0.404(0.043) | 1.06E-17 | -0.398(0.077) | 4.79E-07 | 8.16E-04 | 2.35E-03 | 0.729 |
| BMI | eBMD | 502 | 0.133(0.019) | 5.69E-08 | 2.74E-132 | 0.151(0.019) | 1.56E-07 | 0.194(0.038) | 3.01E-05 | 0.138(0.052) | 0.008 | 0.001 | 8.29E-04 | 0.930 |
| Height | eBMD | 255 | -0.010(0.003) | 2.31E-06 | 6.70E-283 | -0.009(0.002) | 5.78E-05 | -0.004(0.007) | 0.599 | -0.011(0.008) | 0.143 | 0.001 | 0.002 | 0.839 |
| Hand grip strength (left) | eBMD | 156 | -0.137(0.060) | 0.022 | 1.39E-83 | -0.085(0.051) | 0.096 | -0.145(0.136) | 0.286 | -0.117(0.231) | 0.613 | 0.001 | 0.003 | 0.929 |
| Hand grip strength (right) | eBMD | 174 | -0.101(0.061) | 0.095 | 2.53E-112 | -0.089(0.049) | 0.072 | -0.153(0.128) | 0.233 | -0.455(0.225) | 0.044 | 0.004 | 0.003 | 0.104 |
| Usual walking pace | eBMD | 57 | -0.126(0.088) | 0.153 | 2.24E-09 | -0.008(0.089) | 0.928 | 0.128(0.212) | 0.550 | 0.382(0.360) | 0.293 | -0.005 | 0.003 | 0.152 |
| Ankle spacing width | FA-BMD | 336 | -0.163(0.051) | 0.001 | 5.59E-225 | -0.139(0.073) | 0.055 | -0.132(0.152) | 0.384 | -0.172(0.128) | 0.179 | 4.57E-03 | 2.22E-03 | 0.041 |
| Ankle spacing width (left) | FA-BMD | 181 | -0.108(0.056) | 0.052 | 1.00E-562 | -0.071(0.081) | 0.385 | -0.011(0.168) | 0.949 | -0.052(0.153) | 0.734 | 4.16E-03 | 1.31E-03 | 0.002 |
| Ankle spacing width (right) | FA-BMD | 195 | -0.099(0.058) | 0.086 | 1.80E-231 | -0.071(0.075) | 0.347 | -0.061(0.158) | 0.703 | -0.148(0.162) | 0.361 | 3.86E-03 | 2.19E-03 | 0.079 |
| BMI | FA-BMD | 438 | 0.046(0.054) | 0.394 | 3.26E-02 | 0.067(0.085) | 0.432 | 0.192(0.164) | 0.244 | 0.328(0.140) | 0.019 | -5.00E-03 | 0.002 | 0.029 |
| Height | FA-BMD | 230 | -0.013(0.006) | 0.019 | 1.45E-04 | -0.018(0.008) | 0.023 | -0.023(0.018) | 0.208 | -0.023(0.016) | 0.153 | 0.003 | 0.004 | 0.512 |
| Hand grip strength (left) | FA-BMD | 149 | -0.168(0.135) | 0.213 | 0.216 | -0.128(0.185) | 0.489 | -0.377(0.518) | 0.468 | -0.383(0.533) | 0.473 | 0.003 | 0.006 | 0.677 |
| Hand grip strength (right) | FA-BMD | 166 | -0.226(0.139) | 0.105 | 0.009 | -0.282(0.181) | 0.119 | -0.796(0.480) | 0.099 | -0.612(0.528) | 0.247 | 0.005 | 0.006 | 0.448 |
| Usual walking pace | FA-BMD | 54 | -0.080(0.267) | 0.763 | 0.495 | -0.169(0.393) | 0.667 | -0.641(0.923) | 0.490 | -1.326(1.084) | 0.227 | 0.012 | 0.010 | 0.241 |
| Ankle spacing width | FN-BMD | 321 | -0.090(0.031) | 0.004 | 3.36E-03 | -0.060(0.038) | 0.112 | -0.007(0.087) | 0.936 | -0.054(0.080) | 0.506 | -1.72E-03 | 4.37E-03 | 0.695 |
| Ankle spacing width (left) | FN-BMD | 171 | -0.079(0.034) | 0.022 | 7.07E-05 | -0.076(0.041) | 0.066 | -0.031(0.084) | 0.707 | -0.029(0.098) | 0.764 | 2.20E-04 | 2.82E-03 | 0.938 |
| Ankle spacing width (right) | FN-BMD | 183 | -0.095(0.033) | 0.005 | 6.01E-06 | -0.047(0.040) | 0.232 | -0.017(0.085) | 0.841 | -0.081(0.096) | 0.405 | 1.49E-03 | 4.59E-03 | 0.746 |
| BMI | FN-BMD | 470 | 0.033(0.029) | 0.255 | 1.00E-03 | 0.012(0.044) | 0.780 | -0.020(0.071) | 0.782 | 0.001(0.080) | 0.990 | 0.001 | 0.663 | 0.001 |
| Height | FN-BMD | 213 | -0.002(0.004) | 0.624 | 0.001 | -0.007(0.004) | 0.096 | -0.017(0.009) | 0.075 | -0.007(0.010) | 0.479 | 0.002 | 0.568 | 0.002 |
| Hand grip strength (left) | FN-BMD | 138 | 0.090(0.081) | 0.263 | -0.001 | 0.032(0.101) | 0.748 | -0.073(0.307) | 0.811 | 0.133(0.306) | 0.666 | 0.004 | 0.887 | 0.004 |
| Hand grip strength (right) | FN-BMD | 160 | 0.004(0.086) | 0.958 | 0.001 | -0.018(0.097) | 0.855 | -0.022(0.287) | 0.940 | -0.034(0.320) | 0.915 | 0.004 | 0.900 | 0.004 |
| Usual walking pace | FN-BMD | 48 | 0.088(0.136) | 0.517 | 0.005 | 0.197(0.196) | 0.315 | 0.364(0.463) | 0.436 | -0.405(0.545) | 0.462 | 0.005 | 0.355 | 0.005 |
| Ankle spacing width | LS-BMD | 349 | -0.024(0.030) | 0.420 | 1.22E-11 | -0.000(0.041) | 0.996 | -0.028(0.078) | 0.717 | -0.137(0.078) | 0.080 | -3.23E-03 | 2.75E-03 | 0.241 |
| Ankle spacing width (left) | LS-BMD | 188 | -0.012(0.033) | 0.726 | 6.52E-21 | -0.006(0.044) | 0.896 | -0.013(0.085) | 0.881 | -0.106(0.096) | 0.271 | -8.60E-04 | 1.75E-03 | 0.623 |
| Ankle spacing width (right) | LS-BMD | 202 | -0.061(0.036) | 0.088 | 2.44E-13 | -0.014(0.042) | 0.735 | -0.016(0.089) | 0.858 | -0.062(0.105) | 0.556 | -5.25E-03 | 2.71E-03 | 0.054 |
| BMI | LS-BMD | 450 | 0.084(0.034) | 0.012 | 3.53E-04 | 0.142(0.054) | 0.008 | 0.220(0.097) | 0.025 | 0.211(0.095) | 0.026 | -2.00E-03 | 0.001 | 0.152 |
| Height | LS-BMD | 223 | 0.002(0.004) | 0.514 | 1.81E-10 | 0.001(0.004) | 0.793 | 0.006(0.010) | 0.538 | -0.005(0.010) | 0.618 | 0.002 | 0.003 | 0.434 |
| Hand grip strength (left) | LS-BMD | 30 | 0.289(0.218) | 0.186 | 0.001 | 0.467(0.230) | 0.042 | 0.560(0.341) | 0.112 | 0.434(0.839) | 0.609 | -0.002 | 0.011 | 0.859 |
| Hand grip strength (right) | LS-BMD | 37 | 0.337(0.183) | 0.066 | 0.002 | 0.427(0.205) | 0.037 | 0.393(0.323) | 0.232 | -0.252(0.616) | 0.685 | 0.008 | 0.008 | 0.324 |
| Usual walking pace | LS-BMD | 49 | -0.028(0.171) | 0.872 | 0.264817113 | 0.166(0.237) | 0.485 | 0.282(0.482) | 0.561 | 0.909(0.794) | 0.258 | -0.009 | 0.007 | 0.233 |
| Ankle spacing width | TB-BMD | 366 | -0.102(0.027) | 1.26E-04 | 2.62E-05 | -0.141(0.028) | 4.19E-07 | -0.176(0.059) | 0.003 | -0.126(0.066) | 0.057 | 2.82E-03 | 2.69E-03 | 0.296 |
| Ankle spacing width (left) | TB-BMD | 194 | -0.069(0.029) | 0.016 | 2.01E-08 | -0.086(0.030) | 0.005 | -0.114(0.063) | 0.070 | -0.131(0.077) | 0.092 | 2.67E-03 | 1.70E-03 | 0.118 |
| Ankle spacing width (right) | TB-BMD | 206 | -0.088(0.030) | 0.003 | 3.03E-11 | -0.109(0.029) | 1.58E-04 | -0.152(0.066) | 0.022 | -0.115(0.083) | 0.167 | 2.93E-05 | 2.93E-03 | 0.992 |
| BMI | TB-BMD | 502 | 0.043(0.025) | 0.085 | 2.94E-31 | -0.093(0.137) | 0.499 | 0.027(0.031) | 0.392 | 0.070(0.066) | 0.288 | 0.001 | 0.001 | 0.653 |
| Height | TB-BMD | 255 | -0.006(0.003) | 0.057 | 2.03E-83 | -0.009(0.007) | 0.173 | -0.011(0.003) | 0.001 | -0.017(0.009) | 0.070 | 0.003 | 0.002 | 0.222 |
| Hand grip strength (left) | TB-BMD | 156 | 0.069(0.075) | 0.358 | 6.42E-24 | 0.196(0.243) | 0.422 | 0.035(0.076) | 0.651 | 0.149(0.299) | 0.618 | -0.001 | 0.003 | 0.782 |
| Hand grip strength (right) | TB-BMD | 174 | 0.023(0.072) | 0.745 | 0.001 | 0.172(0.237) | 0.470 | 0.099(0.075) | 0.190 | -0.017(0.280) | 0.952 | 0.001 | 0.003 | 0.882 |
| Usual walking pace | TB-BMD | 57 | -0.020(0.111) | 0.860 | 0.044 | 0.121(0.314) | 0.702 | 0.051(0.135) | 0.704 | -0.413(0.480) | 0.394 | 0.004 | 0.004 | 0.404 |

BMD: bone mineral density; FA: forearm; FN: femoral neck; LS: lumbar spine; eBMD: estimated heel BMD; SNP: single-nucleotide polymorphisms; IVW: inverse-variance weighted; BMI: body mass index.
